# Prognosis analysis and validation of lipid metabolism-associated lncRNAs and tumor immune microenvironment in bladder cancer

**DOI:** 10.18632/aging.204975

**Published:** 2023-08-24

**Authors:** Zhiyong Tan, Shi Fu, Jieming Zuo, Jiansong Wang, Haifeng Wang

**Affiliations:** 1 Department of Urology, The Second Affiliated Hospital of Kunming Medical University, Kunming 650101, Yunnan, People’s Republic of China; 2 Urological Disease Clinical Medical Center of Yunnan, The Second Affiliated Hospital of Kunming Medical University, Kunming 650101, Yunnan, People’s Republic of China; 3 Scientific and Technological Innovation Team of Basic and Clinical Research of Bladder Cancer in Yunnan Universities, The Second Affiliated Hospital of Kunming Medical University, Kunming 650101, Yunnan, People’s Republic of China

**Keywords:** bladder cancer, metabolic reprogramming, prognosis, immune landscape

## Abstract

Background: Numerous types of research revealed that long noncoding RNAs (lncRNAs) played a significant role in immune response and the tumor microenvironment of bladder cancer (BLCA). Dysregulated lipid metabolism is considered to be one of the major risk factors for BLCA, the study aimed to detect the lipid metabolism-related lncRNAs (LMRLs) along with their potential prognostic values and immune correlations in BLCA.

Methods: We collected lipid metabolism-related genes, expression profiles, and clinical information on BLCA from the Molecular Signature Database (MSigDB) and the TCGA database, respectively. Differentially expressed lipid metabolism genes (DE-LMRGs) and differentially expressed long non-coding RNAs (DE-lncRNAs) were selected using the limma package. Spearman correlation analysis was employed to explore the correlations between DE-lncRNAs and DE-LMRGs and to further develop protein-protein interaction (PPI) networks and perform mutational analysis. The least absolute shrinkage and selection operator (LASSO) and univariate Cox analysis were then employed to construct a prognostic risk model. The performance of the model was evaluated using Kaplan-Meier survival analysis, receiver operating characteristic (ROC) curves, and consistency indices. In addition, we downloaded the GSE31684 dataset for external validation of the prognostic signature. Moreover, we explored the association of the risk model with immune cell infiltration and chemotherapy response analysis to reveal the tumor immune microenvironment of BLCA. Finally, RT-qPCR was utilized to validate the expression of prognostic genes.

Results: A total of 48 DE-LncRNAs and 33 DE-LMRGs were found to be robustly correlated, and were used to construct a lncRNA-mRNA co-expression network, in which ACACB, ACOX2, and BCHE showed high mutation rates. Then, a risk model based on three LMRLs (RP11-465B22.8, MIR100HG, and LINC00865) was constructed. The risk model effectively distinguished between the clinical outcomes of BLCA patients, with high-risk scores indicating a worse prognosis and with substantial prognostic prediction accuracy. The model's results were consistent in the GSE31684 dataset. In addition, a nomogram was constructed based on the risk score, age, pathological T-stage, and pathological N-stage, which showed robust predictive power. Immune landscape analysis indicated that the risk model was significantly associated with T-cell CD4 memory activation, M1 macrophage, M2 macrophage, dendritic cell activation, and T-cell regulatory. We predicted that 49 drugs would perform satisfactorily in the high-risk group. Additionally, we found five m6A regulators associated with the high- and low-risk groups, suggesting that upstream regulation of LncRNA could be a novel target for BLCA treatment. Finally, RT-qPCR showed that RP11-465B22.8 was highly expressed in BLCA, while MIR100HG and LINC00865 were downregulated in BLCA.

Conclusion: Our findings suggest that the three LMRLs may serve as potential prognostic and immunotherapeutic biomarkers in BLCA. In addition, our study provides new ideas for understanding the pathogenic mechanisms and developing therapeutic strategies for BLCA patients.

## INTRODUCTION

More than 90% of cases of bladder cancer (BLCA), one of the most prevalent urological malignancies, originate from the uroepithelium. Each year, it is predicted that over 200,000 people die and over 550,000 new cases are diagnosed [1]. Currently, it ranks as the fourth most common malignancy in men and the tenth most common in women [2]. Non-muscle-invasive bladder cancer (NMIBC) and muscle-invasive bladder cancer (MIBC) are the two primary pathological subtypes of BLCA. A diagnosis of NMIBC is made in about 75% of patients, while MIBC is made in the remaining 25% [3]. For individuals with high-risk NMIBC or MIBC, according to evidence-based advice, the primary course of treatment should be radical cystectomy along with pelvic lymphadenectomy [4]. Despite the rigorous treatment that patients receive, the 5-year overall survival (OS) rate is still below average, with a median OS of just over 14 months [5]. Delay in diagnosis and ineffective treatment are two factors contributing to this poor prognosis. What’s more, the aggressiveness and extreme propensity of cancer cells to proliferate are directly associated with an unsatisfactory prognosis. Therefore, it appears that a deeper comprehension of the intricate interactions and molecular mechanisms involved in tumorigenesis is of utmost importance for BLCA.

Metabolic reprogramming is a hallmark of cancer, induced by numerous genetic or epigenetic alterations that can promote the proliferation of cancer cells [6]. It has attracted growing attention since the discovery of the Warburg effect, tumor cells can select the appropriate metabolic reprogramming to adapt to the dynamic landscape [7]. In tumorigenesis, lipids are an essential source of energy, increasing evidence points to lipid metabolism is the most noteworthy of metabolic changes ever observed [8, 9]. It is an intermediate in various metabolic activities, providing energy stores for tumor proliferation, metastasis, and progression. For example, Seo J et al. discovered that hepatocellular carcinoma progression is fueled by fatty-acid-induced FABP5 overexpression through HIF-1-driven lipid metabolism reprogramming [10]. Moreover, blocking adipogenesis inhibits the growth of glioblastoma [11]. Furthermore, lipids facilitate cell-to-cell communication in the tumor microenvironment. Su P et al. demonstrated that enhanced lipid accumulation and metabolism are imperative for tumor-associated macrophage differentiation and activation [12]. However, there is an absence of information describing how lipid metabolism is regulated in BLCA. Therefore, the discovery of genes involved in lipid metabolism may unlock novel treatment regimens for BLCA.

Long non-coding RNAs (lncRNAs) were long believed to be a component of the genome’s “dark matter” that had no biological purpose [13]. Recently, numerous studies have shown that lncRNAs interact with many substances to promote the development of tumors [14]. It controls the expression of target genes by competing with the shared miRNAs of those genes, functioning as competing endogenous RNAs. For example, lncRNA RP11-89 could promote carcinogenesis and ferroptosis resistance by sponging miR-129-5p in BLCA through PROM2-activated iron export [15]. Through the sponging of miR-490-3p and the upregulation of AURKA, LINC00958 can promote cell invasion, proliferation, and survival while suppressing apoptosis [16]. However, there is a dearth of information on the function of lipid metabolism-related lncRNAs (LMRLs) in BLCA.

In this work, comprehensive bioinformatics analyses were conducted to discover LMRGs that are predictive of the prognosis for BLCA patients using the Cancer Genome Atlas (TCGA) and Gene Expression Omnibus (GEO) datasets. The landscape of immune infiltration is described, and whether and how lipid metabolism plays a role in BLCA development is determined. To comprehend the potential molecular immunity process that might take place as BLCA progresses, we also thought carefully about the connection between lipid metabolism and invading immune cells. In conclusion, this study offers a fresh understanding that can help with the medical treatment of individuals with BLCA.

## MATERIALS AND METHODS

### Data source

We downloaded transcriptome information about BLCA from the GEO and TCGA databases. The TCGA-BLCA dataset had 19 normal samples, 414 BLCA samples, and 402 BLCA samples with information on survival. The GSE31684 dataset had 58 BLCA samples with survival information. Additionally, we were able to gather 776 genes related to lipid metabolism (LMRGs) from the Molecular Signature Database (MSigDB) [17].

### LncRNA-mRNA co-expression network construction

We used the “limma” R package (version 3.48.3) to identify differentially expressed LMRGs (DE-LMRGs) and differentially expressed lncRNAs (DE-lncRNAs) based on the expression levels of 776 LMRGs and lncRNAs in 19 normal and 414 BLCA samples from the TCGA cohort with the screening criteria of |log2FC| > 1 and adj. *P*.Val < 0.05 [18]. We then conducted Spearman correlation analysis to investigate the correlations among the DE-LMRGs and DE-LncRNAs. We selected lncRNA-LMRG relationship pairs with |R| > 0.3 and *P* < 0.05 to establish a network of lncRNA-mRNA co-expression.

### Identification of key genes, tumor mutation burden, and enrichment analysis

In this study, the STRING database was used for establishing a protein-protein interactions (PPI) network based on LMRGs in the lncRNA-LMRG co-expression network with a combined score greater than 0.4, and the LMRGs with stronger interaction strength were considered as key genes. To analyze the somatic point mutations in BLCA samples, with the help of the “maftools (2.12.0)” R package, waterfall plots were produced. Additionally, the “clusterProfiler” (Version 4.0.2) R package [19] was used to carry out the Kyoto Encyclopedia of Genes and Genomes (KEGG) and Gene Ontology (GO) enrichment analysis.

### Establishment and assessment of a risk model

Based on DE-LncRNAs in the lncRNA-LMRG network, the “survival” (version 3.2–7) R package was employed to do the univariate Cox analysis on the 402 BLCA samples in the TCGA-BLCA dataset. Then, regression analysis using the least absolute shrinkage and selection operator (Lasso) was used to screen key lncRNAs for factors with *P* < 0.05, and a Sanberry plot was constructed using key genes and key lncRNAs to demonstrate the lncRNA-LMRG co-expression relationship. Next, each sample’s risk score was determined using the formula: risk score = ∑β gene(i) × Exp gene(i) (i = 1-n), in which β represents the regression coefficient. BLCA samples in the TCGA dataset were divided into high and low-risk groups according to the median risk score. “ggplot2” (version 3.3.5) R package [20] was used to plot the risk curves for high and low-risk groups. To determine the difference in survival, Kaplan-Meier (K-M) survival curves for the two groups were plotted using the “survminer” (version 0.4.9) R package. The validity of the risk model was assessed by plotting 1-, 3-, 5- year survival Receiver operating characteristic (ROC) curves with the “survivalROC” (version 1.0.3) package. Similarly, we used the GSE31684 dataset to validate the risk model. Finally, clinical factors and risk scores were evaluated by ROC curves to analyze the correlation between clinicopathological factors, risk score, and prognostic survival of BLCA samples. Moreover, the risk model obtained from this study was compared with the published lncRNA risk model.

### Relationship between risk scores and clinical characteristics

To examine the correlations between risk scores and several clinicopathological traits, heat maps of key lncRNAs expression in clinicopathological factors were drawn. Then, risk score, age, gender, and other clinicopathological factors were included in the risk model, and independent prognostic analysis was performed by univariate Cox and multivariate Cox analysis. K-M analysis was also carried out for different clinical traits based on independent prognostic factors.

### Functional enrichment analysis between high and low-risk groups

To analyze the signaling pathway enrichment of DEGs in patients of high and low-risk groups, DEGs between two groups were screened by “limma” (version 3.48.3) R package [18]. Screening conditions were |log_2_FC| > 1, adj. *P*.Val < 0.05. The “ggplot2” package (version 3.3.5) [20] and “pheatmap” package (version 1.0.12) [21] were used to map the volcanoes and heat maps of DEGs. Ingenuity pathway analysis (IPA) was then performed on DEGs between two groups.

### Analysis of immunotherapy response and immune cell infiltration status

In this study, utilizing the Cell type Identification by Estimating Relative Subsets of RNA Transcripts (CIBERSORT) algorithm, the association between risk scores and immune cells was examined. The immune cell percentage was calculated using the CIBERSORT method, and the relationship between the risk scores and the 22 immune cells was discovered using Pearson correlation analysis. Via the Gene set variation analysis (GSVA) algorithm, the immune enrichment scores were obtained based on biomarkers expression in the IMvigor210 dataset which was generated from Charoentong’s research (IMvigor210CoreBiology package), and the samples were divided into high and low-risk groups based on the score, and the survival differences between the high and low-risk groups were compared. Then, according to the trait of treatment response of the samples (R: remission, NR: non-remission), it was evaluated how NR samples and R samples differed in the two groups.

### Analysis of the sensitivity to chemotherapy drugs

The treatment response of chemotherapeutic protocols was investigated between low- and high-risk groups using the Genomics of Drug Sensitivity in Cancer (GDSC) online website (https://www.cancerrxgene.org/). The following drug listings were taken from the GDSC website, the half maximal inhibitory concentration (IC50) value of each BLCA patient was obtained using the oncoPredict R package (version 0.2) for the drug sensitivity response evaluation, where the smaller the IC50 value of a drug, the better the ability of the drug to inhibit cell growth, that is, the more effective it is in treating cancer. In addition, using a box plot, an analysis of the expression of nine immune checkpoint inhibitors (ICIs) in the two groups was performed.

### Risk score correlation analysis with m6A moderators

N6-methyladenosine (m6A) modifications are effective biomarkers of immunotherapeutic responsiveness. Finally, we assessed the expression of regulators in high-risk and low-risk groups, using spearman analysis to examine correlations between risk score and m6A regulators.

### RT-qPCR (real-time quantitative PCR) experiments

According to the manufacturer’s recommendations, RT-qPCR was used to validate the hub gene levels. From BLCA patients who underwent radical cystectomy at Kunming Medical University’s Second Affiliated Hospital, we removed 10 cancer tissues and 10 pericarcinomatous tissues. In addition, a normal bladder uroepithelial cell line (SV-HUC-1) and BLCA cell lines (UM-UC-3, RT4, T24, 5627, SW780, and J82) were acquired from the Chinese Academy of Sciences’ Shanghai Cell Bank. Roswell Park Memorial Institute (RPMI) 1640 medium supplemented with 10% fetal bovine serum was used to cultivate these cells. Following the manufacturer’s instructions, total RNA was extracted using the TRIzol reagent (Life Technology, CA, USA) and subsequently reverse transcribed into cDNA using the PrimeScript RT Master Mix (Takara, Tokyo, Japan). As an internal control, glyceraldehyde 3-phosphate dehydrogenase (GAPDH) was utilized. We calculated the relative gene expression levels using the 2^−ΔΔCt^ method. Supplementary Table 1 includes a list of all primers utilized.

### Statistical analysis

The statistical analysis was performed using the R program (Version 4.2.0). The R packages, comparison methods, and cutoff values used for each analysis were clarified in the corresponding sections. The data from different groups were compared by the Wilcoxon test and the Kruskal-Wallis test. A *P*-value less than 0.05 was regarded as statistically significant unless otherwise stated above.

### Availability of data and materials

The analyzed datasets generated during the study are available from the corresponding author upon reasonable request.

## RESULTS

### Identification of DE-LMRGs and DE-LncRNAs between BLCA samples and normal samples

**Figure 1:**
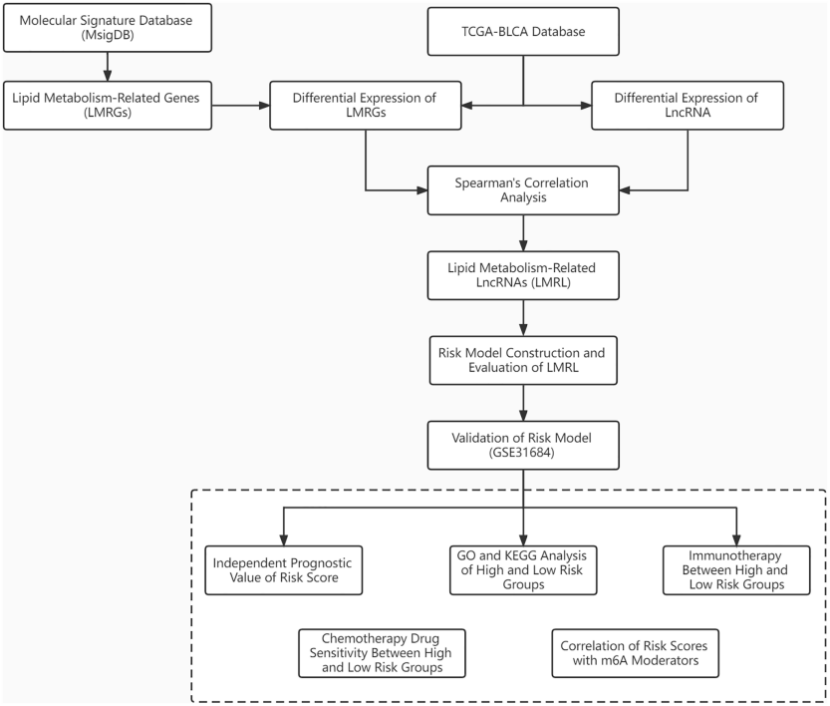
Workflow chart shows the process for identifying LMRLs-related signature and their application in BLCA.

**Figure 2:**
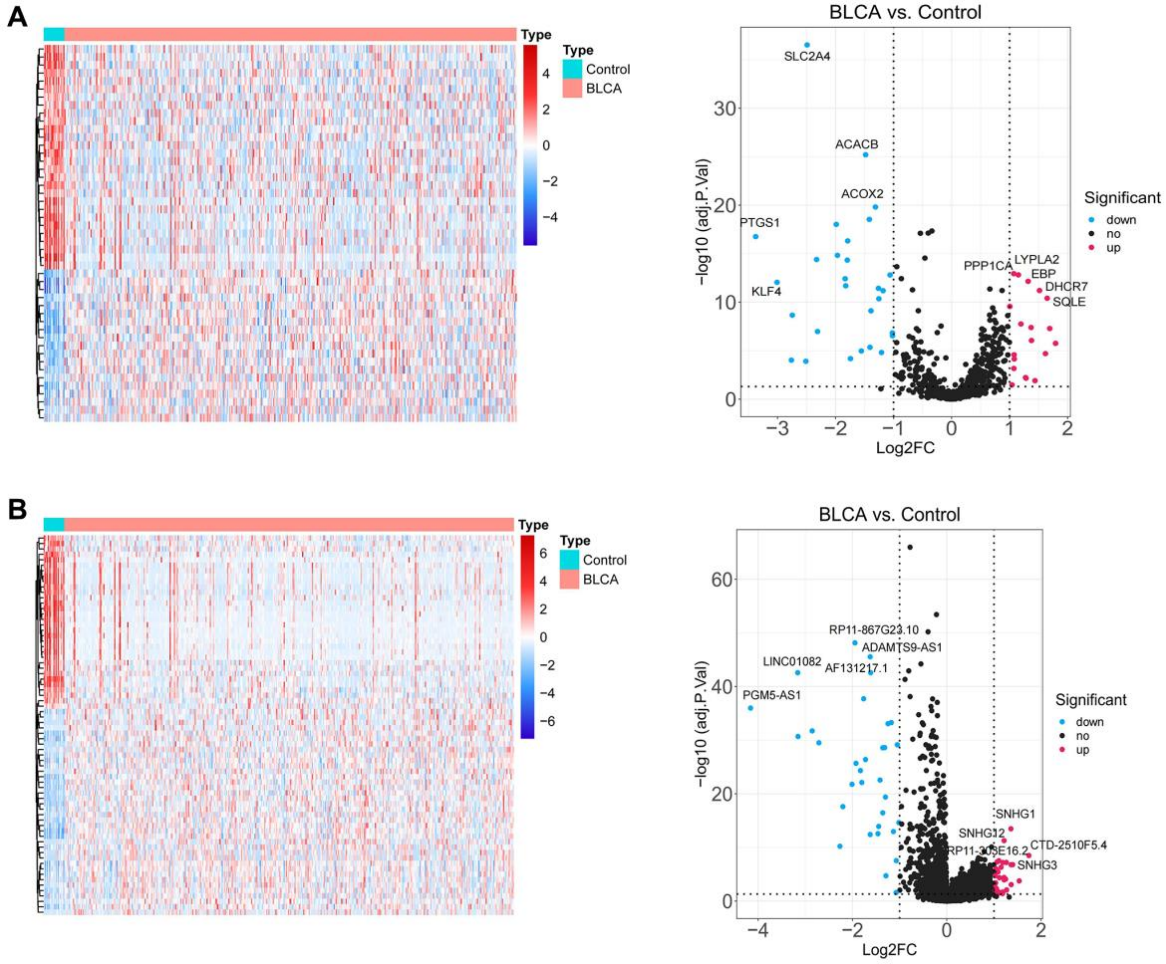
**Identification of lipid metabolism-related DEGs.** (**A**) The heatmap plot and volcano diagram show the differentially expressed LMRGs. (**B**) The heatmap plot and volcano diagram show the differentially expressed lncRNAs.

Figure 1 depicted the overall schematic layout of the current investigation. Our principle components analysis (PCA) revealed that the disease and normal groups in our dataset could be distinguished (Supplementary Figure 1). Between BLCA samples and normal samples, there were 47 DE-LMRGs, including 19 DE-LMRGs that were up-regulated and 28 DE-LMRGs that were down-regulated (Figure 2A, Supplementary Table 2). 70 DE-LncRNAs, comprising 38 up-regulated DE-LncRNAs and 32 down-regulated DE-LncRNAs, were found between BLCA samples and normal samples (Figure 2B, Supplementary Table 3).

### Identification of key DE-LMRGs and construction of the lncRNA-mRNA co-expression network

**Figure 3:**
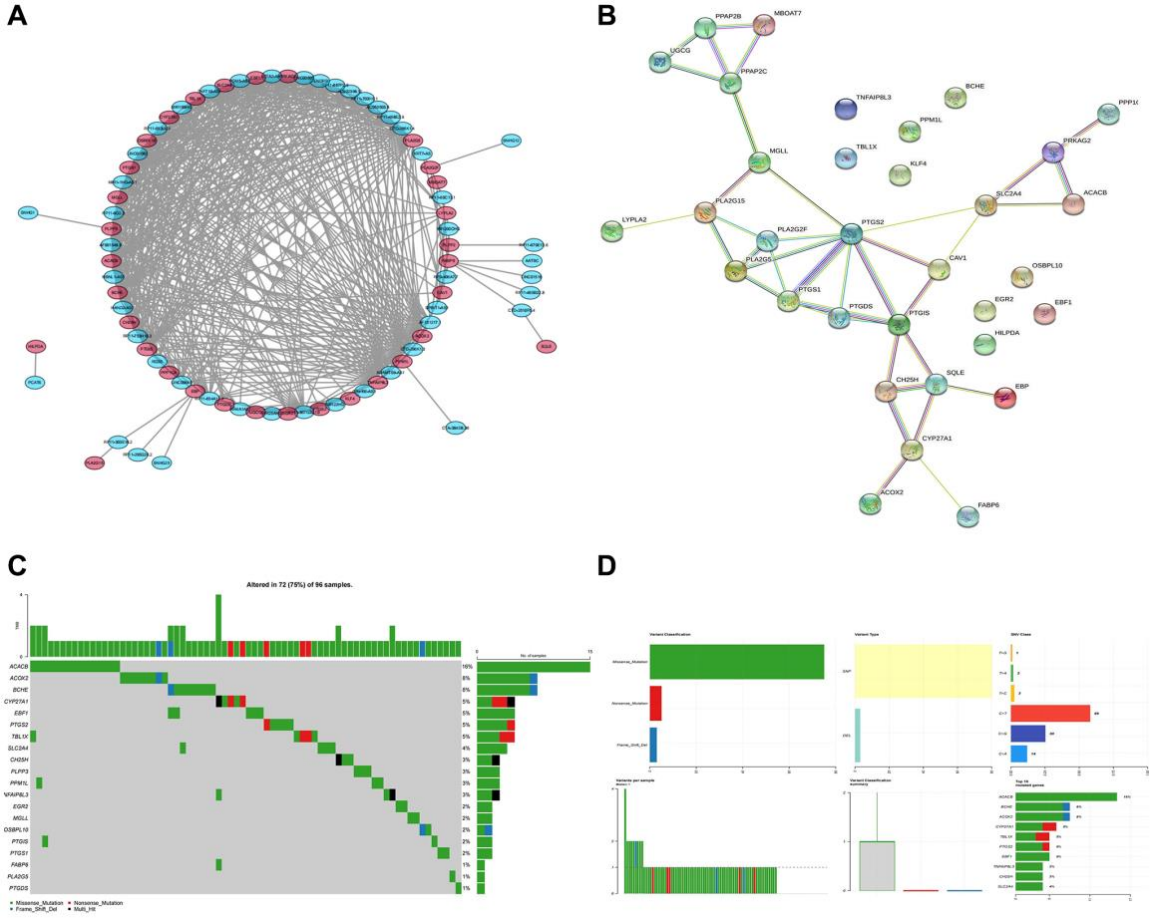
**Identification of key genes and calculation of tumor mutation burden.** (**A**) LncRNA-mRNA co-expression network. (**B**) The PPI network shows 24 genes with strong interaction. (**C**, **D**) The waterfall plot shows the mutation frequency of the LMRGs in the TCGA-BLCA cohort.

**Figure 4:**
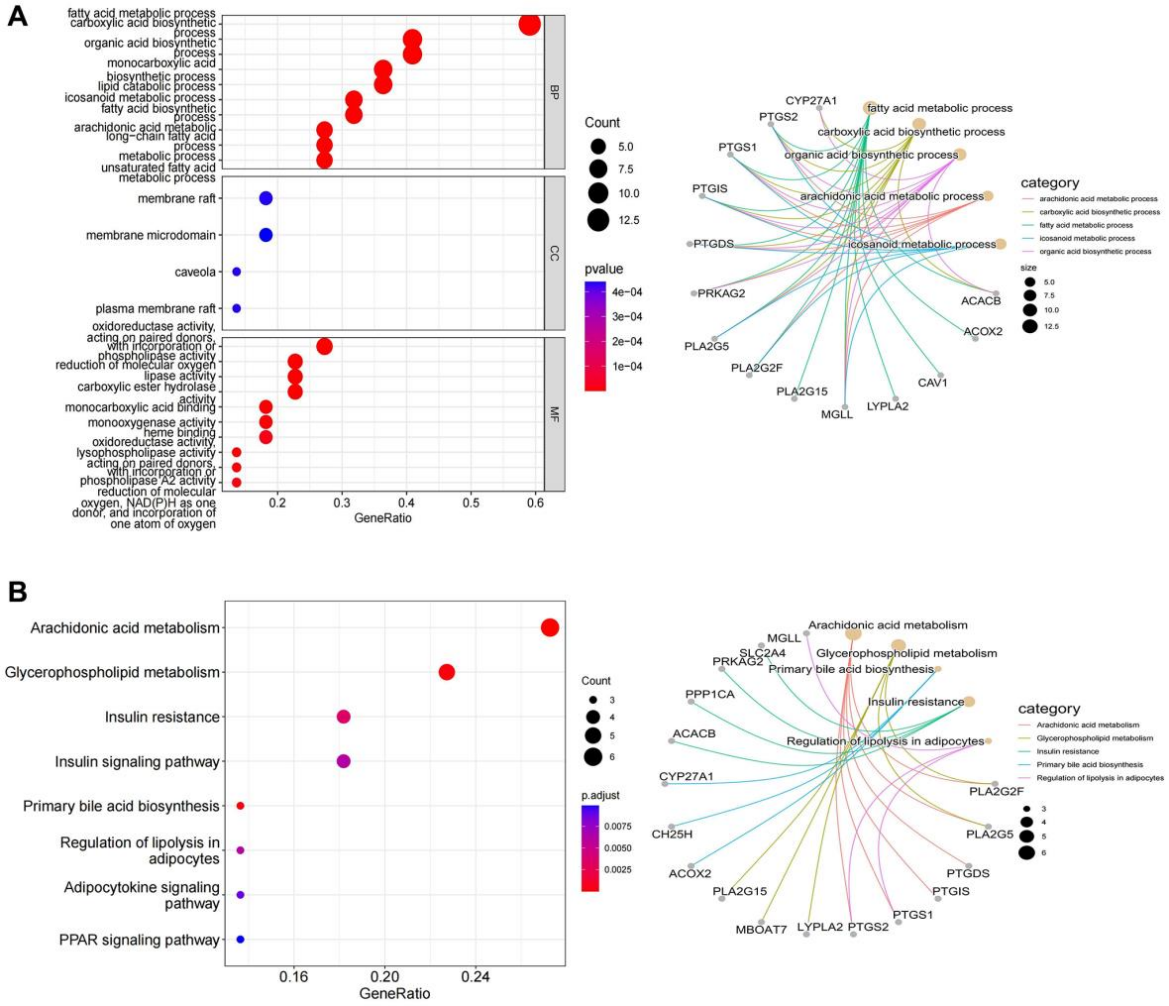
**The results of functional enrichment analysis.** (**A**) Distinctly enriched GO terms of differentially expressed LMRGs. (**B**) Significant KEGG pathway terms of differentially expressed LMRGs.

According to the spearman correlation coefficient of more than 0.3, lncRNA-mRNA co-expression networks containing 48 DE-LncRNAs and 33 DE-LMRGs were constructed (Figure 3A). The LMRGs in the co-expression network were further applied to create a PPI network with a combined score >0.4, hence 24 LMRGs with strong interaction strength were further detected as key genes (Figure 3B). Of these, the number of PTGS2 targeted genes is the largest, which it was associated with among PTGS1, CAV1, PTGIS, etc. Followed by PTGIS, it was correlated with SQLE, GH25H, GAV1, etc. Then, we found that ACACB, ACOX2, and BCHE had higher mutation frequencies (Figure 3C, 3D). Additionally, 24 key genes were enriched in 104 GO Biological Processes (BP), 4 GO Cellular Component (CC), 42 GO Molecular Functions (MF), and 16 KEGG pathways mainly involving multiple metabolism-related GO terms and KEGG pathways including arachidonic acid metabolic processes, carboxylic acid biosynthetic process, fatty acid metabolic process, and icosanoid metabolic process and pathways such as arachidonic acid metabolic process, carboxylic acid biosynthetic process, fatty acid metabolic process, and icosanoid metabolic process (Figure 4A, 4B).

### Establishment and assessment of a key lncRNAs-based-risk model

**Figure 5:**
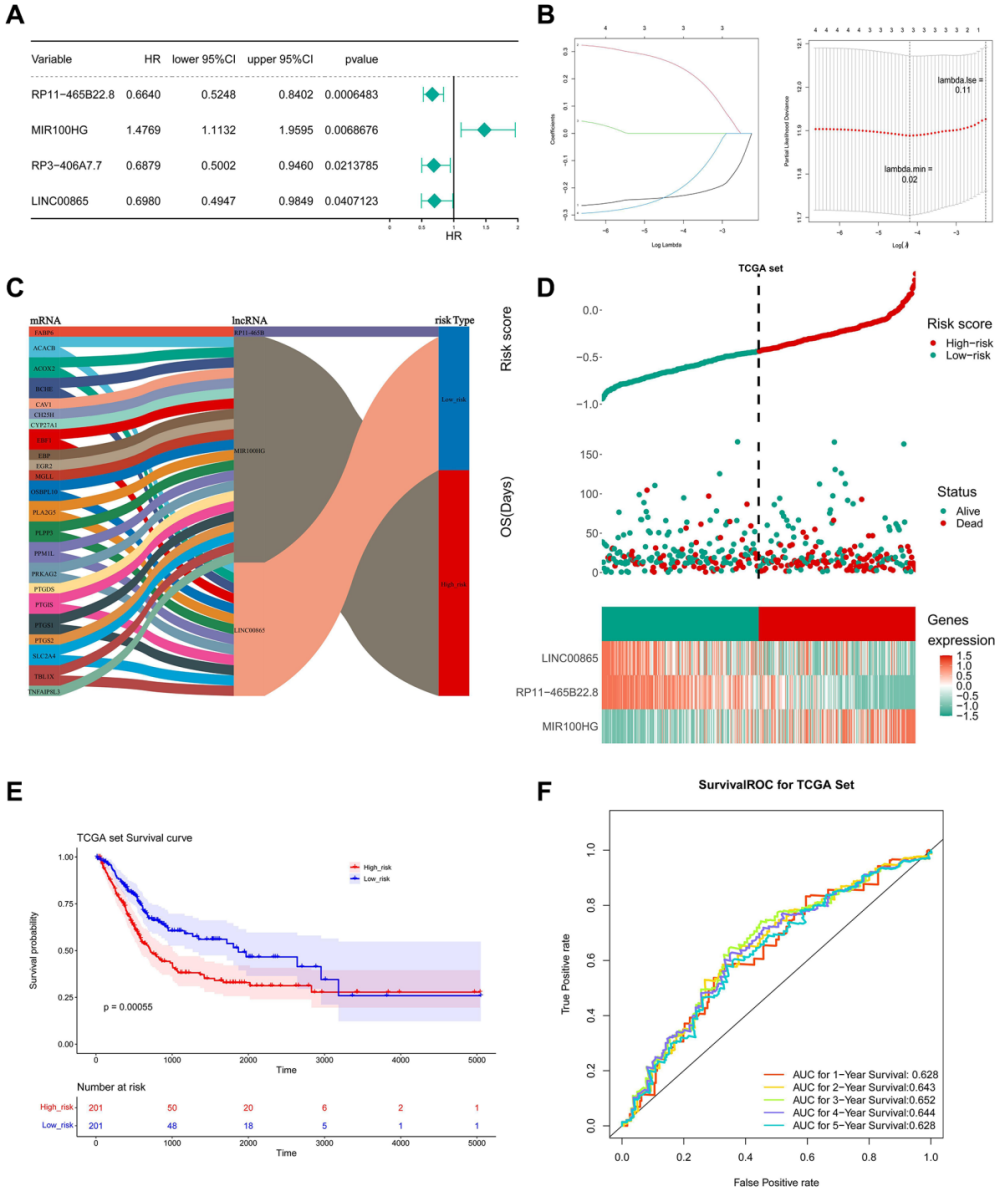
**Construction and evaluation of LMRLs-based prognostic signature.** (**A**) The LMRLs associated with the prognosis of BLCA patients were extracted by univariate Cox regression analysis. (**B**) LASSO regression analysis reserved 3 prognostic features LMRLs. (**C**) The Sanberry plot demonstrates lncRNA-mRNA co-expression relationships. (**D**) Distributions of risk score and survival status of BLCA patients and heatmap of the 3 genes signature in the training set. (**E**) KM survival curves for high and low-risk groups in the training set. (**F**) ROC curve of the 3 gene signature for predicting the 1, 3, and 5 years survival in the training set.

In the univariate Cox analysis, the *P* values of RP11-465B22.8, MIR100HG, 10RP3-406A7.7, and LINC00865 were less than 0.05 (Figure 5A). LASSO regression analysis further screened three key lncRNAs, namely RP11-465B22.8, MIR100HG, and LINC00865 (Figure 5B). 3 key lncRNAs with 23 key genes constituted the Sanberry plot in Figure 5C, indicating that ACACB, BCHE et al. were associated with LINC00865 and MIR100HG. FABP6 and RP11-465B were co-expressed in the low-risk group.

**Figure 6:**
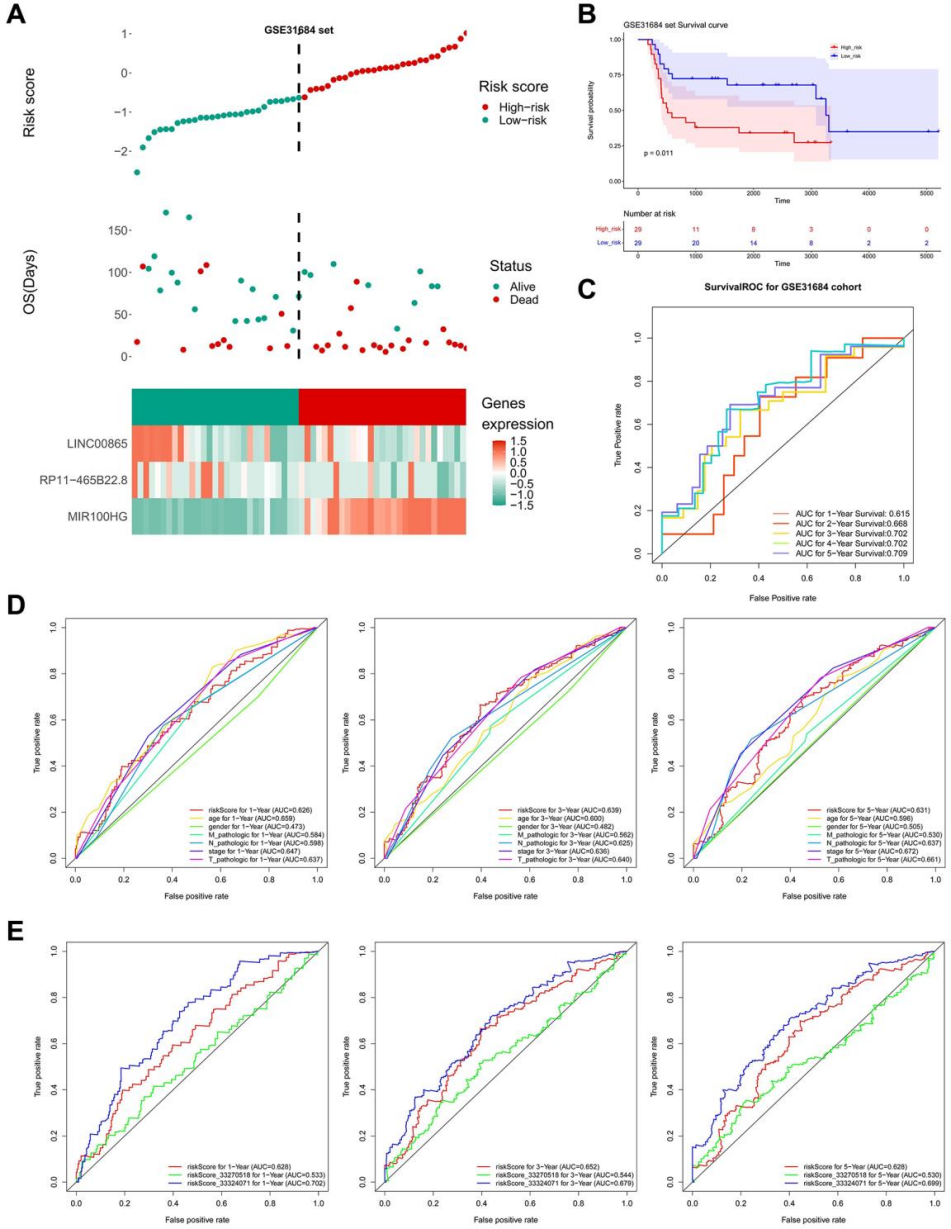
**Validation of prognostic model.** (**A**) High and low-risk group curves and the heat map of the model in the validation set. (**B**) KM survival curve of high and low-risk groups in the validation set. (**C**) Survival ROC curve of the validation set. (**D**) ROC curve for clinicopathological factors and risk score of patients at 1, 3, and 5 years. (**E**) Comparing the risk model with other models.

Simultaneously, the risk score in the TCGA dataset was calculated using the LASSO coefficients of three important lncRNAs, and the median risk score was used to divide the BLCA samples into high- and low-risk groups. In the low-risk group, it was evident that LINC00865 and RP11-465B22.8 were significantly expressed, while MIR100HG was highly expressed in the high-risk group (Figure 5D). Compared to people with low-risk scores, the prognosis for high-risk persons was poorer (Figure 5E). The under the curve (AUC) values of ROC analysis were consistently larger than 0.6, showing that the risk model could effectively predict the survival of BLCA (Figure 5F). The outcomes in the GSE31684 dataset were in agreement with those in the TCGA dataset (Figure 6A–6C).

For the comparison of predictive capabilities of the key lncRNAs-based-risk model and clinical characteristics, ROC results suggested that among risk score, stage, and T-stage had gentle performances at 1, 3, and 5 years (Figure 6D). Figure 6E showed that the model obtained in this study did not differ much from the prediction of previous models in the literature, however, given the streamlining of the model genes (from eight to three), it was believed that our model will benefit further exploration of the BLCA prognosis.

### Relationship between risk score and clinical characteristics

**Figure 7:**
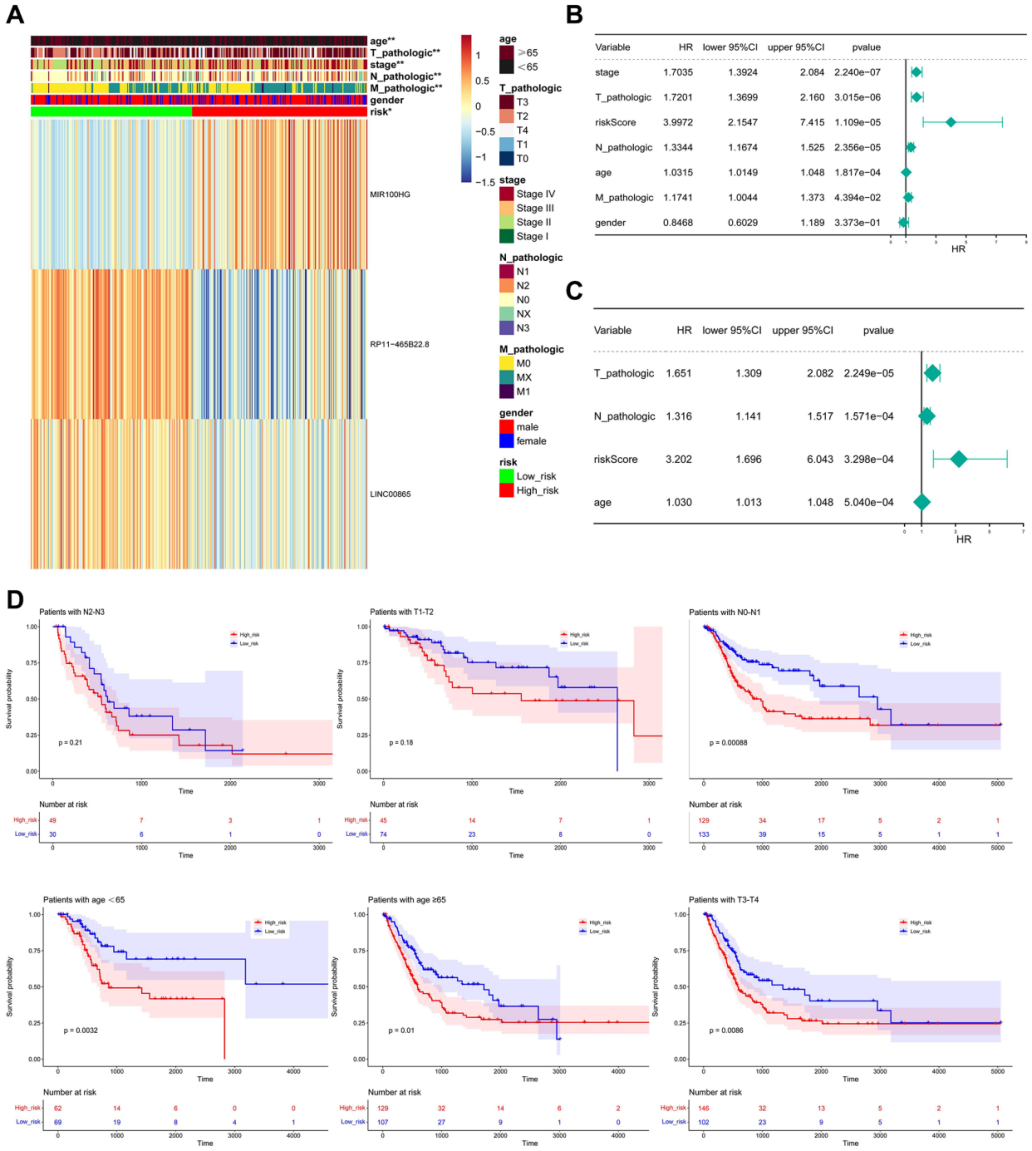
**Relationship between risk score and clinicopathological characteristics.** (**A**) The heatmap shows the relationship among gender, age, grade, T stage, N stage, M stage, tumor stage, and risk score. (**B**, **C**) Univariate and multivariate assays identified independent prognostic factors in BLCA patients. (**D**) KM survival curve for high and low-risk groups in independent prognostic factors.

Considering the important prognostic significance of clinical characteristics, the correlation of risk score and clinical characteristics was explored, where the expression of MIR100HG, PR11-465B22.8, and LINC00B85 in different clinical characteristics was exhibited in Figure 7A. Crucially, risk score, T-stage, N-stage, and age were selected as the independent prognostic factors (Figure 7B, 7C). The outcomes of the hierarchical analysis showed that in the age <65, age >65, N0-N1 period, T3-T4, the survival rate of individuals in the high-risk group was lower (Figure 7D).

### Analysis of functional enrichment in high- and low-risk groups

**Figure 8:**
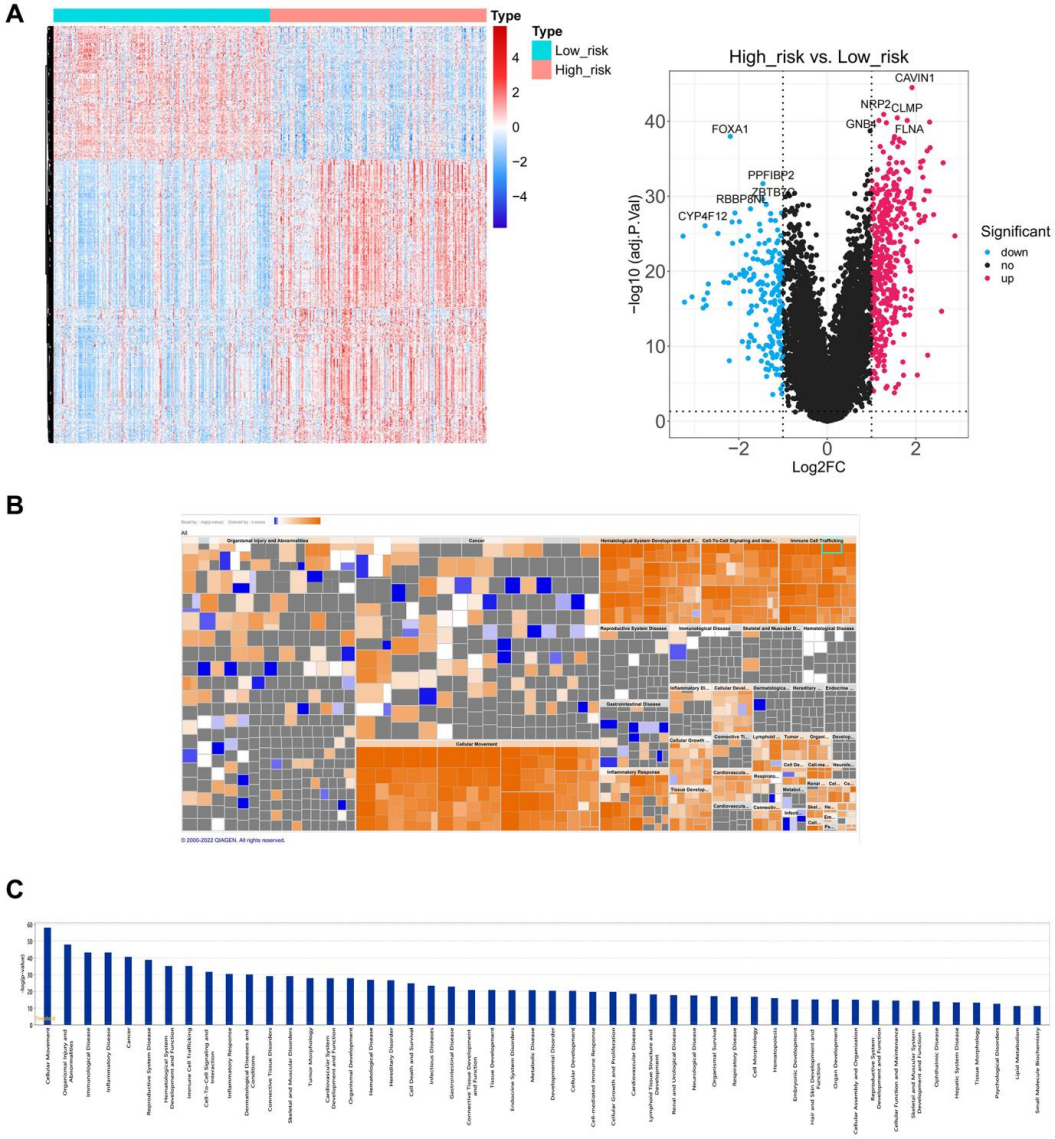
**Functional enrichment analysis between high and low-risk groups.** (**A**) The heatmap plot and volcano diagram show the DEGs between patients in high and low-risk groups. (**B**, **C**) IPA analysis shows 620 DEGs significantly acted in 50 pathways.

Furthermore, different biological significances in the two risk groups were investigated through the IPA functional enrichment analysis. Between the two groups, 620 DEGs were evaluated (Figure 8A). IPA analysis showed that 620 DEGs significantly acted in 50 pathways, including cellular movement, organismal injury, abnormalities, immunological disease, etc. (Figure 8B, 8C).

### Evaluation of immune cell infiltration status and treatment response

**Figure 9:**
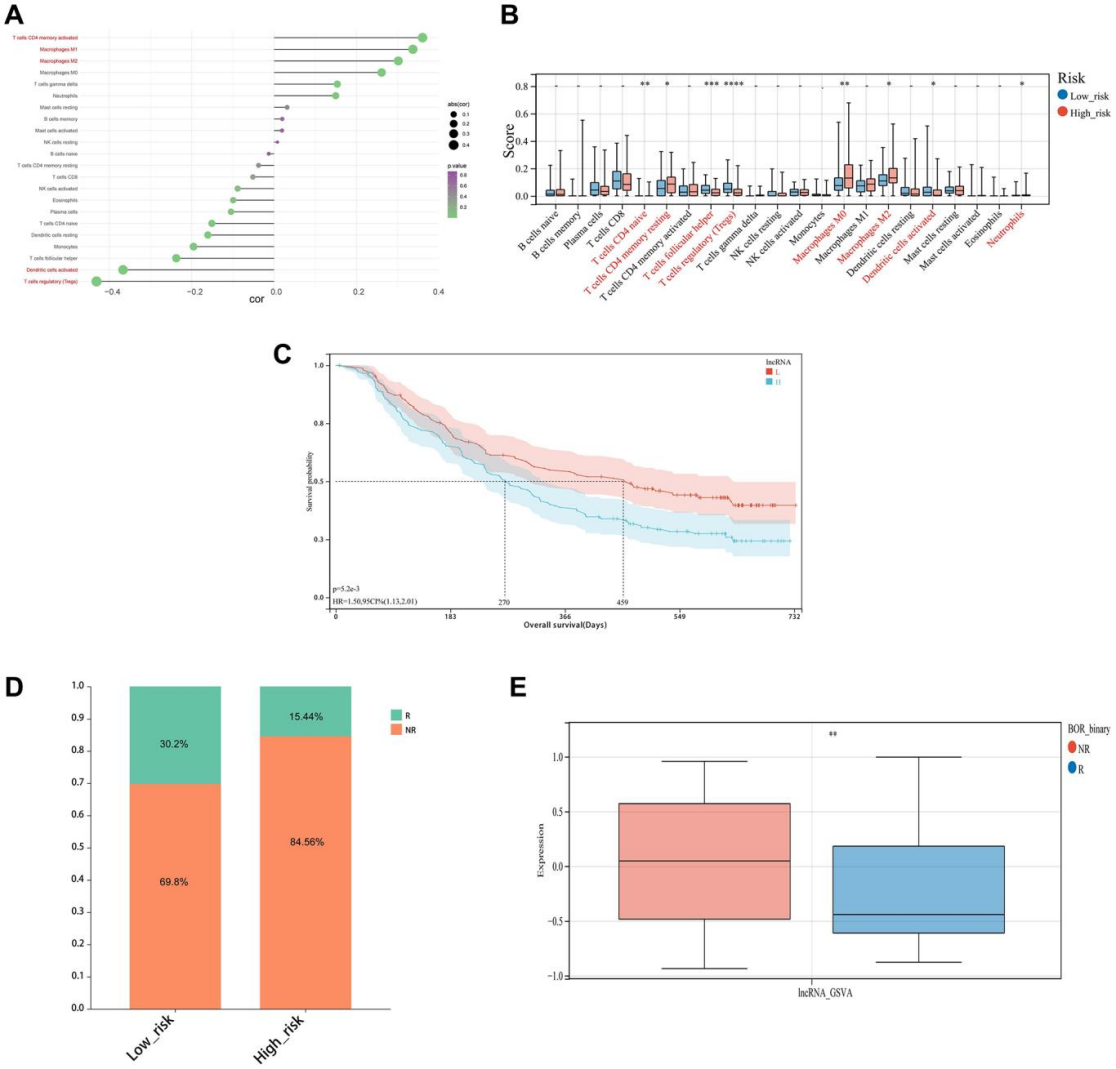
**Immune infiltration analysis and immunotherapy response.** (**A**) Correlation chart between risk score and immune infiltration cells. (**B**) The relative proportions of 22 kinds of immune cells in the two risk subgroups. (**C**) KM survival curves for overall survival between the high and low-risk groups in the IMvigor210 cohort. (**D**, **E**) Differences of non-responders and responders to immunotherapy response between high and low-risk groups in the IMvigor210 cohort.

**Figure 10:**
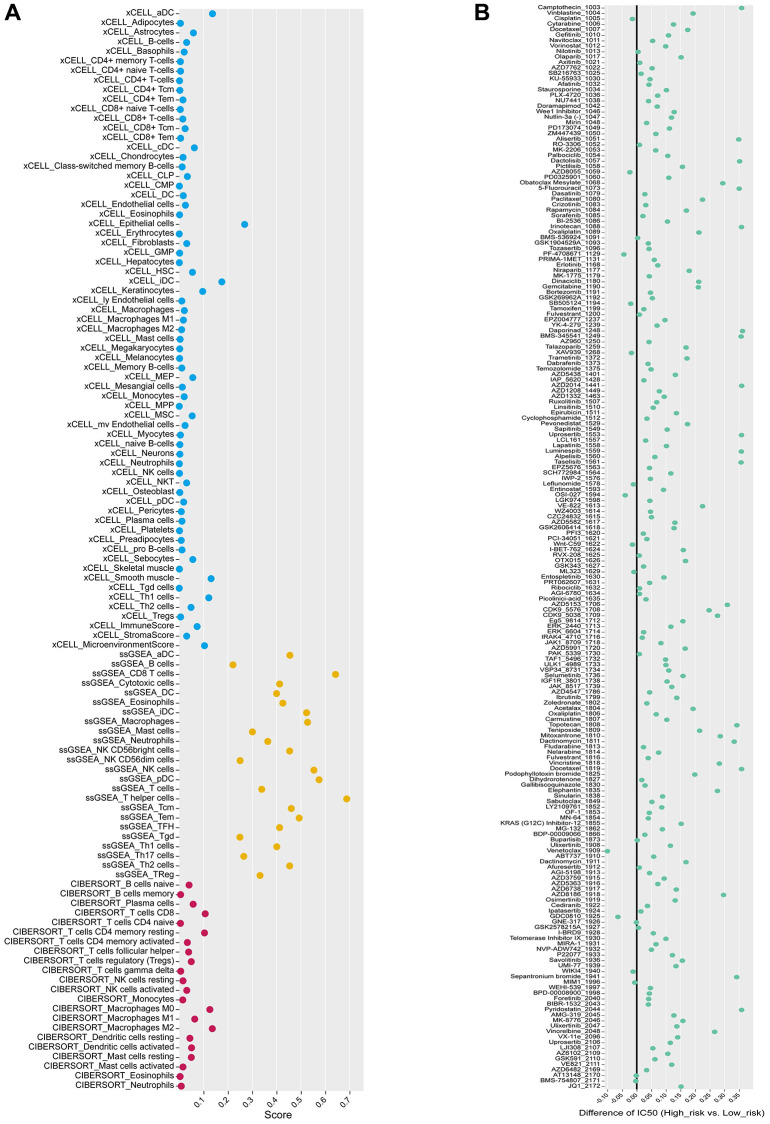
**Analysis of immune landscape and drug sensitivity.** (**A**) Three algorithms (xCELL, ssGSEA, and CIBERSORT), labeled by different colors, are applied to quantify immune-infiltration cells in the two risk score groups from the IMvigor210 cohort. (**B**) Drugs with significant differences between high and low-risk groups are presented in the bubble plot.

Analysis for immune infiltration correlation of risk score was a common and important approach to explore the potential immunotherapy targets related to the key lncRNAs. The risk score was found to have a strong positive correlation with T cells CD4 memory activation, Macrophages M1, and Macrophages M2, and a weak negative correlation with Dendritic cell activation and T cells regulatory (Tregs) (Figure 9A, 9B). Besides, the IMvigor210CoreBiology cohorts, an immunotherapy cohorts associated package, was conducted in this study, where the difference in survival between the high-risk and low-risk groups according to the GSVA score was considerable (Figure 9C). And meanwhile, the frequency of NR samples was larger among the samples from the high-risk group, and their GSVA scores were significantly different from R samples, demonstrating that the patient’s immunotherapy treatment was highly correlated with the risk model (Figure 9D, 9E). Moreover, we employed the xCELL, ssGSEA, and CIBERSORT algorithms to assess the proportion of each type of immune cell, and we discovered that the ssGSEA algorithm’s assessment of the immune cell proportion was outstanding (Figure 10A).

### Analysis of the sensitivity to chemotherapy drugs

**Figure 11:**
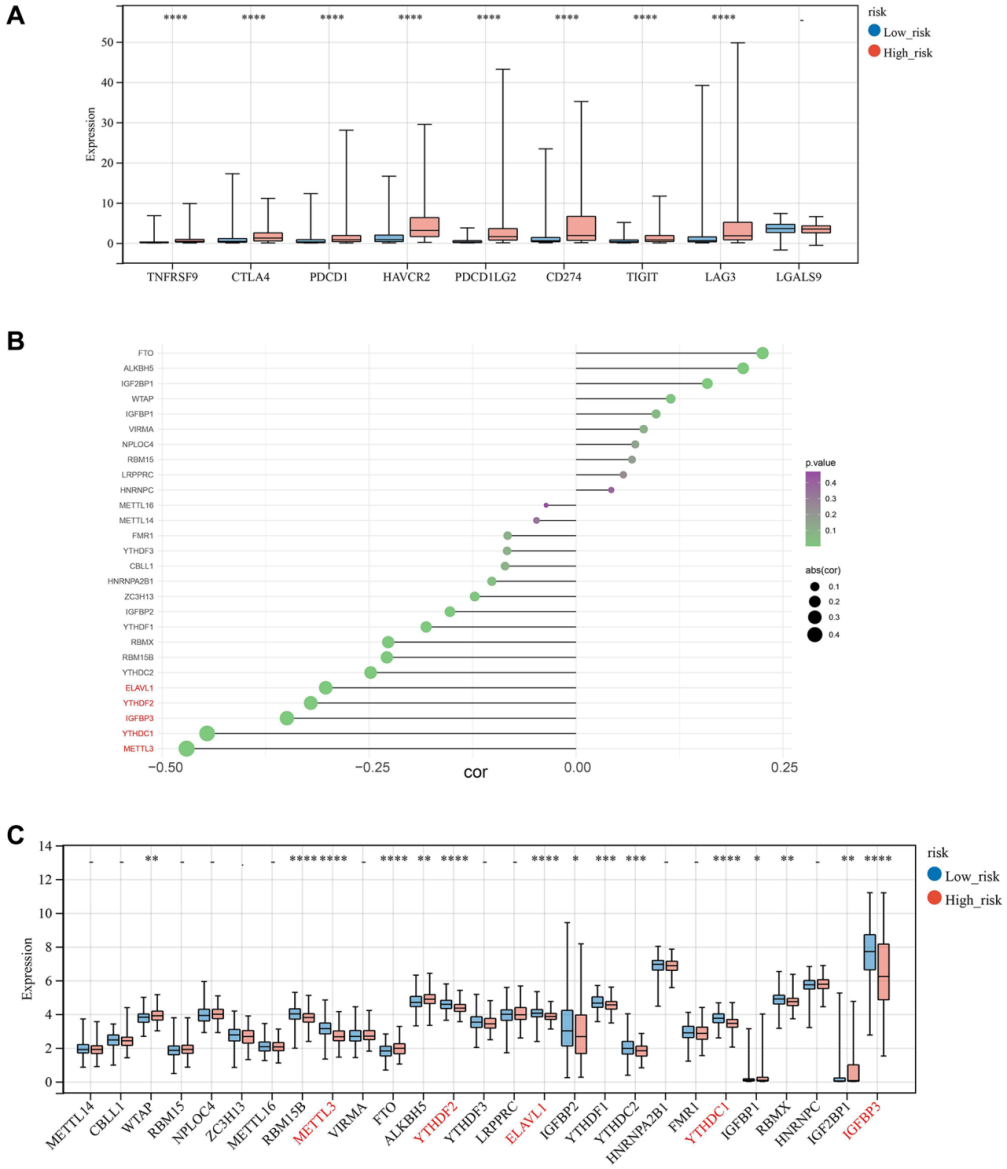
**Correlation analysis of risk score with m6A moderators.** (**A**) Expression of Immune-checkpoint inhibitors in high and low-risk groups. (**B**, **C**) The correlation and relative proportions between risk score and m6A moderators in the two risk subgroups.

For the potential treatment response of chemotherapeutic protocols in BLCA, the IC50 values for 147 out of 198 anticancer drugs differed significantly between the high and low-risk groups, with the high-risk group being more sensitive to 49 drugs, such as 5-fluorouracil 1073, and AGI 5198 1913 (Supplementary Figure 2). The 98 medicines were more responsive to the low-risk group (ABT737 1910, AFATINIB 1032, etc.) (Supplementary Figure 3). In addition, we examined the variances in IC50 values between the two groups by averaging the IC50 values (Figure 10B). In the high-risk and low-risk groups, we examined the sensitivity of nine immune checkpoint inhibitors (ICIs). The expression of 8 ICIs, TNFRSF9, CTLA4, PDCD1, HAVCR2, PDCD1LG2, CD274, TIGIT, LAG3, and LGALS9, differed significantly between two groups (Figure 11A).

### Correlation analysis of risk score with m6A moderators

As the effective prognostic and immunotherapy response-related biomarkers, the correlations of risk score and five m6A regulators, (ELAVL1, YTHDF2, IGFBP3, YTHDC1, and METTL3) were analyzed, demonstrating that these regulators have a strong negative correlation with risk values (Figure 11B), and the low-risk group displayed increased expression of these five regulators. In addition, WTAP, RBM15B, FTO, ALKBH5, IGFBP2, YTHDF1, YTHDC2, IGFBP1, RBMX, and IGF2BP1 were significantly different between the two groups (Figure 11C).

### Using RT-qPCR to verify the model gene expression

**Figure 12:**
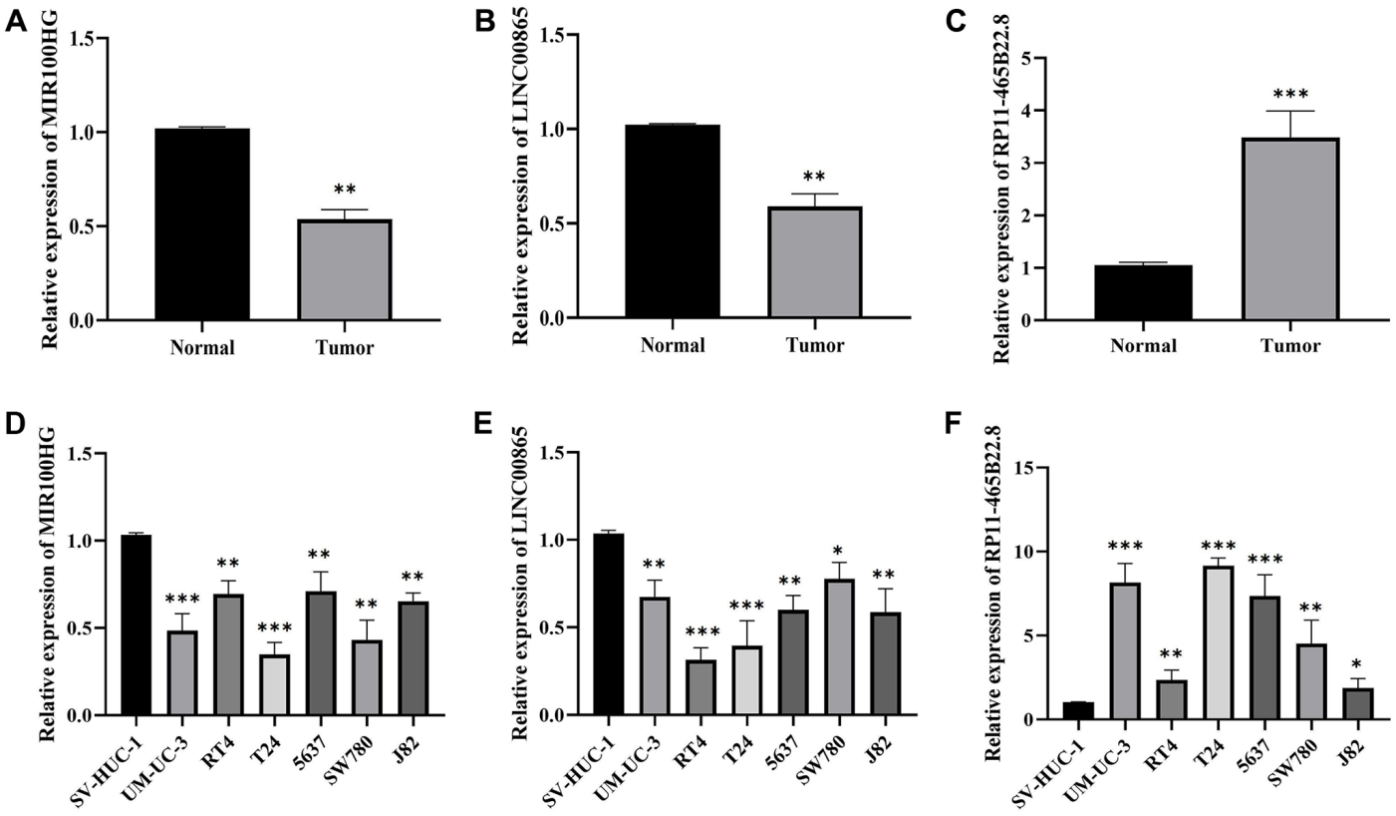
**The expression of MIR100HG, LINC00865 and RP11-465B22.8 in tissues and cell lines of BLCA detected by RT-qPCR.** (**A**) The expression of MIR100HG in BLCA tissues. (**B**) The expression of LINC00865 in BLCA tissues. (**C**) The expression of RP11-465B22.8 in BLCA tissues. (**D**) The expression of MIR100HG in BLCA cell lines. (**E**) The expression of LINC00865 in BLCA cell lines. (**F**) The expression of RP11-465B22.8 in BLCA cell lines.

We found significantly lower expression of MIR100HG and LINC00865 in normal cell lines and tissues of BLCA (Figure 12A, 12B, 12D, 12E). However, RP11-465B22.8 expression was significantly higher in BLCA tissues and cell lines (Figure 12C, 12F). The expression trends of the pivotal genes were matched with the TCGA transcriptome data. Therefore, we hypothesize that MIR100HG, LINC00865, and RP11-465B22.8 can be considered reliable and accurate model genes for BLCA.

## DISCUSSION

One of the most prevalent cancers in the world is BLCA, and its prevalence is rising in many nations. Although there have been advances in the management of BLCA over the past few years, the disease’s heterogeneous and aggressive nature has led to the failure of TNM staging to accurately predict patient prognosis. For patients with locally advanced disease, the prognosis remains poor [22]. Due to the heterogeneous molecular expression of BLCA, current diagnostic methods are limited in their ability to assess prognosis. Therefore, it is crucial and urgent to screen new biomarkers to create patient-specific medicines and enhance prognosis. Lipids are well-known to be important components of biological membranes and structural units of cells, playing a crucial role in cellular activity. Growing research has shown that lipid metabolic dysregulation is directly related to the emergence of inflammation, cancer, and medication resistance [23]. Recent research indicates that all metabolic pathways, including those for glucose, amino acids, nucleotides, and other pathways, may function as possible prognostic biomarkers for BLCA [24], on their molecular-level metabolic control, few investigations have been conducted.

Bioinformatics analysis was used in the current work to thoroughly analyze the potential processes and prognostic significance of LMRGs in BLCA. Based on Spearman correlation analysis, there was discovered to be a strong correlation between 33 differentially expressed mRNAs and 48 differentially expressed lncRNAs. These were used to create a network of lncRNA and mRNA co-expression, in which ACACB, ACOX2, and BCHE exhibited high mutation rates. We then performed PPI network and functional enrichment analyses to investigate the biological functions of LMRGs in BLCA. The GO and KEGG analyses revealed that LMRGs were associated with fatty acid metabolism, carboxylic acid biosynthetic membrane rafts, phospholipase activity, arachidonic acid metabolism, and the PPAR signaling pathway. Furthermore, we established a novel signature for LMRLs in a large-scale BLCA cohort, including a test dataset and a validation dataset. This revealed its successful prediction of BLCA patients’ prognosis and its potential applicability to clinical traits and the immunological microenvironment.

Recently, the construction of predictive models based on datasets related to specific biological features has shown advantages in the prognostic assessment of various malignancies. It is known that accurate prognosis prediction by classifying patients into two groups based on reliable predictive characteristics improves the ability of clinicians to carry out personalized treatment decisions [25, 26]. In the present study, RP11-465B22.8, MIR100HG, and LINC00865 were identified as the best prognostic LMRGs, and a risk model was constructed, most of which are associated with the development of tumors. For example, a MIR100HG/hnRNPA2B1/TCF7L2 feedback loop may be activated by the miRNA-host gene lncRNA MIR100HG, a powerful inducer of epithelial-to-mesenchymal transition in colorectal cancer, and may help explain cetuximab resistance and metastasis [27]. In addition, MIR100HG may be one of the immune-related prognostic lncRNA signature genes for BLCA, according to research by Luo WJ et al. This discovery offers BLCA patients a new target for customized immunotherapy [28]. Furthermore, it was discovered that RP11-465B22.8 promoted esophageal cancer cell proliferation, migration, and invasion while RP11-465B22.8 knockdown had the opposite impact. RP11-465B22.8 was demonstrated to function mechanistically as a miR-765 sponge to boost the expression of KLK4. Additionally, it was discovered that exosomes may transport LncRNA RP11-465B22.8 from esophageal cancer cells to macrophages. Subsequently, this administration triggered cell migration and invasion brought on by M2 macrophages [29]. Ma M et al. showed that high-risk groups based on the LINC00865 signature gene may benefit more from immunotherapy [30]. Finally, we have found that risk score is an independent survival predictive factor that can separate clinical outcomes in BLCA patients, with higher risk scores indicating a worse prognosis. The three LMRGs-based risk scores also had significant prognostic prediction accuracy, which was further validated in the external dataset GSE31684 and *in vitro* experiments.

With a better understanding of the mechanisms of tumor development, it is now believed that tumor development is not solely the result of oncogene amplification or suppressor gene deletion, but that the surrounding environment also plays an important role [31]. The tumor microenvironment (TME) is a complex intra-tissue environment that interacts with the tumor and surrounding tissues, creating an environment that promotes tumor cell development, growth, and metastasis. Mounting evidence suggests a strong correlation between cancer prognosis and the TME, particularly concerning immune cells and their interactions with cancer cells [32]. Our findings showed that the risk model and five immune cells had a strong association: dendritic cell activation, M1 and M2 macrophages, T-cell regulatory (Tregs), and T-cell CD4 memory activation. Previous studies have reported that increased lipid accumulation within tumor-associated dendritic cells can lead to adverse stimulation of T-cell responses by reducing antigen presentation. Ultimately, this contributes to enhanced immunosuppression within the TME [33, 26]. Regarding the effect of Tregs, it is important to acknowledge their key role in the immunosuppressive TME of dysregulated lipid metabolism. Tregs may secrete regulatory cytokines such as IL-10 and TGF-β, which help to maintain the immunosuppressive microenvironment and facilitate cancer cells in evading immune surveillance [34]. Moreover, our findings are consistent with the view that tumor-associated macrophages are crucial immunosuppressive cells that promote tumorigenesis and metastasis. M2 macrophages have been identified as important promoters of tumor progression and have been connected to negative outcomes, in contrast to M1 macrophages, which have pro-inflammatory and anti-tumor activities [35]. Taken together, our results are consistent with previous research and provide novel insights into effective cancer immunotherapy. They further propose that by triggering immune cell infiltration and immunological responses, aberrant lipid metabolism may aid in the development of BLCA.

Several studies have pointed out that chemotherapy and immunotherapy are considered to be essential adjuvant treatment options for BLCA [36]. In this study, the BLCA cohort was divided into two groups using GSVA. We discovered that patients in the high-risk group had a worse prognosis and that they were more susceptible to immunotherapy, suggesting that the risk model can be effective in assessing patient outcomes. Moreover, among 198 common chemotherapeutic drugs, we screened 49 drugs that performed satisfactorily in the high-risk group. For example, the combination of Irinotecan and Gemcitabine is an effective treatment for patients with metastatic BLCA, and radiotherapy regimens with the addition of 5-Fluorouracil are a relatively well-established treatment option for BLCA [37]. In this study, high-risk patients were more responsive to treatment with Irinotecan and 5-Fluorouracil. In addition, Camptothecin showed strong cytotoxicity *in vitro* and *in vivo* against a wide range of tumor types and its sensitivity response in this study was excellent in the low-risk group [38]. For the eight immune checkpoint inhibitors, we also discovered substantial variations between the two groups. For example, compared to CD28, CTLA4 has a higher affinity and can initiate a cascade of events leading to the suppression of T-cell responses. In addition, Treg cells constitutively express CTLA4, which further plays a key role in suppressing anti-tumor immunity [39]. When T cells are stimulated, LAG3 is expressed on the surface of many different lymphocytes and rapidly appears on the surface. This reduces the tumor immunological milieu by speeding T cell depletion and reducing T cell proliferation [40]. These intriguing results show that the immune checkpoint profiles of patients in the two groups differ, and our model can identify patients who are suited for therapy with particular checkpoint inhibitors. Moreover, N6-methyladenosine (m6A) modifications have been reported as powerful prognostic and immunotherapeutic biomarkers that can affect the splicing and maturation of ncRNAs, ultimately leading to tumorigenesis [41]. As a result, we used spearman analysis to examine the relationship between risk score and m6A regulators. According to our findings, the risk scores of five modifiers—ELAVL1, YTHDF2, IGFBP3, YTHDC1, and METTL3—were strongly correlated with each other. This suggests that upstream regulation of LncRNAs could be a novel therapeutic approach for treating BLCA.

In summary, we developed and validated a new LMRG signature to predict the survival of BLCA. The aberrantly expressed LMRGs selected in this study could serve as prognostic biomarkers for BLCA patients. However, there are still limitations to consider when interpreting these results. Although it is important to be able to forecast how an immune checkpoint inhibitor will react, further study is required to understand their precise mechanisms and biological functions in BLCA. Furthermore, this retrospective study cannot rule out the possibility of selection bias, and additional research is required to verify our findings.

## CONCLUSIONS

We were able to successfully identify a signature of LMRGs in our investigation that can forecast the general survival of BLCA patients. Our signature's substantial predictive value was confirmed in a separate cohort, proving it. These findings imply that LMRGs have a significant impact on BLCA development and may be a valid indicator of therapy effectiveness. Our research opens up new possibilities for the future treatment of BLCA with precision.

## Supplementary Materials

Supplementary Figure 1

Supplementary Figure 2

Supplementary Figure 3

Supplementary Tables
