# Supplementary material for: Prognosis analysis and validation of lipid metabolism-associated lncRNAs and tumor immune microenvironment in bladder cancer
Source: Aging (Albany NY). 2023 Aug 24;15(16):8384–407. doi: 10.18632/aging.204975 (PMC10496992; doi:10.18632/aging.204975)
Supplement: Supplementary Figure 1 [file aging-15-204975-s001.pdf]

SUPPLEMENTARY FIGURE

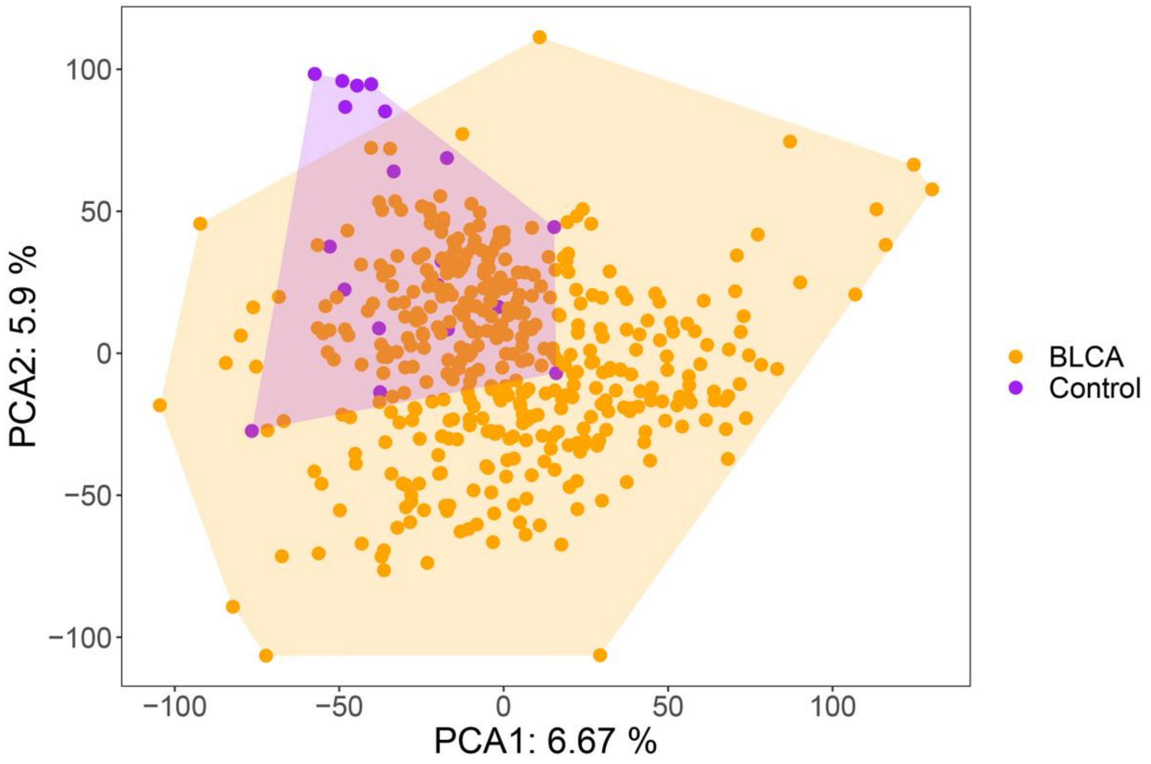

Supplementary Figure 1. Principal components analysis of tumor and normal sample in the TCGA dataset.
