# Supplementary material for: Prognosis analysis and validation of lipid metabolism-associated lncRNAs and tumor immune microenvironment in bladder cancer
Source: Aging (Albany NY). 2023 Aug 24;15(16):8384–407. doi: 10.18632/aging.204975 (PMC10496992; doi:10.18632/aging.204975)
Supplement: Supplementary Figure 2 [file aging-15-204975-s002.pdf]

Supplementary Figure 2. 49 chemotherapeutic drugs were sensitive in the high-risk group.

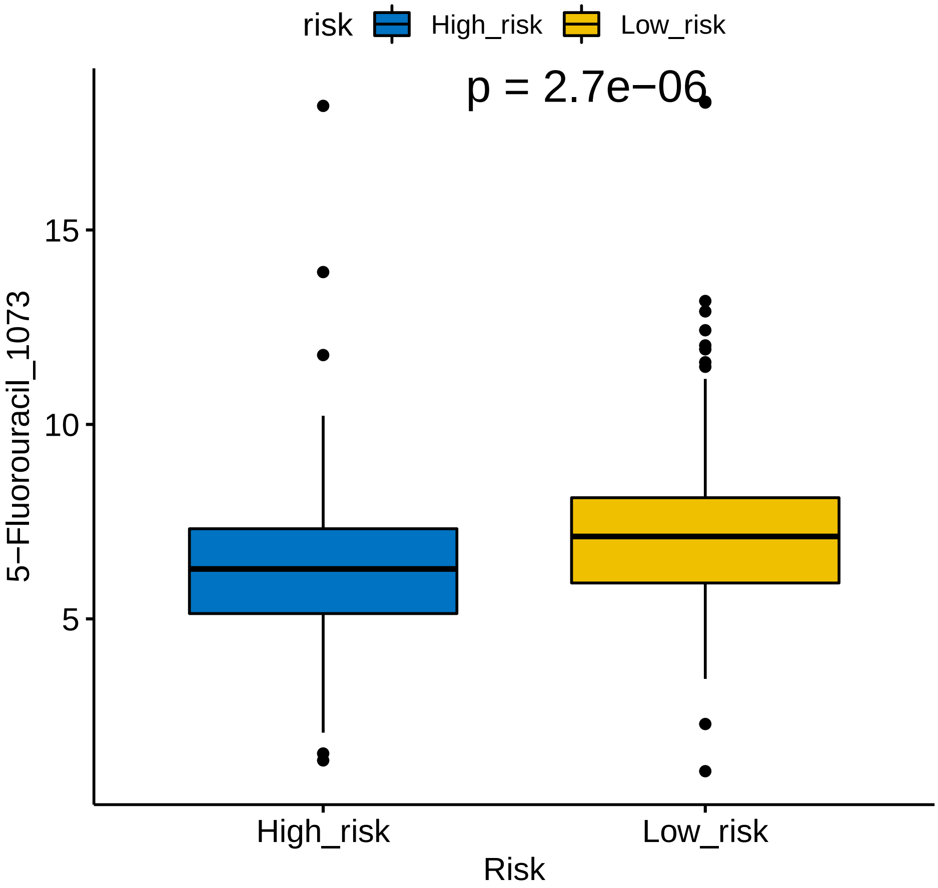

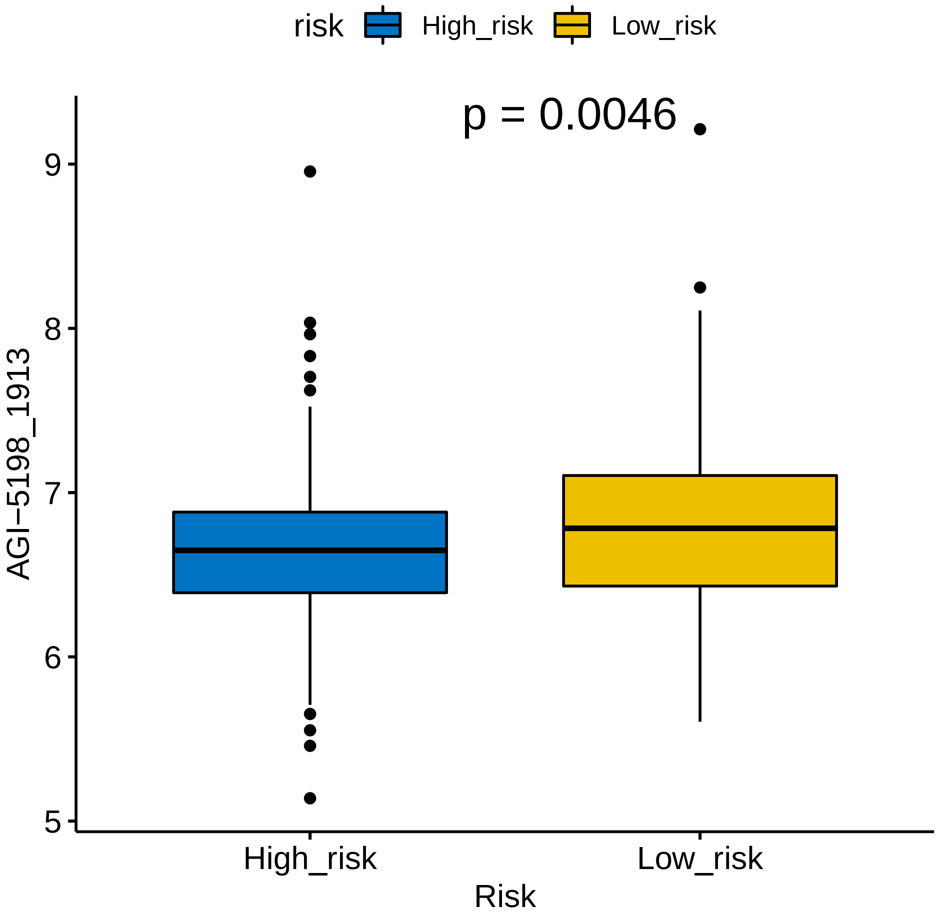
risk
High_risk
Low_risk
p = 0.0046
9
8
7
6
5
AGI−5198_1913
High_risk
Low_risk
Risk

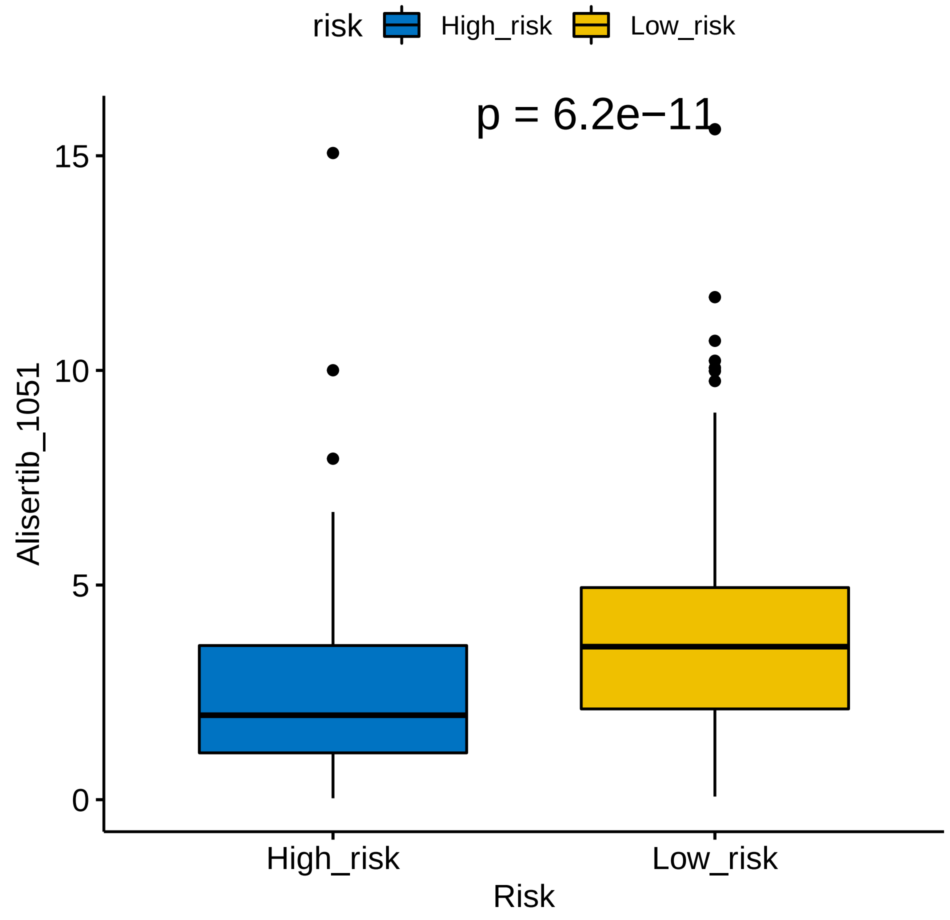

risk
High_risk
Low_risk
p = 6.2e−11
15
10
5
0
Alisertib_1051
High_risk
Low_risk
Risk

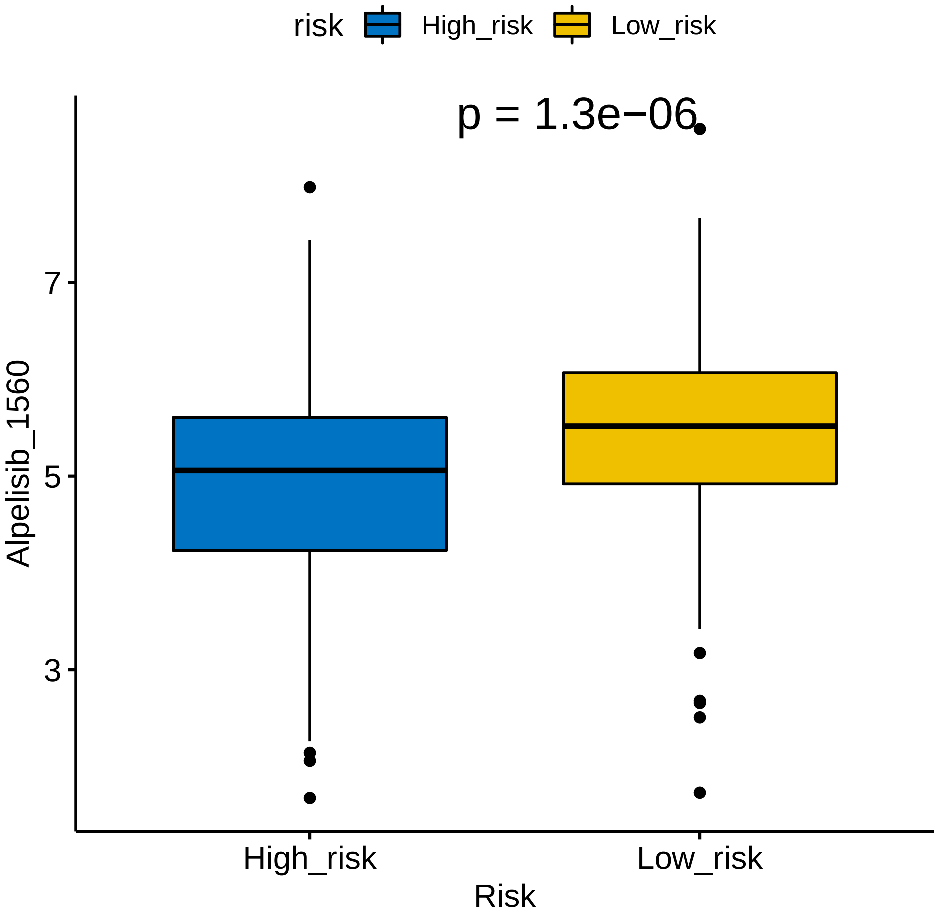

risk
High_risk
Low_risk
p = 1.3e−06
Alpelisib_1560
7
5
3
High_risk
Low_risk
Risk

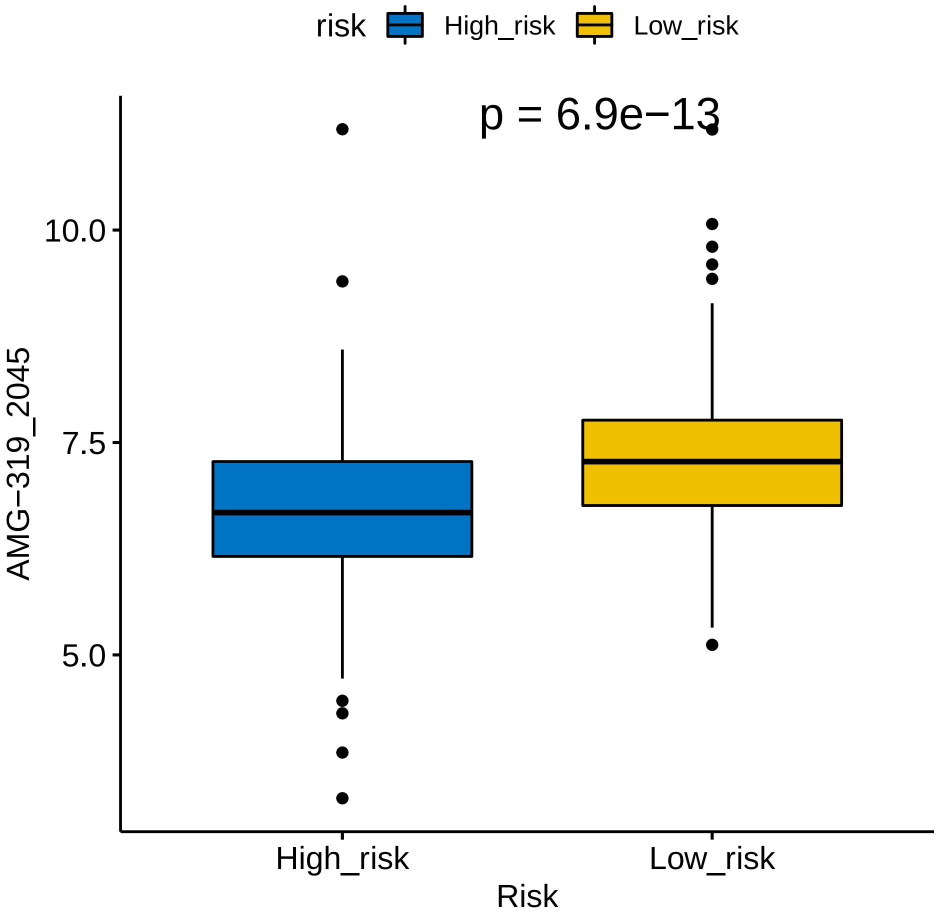

risk
High_risk
Low_risk
p = 6.9e−13
10.0
7.5
5.0
AMG−319_2045
High_risk
Low_risk
Risk

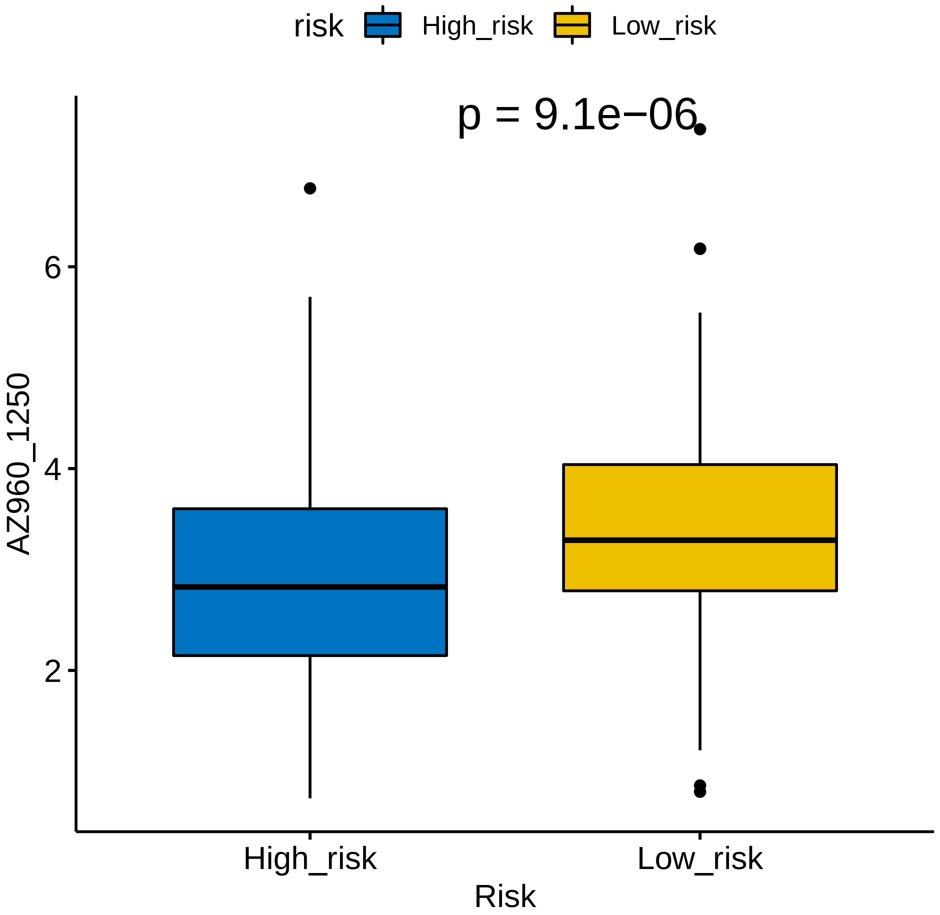

risk
High_risk
Low_risk
p = 9.1e−06
AZ960_1250
6
4
2
High_risk
Low_risk
Risk

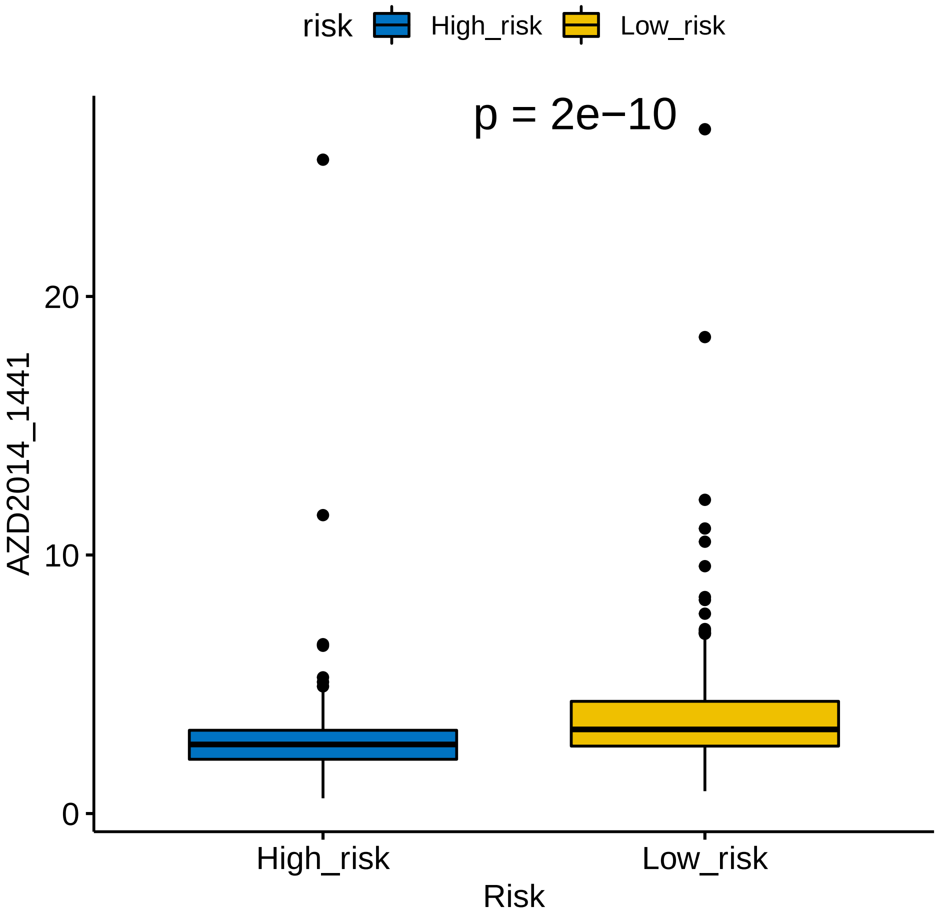
risk
High_risk
Low_risk
p = 2e−10
AZD2014_1441
20
10
0
High_risk
Low_risk
Risk

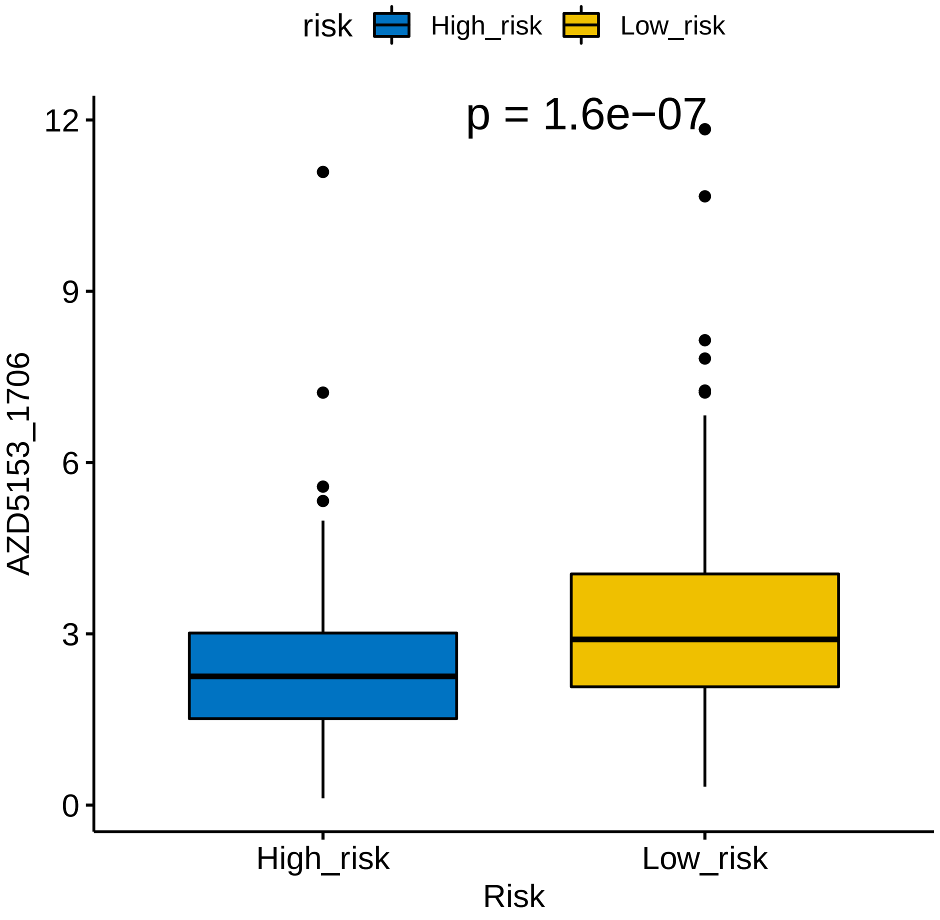
risk
High_risk
Low_risk
p = 1.6e−07
AZD5153_1706
12
9
6
3
0
High_risk
Low_risk
Risk

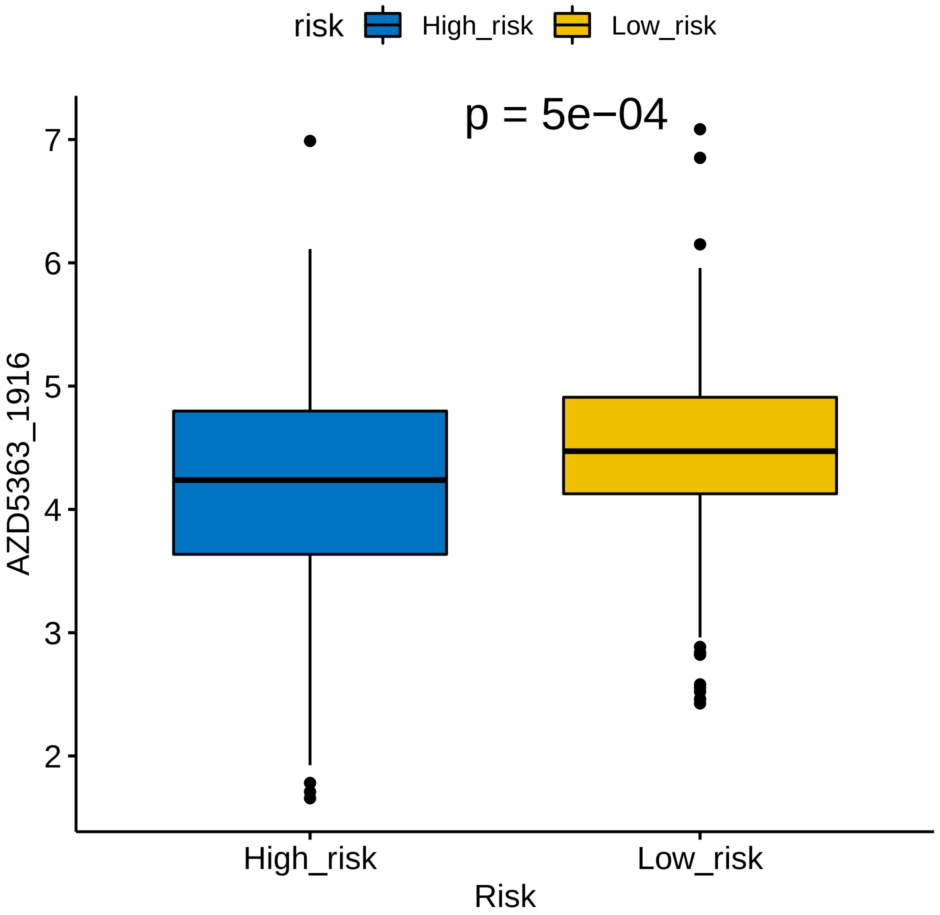

risk
High_risk
Low_risk
p = 5e−04
7
6
5
4
3
2
AZD5363_1916
High_risk
Low_risk
Risk

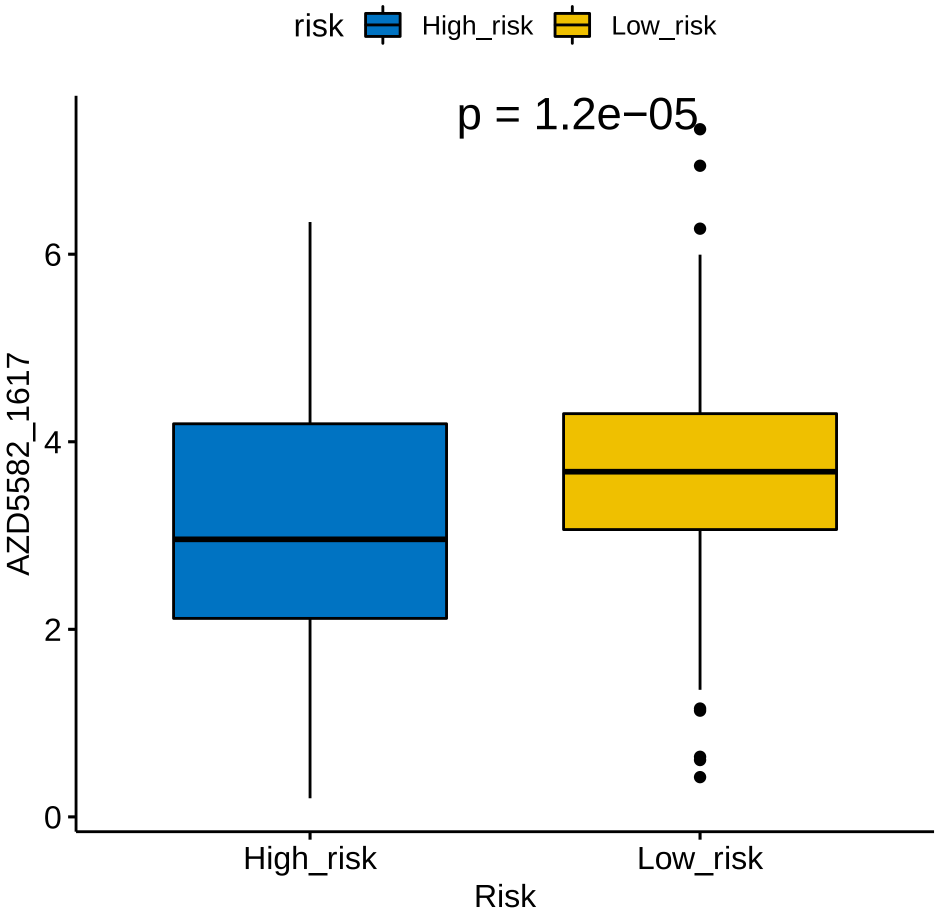

risk
High_risk
Low_risk
p = 1.2e−05
AZD5582_1617
6
4
2
0
High_risk
Low_risk
Risk

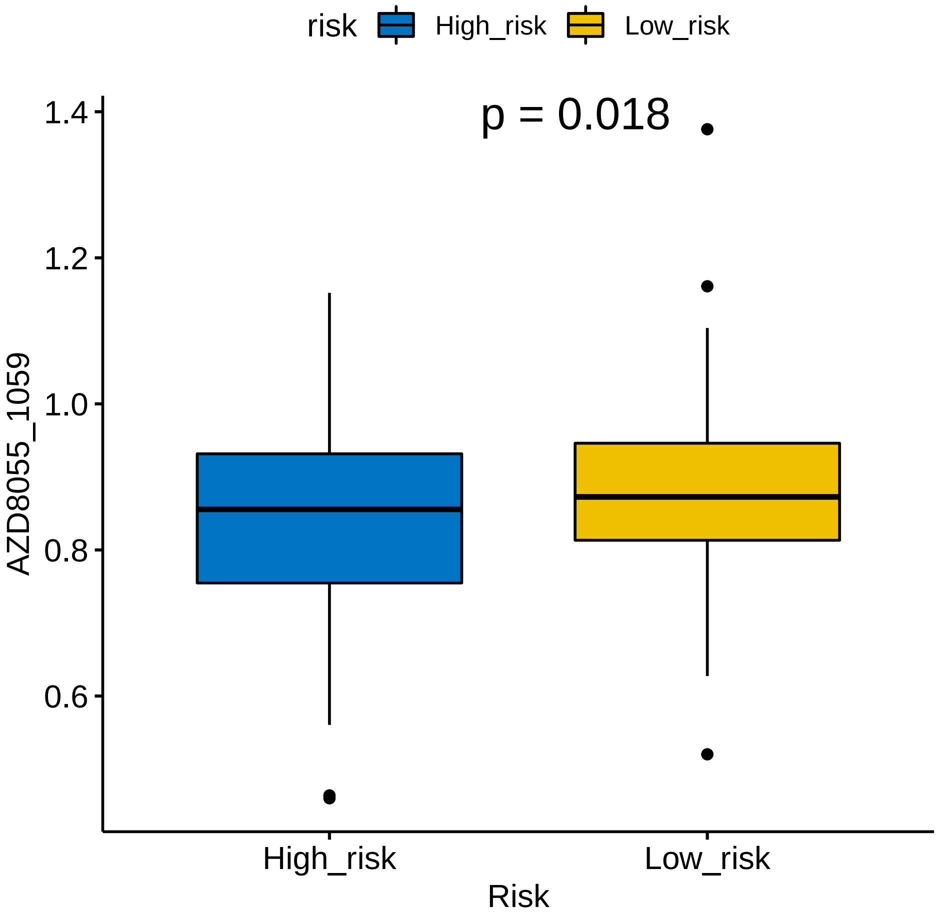

risk
High_risk
Low_risk
p = 0.018
1.4
1.2
1.0
0.8
0.6
AZD8055_1059
High_risk
Low_risk
Risk

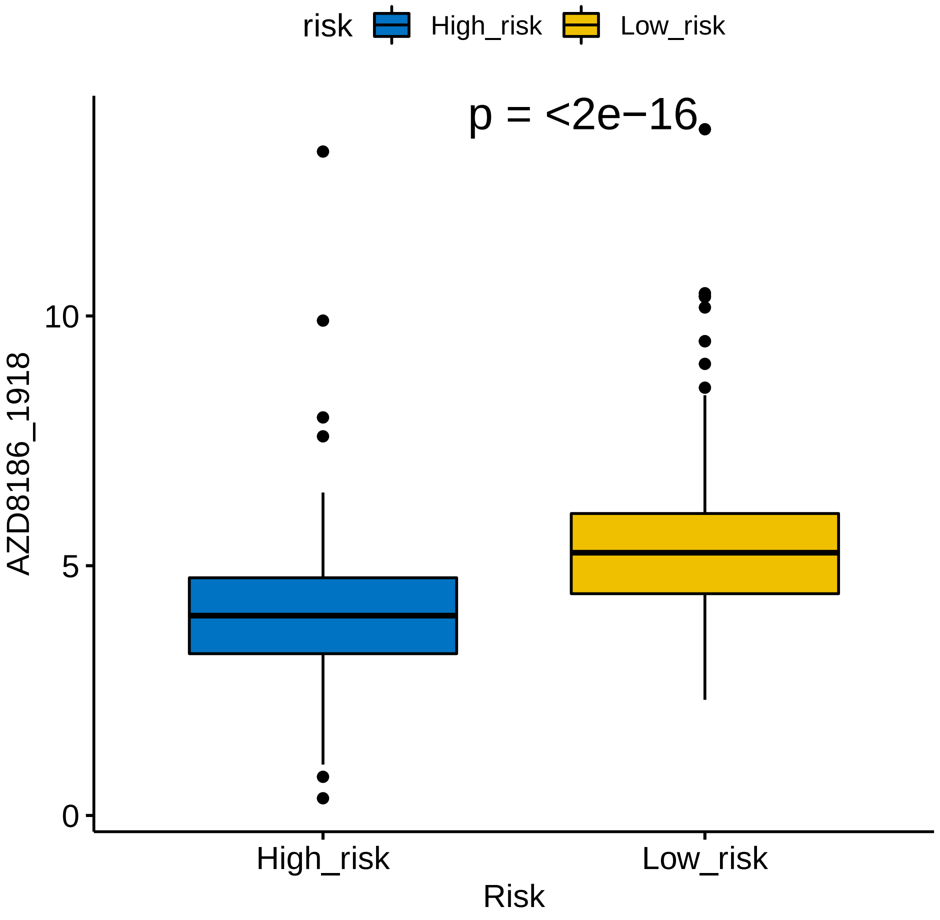
risk
High_risk
Low_risk
p = <2e−16
10
5
0
AZD8186_1918
High_risk
Low_risk
Risk

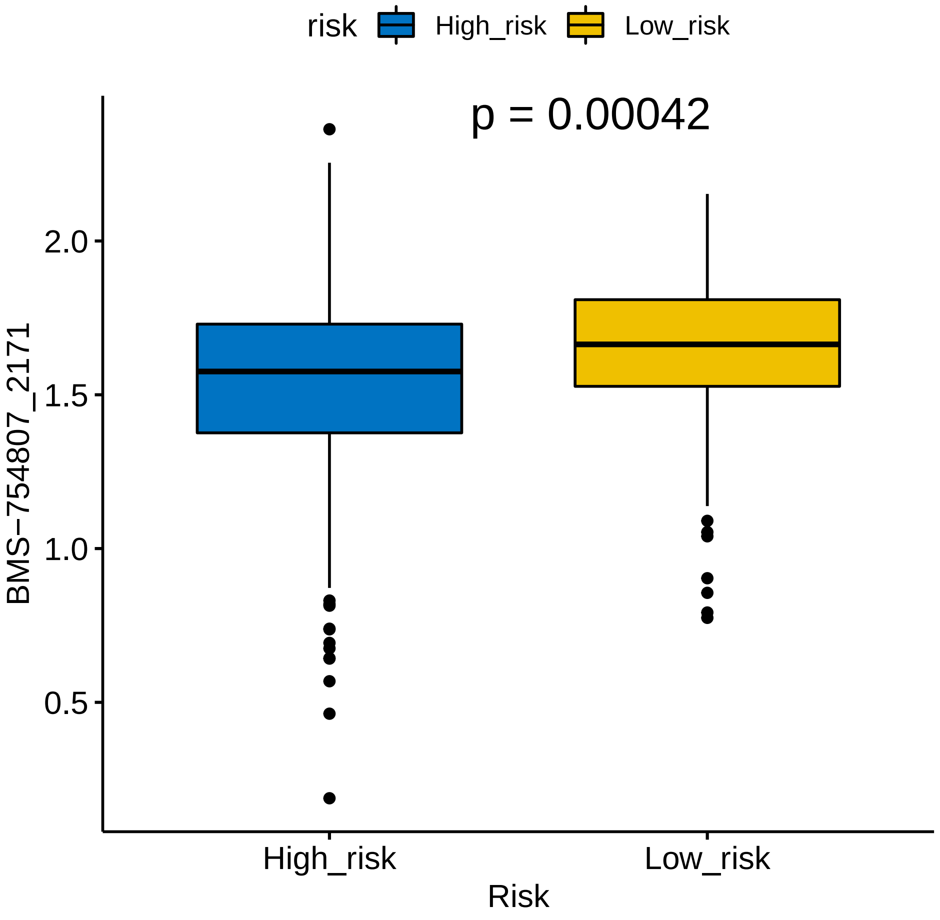
risk
High_risk
Low_risk
p = 0.00042
BMS−754807_2171
2.0
1.5
1.0
0.5
High_risk
Low_risk
Risk

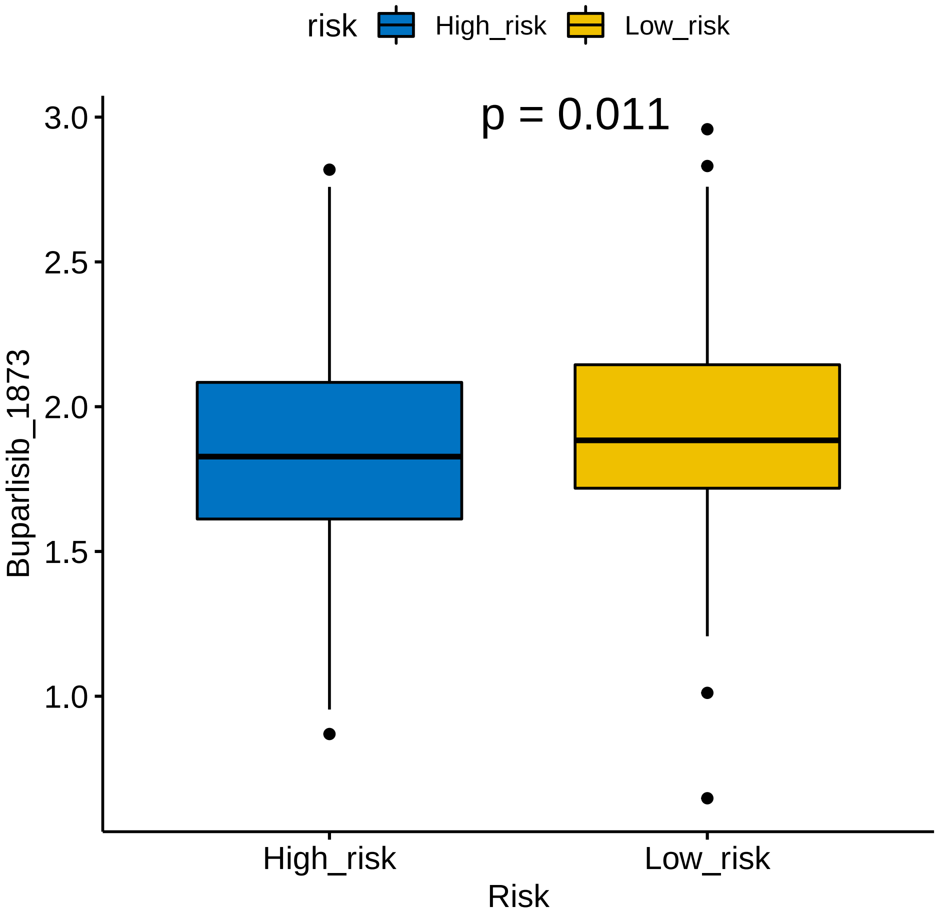

risk
High_risk
Low_risk
p = 0.011
3.0
2.5
2.0
1.5
1.0
Buparlisib_1873
High_risk
Low_risk
Risk

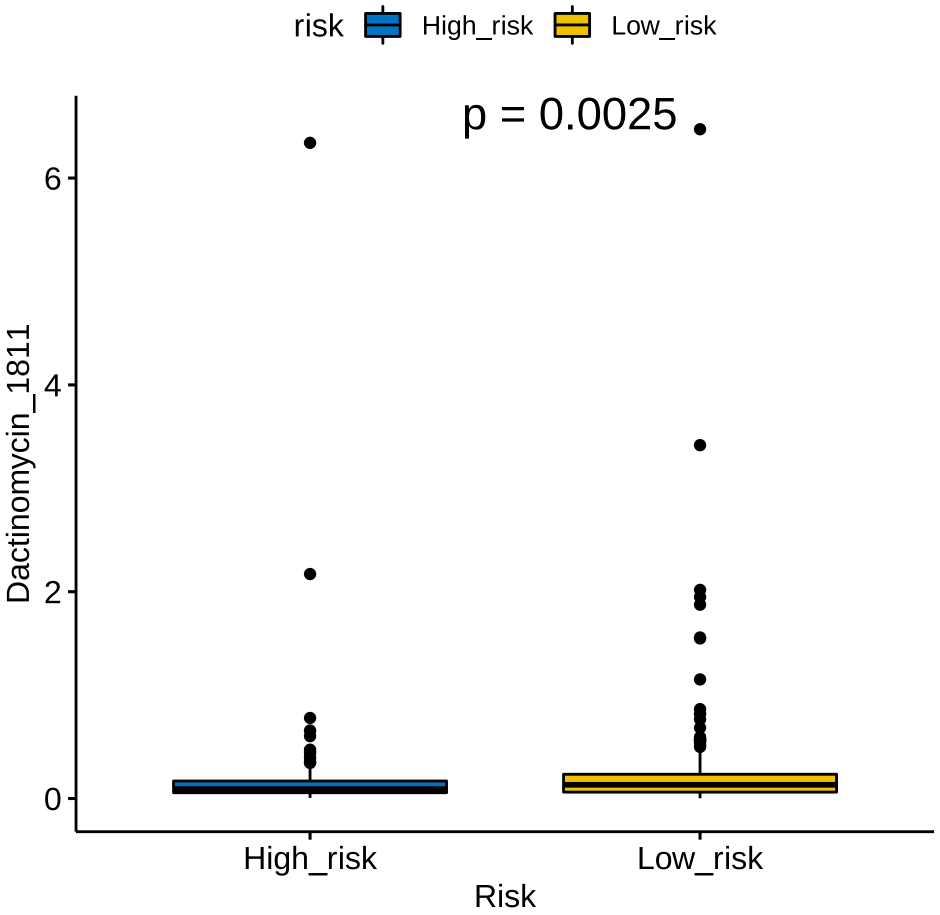
risk
High_risk
Low_risk
p = 0.0025
Dactinomycin_1811
6
4
2
0
High_risk
Low_risk
Risk

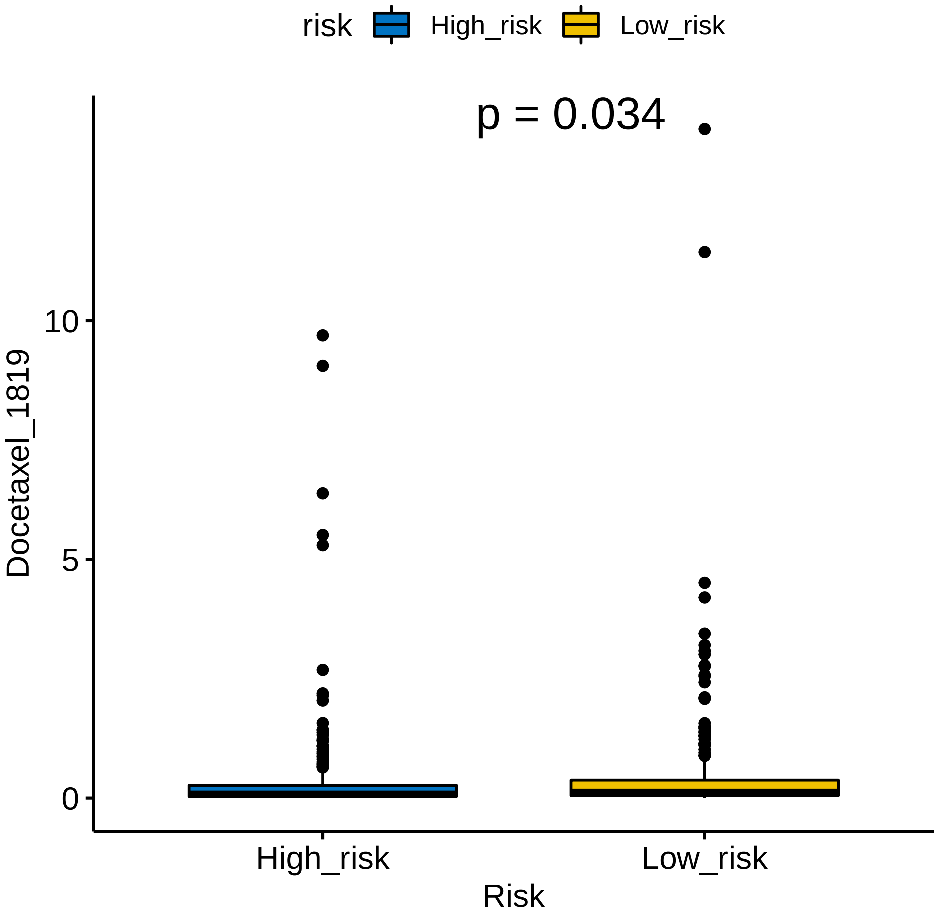
risk
High_risk
Low_risk
p = 0.034
Docetaxel_1819
10
5
0
High_risk
Low_risk
Risk

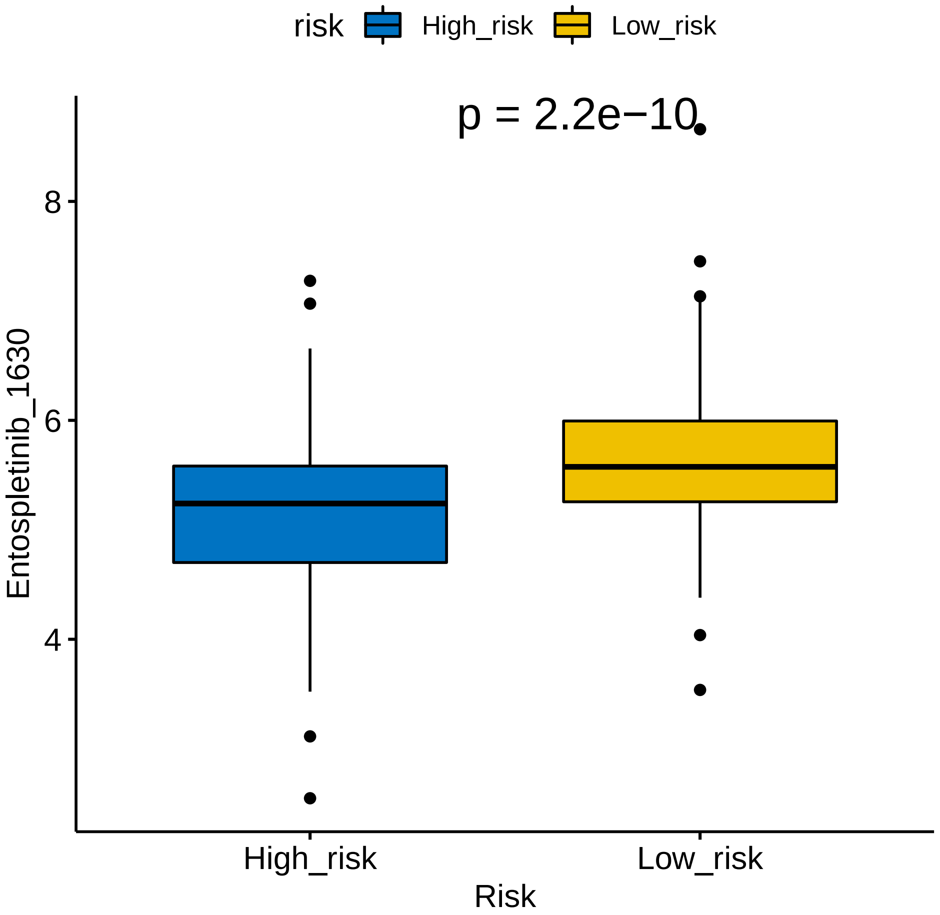

risk
High_risk
Low_risk
p = 2.2e−10
8
6
4
Entospletinib_1630
High_risk
Low_risk
Risk

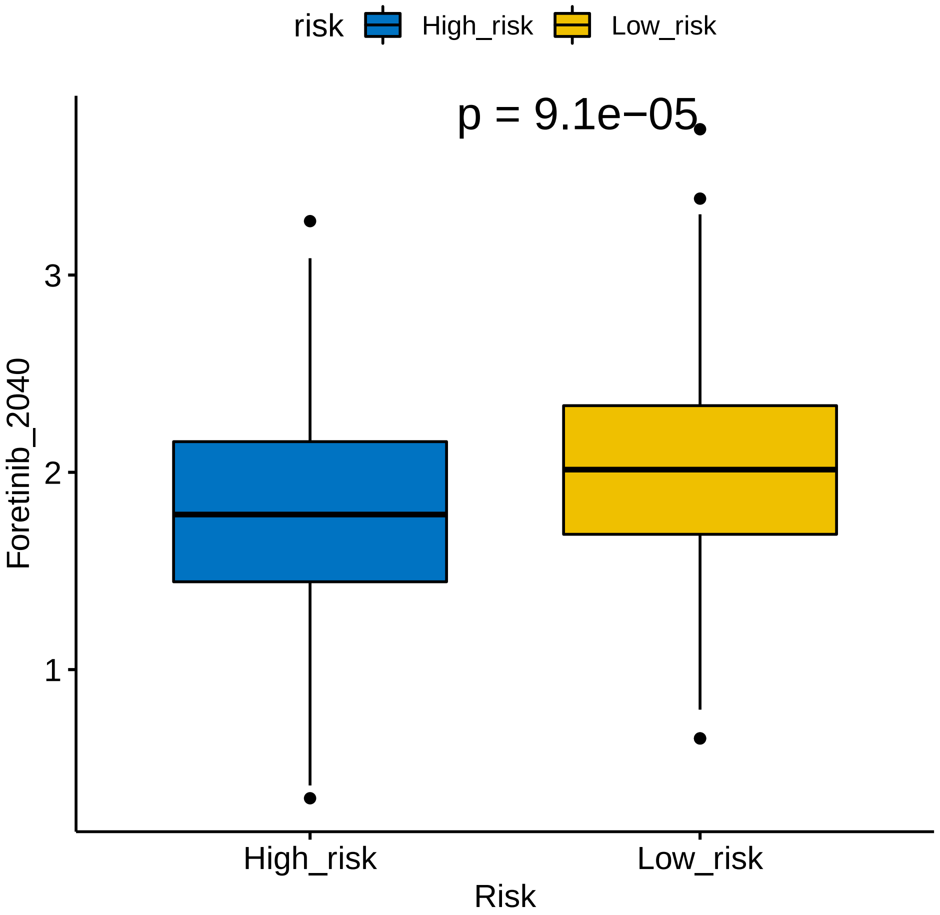

risk
High_risk
Low_risk
p = 9.1e−05
Foretinib_2040
3
2
1
High_risk
Low_risk
Risk

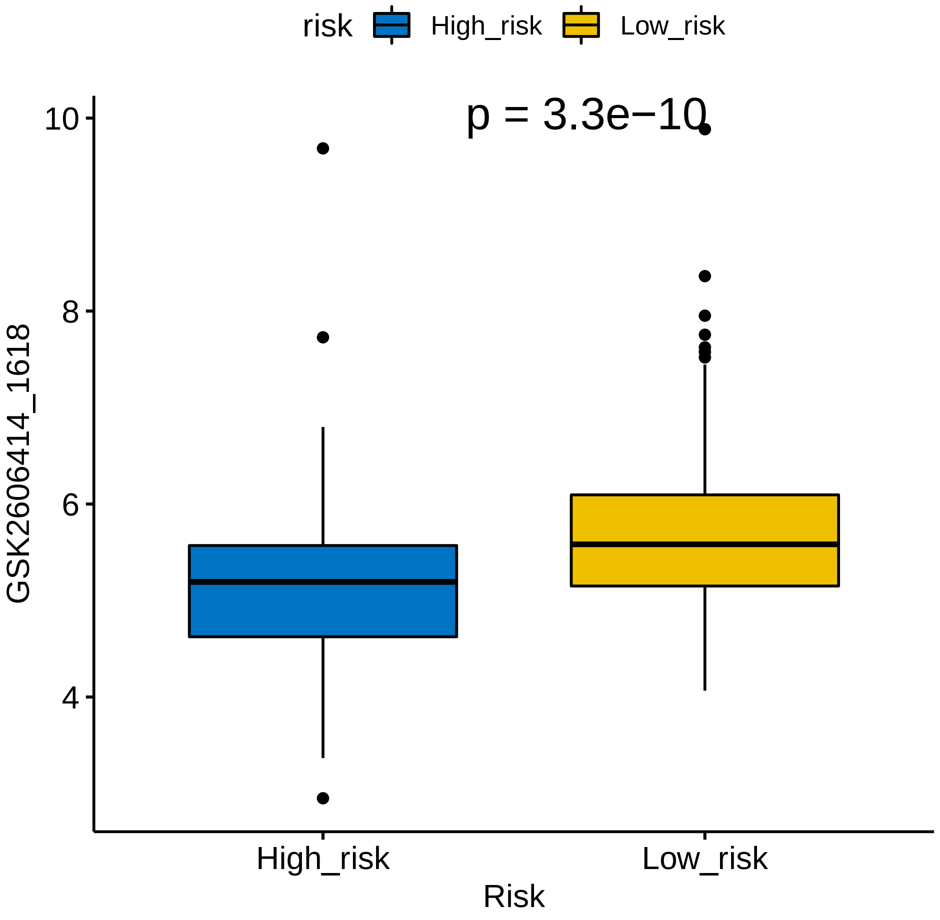
risk
High_risk
Low_risk
p = 3.3e−10
10
8
6
4
GSK2606414_1618
High_risk
Low_risk
Risk

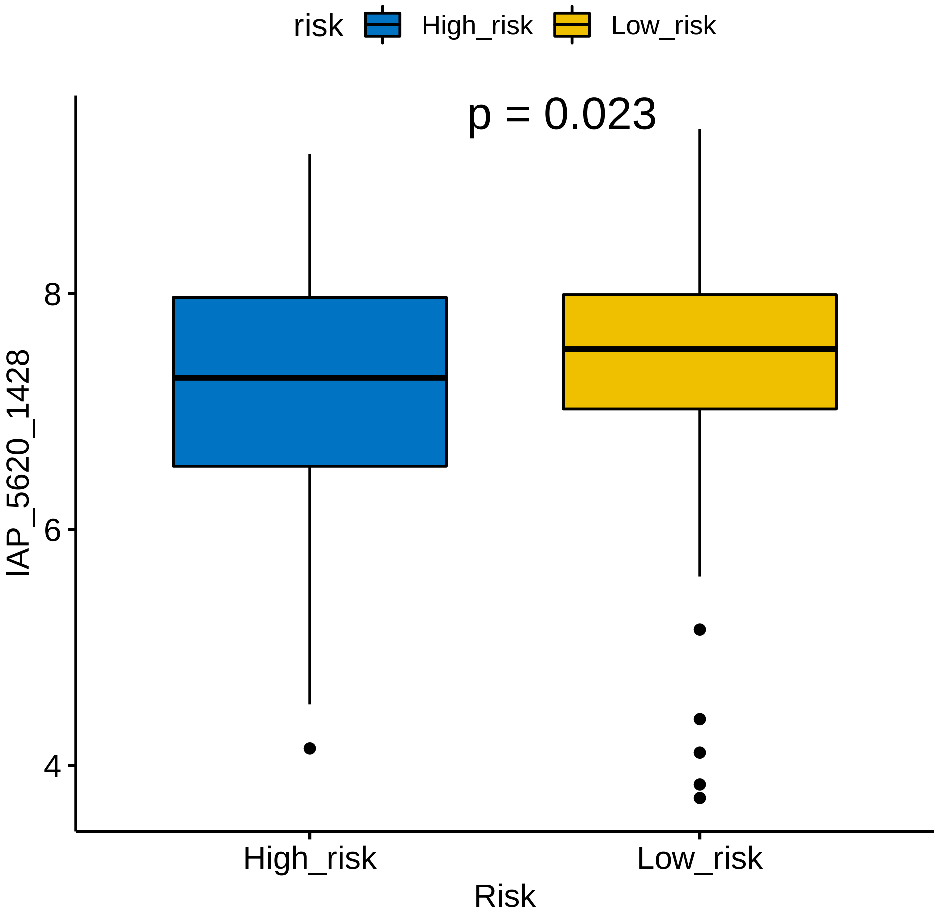

risk
High_risk
Low_risk
p = 0.023
8
6
4
IAP_5620_1428
High_risk
Low_risk
Risk

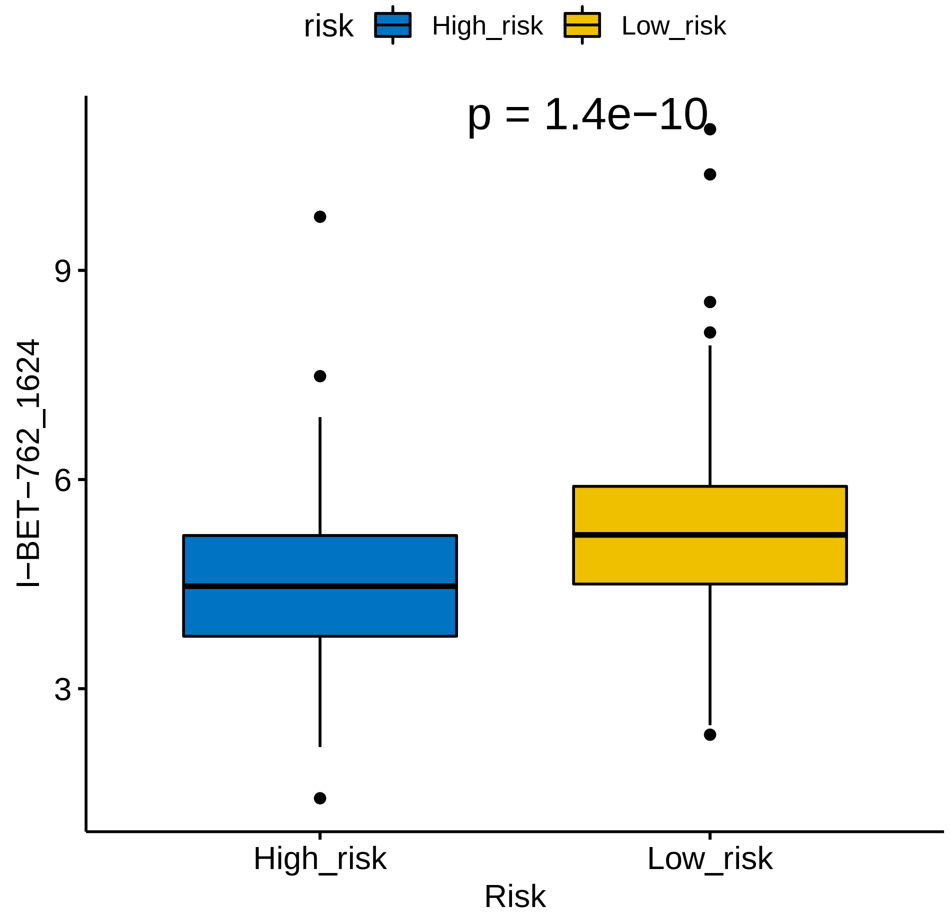

risk
High_risk
Low_risk
p = 1.4e−10
I−BET−762_1624
9
6
3
High_risk
Low_risk
Risk

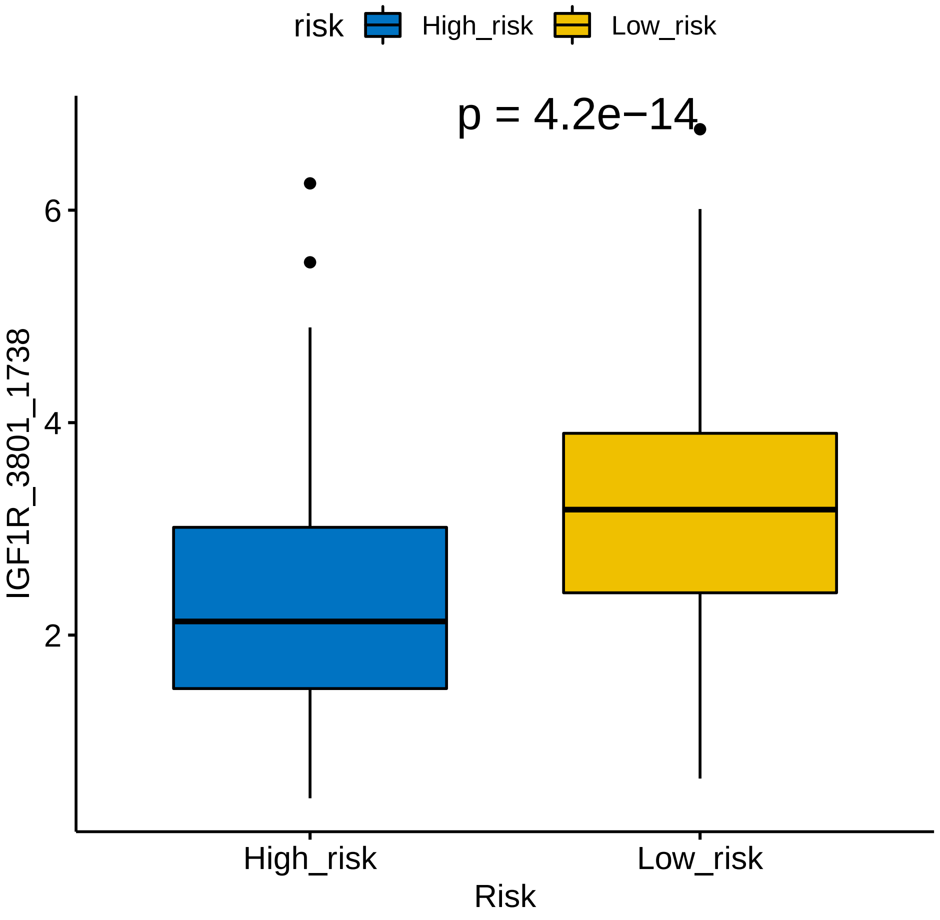

risk
High_risk
Low_risk
p = 4.2e−14
IGF1R_3801_1738
6
4
2
High_risk
Low_risk
Risk

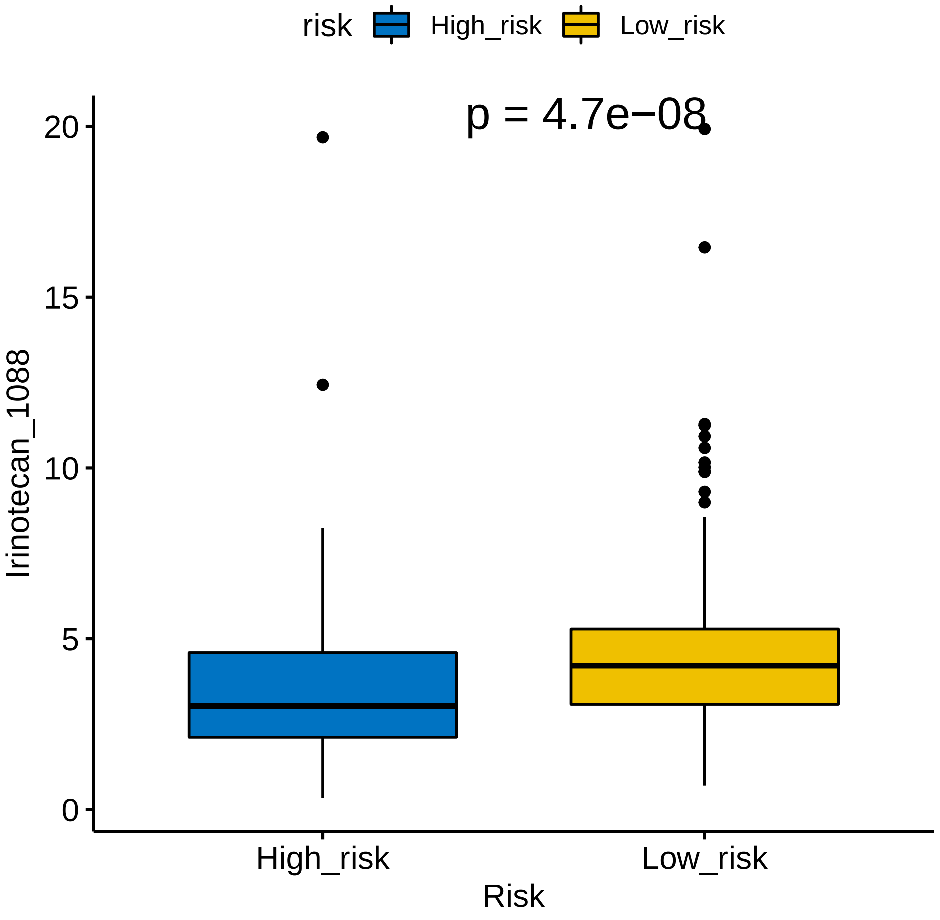

risk
High_risk
Low_risk
p = 4.7e−08
Irinotecan_1088
20
15
10
5
0
High_risk
Low_risk
Risk

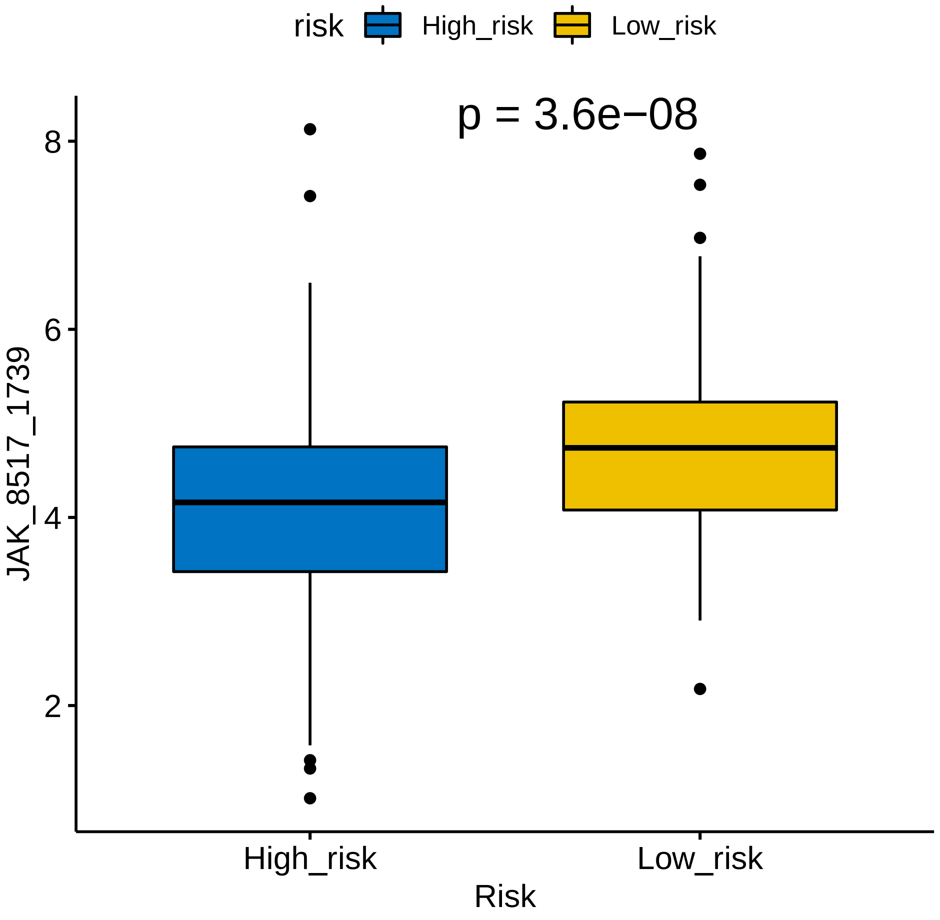

risk
High_risk
Low_risk
p = 3.6e−08
8
6
4
2
JAK_8517_1739
High_risk
Low_risk
Risk

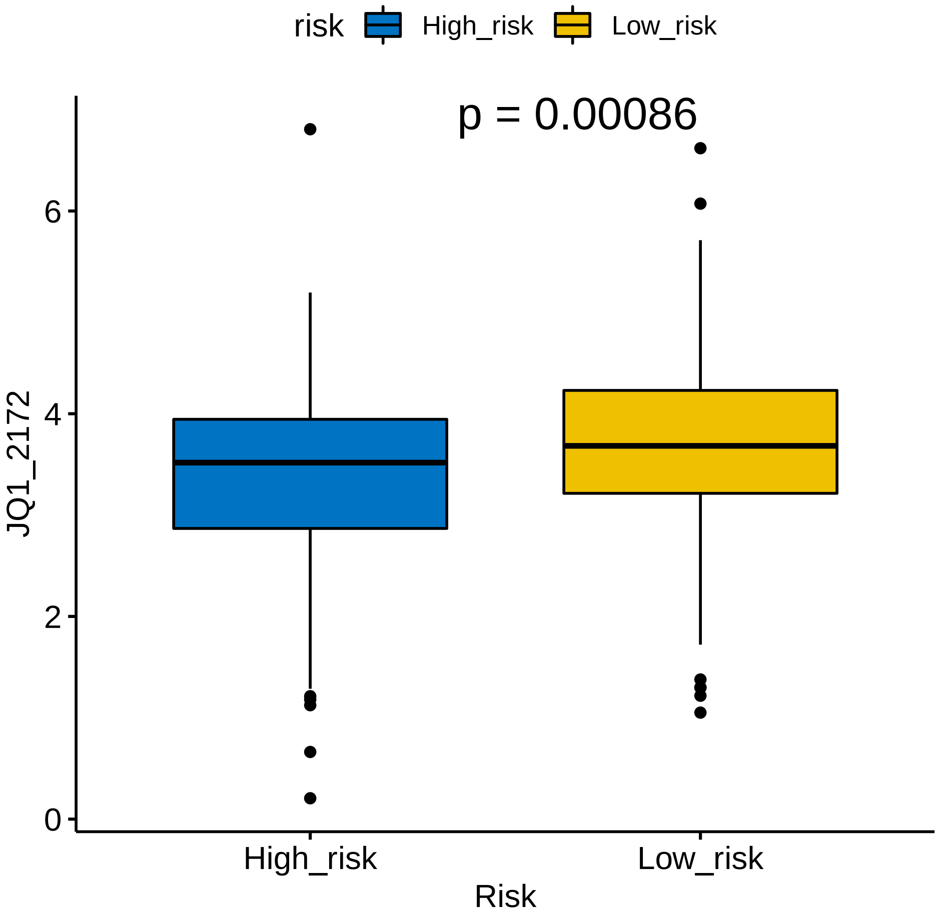
risk
High_risk
Low_risk
p = 0.00086
6
4
2
0
JQ1_2172
High_risk
Low_risk
Risk

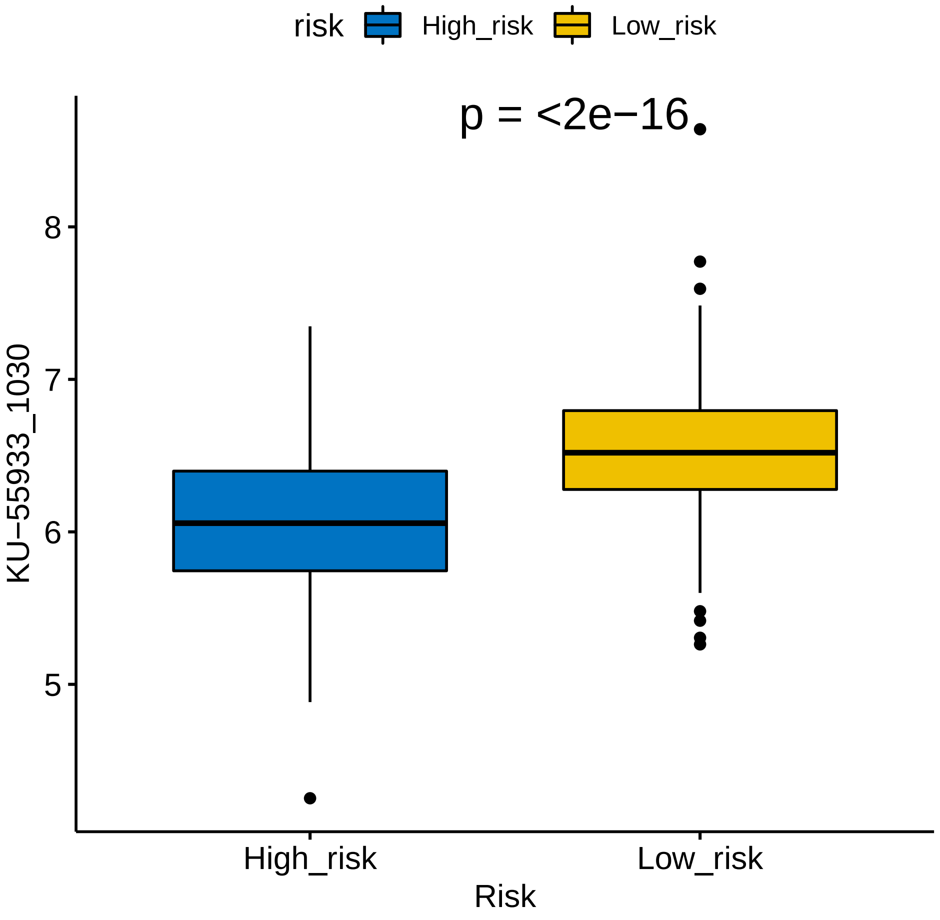
risk
High_risk
Low_risk
p = <2e−16
8
7
6
5
KU−55933_1030
High_risk
Low_risk
Risk

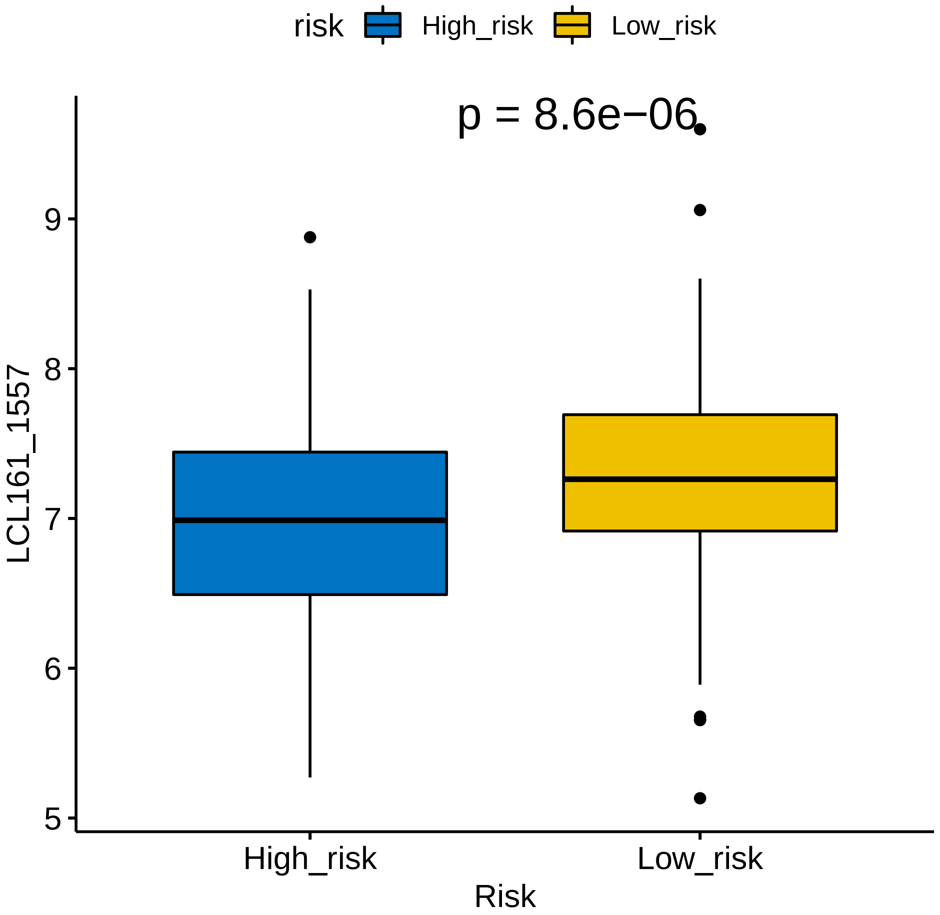

risk
High_risk
Low_risk
p = 8.6e−06
LCL161_1557
9
8
7
6
5
High_risk
Low_risk
Risk

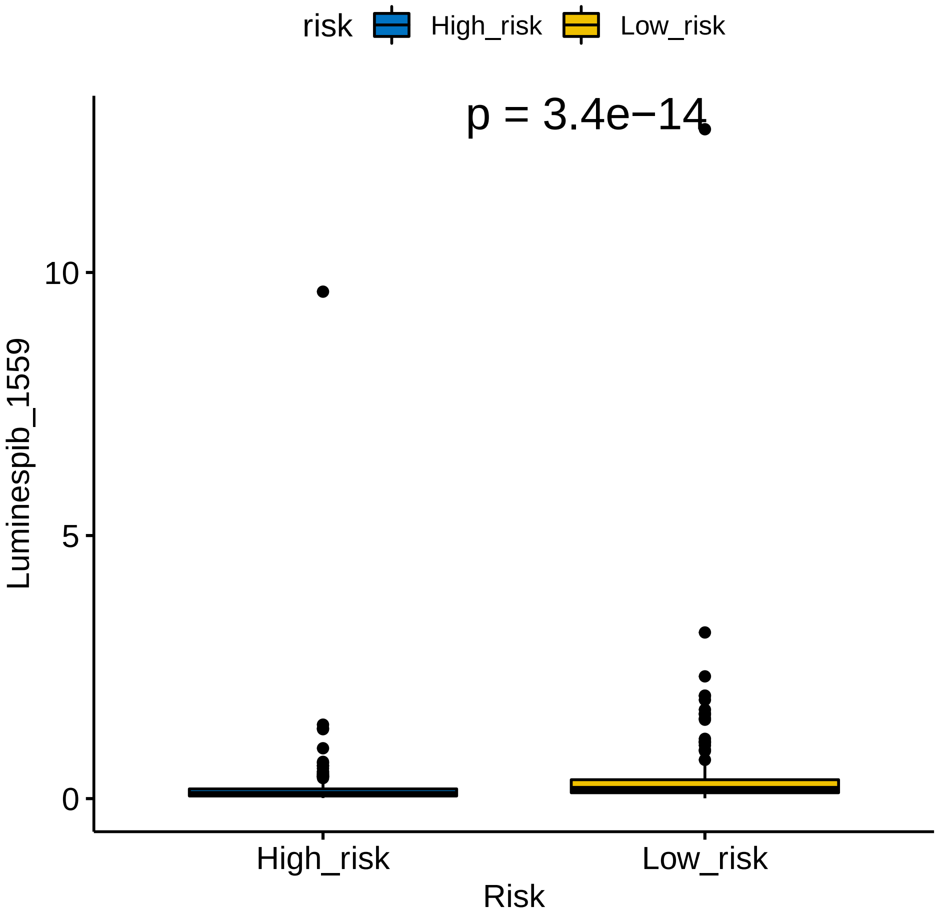

risk
High_risk
Low_risk
p = 3.4e−14
10
5
0
Luminespib_1559
High_risk
Low_risk
Risk

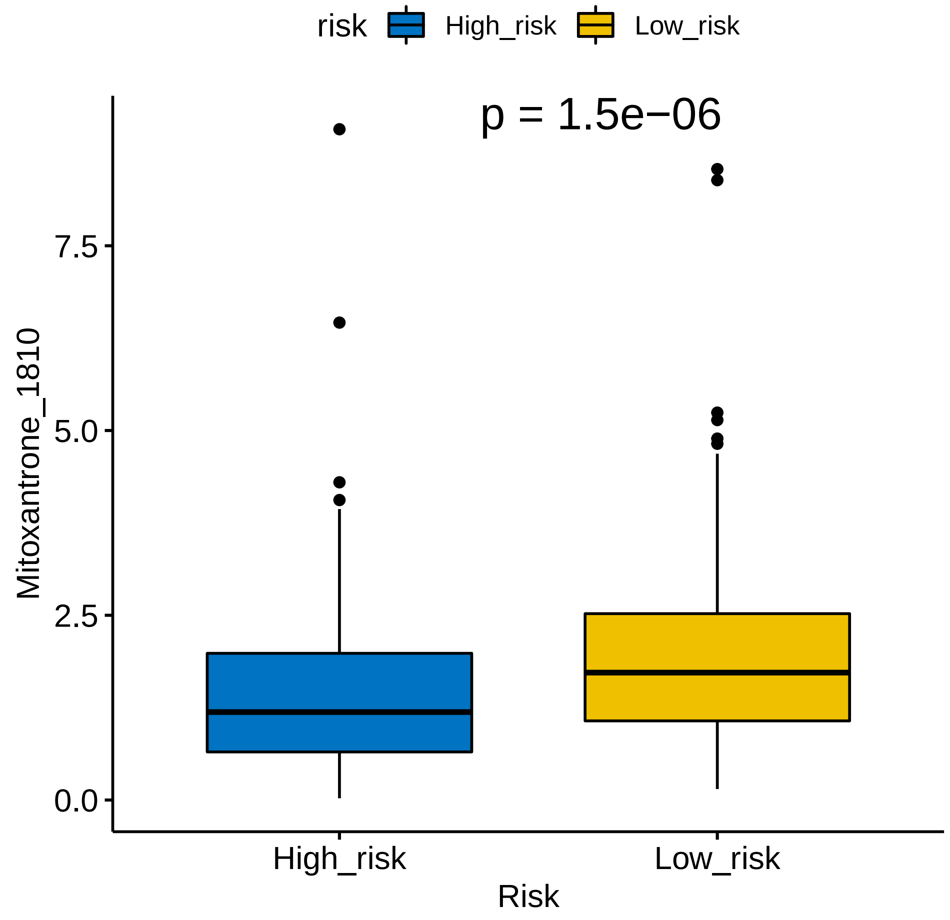
risk
High_risk
Low_risk
p = 1.5e−06
7.5
5.0
2.5
0.0
Mitoxantrone_1810
High_risk
Low_risk
Risk

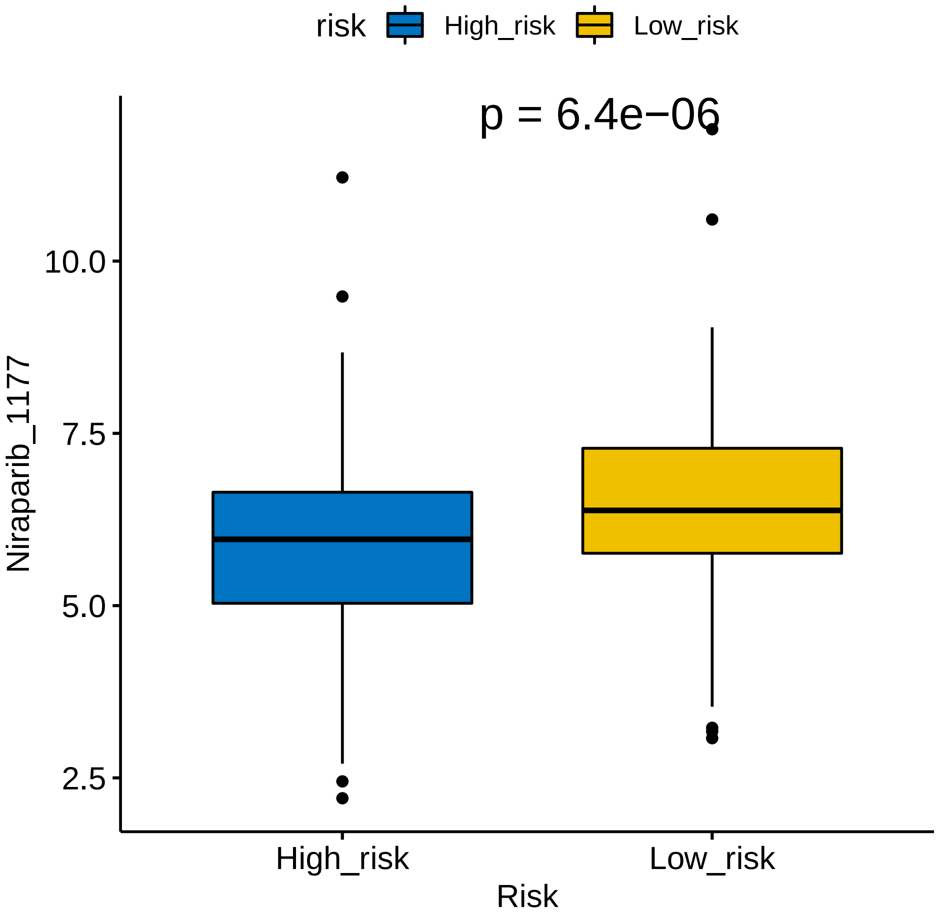
risk
High_risk
Low_risk
p = 6.4e−06
10.0
7.5
5.0
2.5
Niraparib_1177
High_risk
Low_risk
Risk

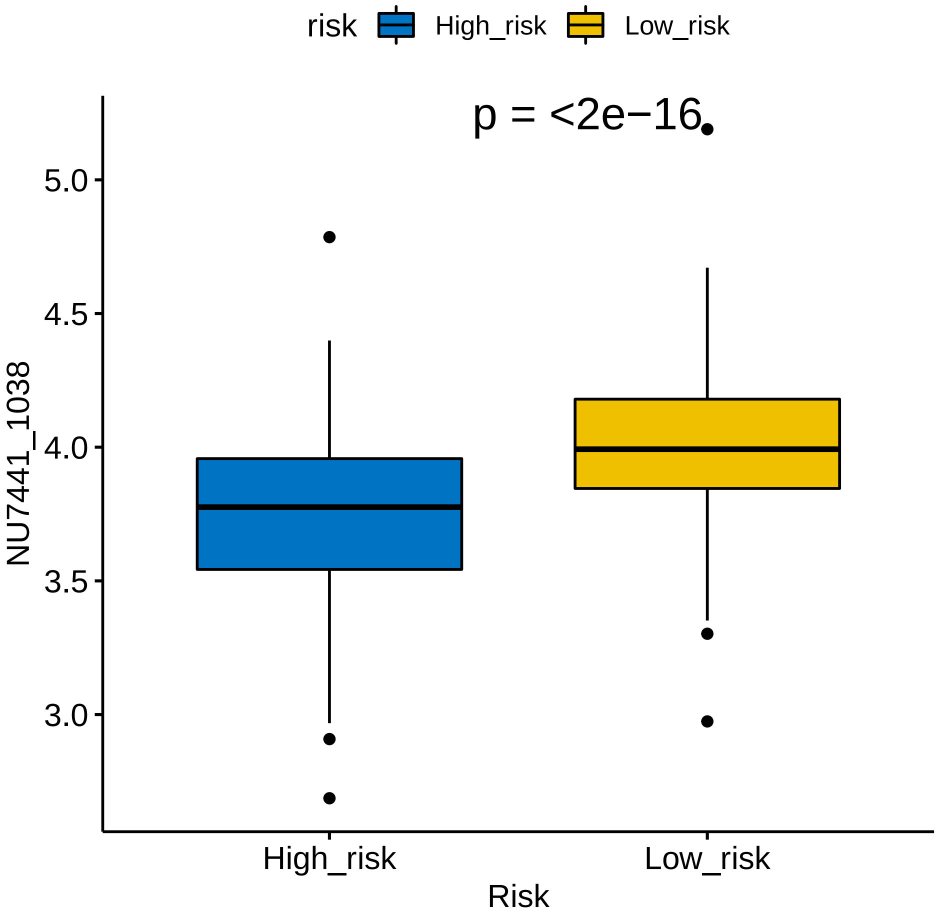

risk
High_risk
Low_risk
p = <2e−16
5.0
4.5
4.0
3.5
3.0
NU7441_1038
High_risk
Low_risk
Risk

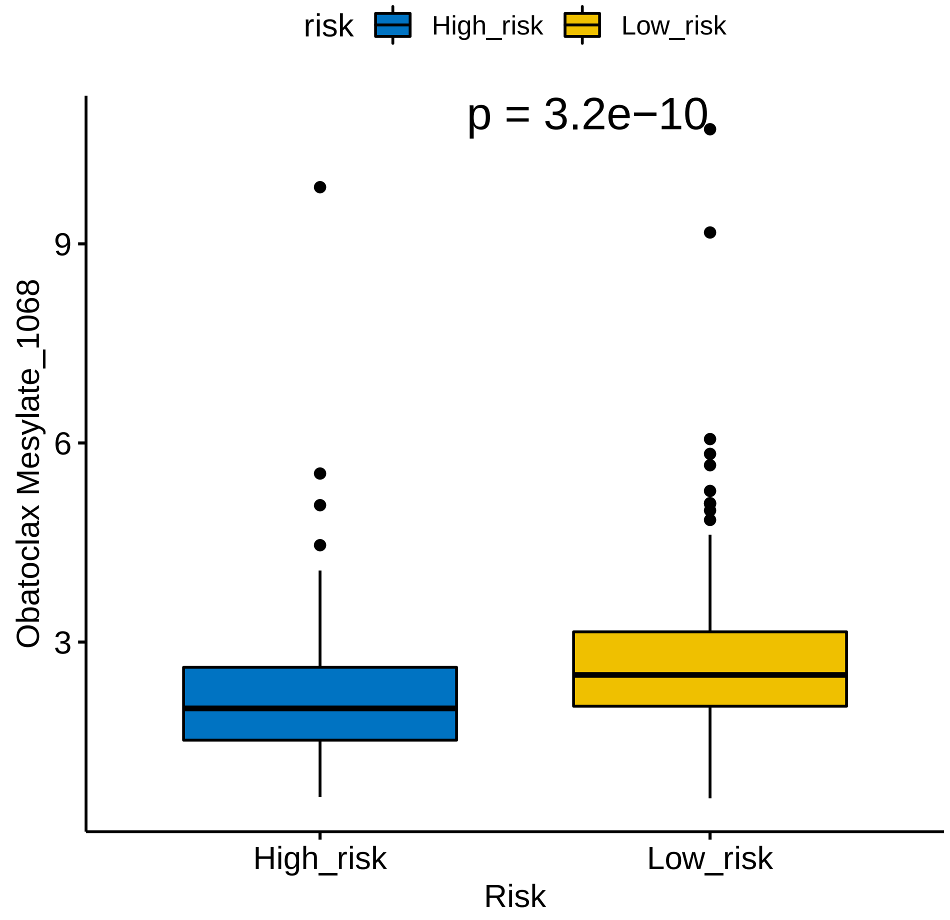

risk
High_risk
Low_risk
p = 3.2e−10
9
6
3
Obatoclax Mesylate_1068
High_risk
Low_risk
Risk

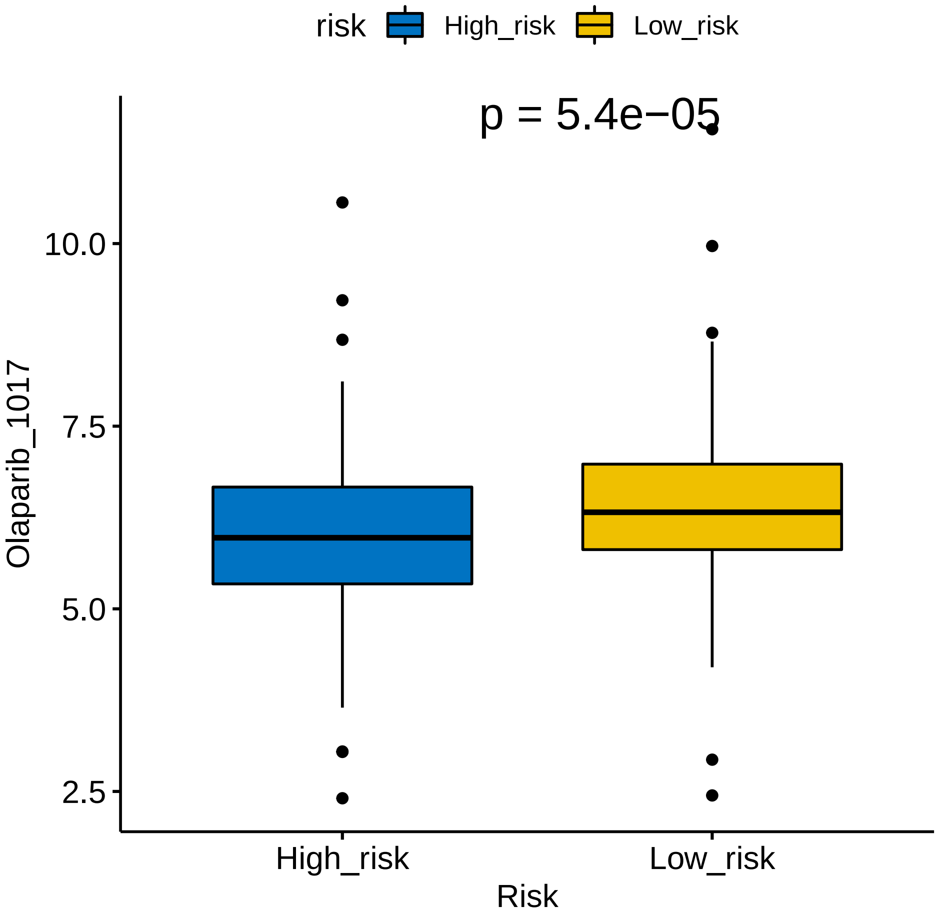

risk
High_risk
Low_risk
p = 5.4e−05
Olaparib_1017
10.0
7.5
5.0
2.5
High_risk
Low_risk
Risk

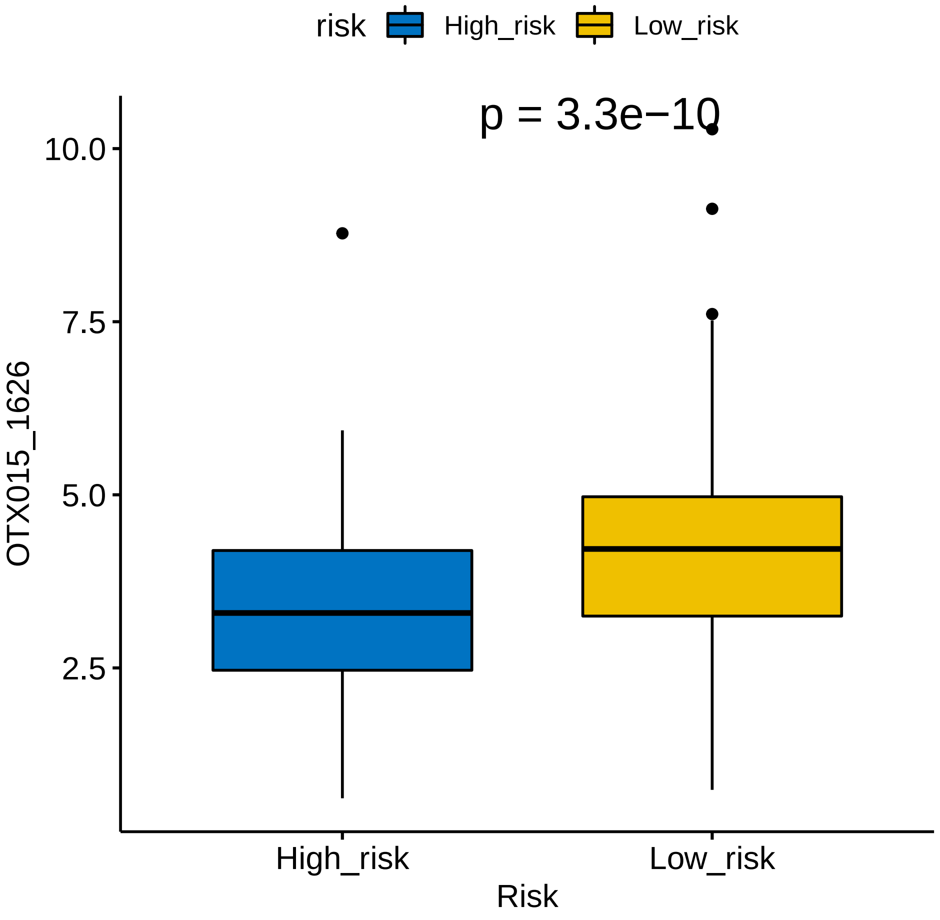
risk
High_risk
Low_risk
p = 3.3e−10
10.0
7.5
5.0
2.5
OTX015_1626
High_risk
Low_risk
Risk

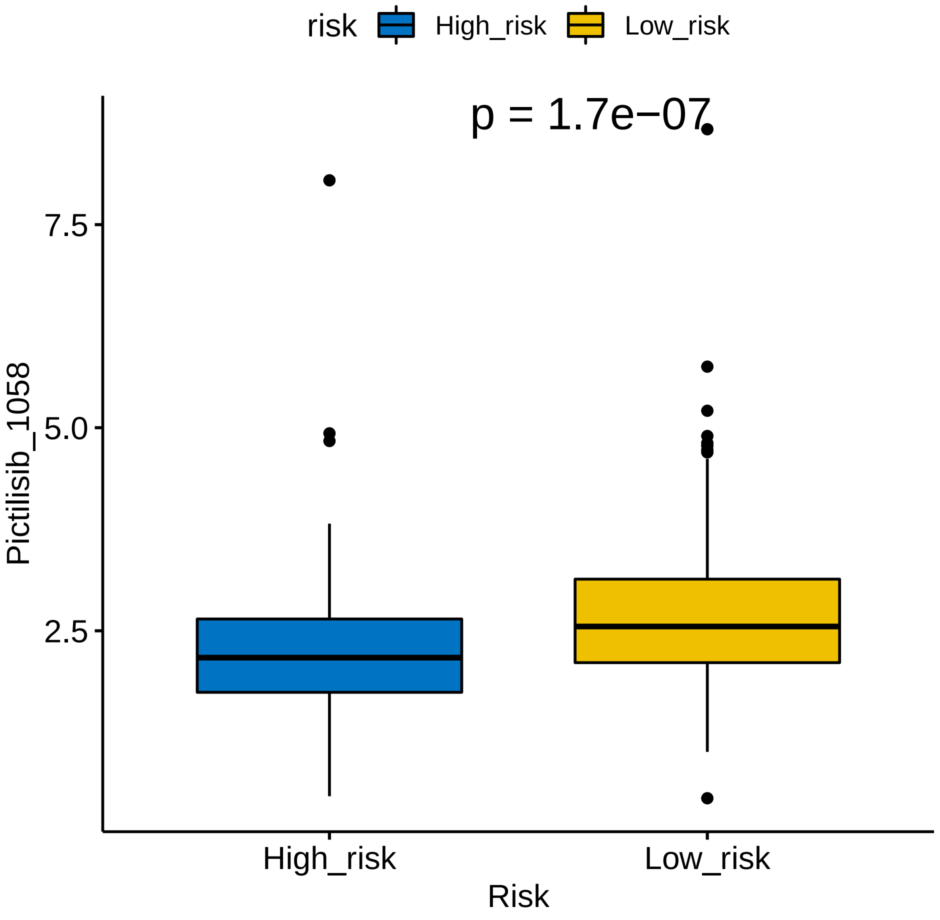
risk
High_risk
Low_risk
p = 1.7e−07
7.5
5.0
2.5
Pictilisib_1058
High_risk
Low_risk
Risk

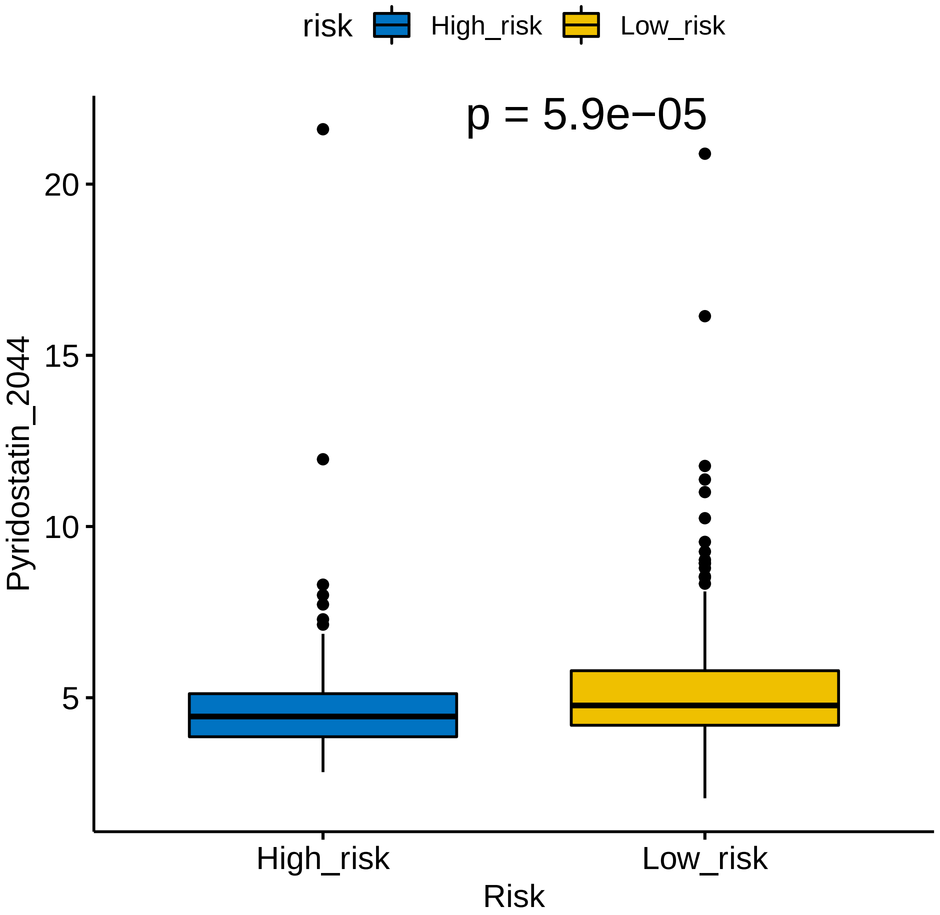

risk
High_risk
Low_risk
p = 5.9e−05
20
15
10
5
Pyridostatin_2044
High_risk
Low_risk
Risk

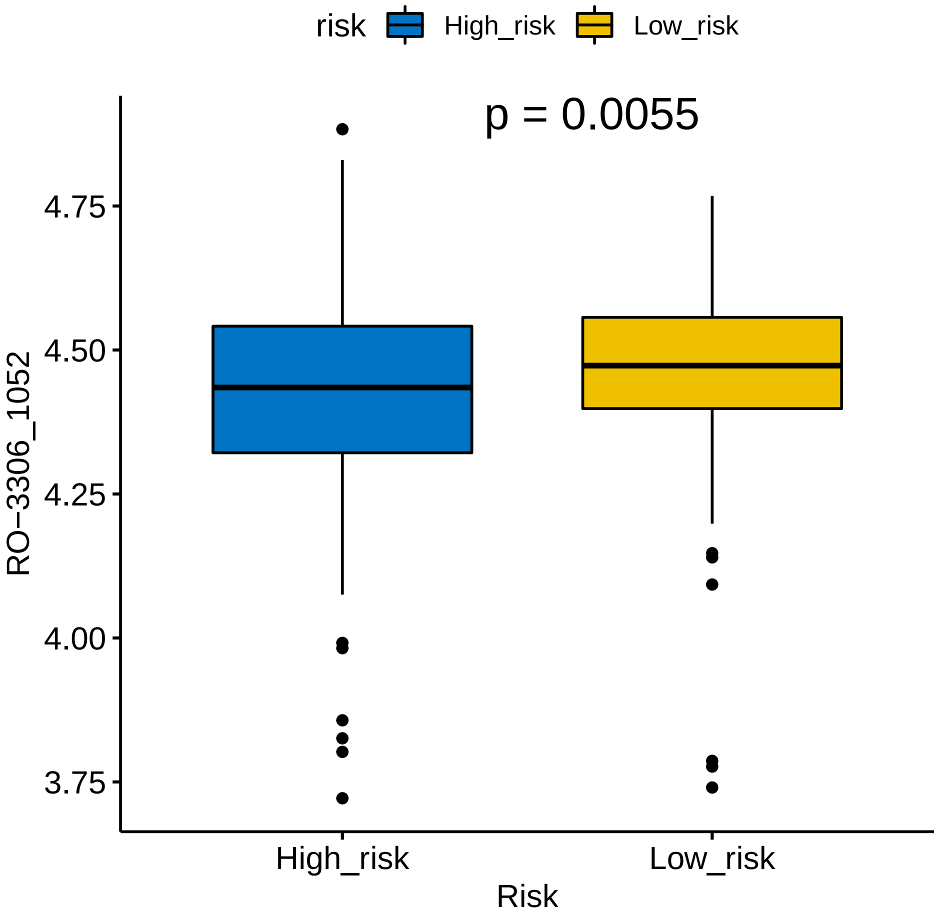

risk
High_risk
Low_risk
p = 0.0055
RO−3306_1052
4.75
4.50
4.25
4.00
3.75
High_risk
Low_risk
Risk

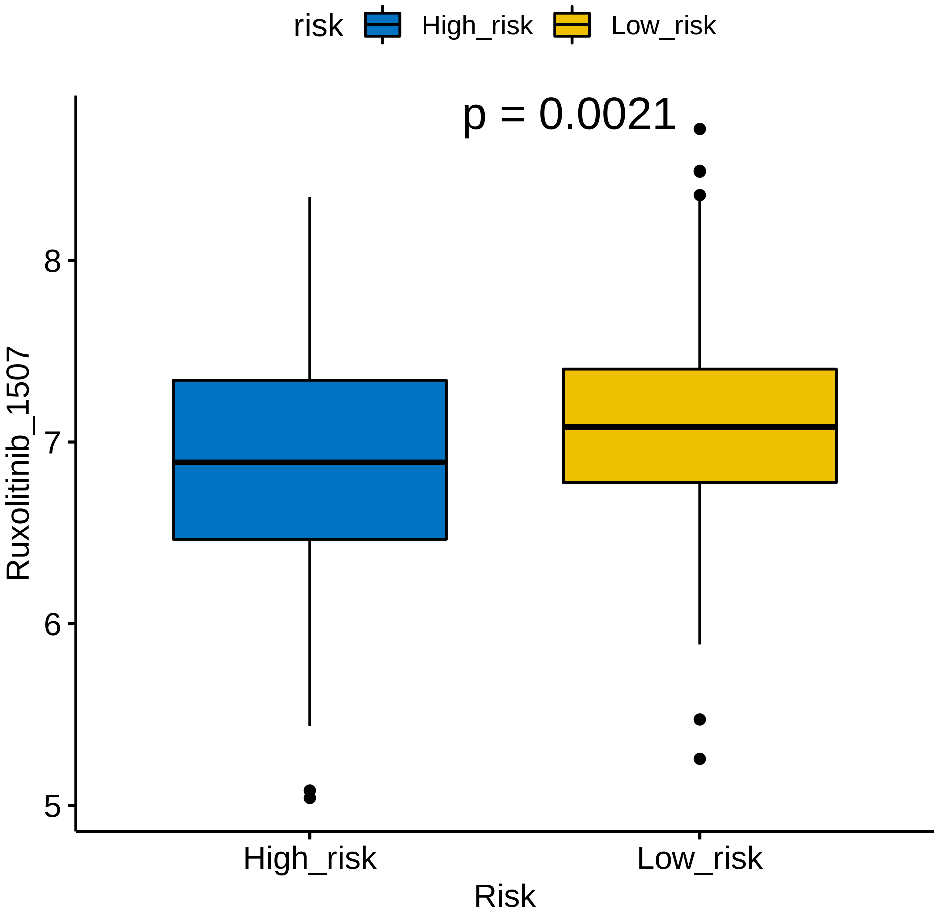

risk
High_risk
Low_risk
p = 0.0021
8
7
6
5
Ruxolitinib_1507
High_risk
Low_risk
Risk

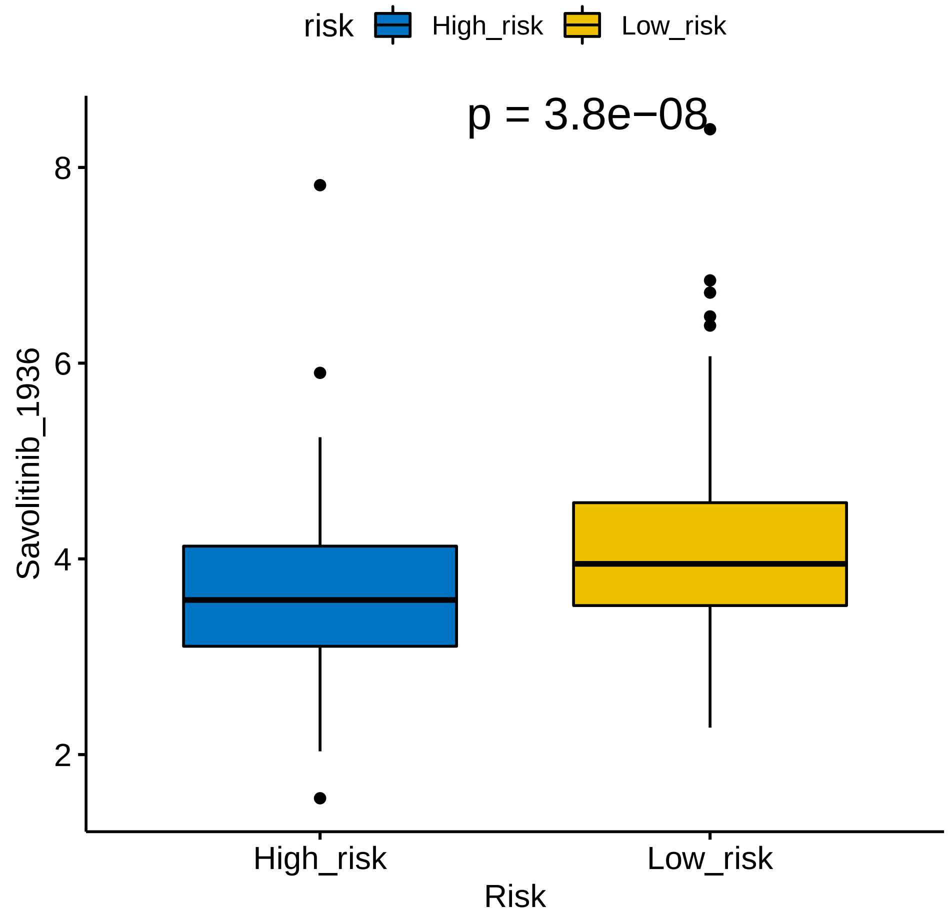
risk
High_risk
Low_risk
p = 3.8e−08
8
6
4
2
Savolitinib_1936
High_risk
Low_risk
Risk

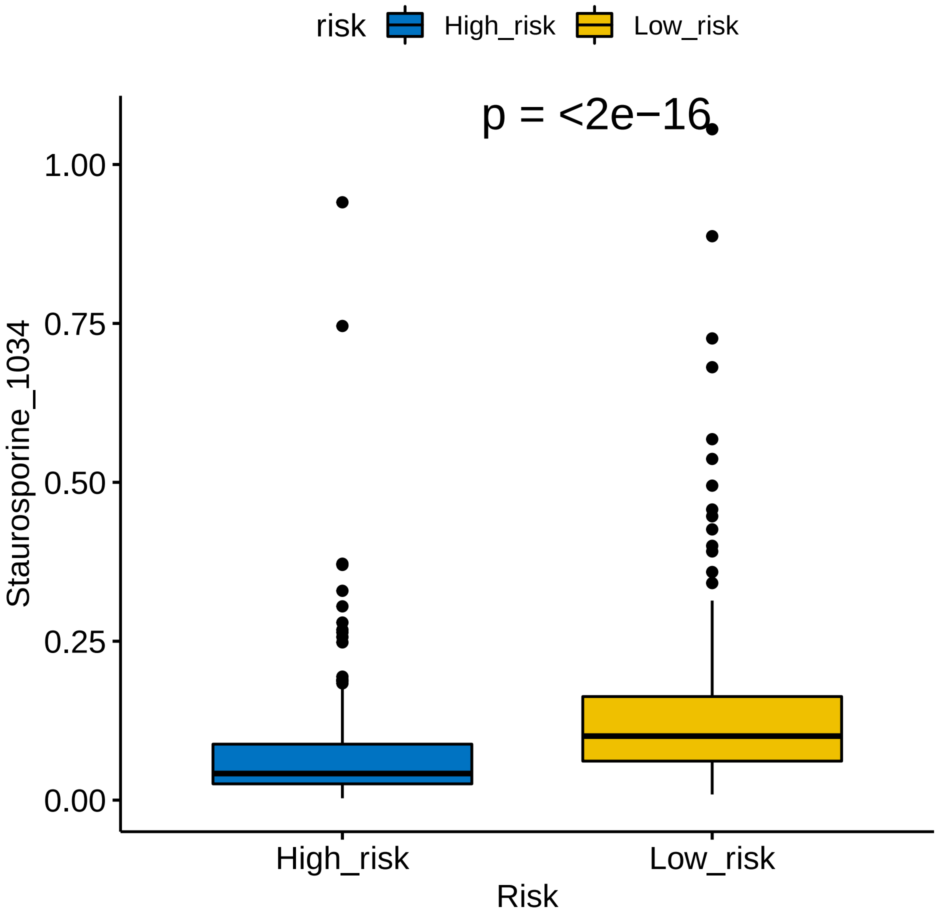
risk
High_risk
Low_risk
p = <2e−16
1.00
0.75
0.50
0.25
0.00
Staurosporine_1034
High_risk
Low_risk
Risk

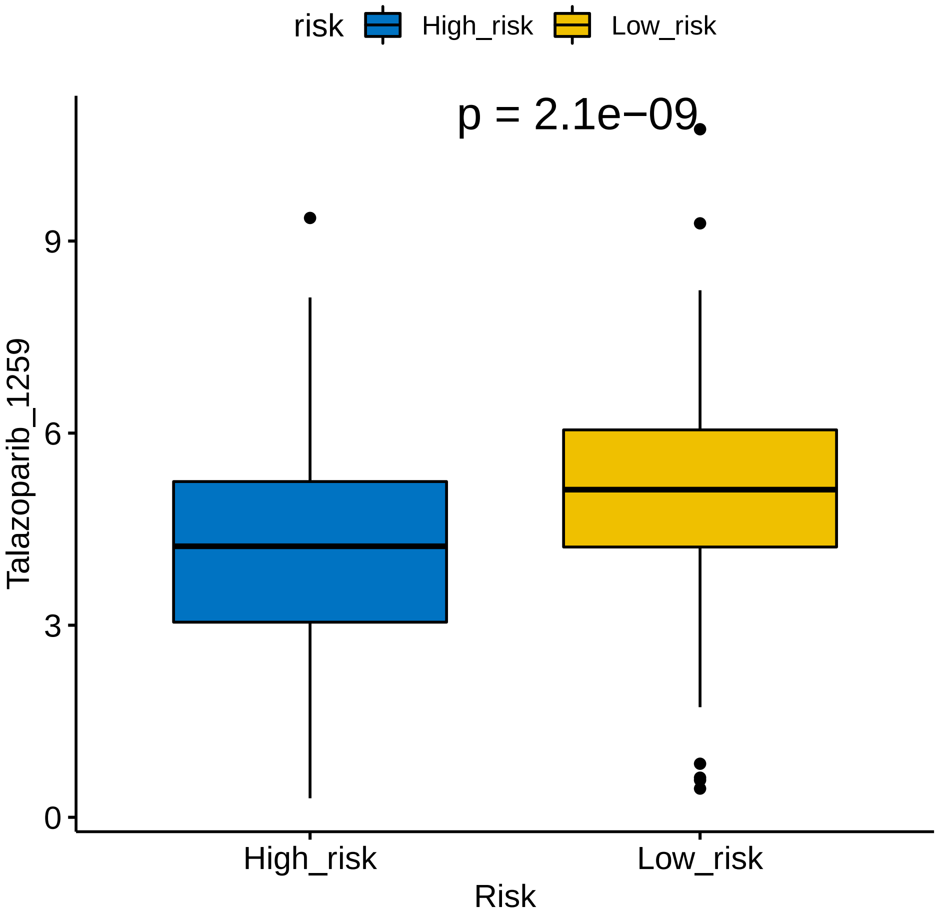
risk
High_risk
Low_risk
p = 2.1e−09
Talazoparib_1259
9
6
3
0
High_risk
Low_risk
Risk

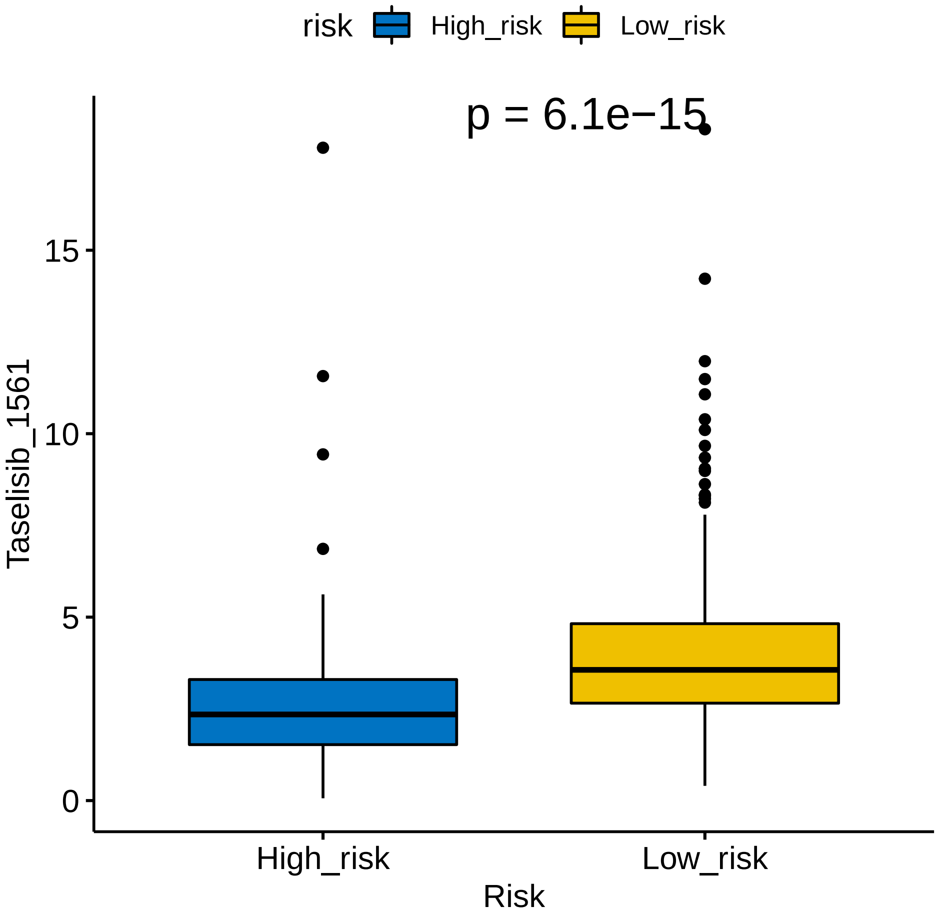
risk
High_risk
Low_risk
p = 6.1e−15
Taselisib_1561
0
5
10
15
High_risk
Low_risk
Risk

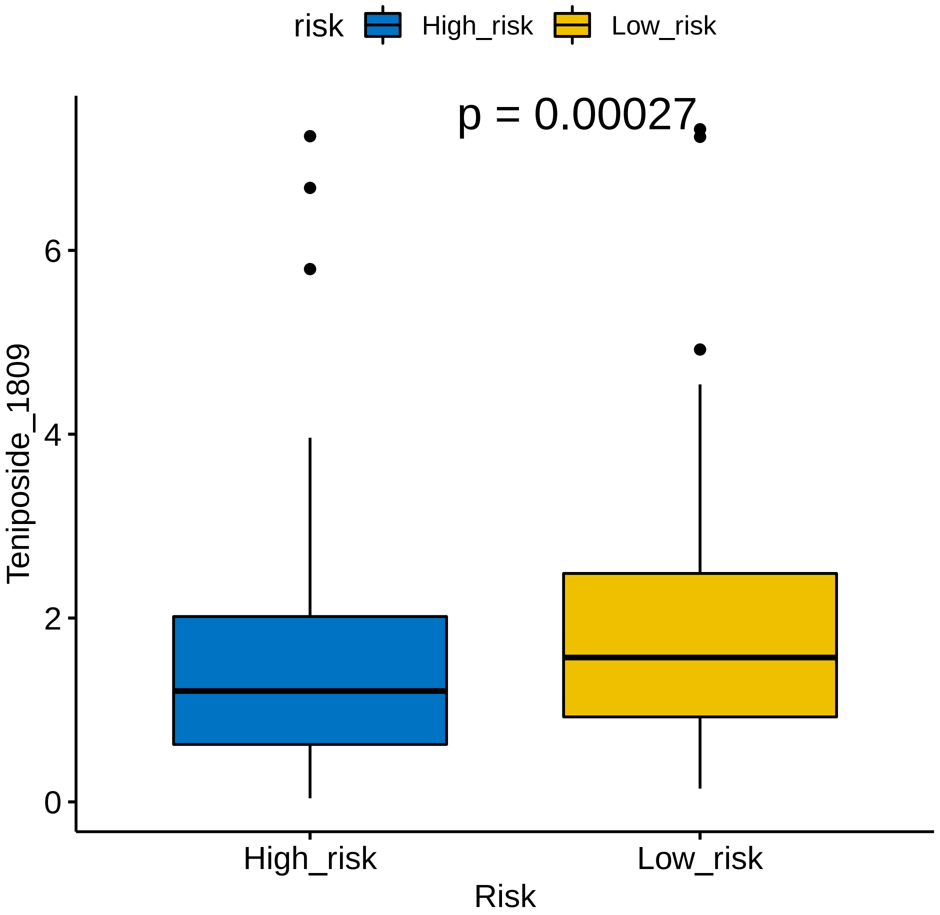
risk
High_risk
Low_risk
p = 0.00027
Teniposide_1809
0
2
4
6
High_risk
Low_risk
Risk

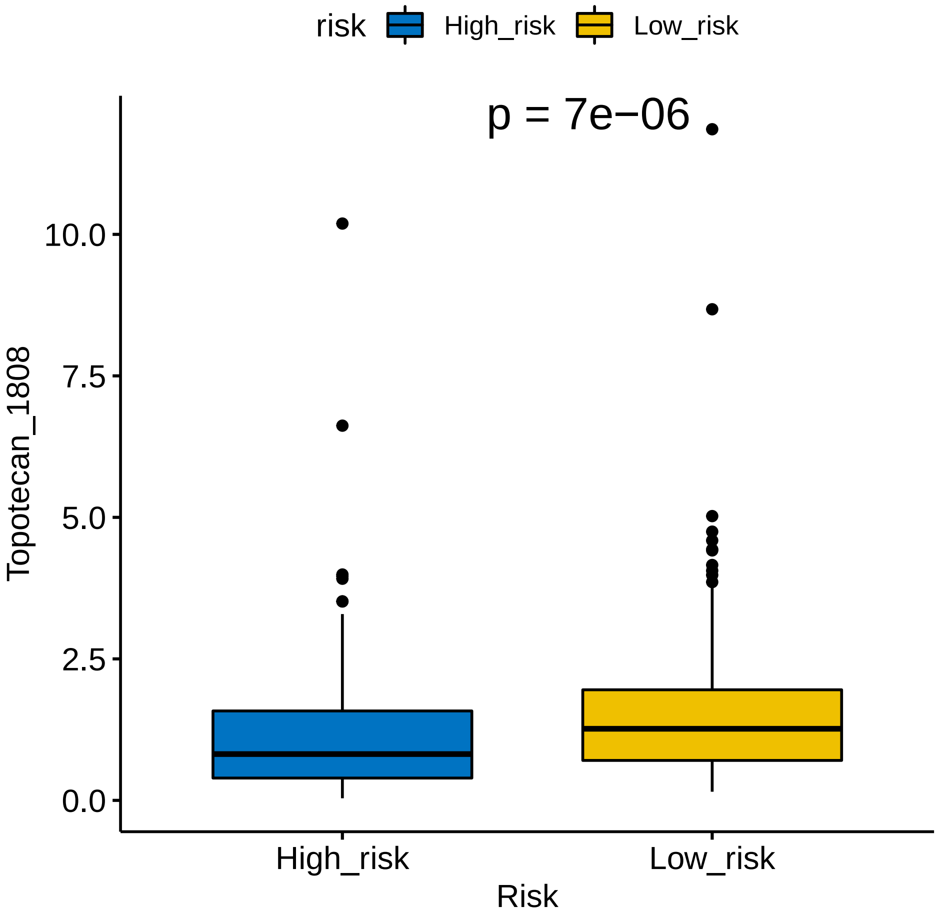

risk
High_risk
Low_risk
p = 7e−06
Topotecan_1808
10.0
7.5
5.0
2.5
0.0
High_risk
Low_risk
Risk

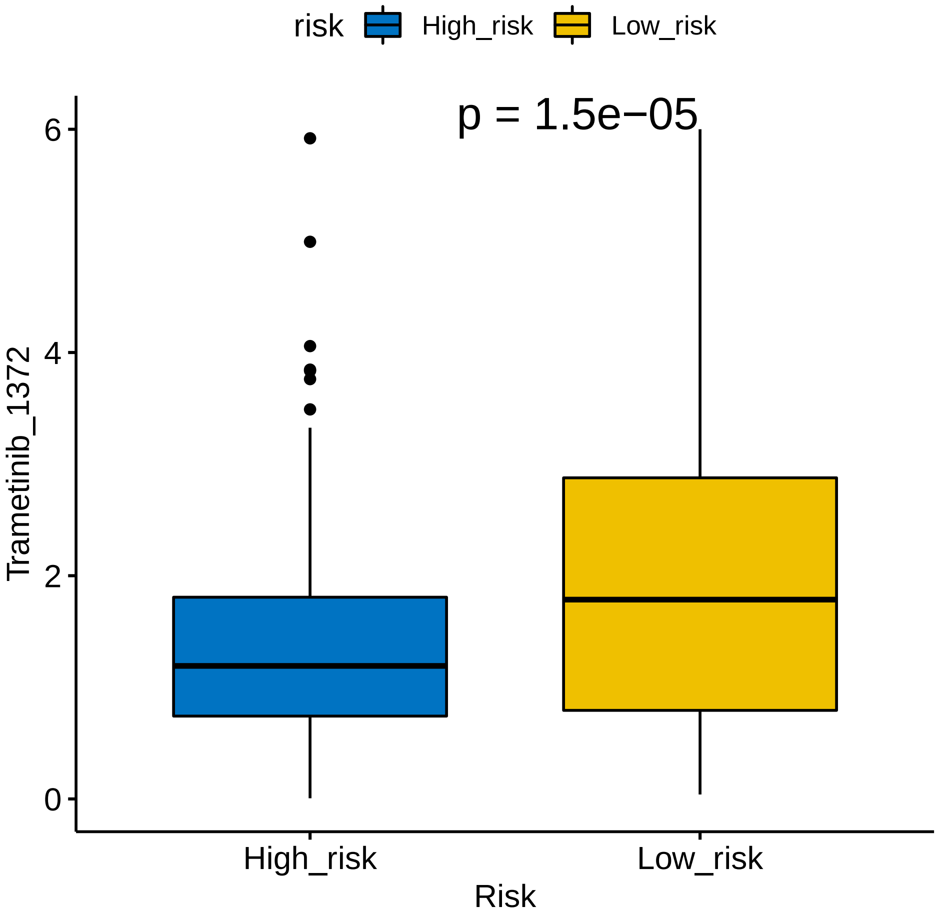

risk
High_risk
Low_risk
p = 1.5e−05
Trametinib_1372
6
4
2
0
High_risk
Low_risk
Risk

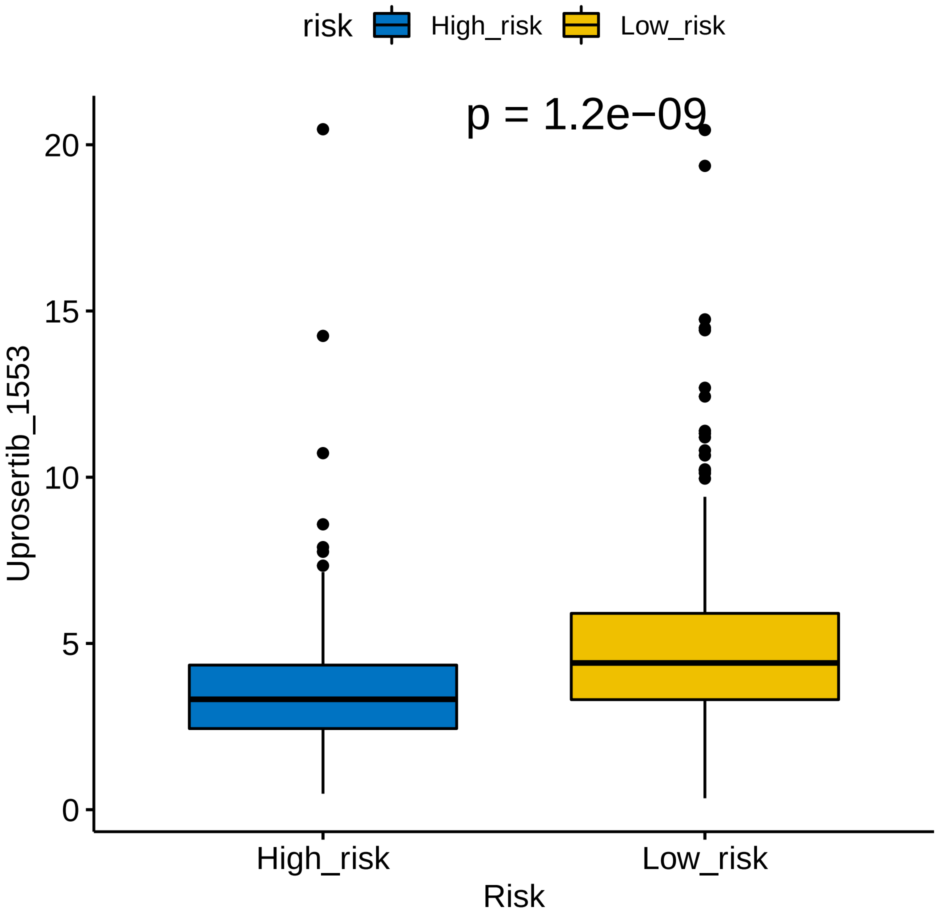

risk
High_risk
Low_risk
p = 1.2e−09
Uprosertib_1553
20
15
10
5
0
High_risk
Low_risk
Risk

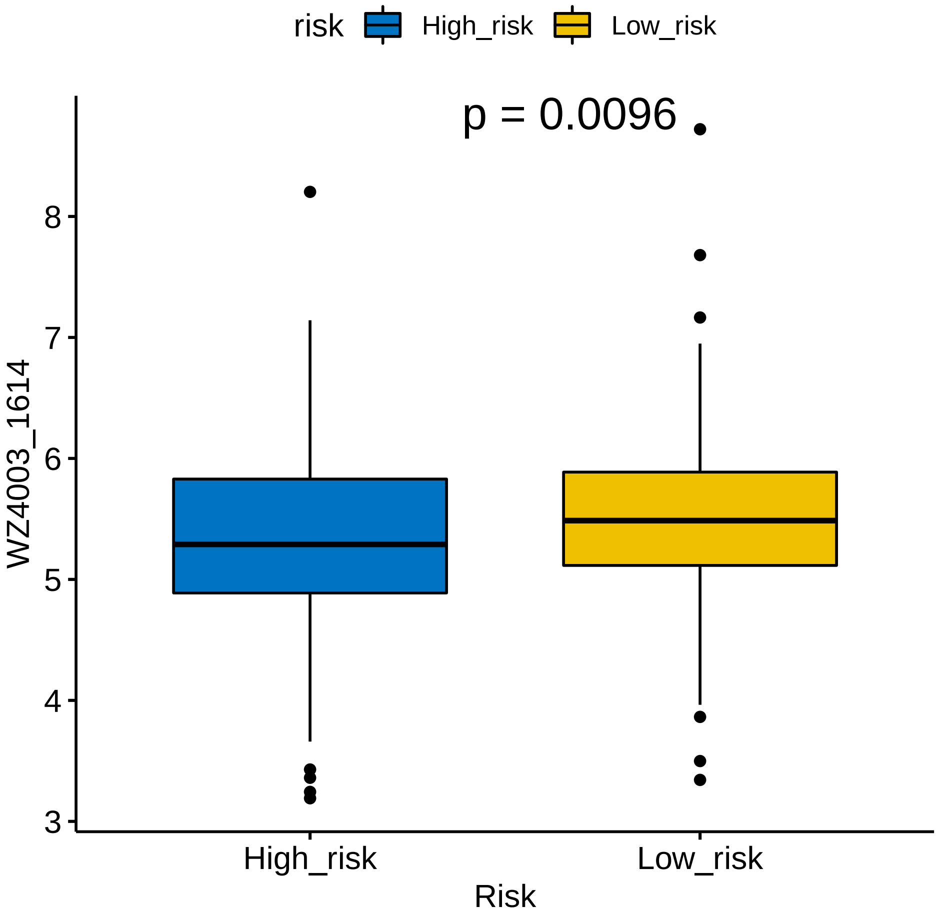
risk
High_risk
Low_risk
p = 0.0096
WZ4003_1614
8
7
6
5
4
3
High_risk
Low_risk
Risk

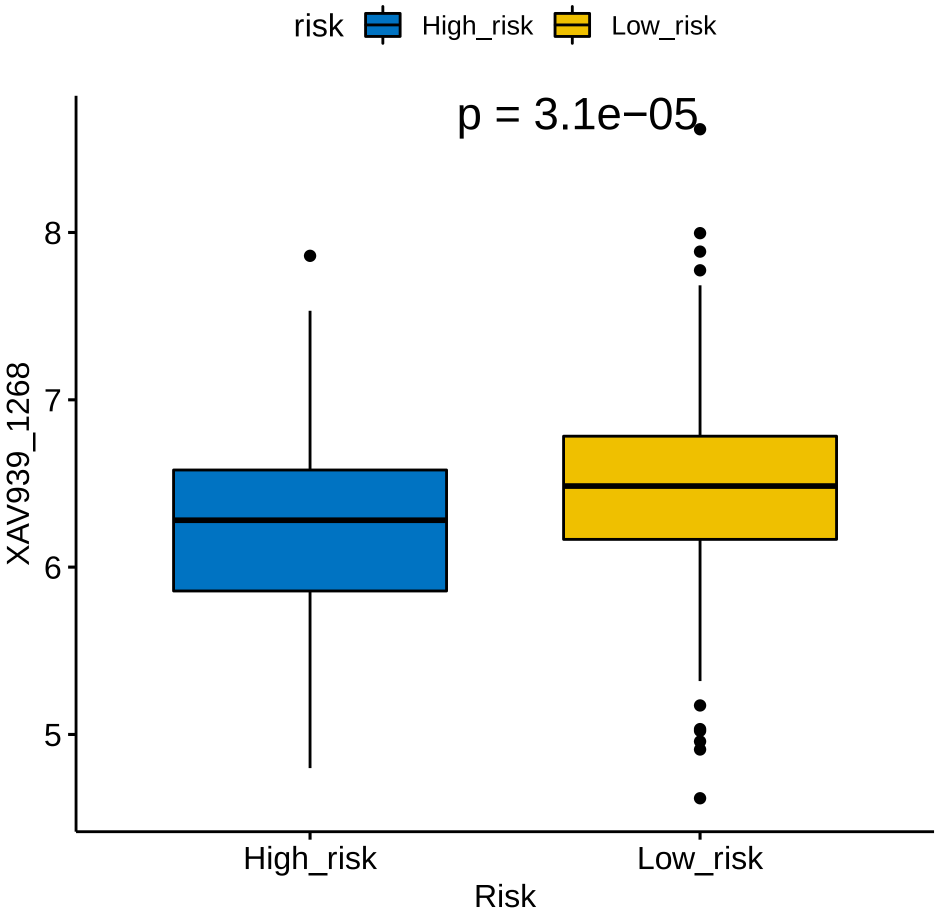
risk
High_risk
Low_risk
p = 3.1e−05
8
7
6
5
XAV939_1268
High_risk
Low_risk
Risk

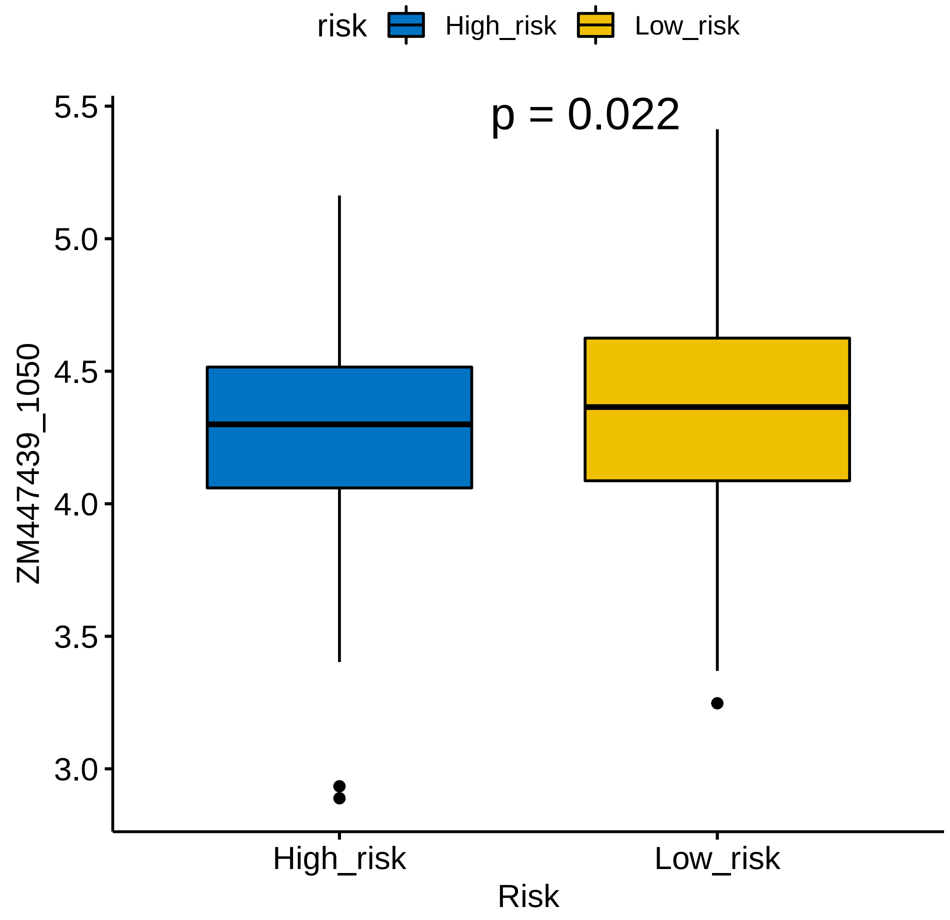
risk
High_risk
Low_risk
p = 0.022
5.5
5.0
4.5
4.0
3.5
3.0
ZM447439_1050
High_risk
Low_risk
Risk
