# Supplementary material for: Prognosis analysis and validation of lipid metabolism-associated lncRNAs and tumor immune microenvironment in bladder cancer
Source: Aging (Albany NY). 2023 Aug 24;15(16):8384–407. doi: 10.18632/aging.204975 (PMC10496992; doi:10.18632/aging.204975)
Supplement: Supplementary Figure 3 [file aging-15-204975-s003.pdf]

Supplementary Figure 3. 98 chemotherapeutic drugs were sensitive in the low-risk group.

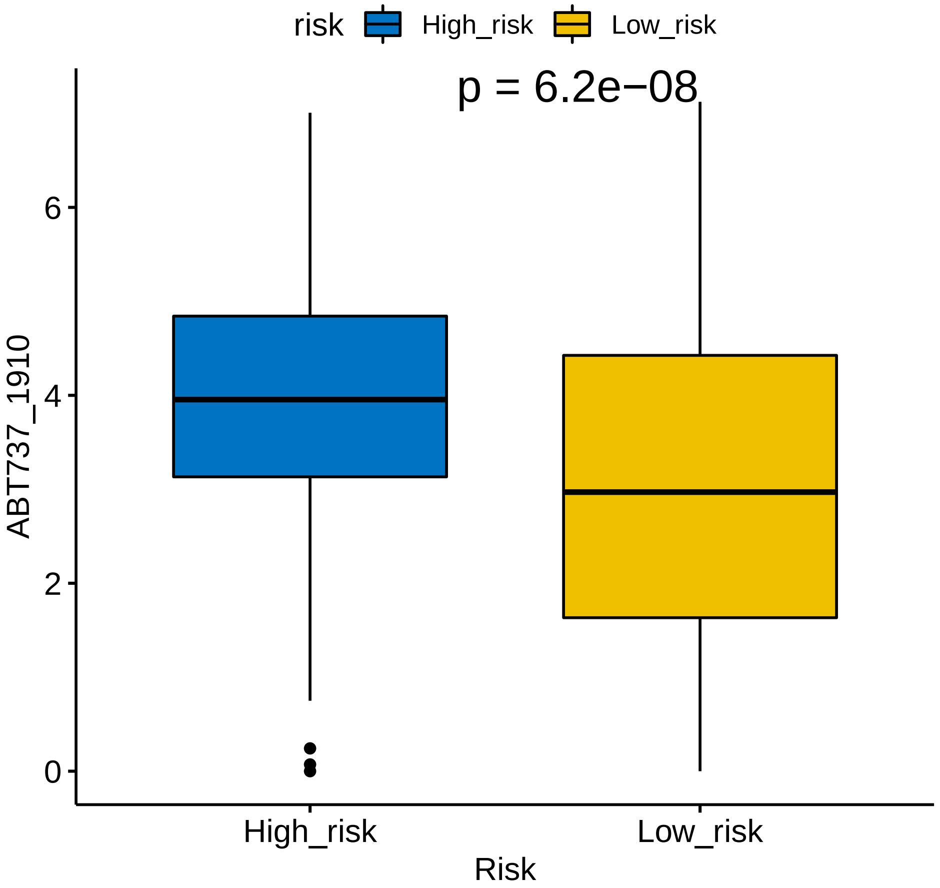

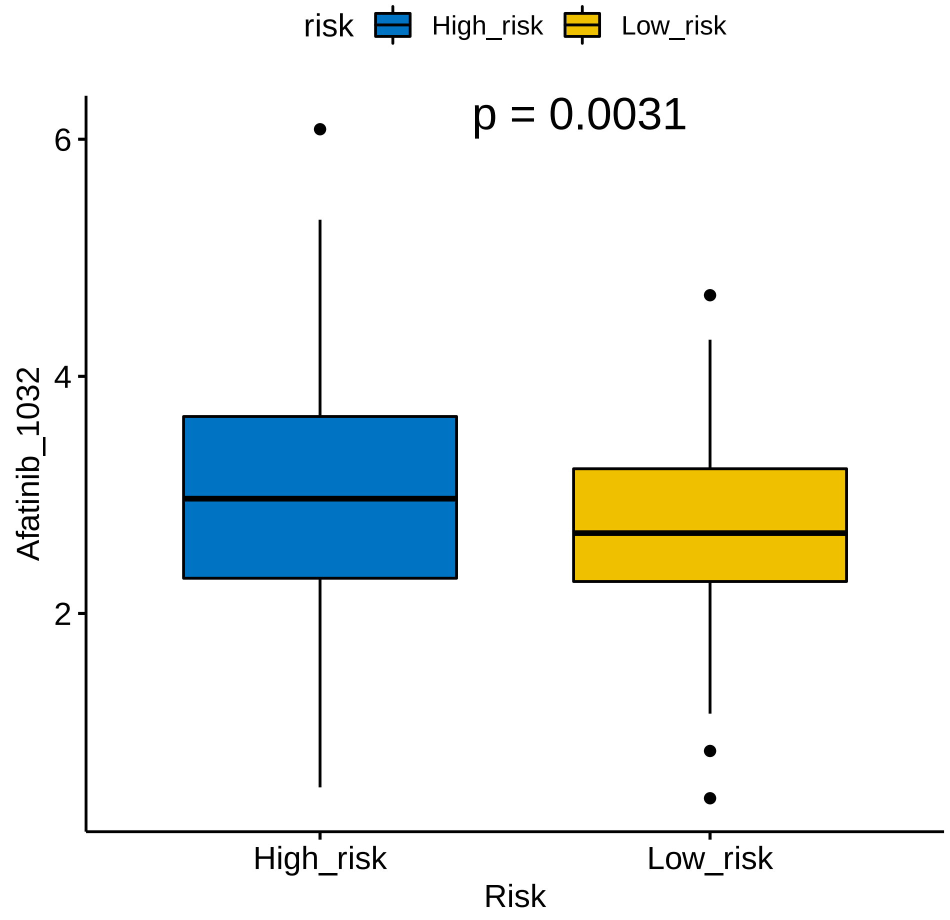

risk
High_risk
Low_risk
p = 0.0031
Afatinib_1032
6
4
2
High_risk
Low_risk
Risk

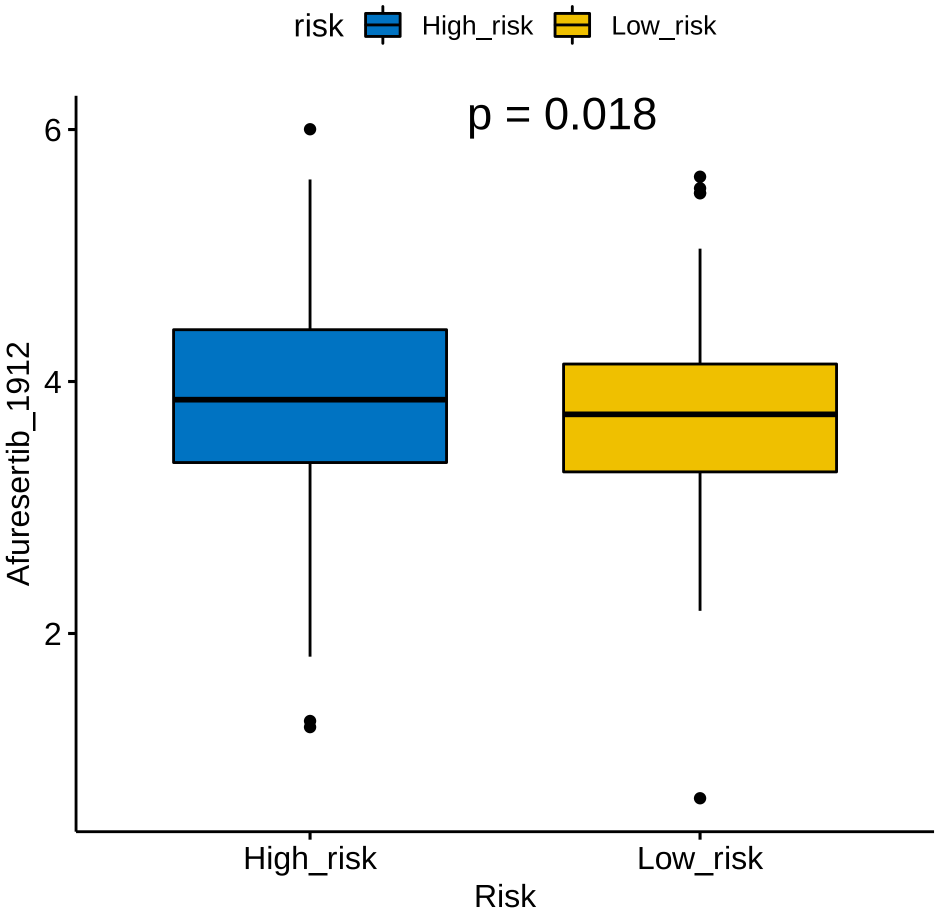

risk
High_risk
Low_risk
p = 0.018
6
4
2
Afuresertib_1912
High_risk
Low_risk
Risk

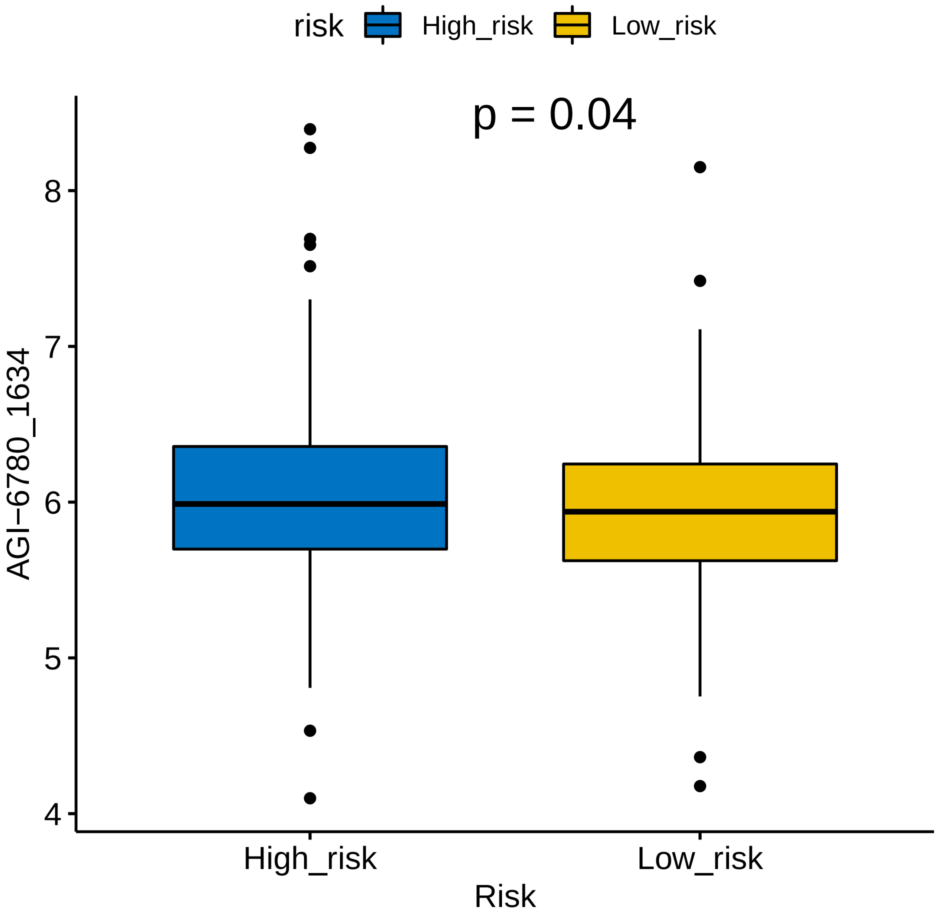
risk
High_risk
Low_risk
p = 0.04
AGI−6780_1634
8
7
6
5
4
High_risk
Low_risk
Risk

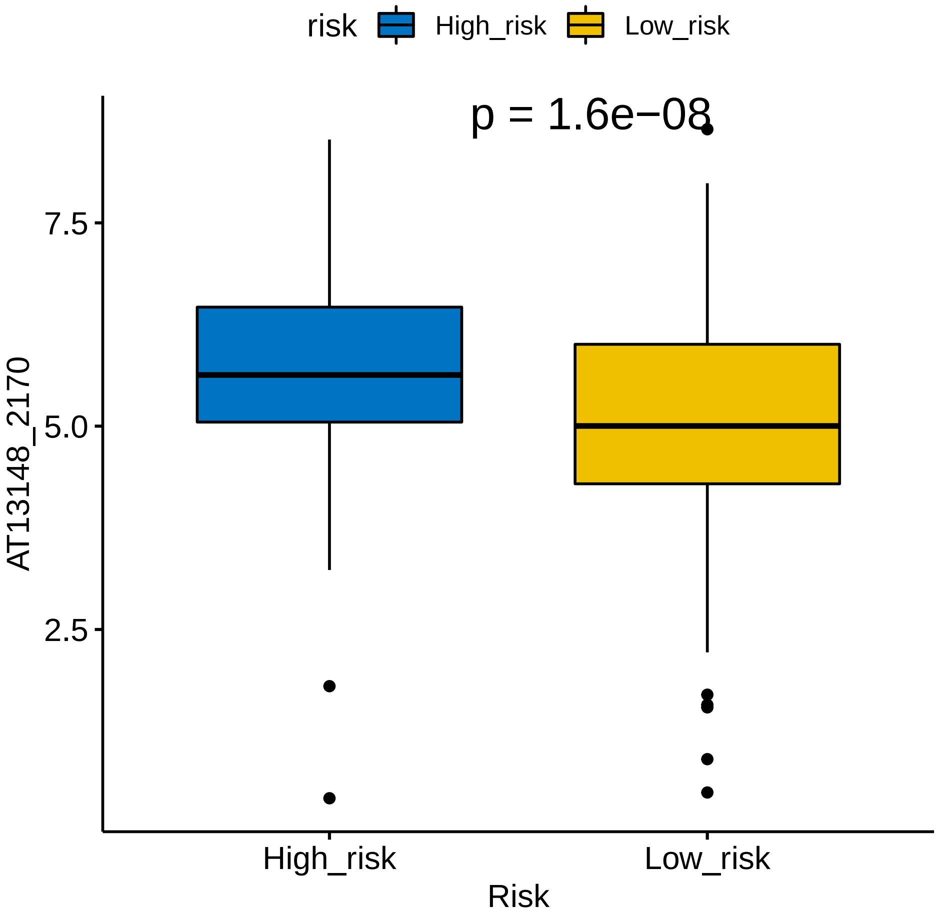

risk
High_risk
Low_risk
p = 1.6e−08
7.5
5.0
2.5
AT13148_2170
High_risk
Low_risk
Risk

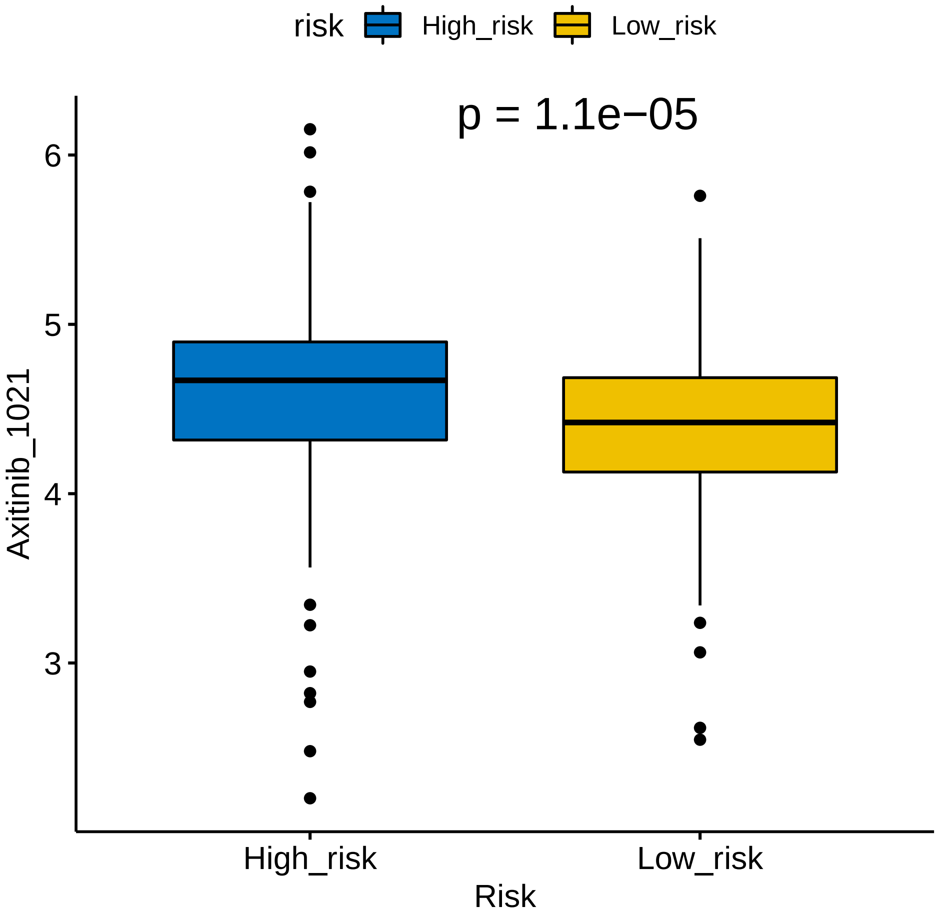
risk
High_risk
Low_risk
p = 1.1e−05
6
5
4
3
Axitinib_1021
High_risk
Low_risk
Risk

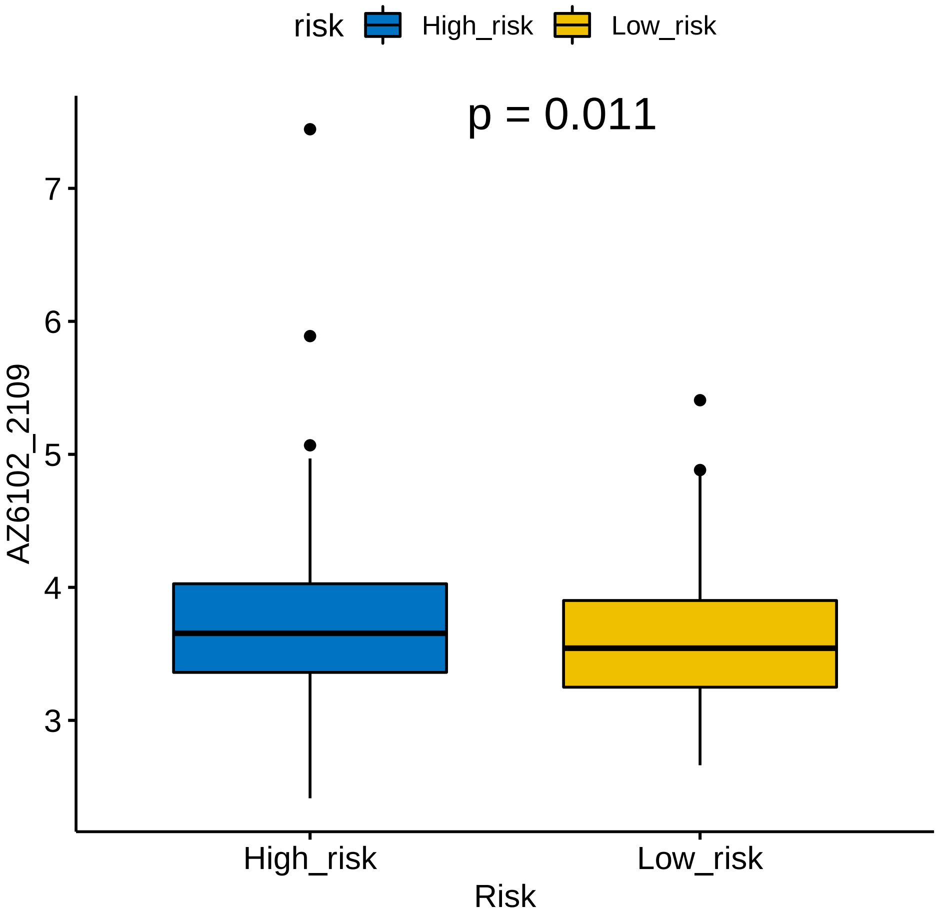

risk
High_risk
Low_risk
p = 0.011
AZ6102_2109
7
6
5
4
3
High_risk
Low_risk
Risk

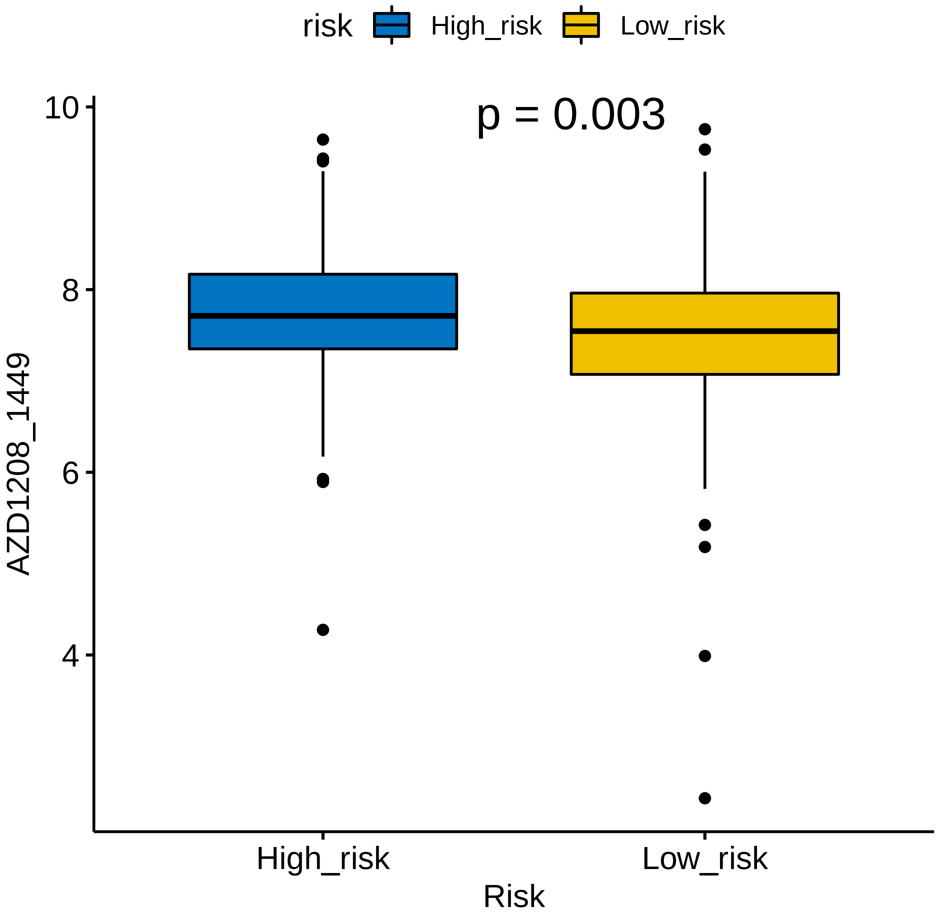

risk
High_risk
Low_risk
p = 0.003
10
8
6
4
AZD1208_1449
High_risk
Low_risk
Risk

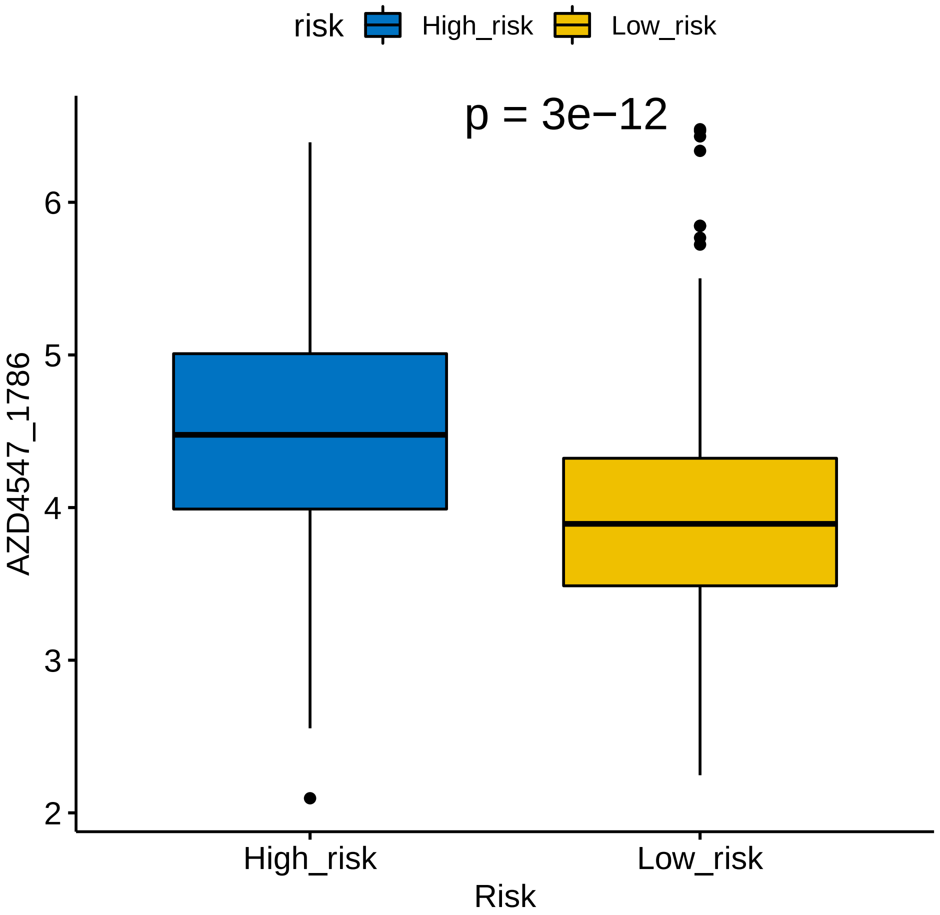
risk
High_risk
Low_risk
p = 3e−12
AZD4547_1786
6
5
4
3
2
High_risk
Low_risk
Risk

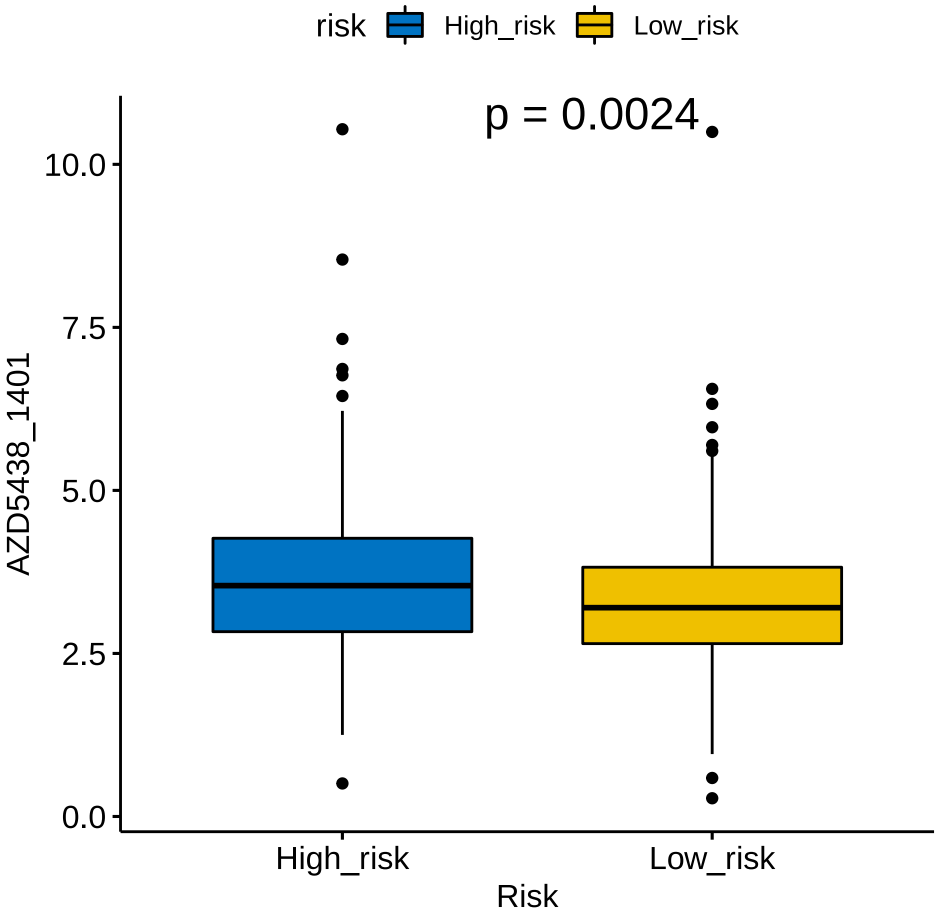

risk
High_risk
Low_risk
p = 0.0024
AZD5438_1401
10.0
7.5
5.0
2.5
0.0
High_risk
Low_risk
Risk

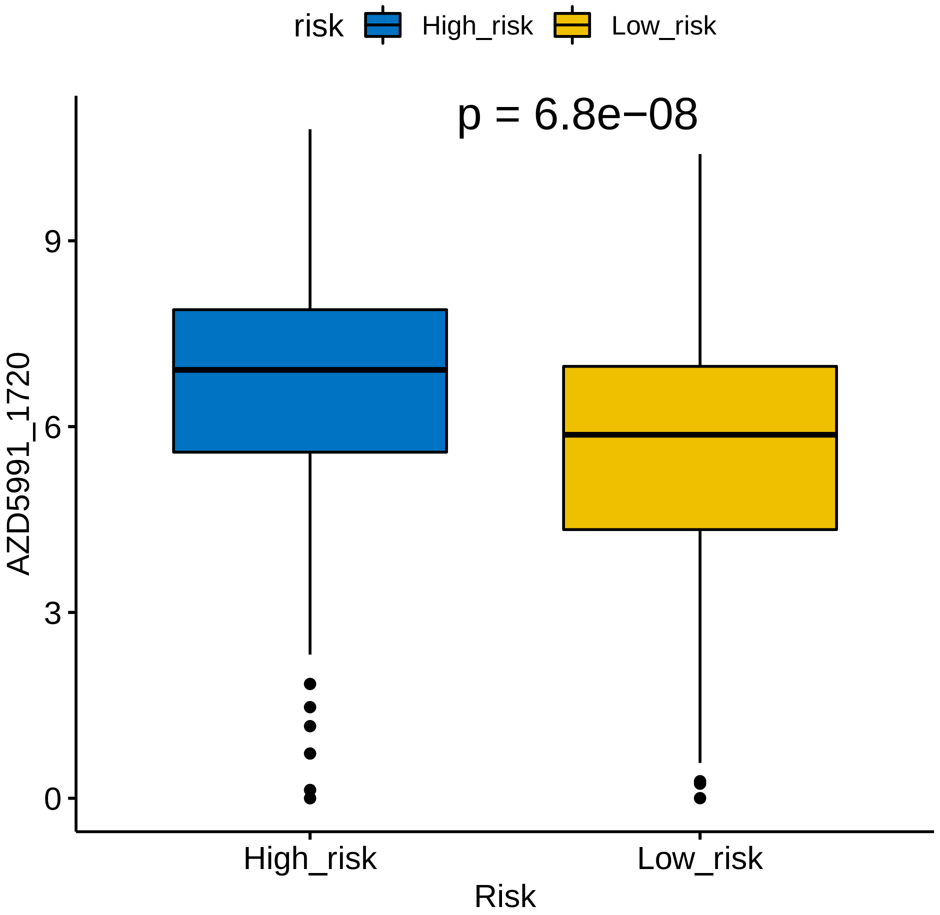

risk
High_risk
Low_risk
p = 6.8e−08
AZD5991_1720
9
6
3
0
High_risk
Low_risk
Risk

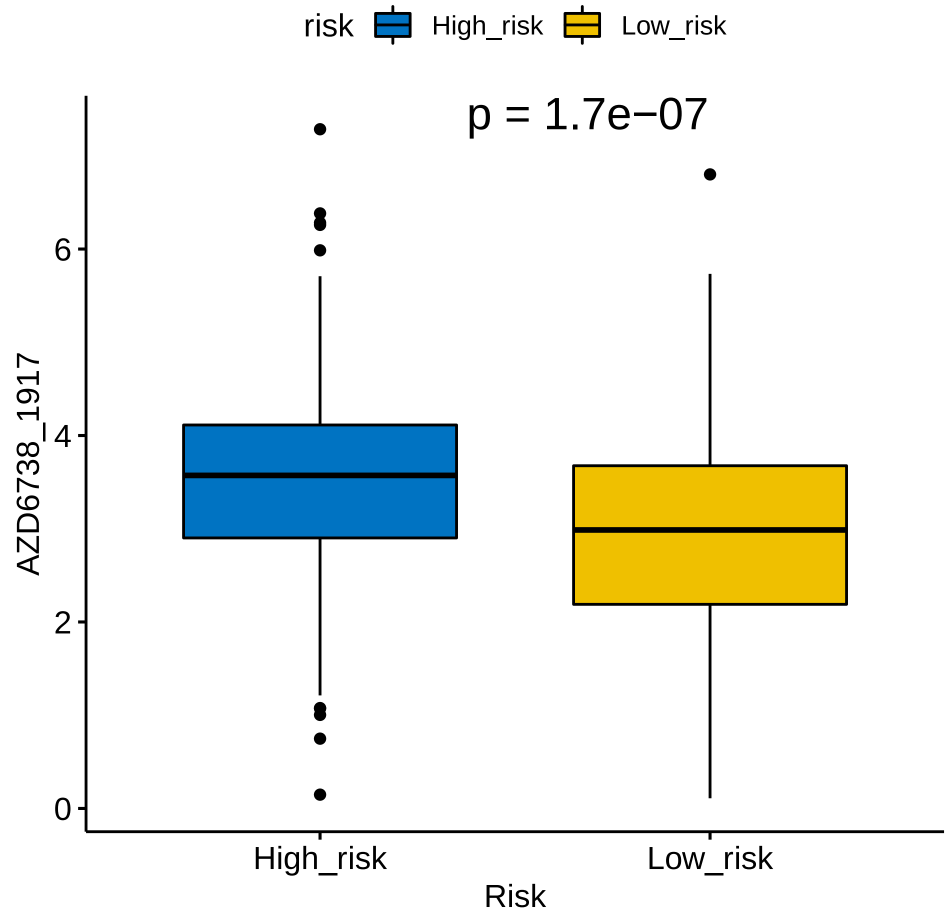

risk
High_risk
Low_risk
p = 1.7e−07
AZD6738_1917
0
2
4
6
High_risk
Low_risk
Risk

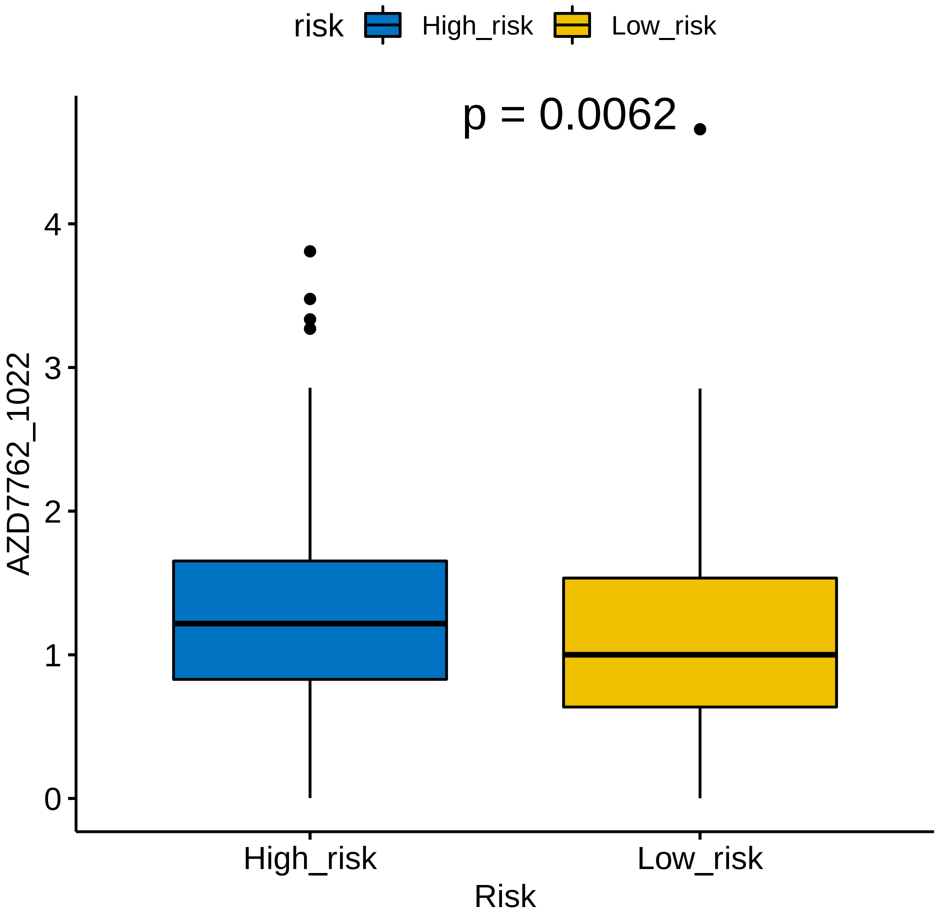
risk
High_risk
Low_risk
p = 0.0062
AZD7762_1022
0
1
2
3
4
High_risk
Low_risk
Risk

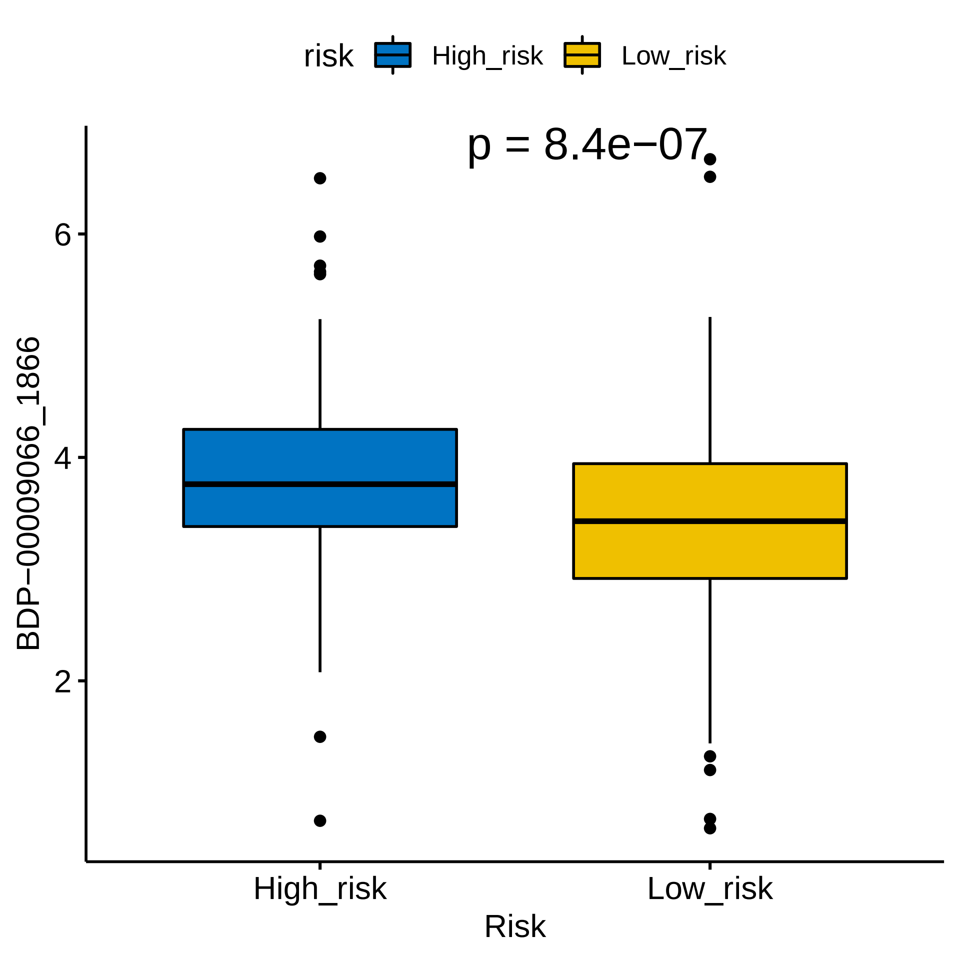
risk
High_risk
Low_risk
p = 8.4e−07
BDP−00009066_1866
6
4
2
High_risk
Low_risk
Risk

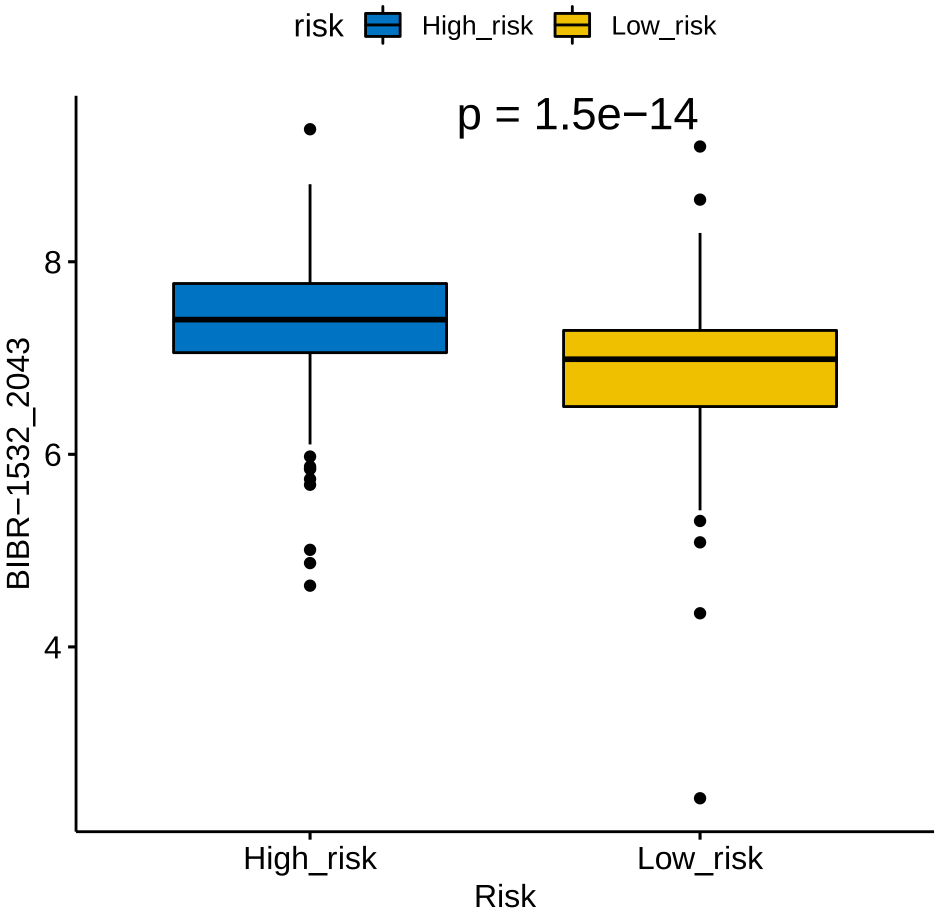
risk
High_risk
Low_risk
p = 1.5e−14
8
6
4
BIBR−1532_2043
High_risk
Low_risk
Risk

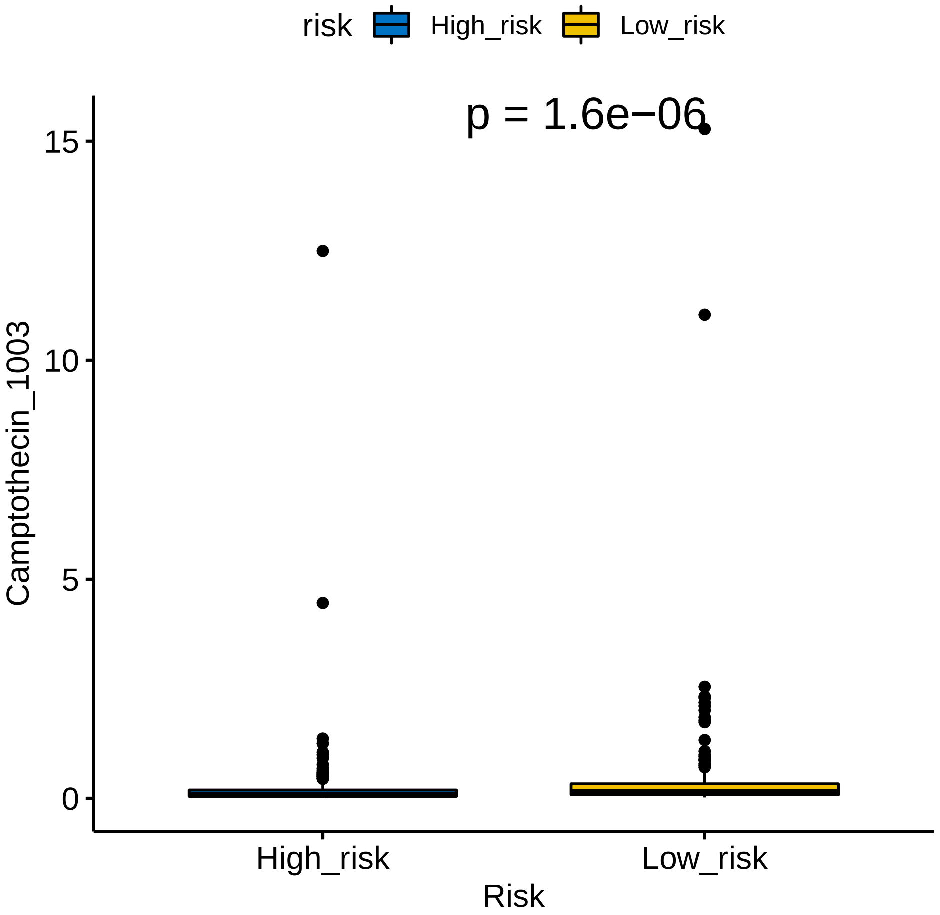
risk
High_risk
Low_risk
p = 1.6e−06
15
10
5
0
Camptothecin_1003
High_risk
Low_risk
Risk

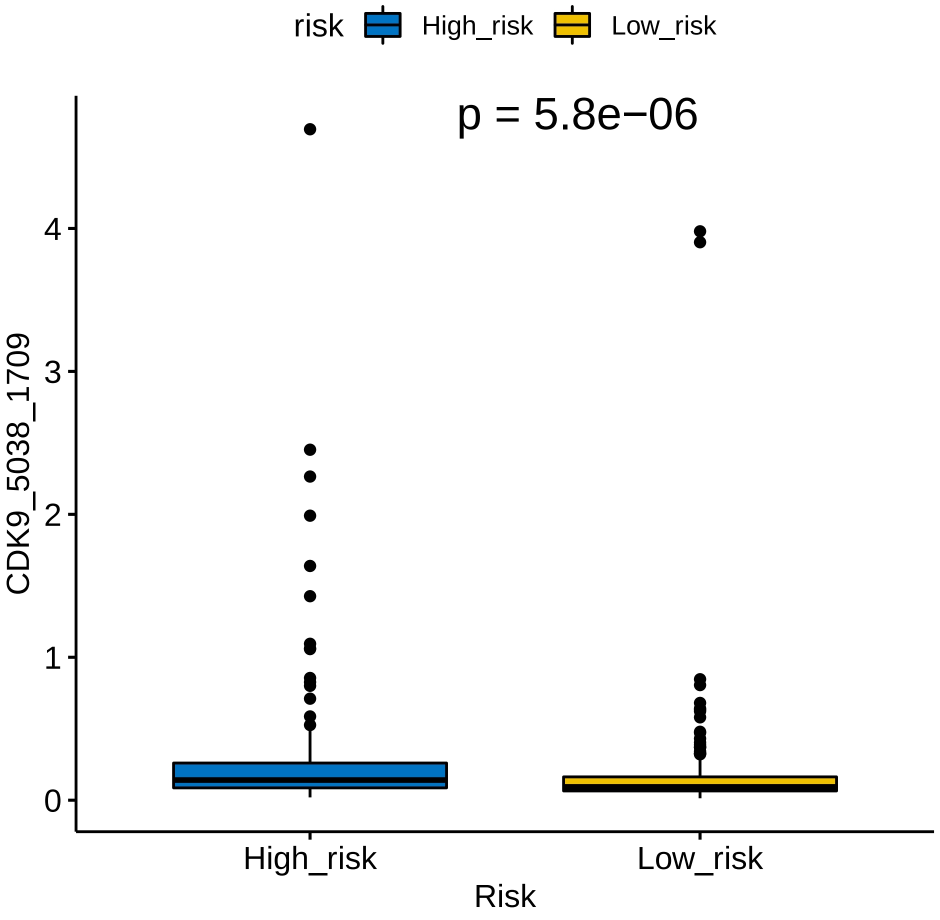

risk
High_risk
Low_risk
p = 5.8e−06
CDK9_5038_1709
0
1
2
3
4
High_risk
Low_risk
Risk

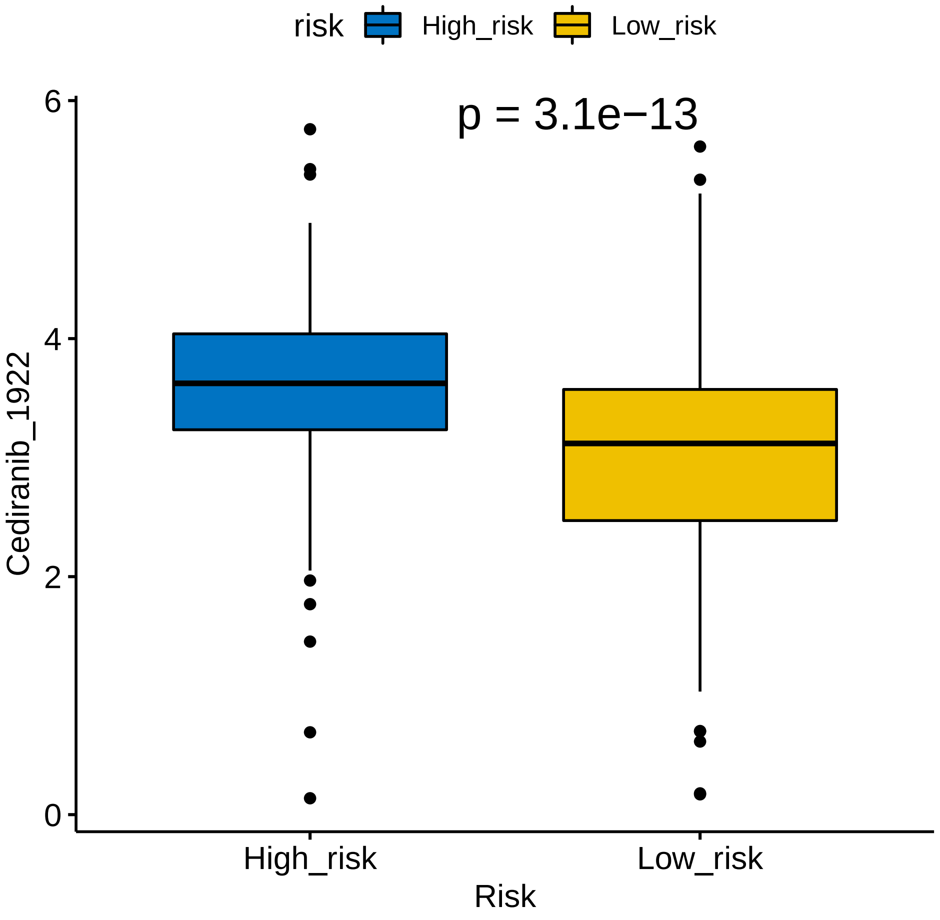

risk
High_risk
Low_risk
p = 3.1e−13
6
4
2
0
Cediranib_1922
High_risk
Low_risk
Risk

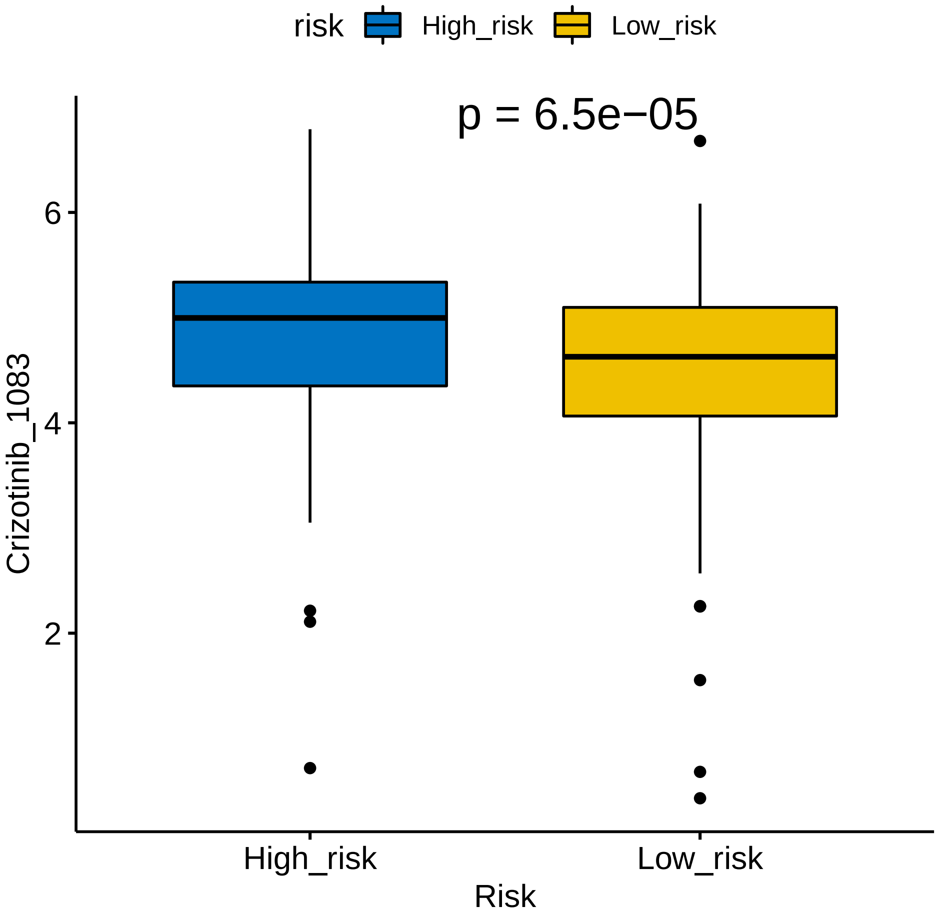
risk
High_risk
Low_risk
p = 6.5e−05
6
4
2
Crizotinib_1083
High_risk
Low_risk
Risk

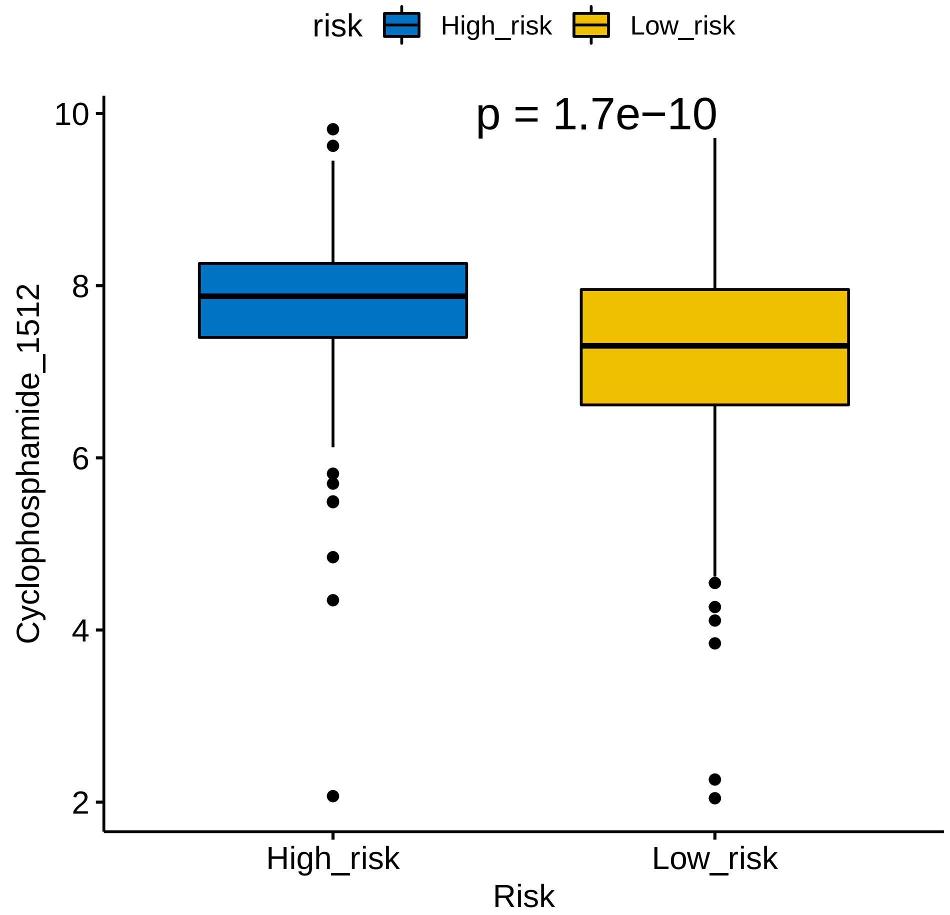

risk
High_risk
Low_risk
p = 1.7e−10
10
8
6
4
2
Cyclophosphamide_1512
High_risk
Low_risk
Risk

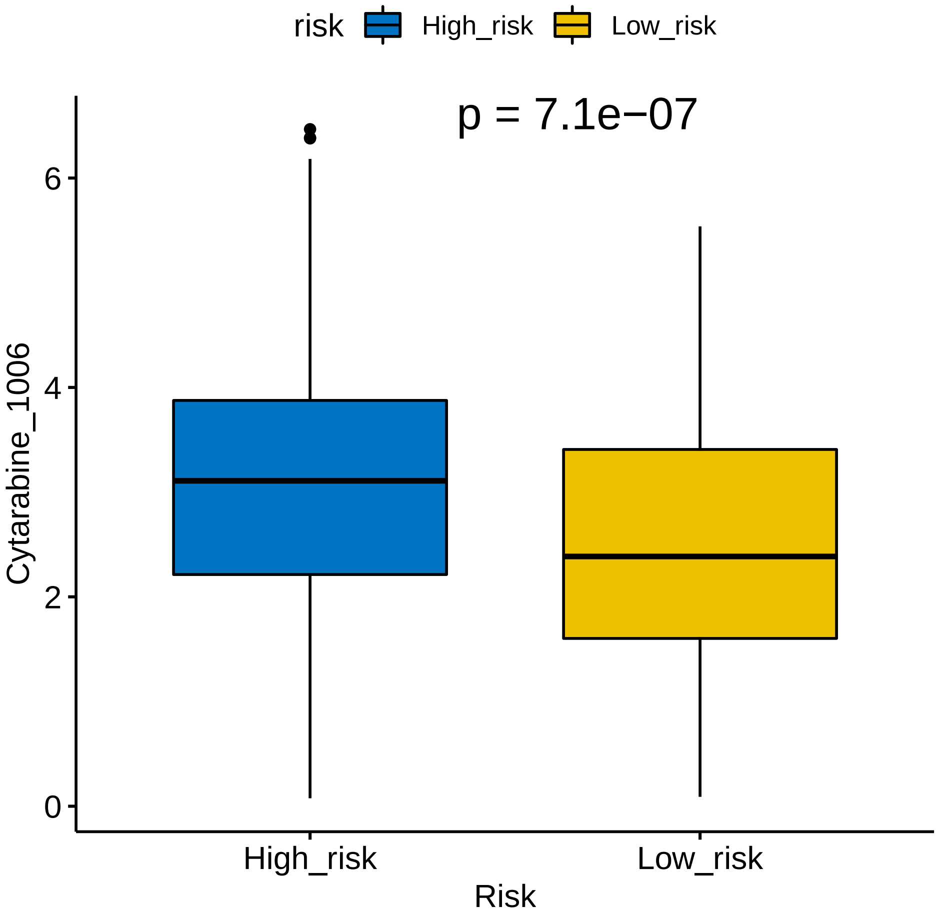
risk
High_risk
Low_risk
p = 7.1e−07
Cytarabine_1006
6
4
2
0
High_risk
Low_risk
Risk

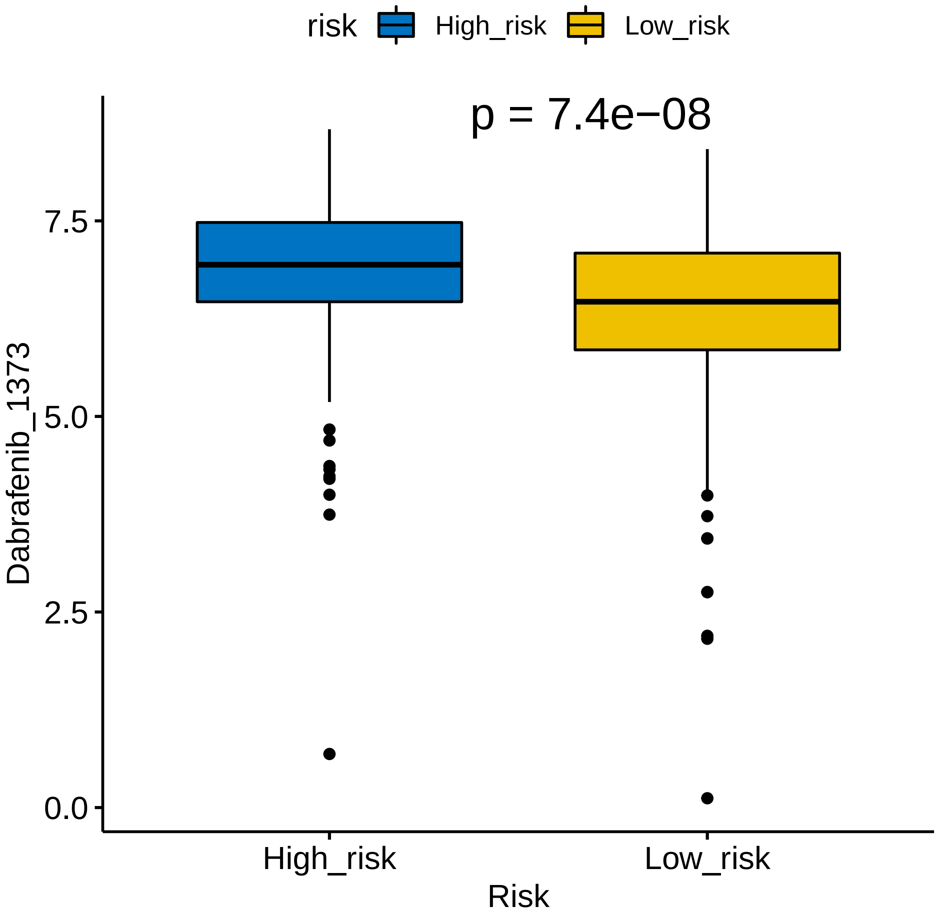

risk
High_risk
Low_risk
p = 7.4e−08
7.5
5.0
2.5
0.0
Dabrafenib_1373
High_risk
Low_risk
Risk

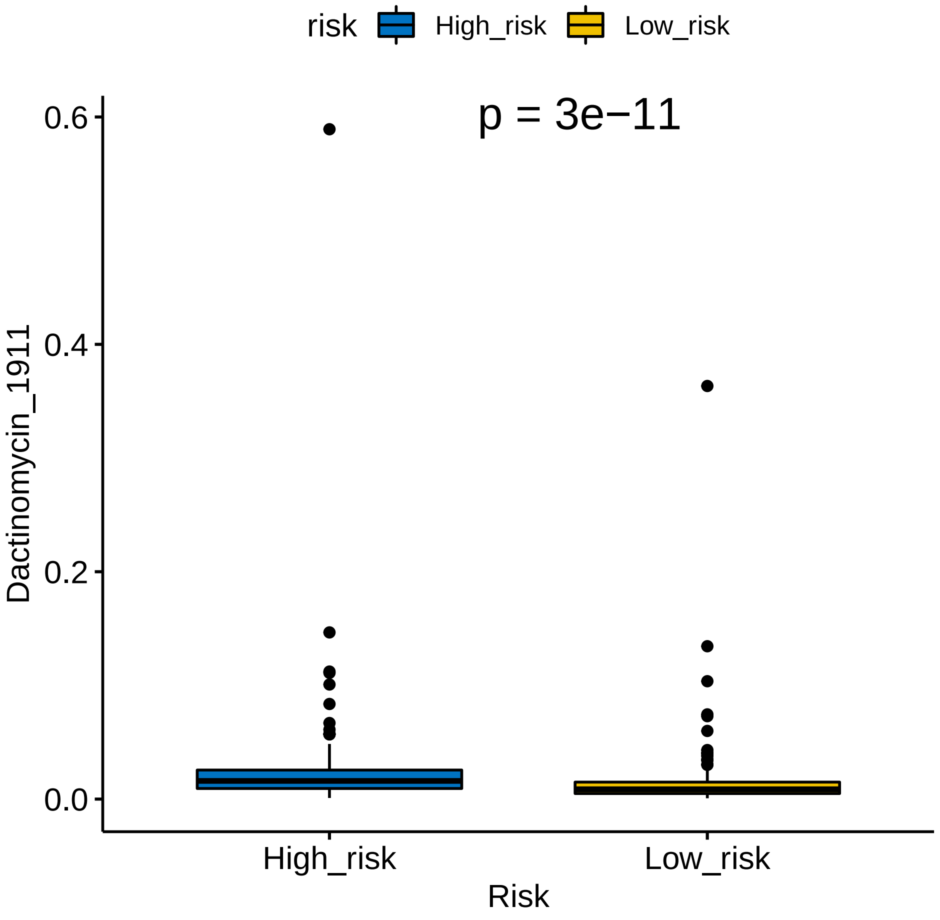

risk
High_risk
Low_risk
p = 3e−11
0.6
0.4
0.2
0.0
Dactinomycin_1911
High_risk
Low_risk
Risk

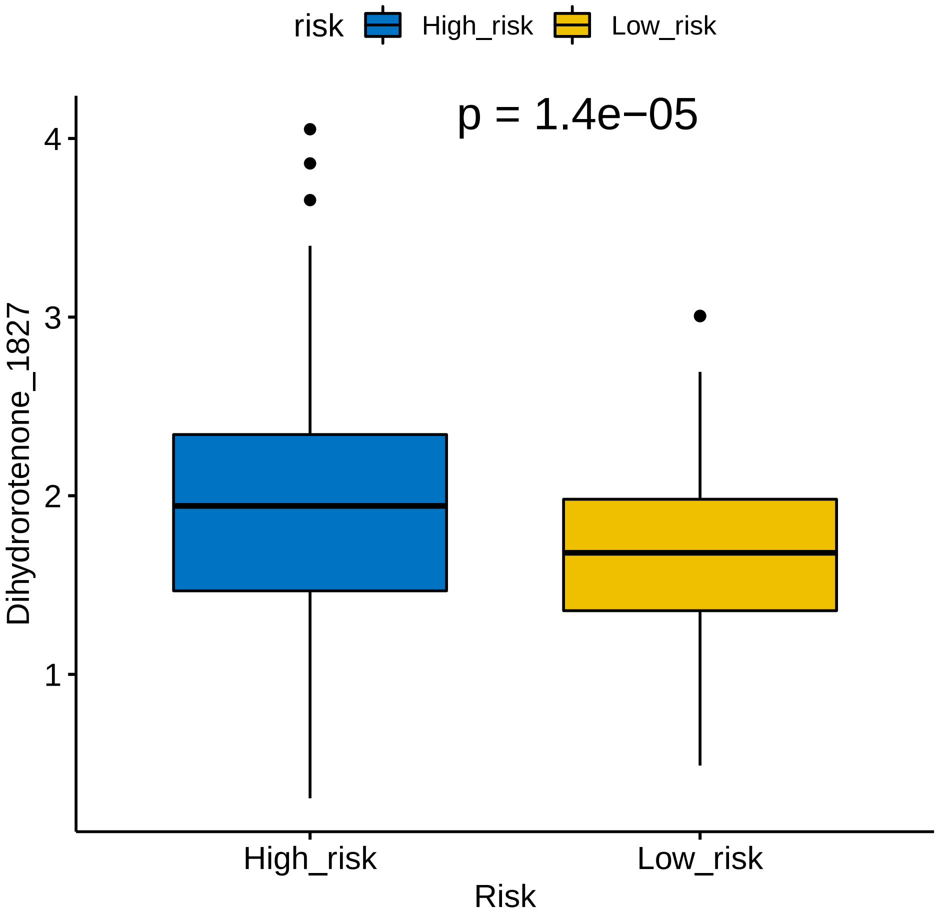

risk
High_risk
Low_risk
p = 1.4e−05
4
3
2
1
Dihydrorotenone_1827
High_risk
Low_risk
Risk

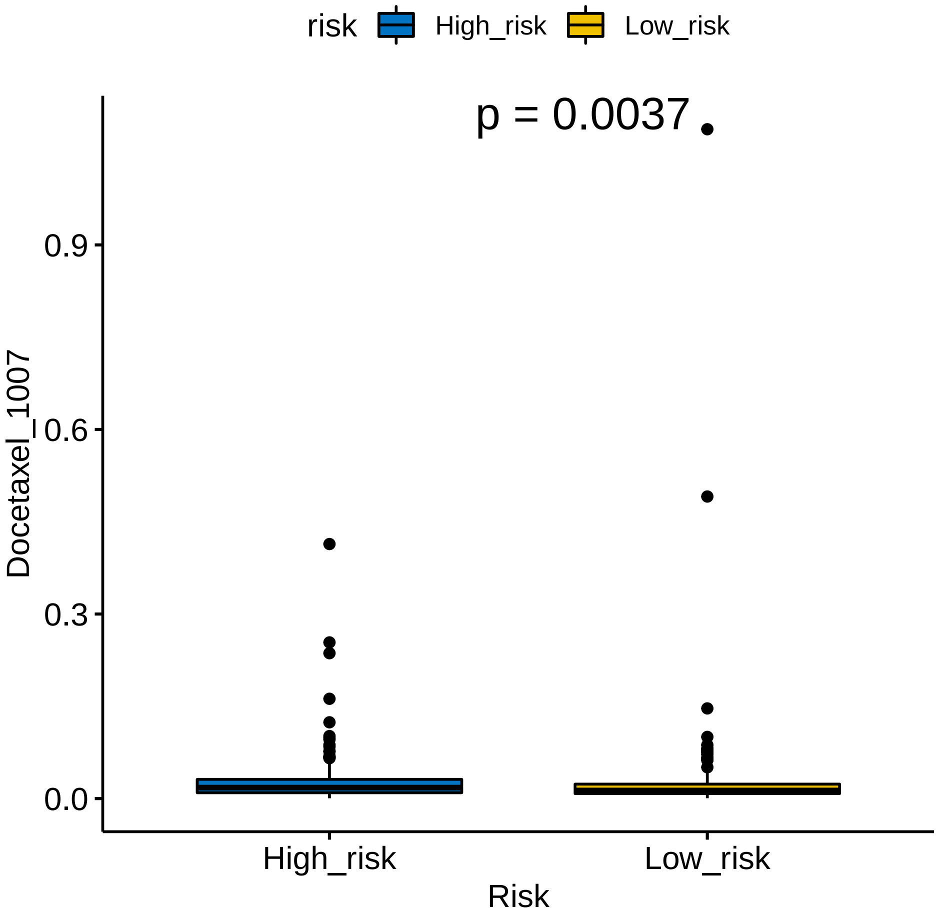
risk
High_risk
Low_risk
p = 0.0037
0.9
0.6
0.3
0.0
Docetaxel_1007
High_risk
Low_risk
Risk

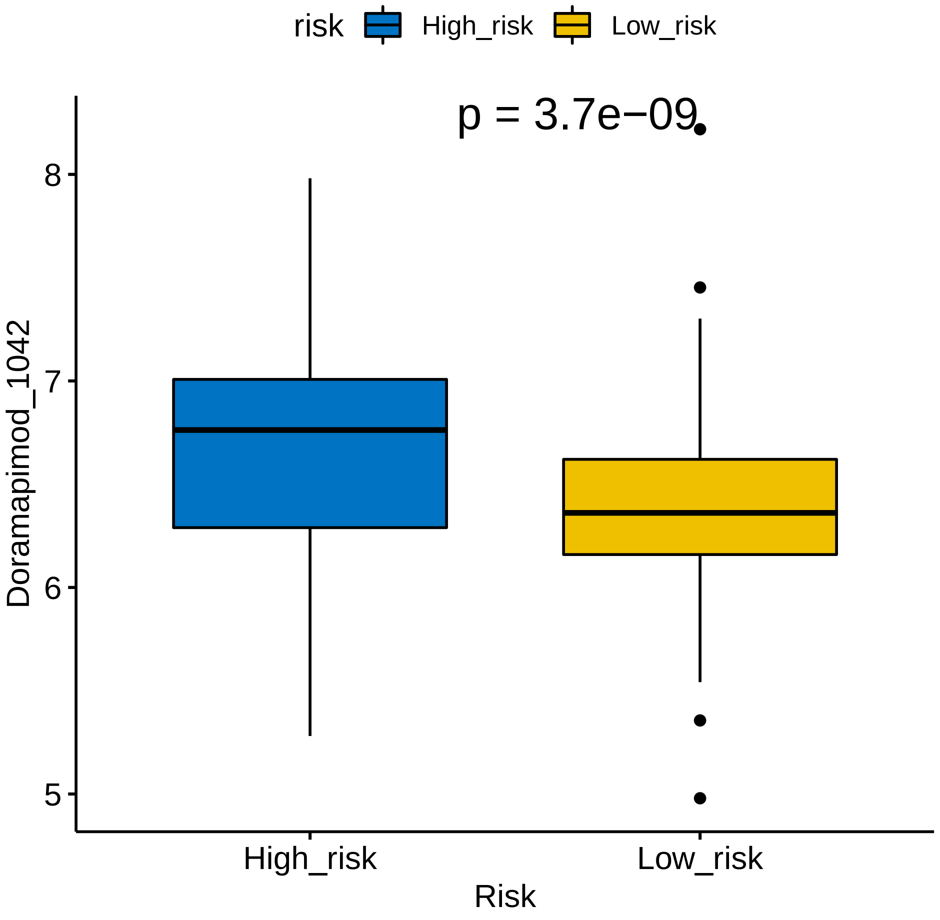

risk
High_risk
Low_risk
p = 3.7e−09
Doramapimod_1042
8
7
6
5
High_risk
Low_risk
Risk

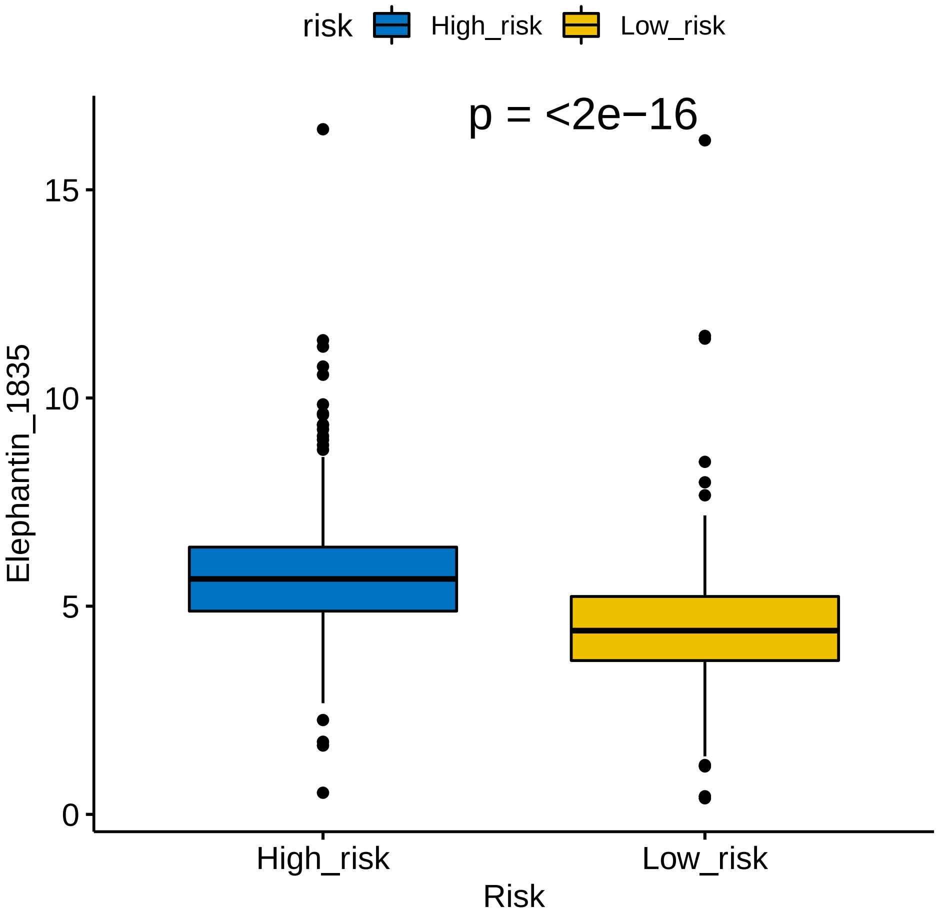
risk
High_risk
Low_risk
p = <2e−16
15
10
5
0
Elephantin_1835
High_risk
Low_risk
Risk

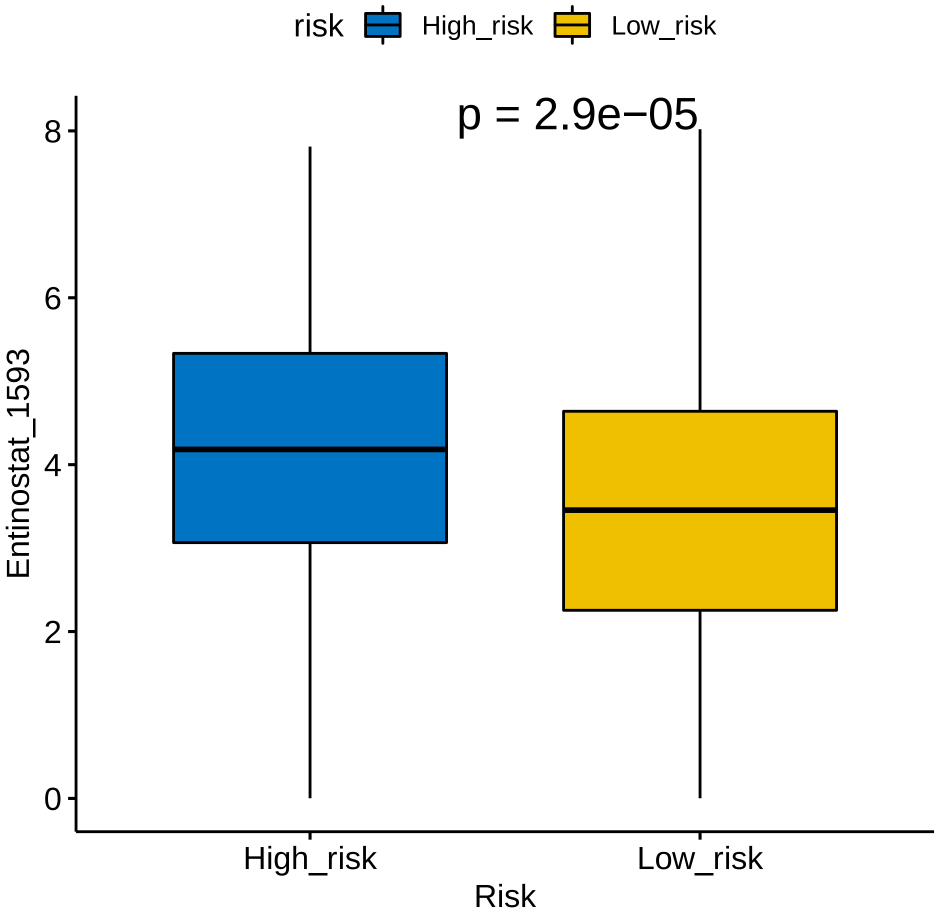

risk
High_risk
Low_risk
p = 2.9e−05
Entinostat_1593
8
6
4
2
0
High_risk
Low_risk
Risk

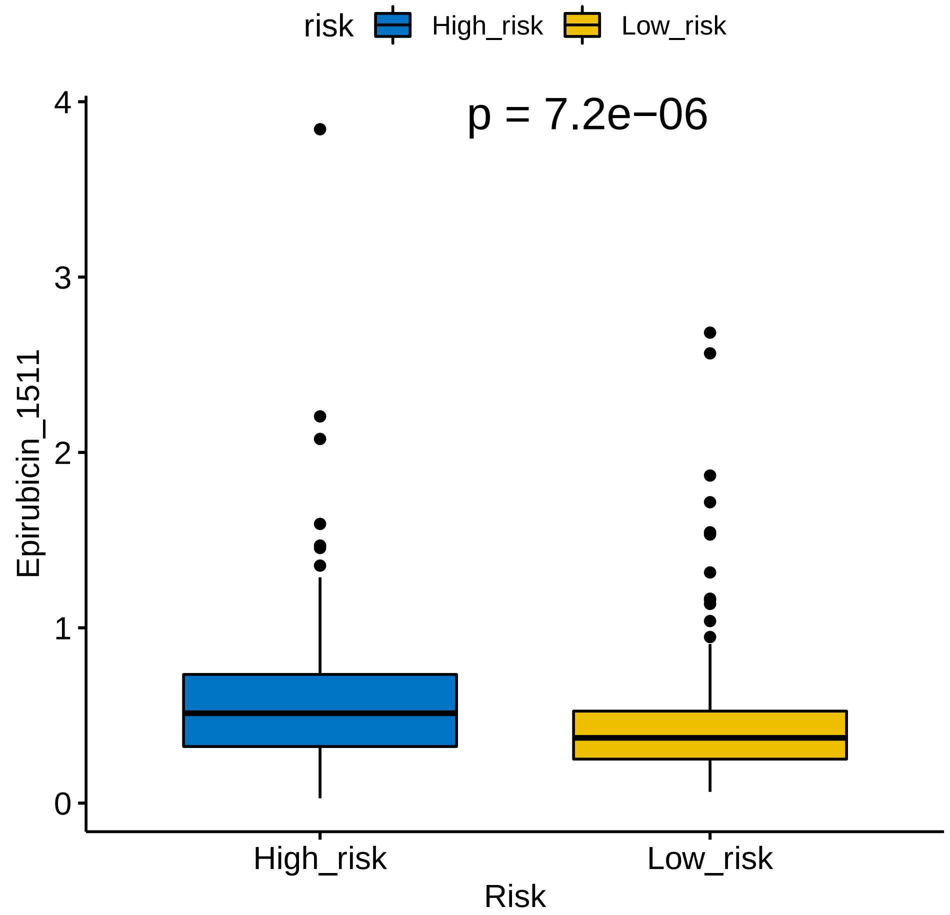
risk
High_risk
Low_risk
p = 7.2e−06
Epirubicin_1511
4
3
2
1
0
High_risk
Low_risk
Risk

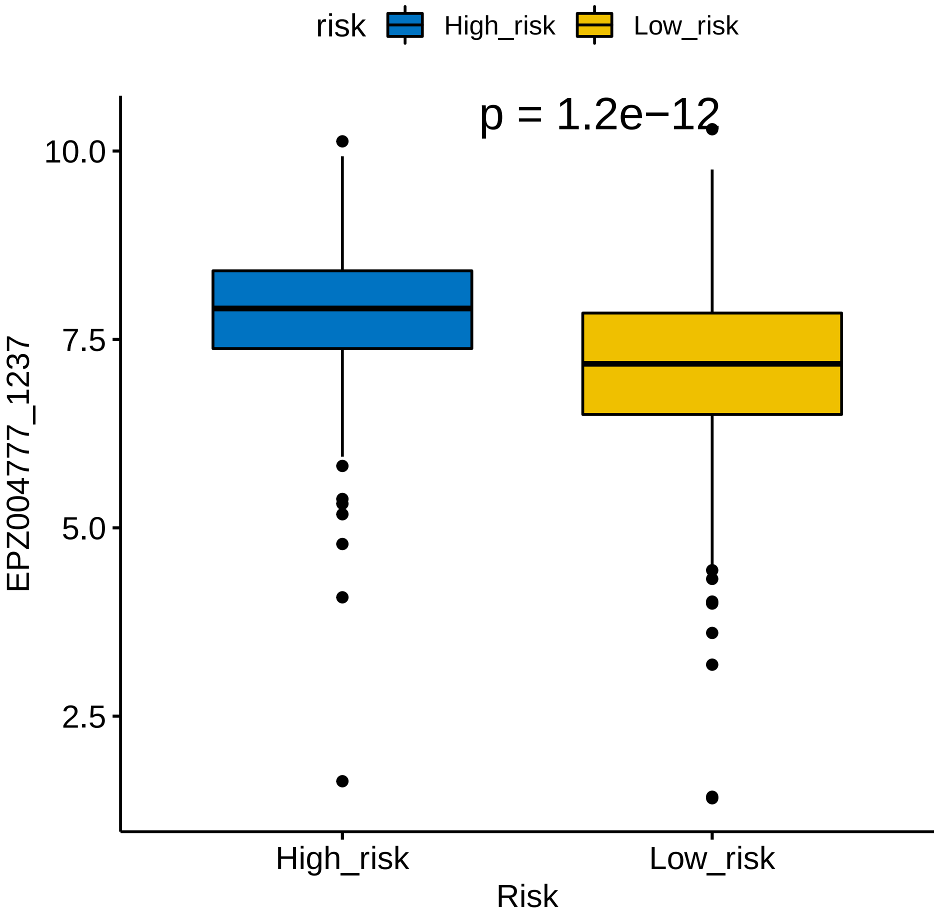
risk
High_risk
Low_risk
p = 1.2e−12
10.0
7.5
5.0
2.5
EPZ004777_1237
High_risk
Low_risk
Risk

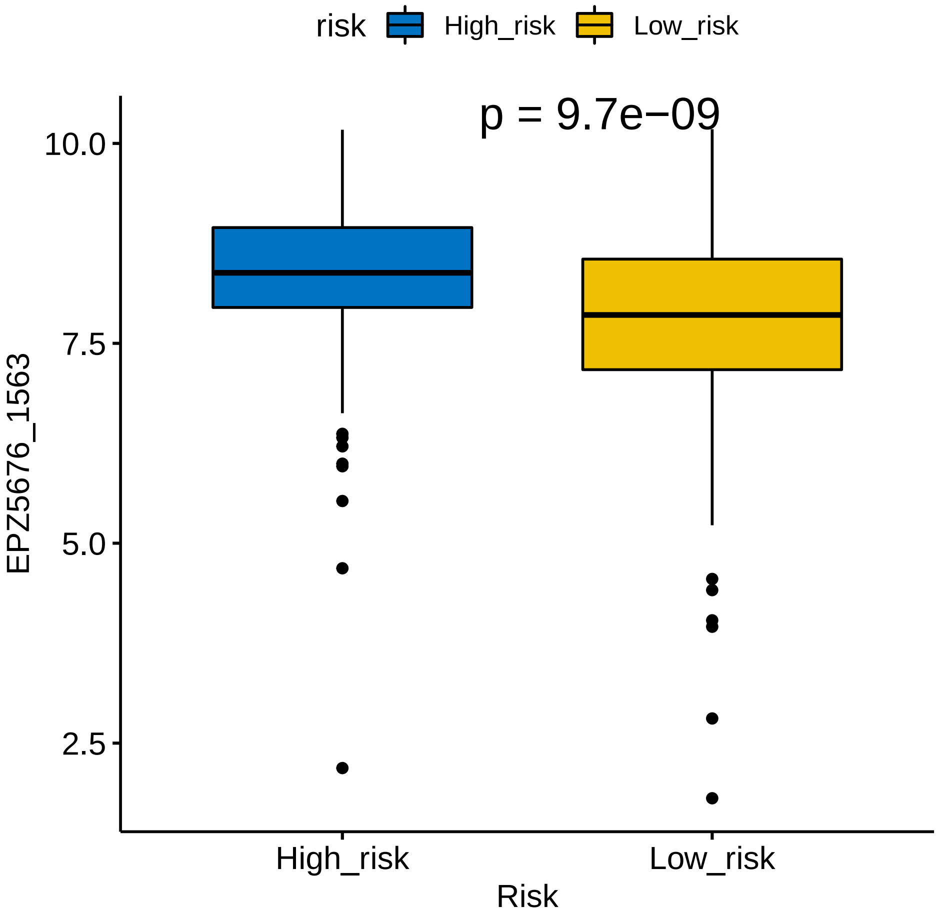

risk
High_risk
Low_risk
p = 9.7e−09
10.0
7.5
5.0
2.5
EPZ5676_1563
High_risk
Low_risk
Risk

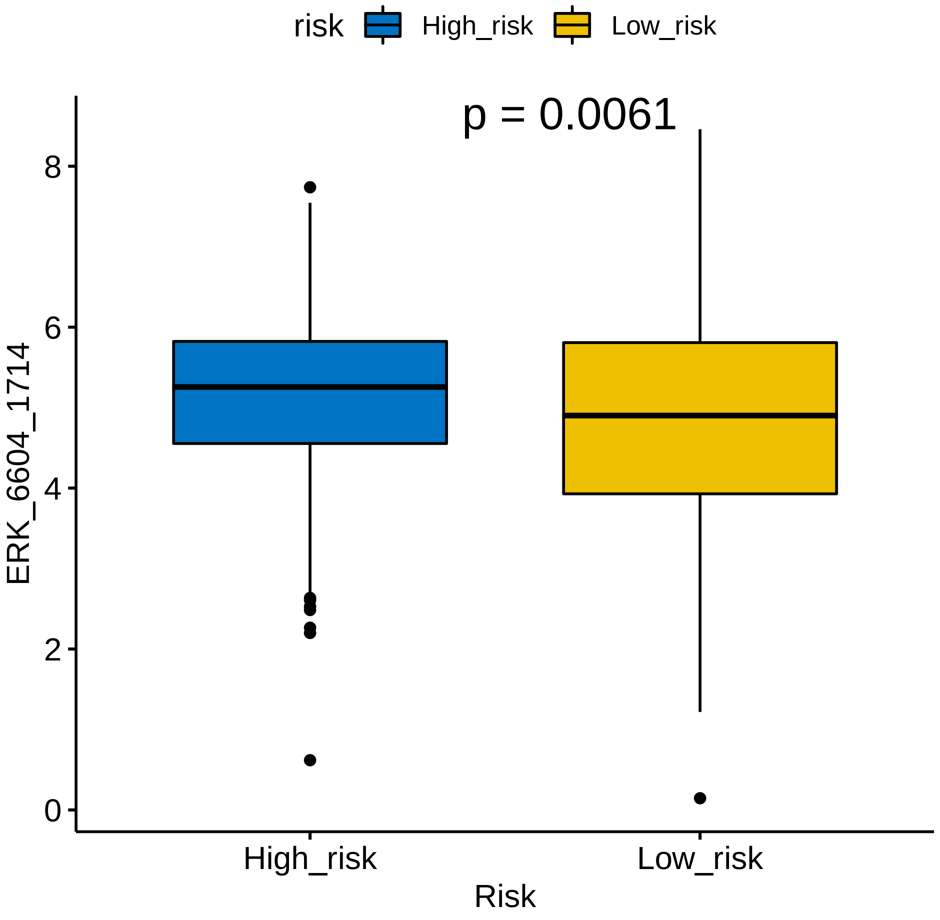

risk
High_risk
Low_risk
p = 0.0061
ERK_6604_1714
8
6
4
2
0
High_risk
Low_risk
Risk

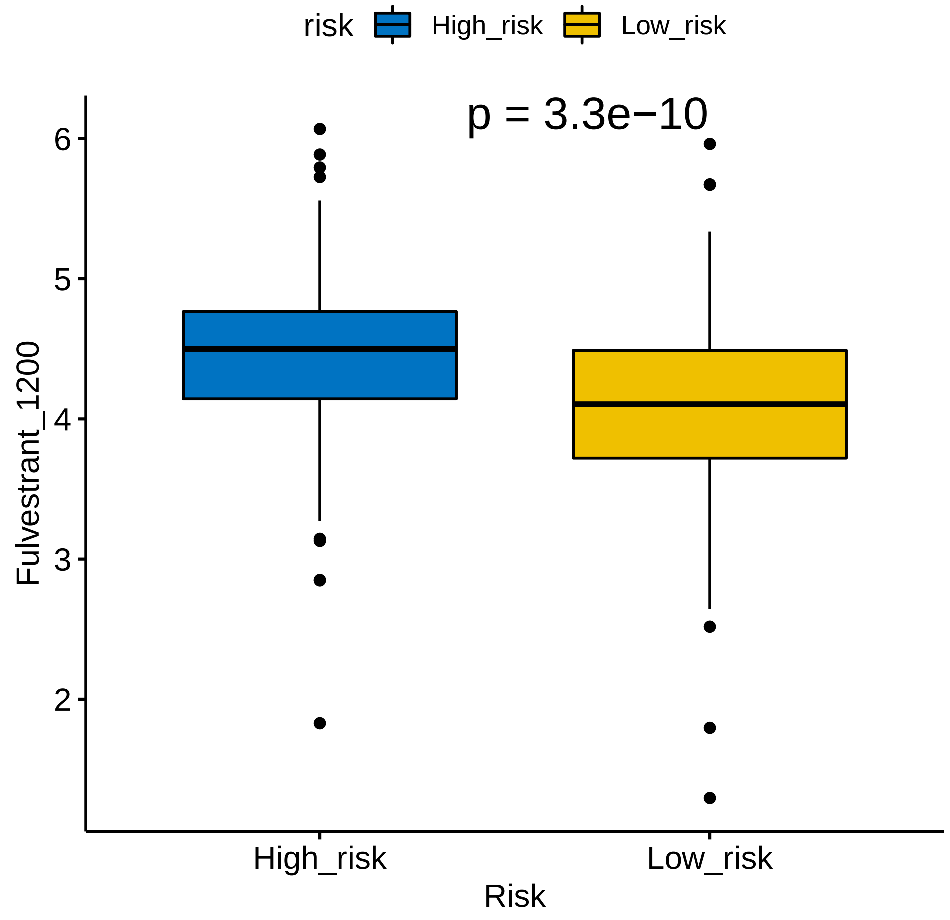

risk
High_risk
Low_risk
p = 3.3e−10
Fulvestrant_1200
6
5
4
3
2
High_risk
Low_risk
Risk

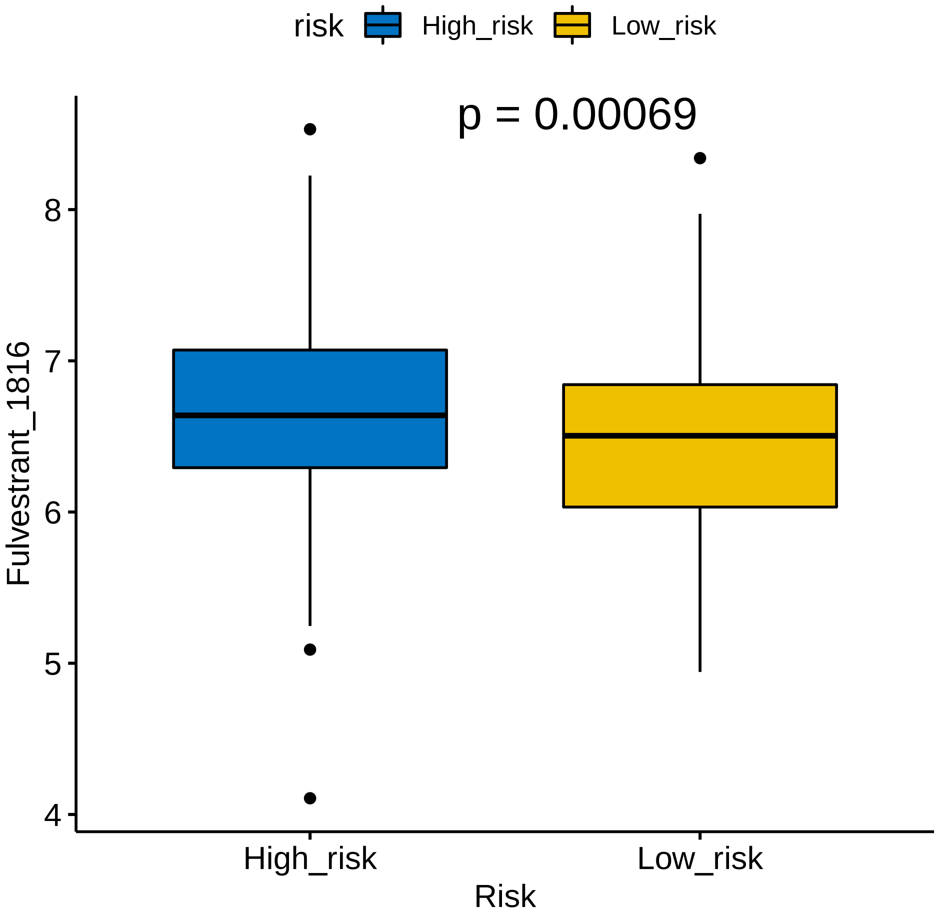

risk
High_risk
Low_risk
p = 0.00069
Fulvestrant_1816
8
7
6
5
4
High_risk
Low_risk
Risk

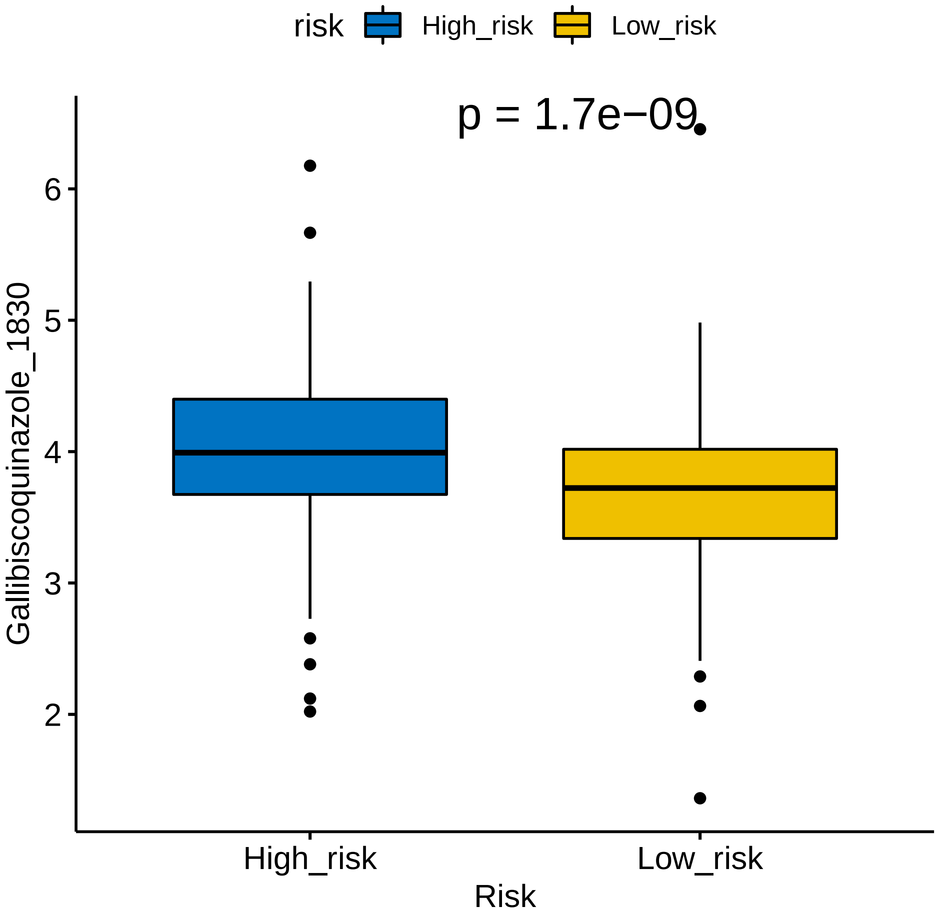

risk
High_risk
Low_risk
p = 1.7e−09
Gallibiscoquinazole_1830
6
5
4
3
2
High_risk
Low_risk
Risk

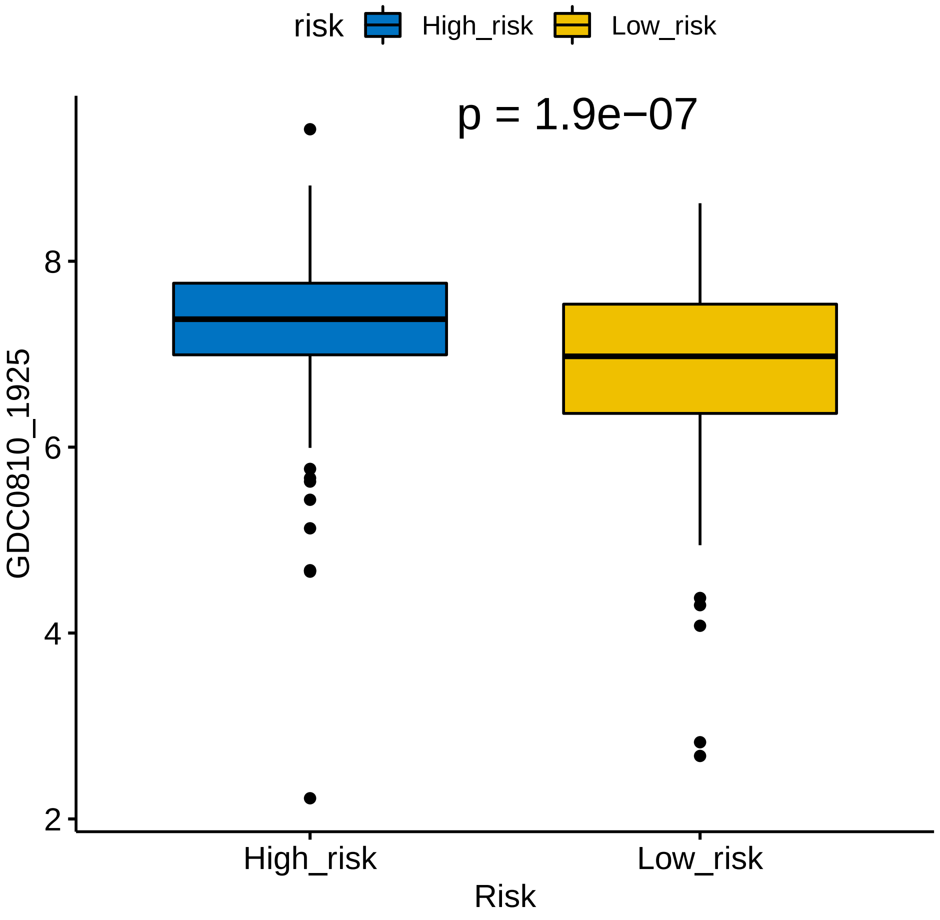

risk
High_risk
Low_risk
p = 1.9e−07
8
6
4
2
GDC0810_1925
High_risk
Low_risk
Risk

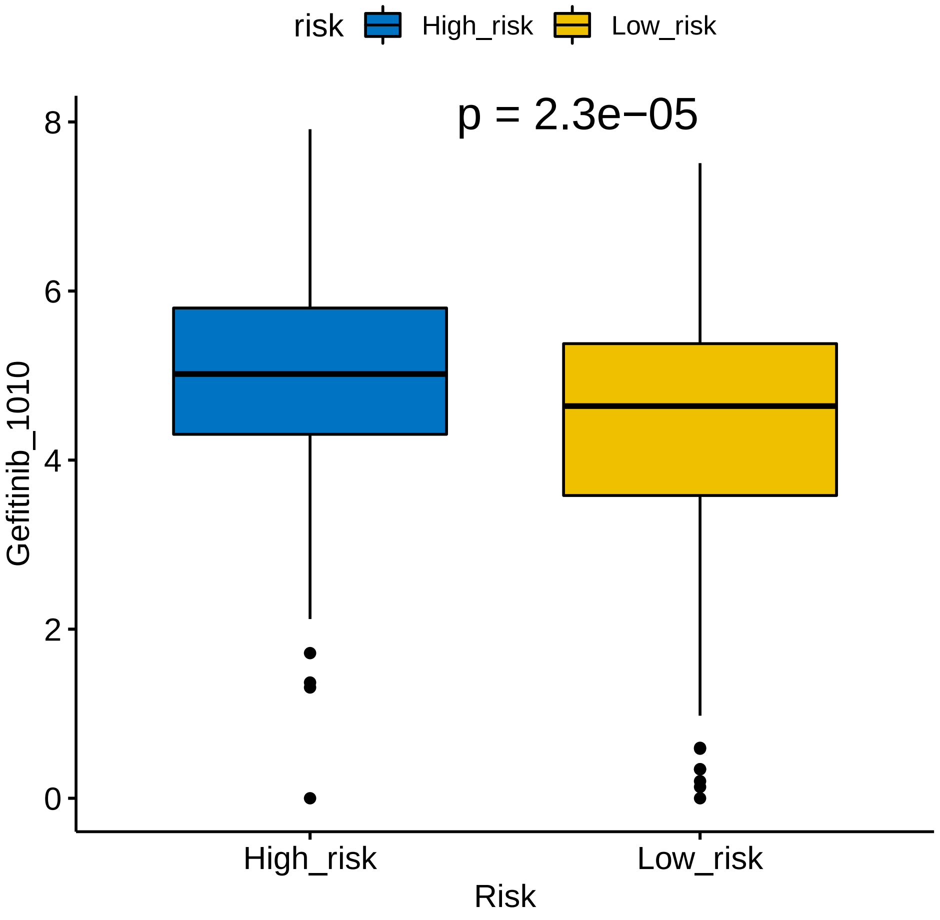

risk
High_risk
Low_risk
p = 2.3e−05
8
6
4
2
0
Gefitinib_1010
High_risk
Low_risk
Risk

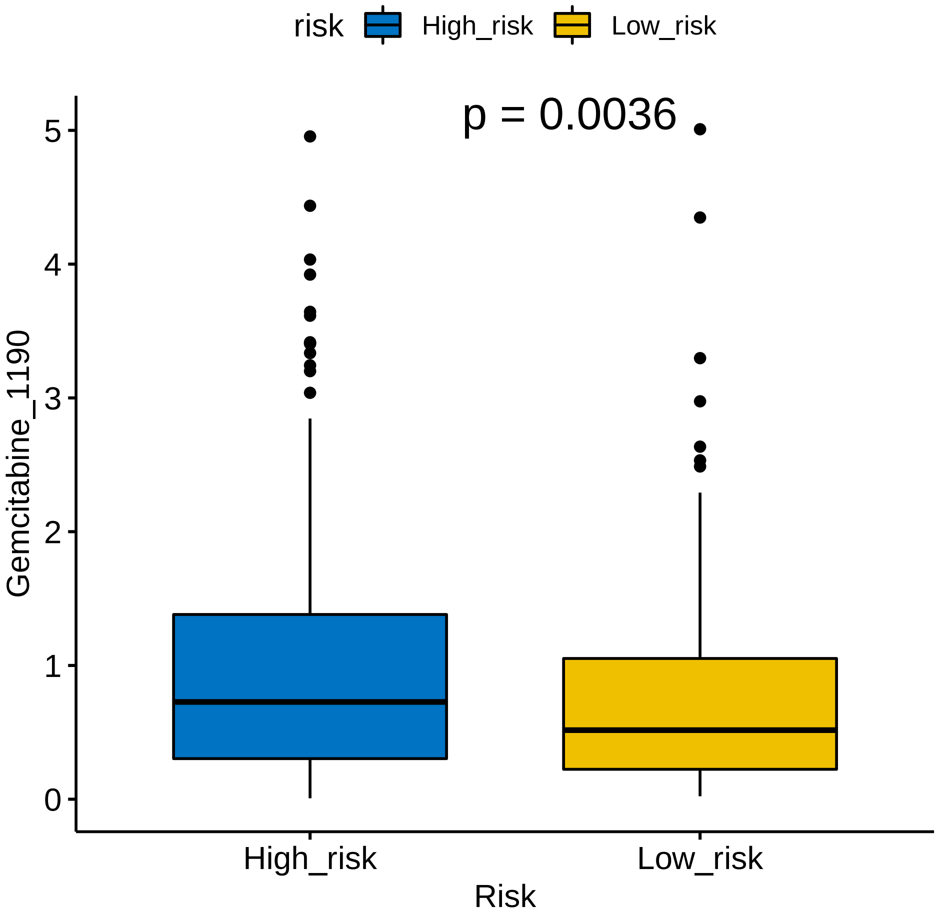
risk
High_risk
Low_risk
p = 0.0036
Gemcitabine_1190
5
4
3
2
1
0
High_risk
Low_risk
Risk

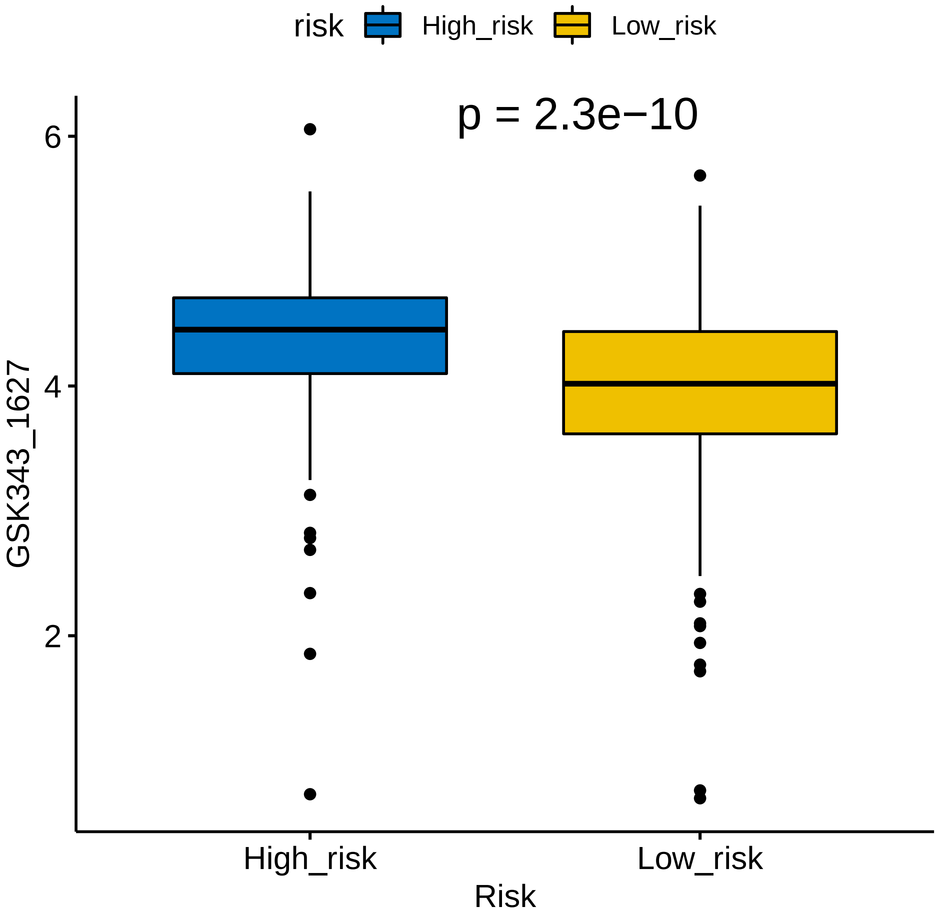

risk
High_risk
Low_risk
p = 2.3e−10
6
4
2
GSK343_1627
High_risk
Low_risk
Risk

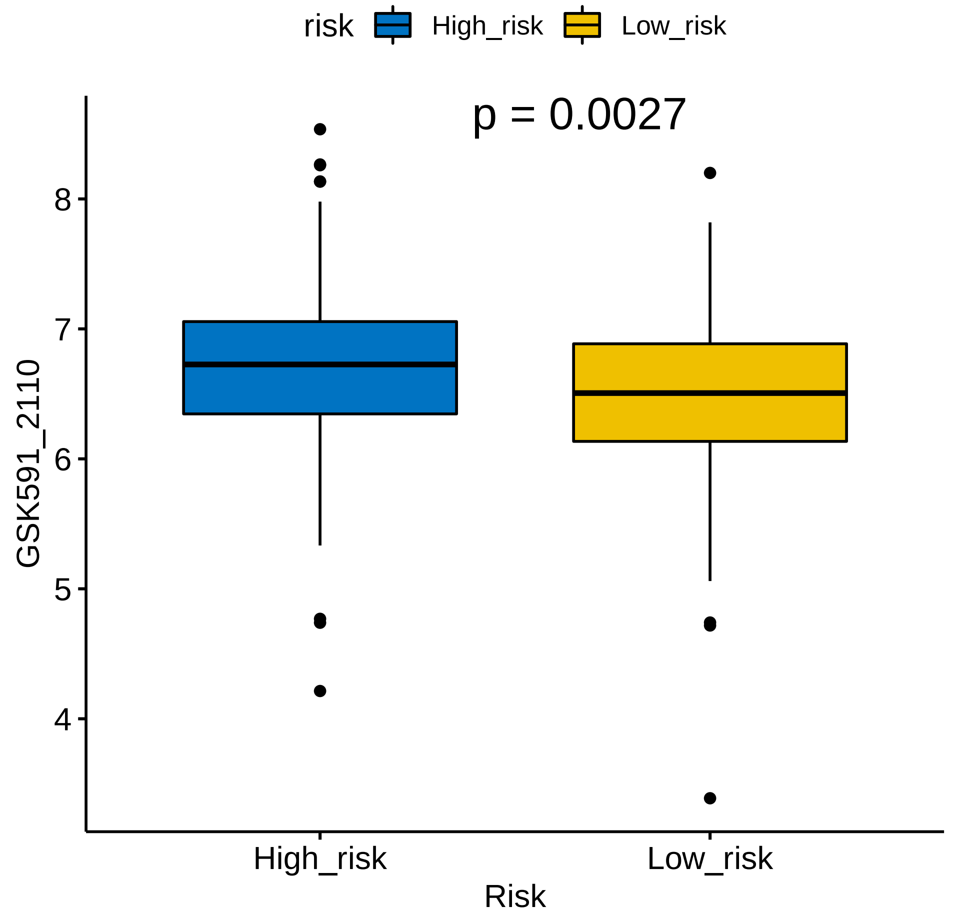
risk
High_risk
Low_risk
p = 0.0027
GSK591_2110
8
7
6
5
4
High_risk
Low_risk
Risk

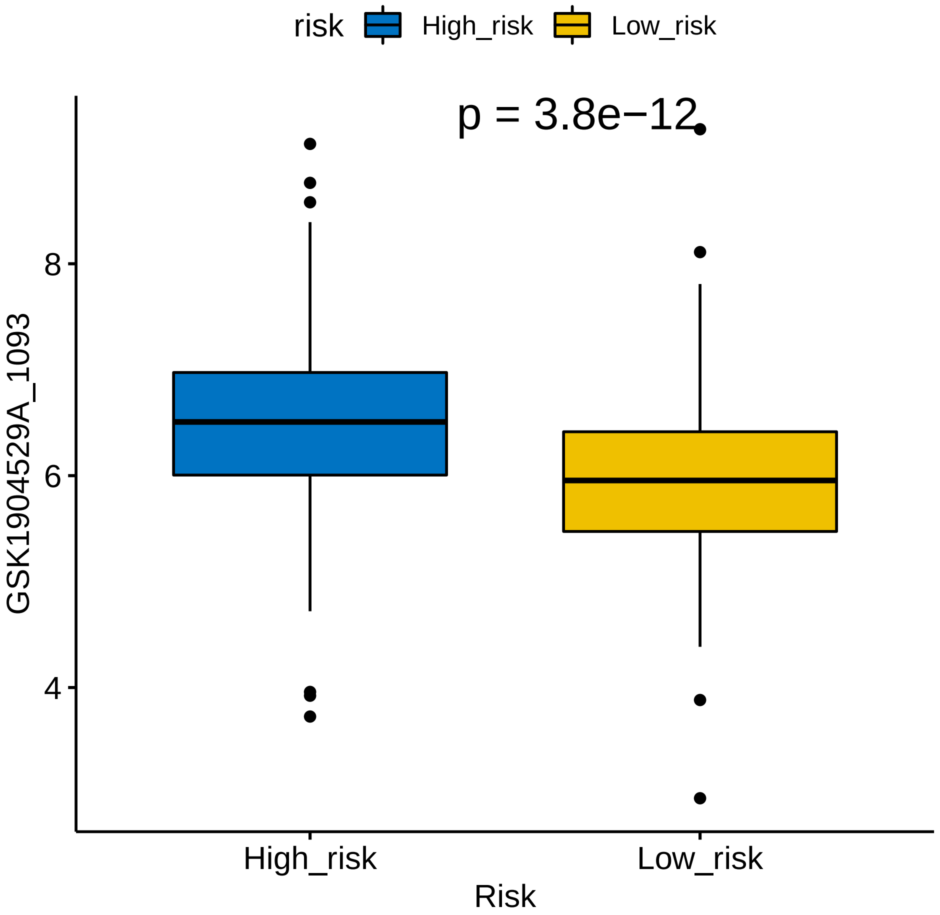

risk
High_risk
Low_risk
p = 3.8e−12
8
6
4
GSK1904529A_1093
High_risk
Low_risk
Risk

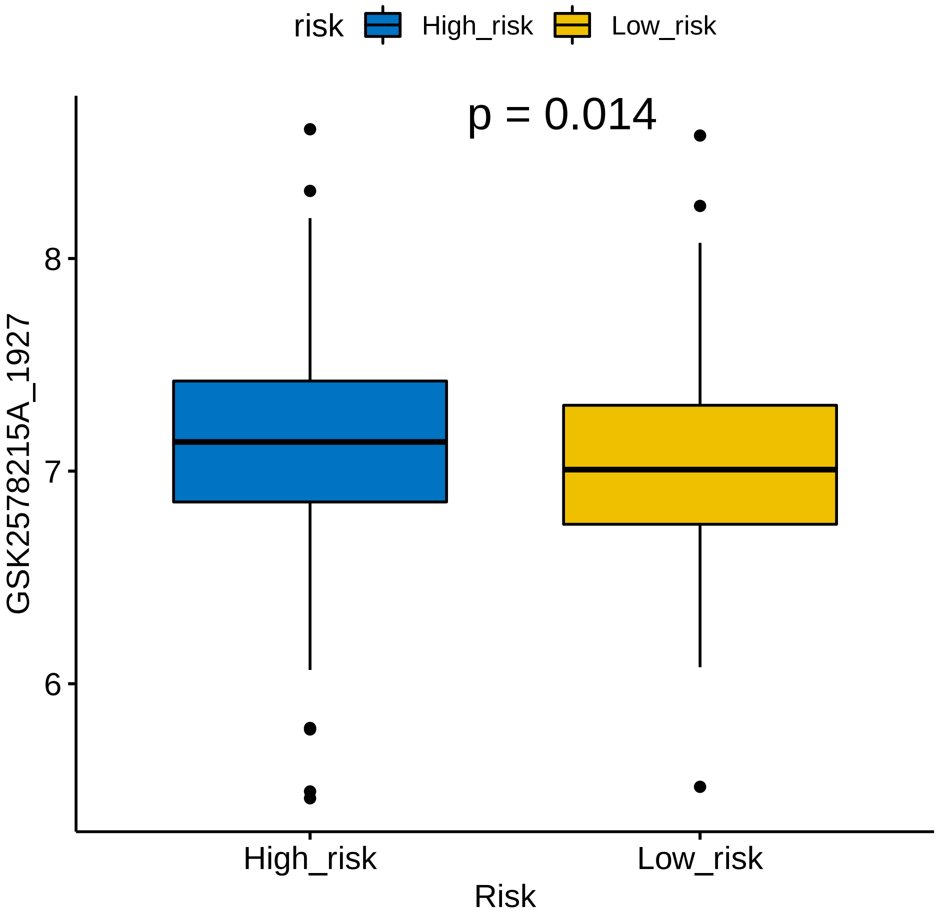

risk
High_risk
Low_risk
p = 0.014
8
7
6
GSK2578215A_1927
High_risk
Low_risk
Risk

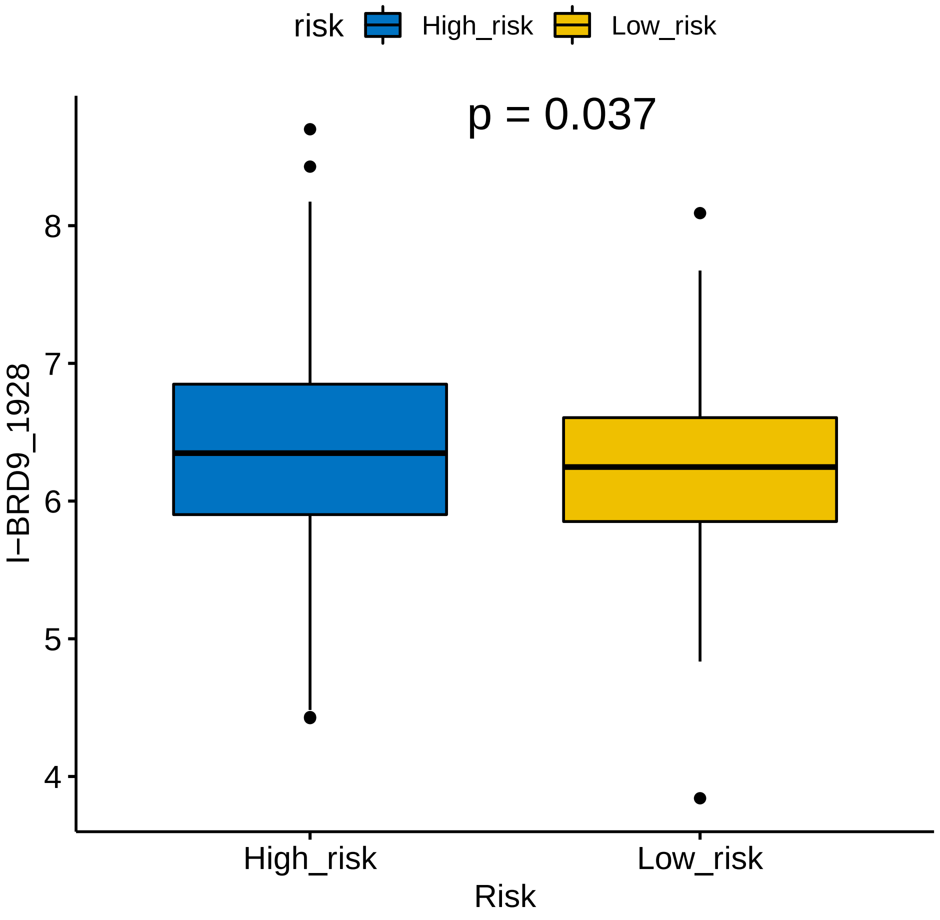

risk
High_risk
Low_risk
p = 0.037
8
7
6
5
4
l−BRD9_1928
High_risk
Low_risk
Risk

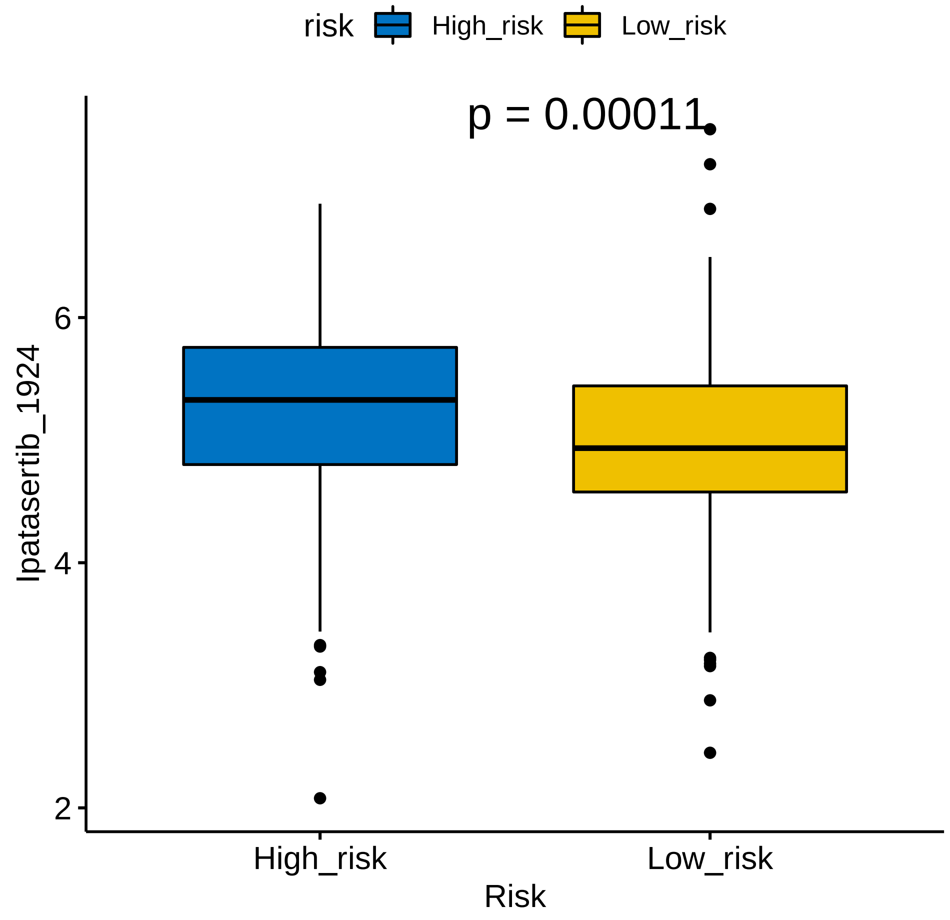
risk
High_risk
Low_risk
p = 0.00011
Ipatasertib_1924
6
4
2
High_risk
Low_risk
Risk

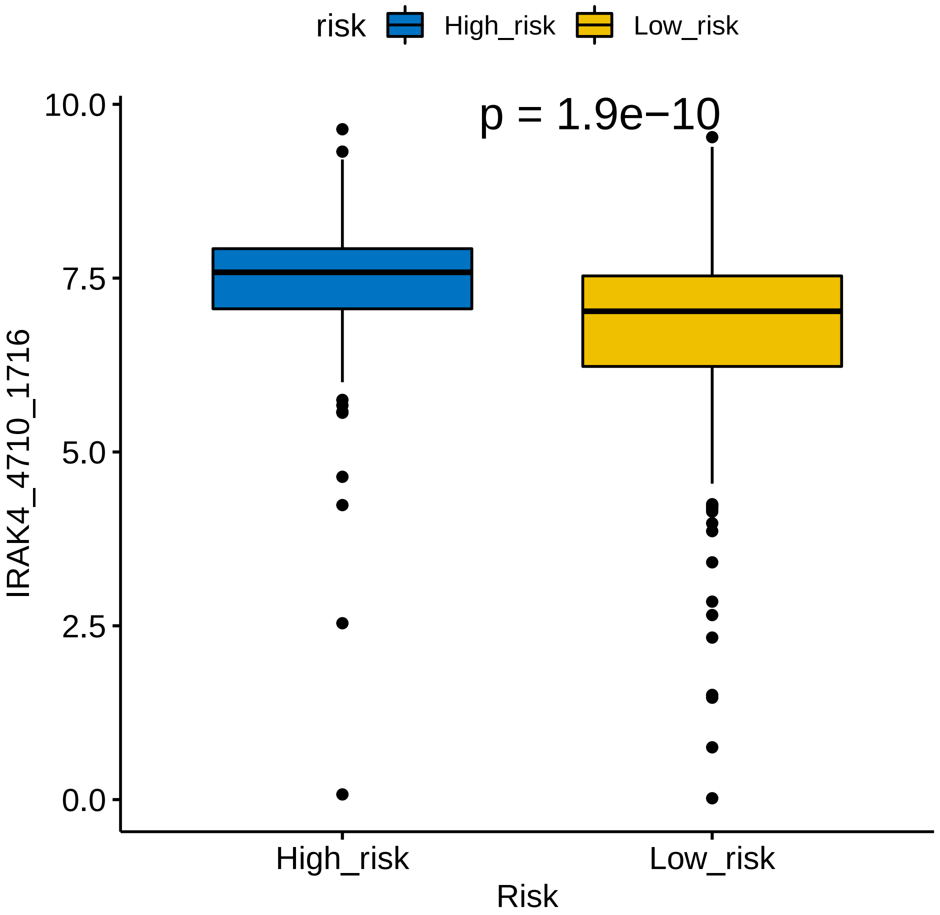

risk
High_risk
Low_risk
p = 1.9e−10
10.0
7.5
5.0
2.5
0.0
IRAK4_4710_1716
High_risk
Low_risk
Risk

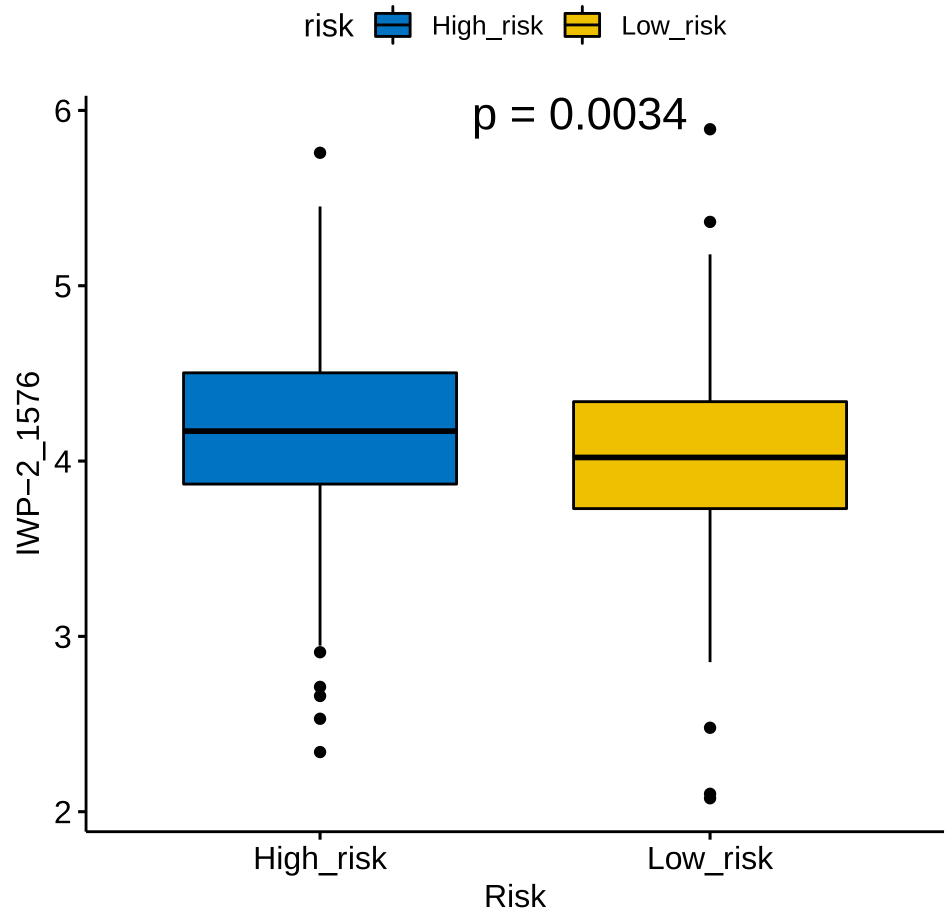

risk
High_risk
Low_risk
p = 0.0034
6
5
4
3
2
IWP−2_1576
High_risk
Low_risk
Risk

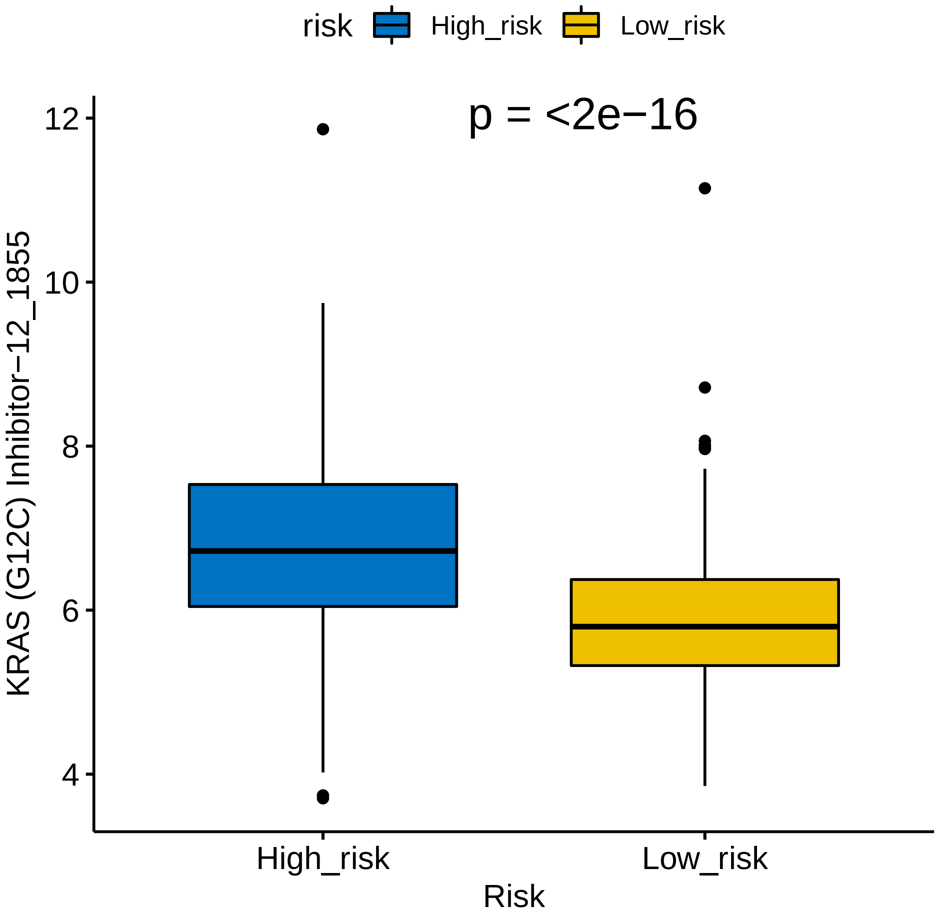

risk
High_risk
Low_risk
p = <2e−16
12
10
8
6
4
KRAS (G12C) Inhibitor−12_1855
High_risk
Low_risk
Risk

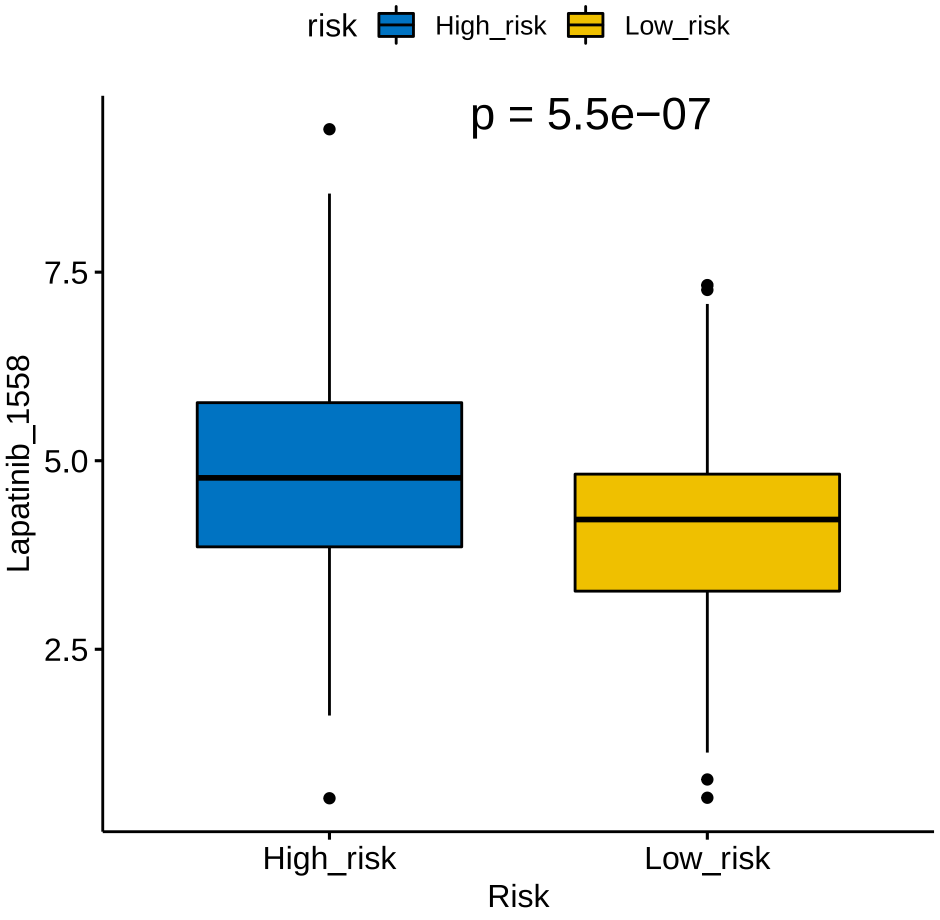
risk
High_risk
Low_risk
p = 5.5e−07
7.5
5.0
2.5
Lapatinib_1558
High_risk
Low_risk
Risk

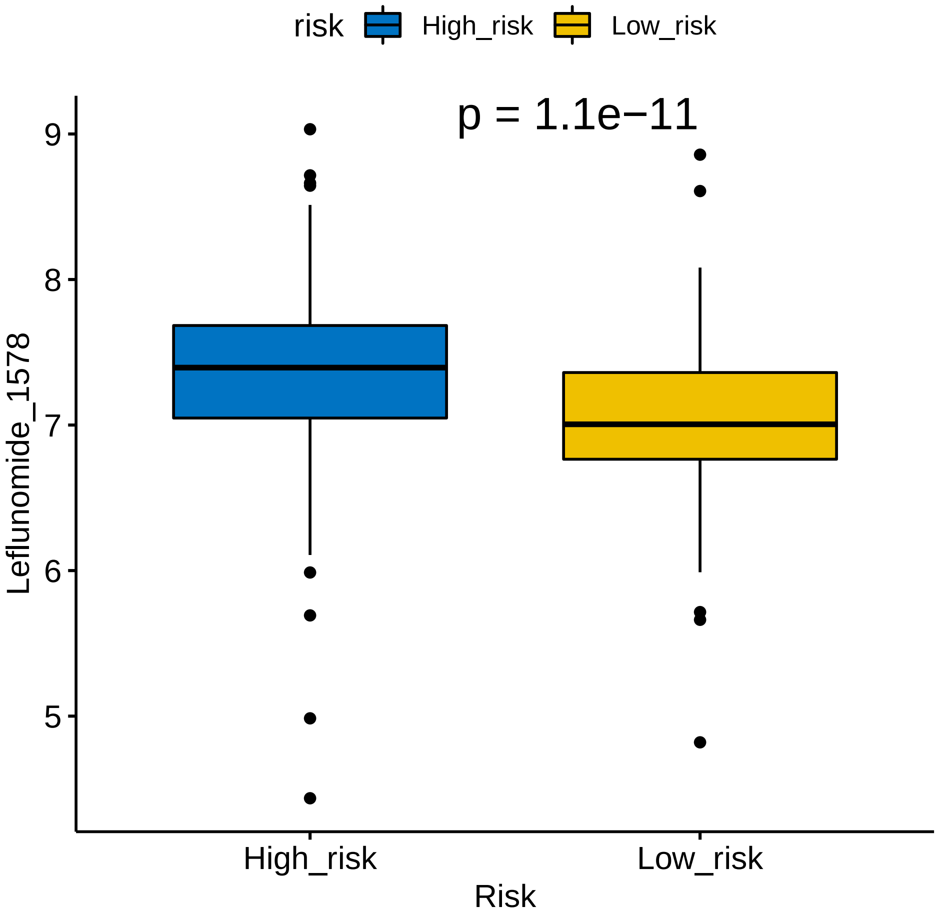
risk
High_risk
Low_risk
p = 1.1e−11
Leflunomide_1578
9
8
7
6
5
High_risk
Low_risk
Risk

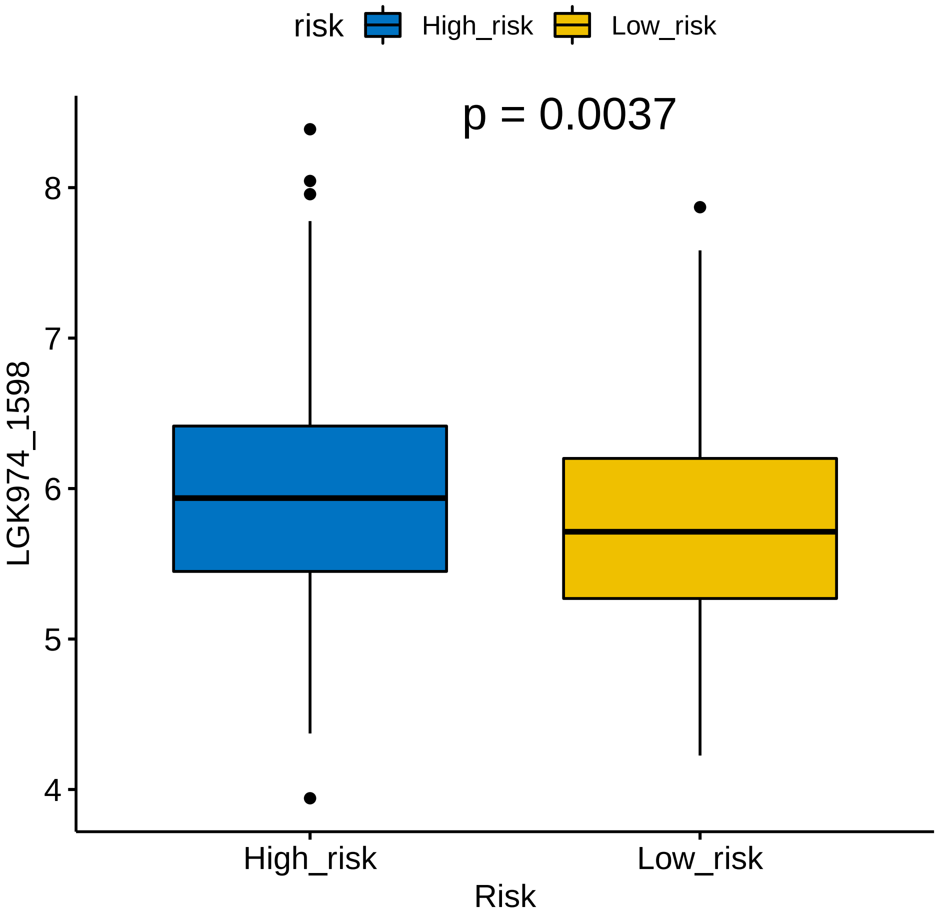

risk
High_risk
Low_risk
p = 0.0037
LGK974_1598
8
7
6
5
4
High_risk
Low_risk
Risk

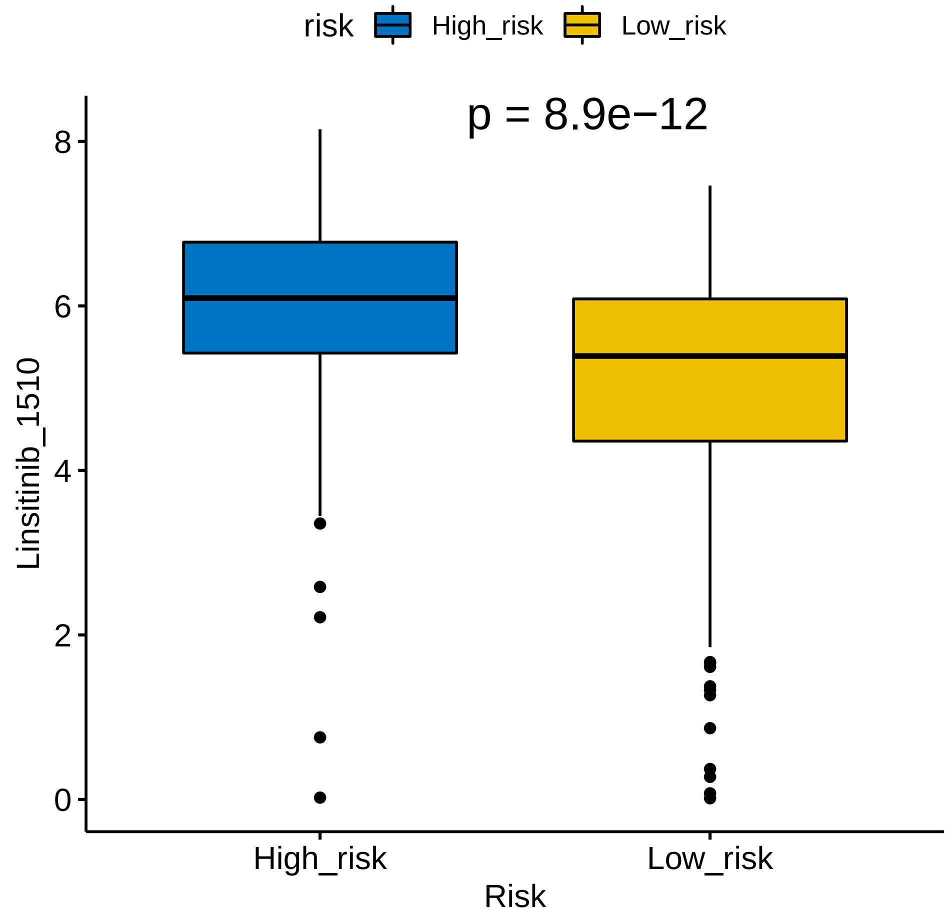

risk
High_risk
Low_risk
p = 8.9e−12
8
6
4
2
0
Linsitinib_1510
High_risk
Low_risk
Risk

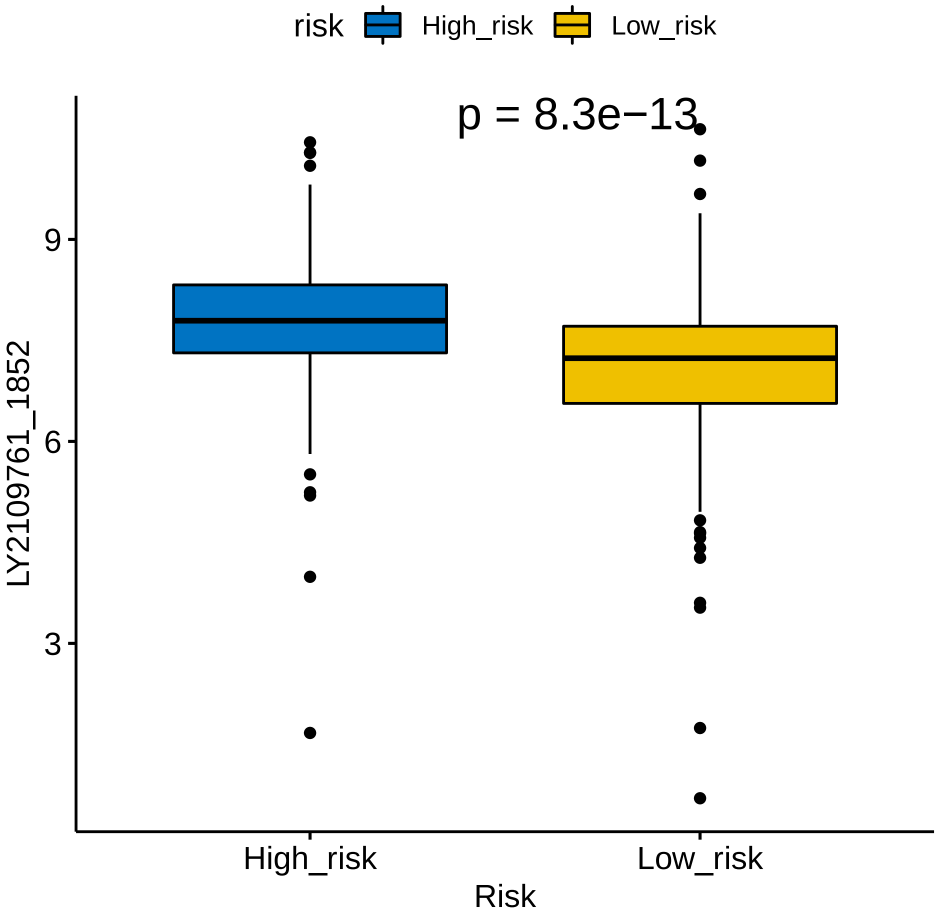

risk
High_risk
Low_risk
p = 8.3e−13
LY2109761_1852
9
6
3
High_risk
Low_risk
Risk

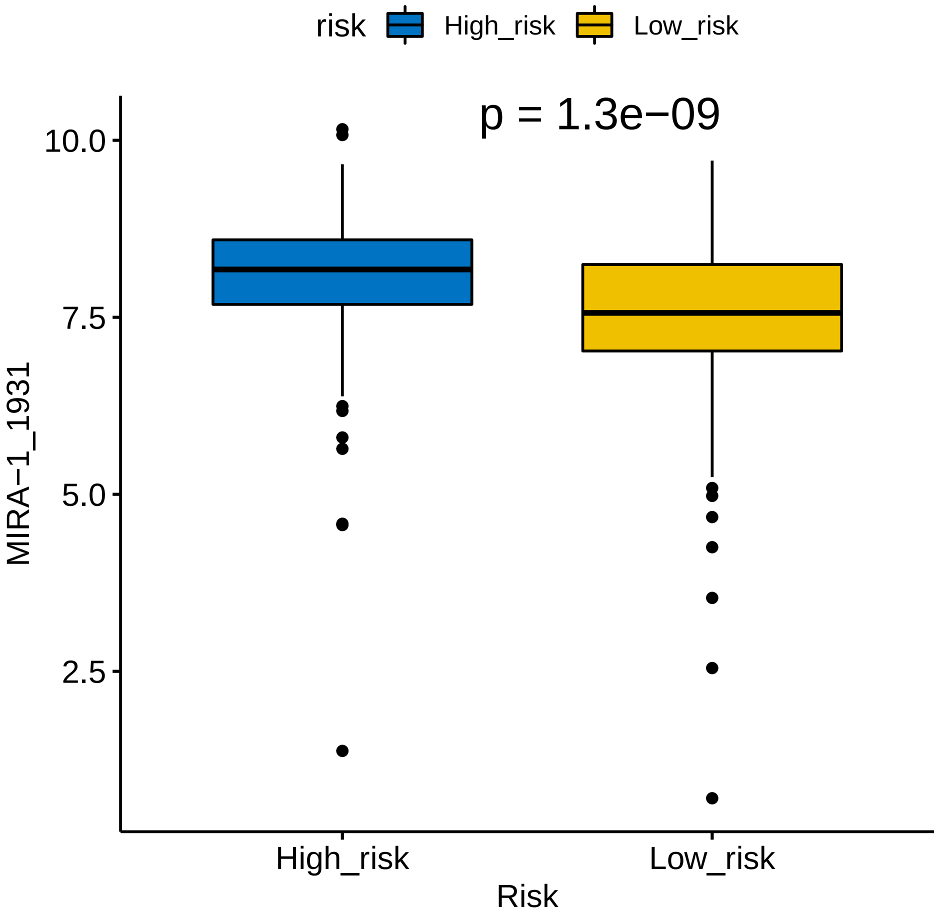
risk
High_risk
Low_risk
p = 1.3e−09
10.0
7.5
5.0
2.5
MIRA−1_1931
High_risk
Low_risk
Risk

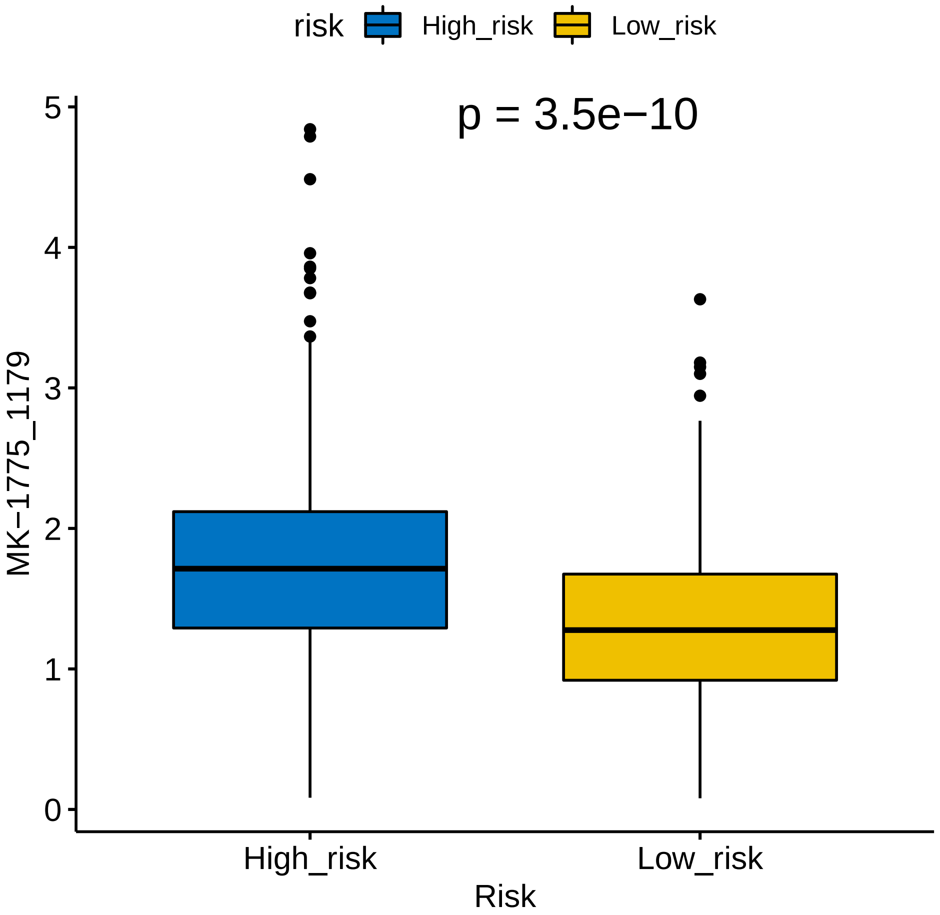
risk
High_risk
Low_risk
p = 3.5e−10
MK−1775_1179
0
1
2
3
4
5
High_risk
Low_risk
Risk

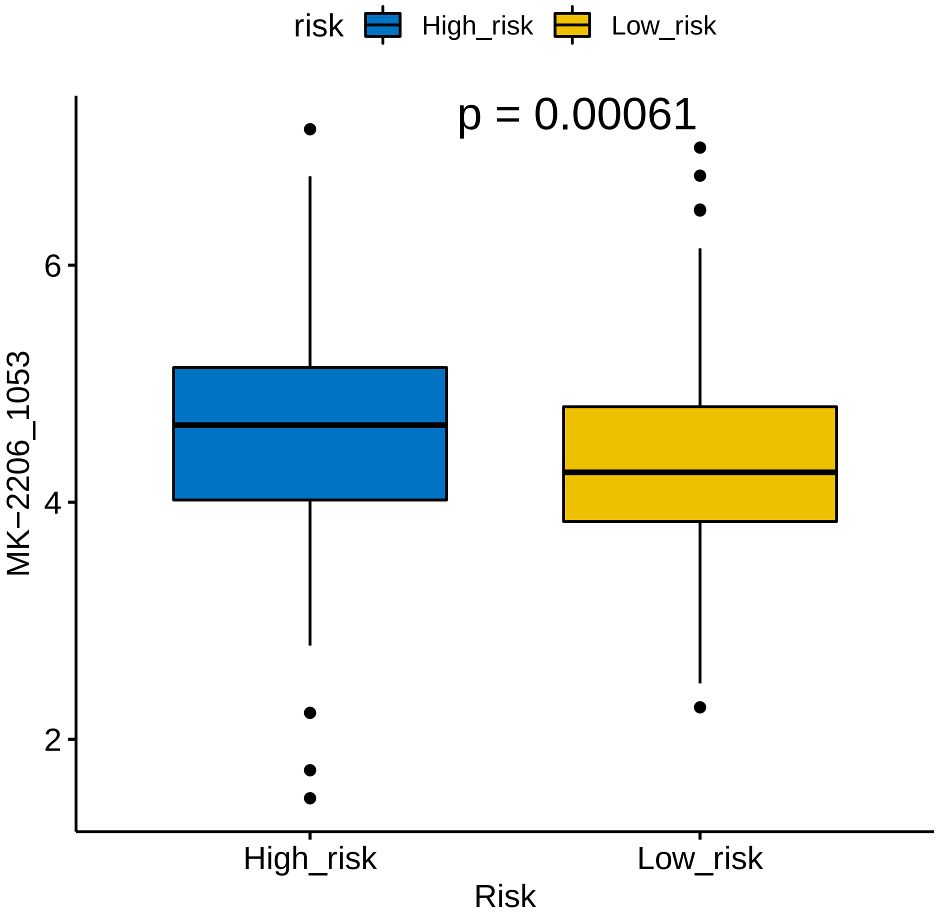
risk
High_risk
Low_risk
p = 0.00061
6
4
2
MK−2206_1053
High_risk
Low_risk
Risk

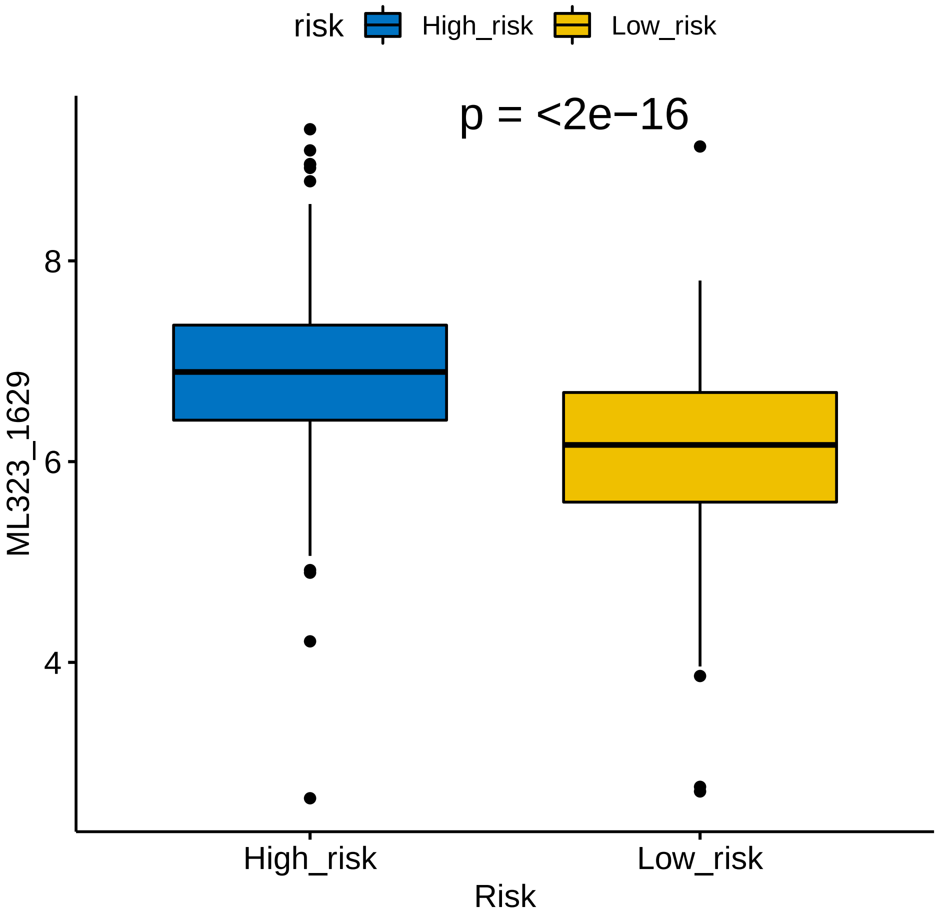

risk
High_risk
Low_risk
p = <2e−16
8
6
4
ML323_1629
High_risk
Low_risk
Risk

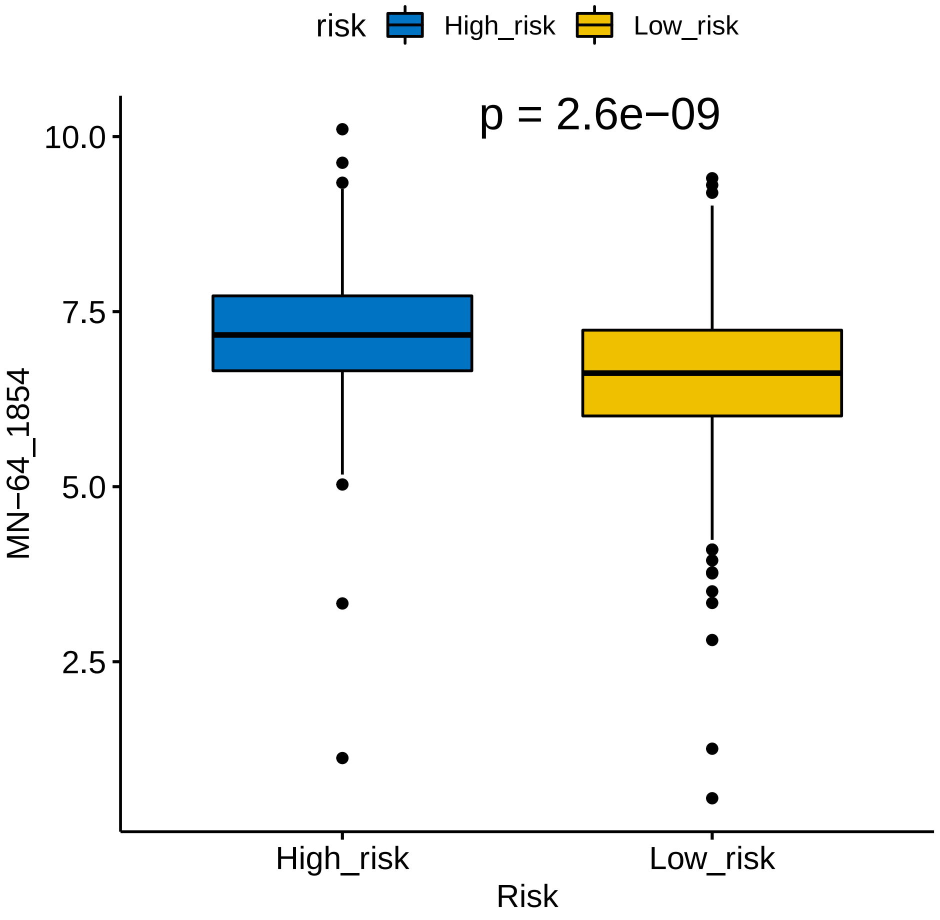

risk
High_risk
Low_risk
p = 2.6e−09
10.0
7.5
5.0
2.5
MN−64_1854
High_risk
Low_risk
Risk

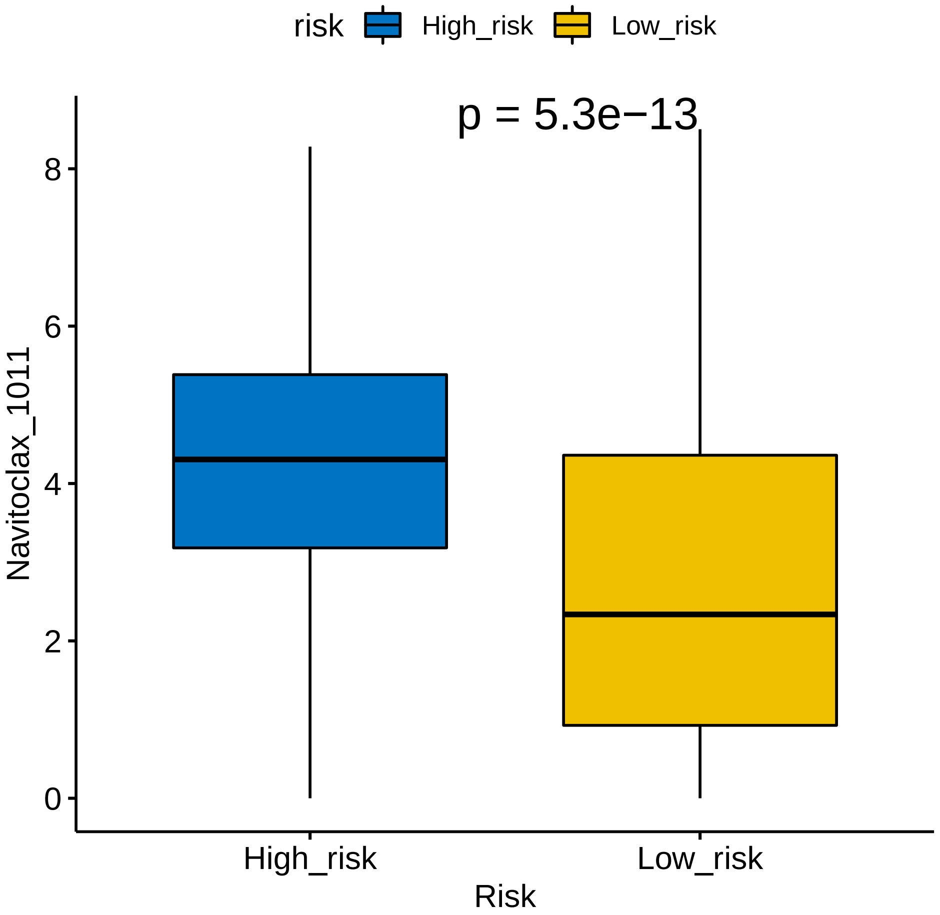
risk
High_risk
Low_risk
p = 5.3e−13
Navitoclax_1011
8
6
4
2
0
High_risk
Low_risk
Risk

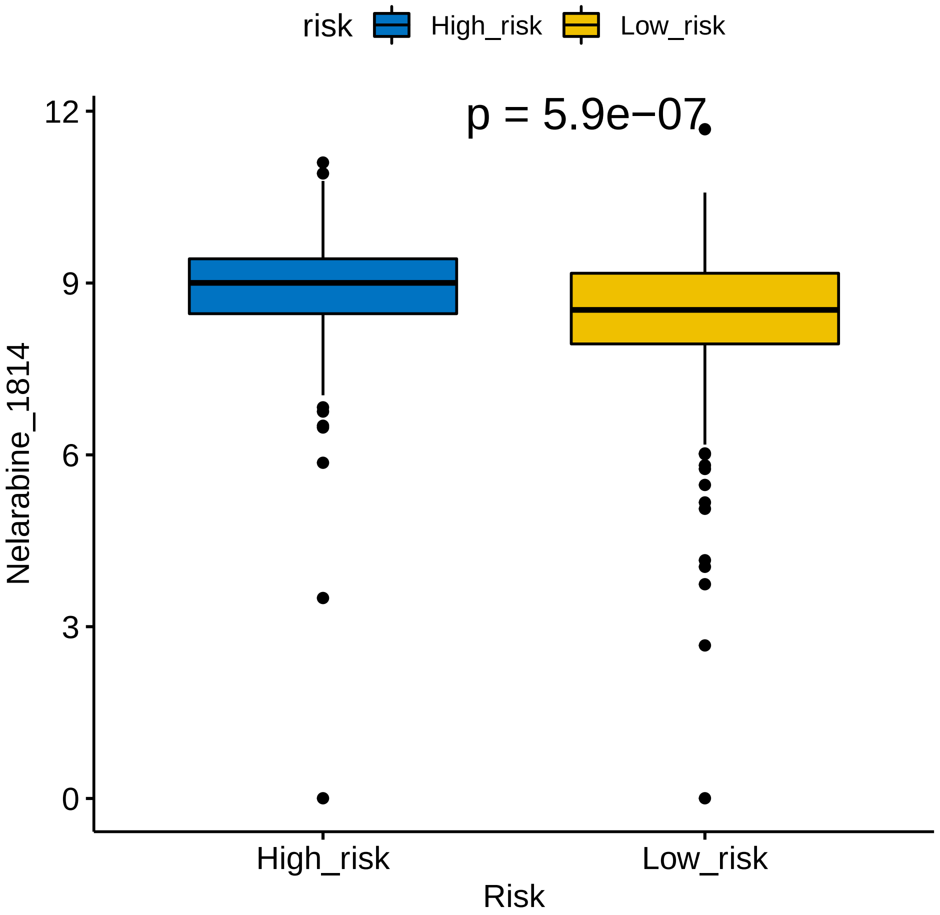

risk
High_risk
Low_risk
p = 5.9e−07
Nelarabine_1814
12
9
6
3
0
High_risk
Low_risk
Risk

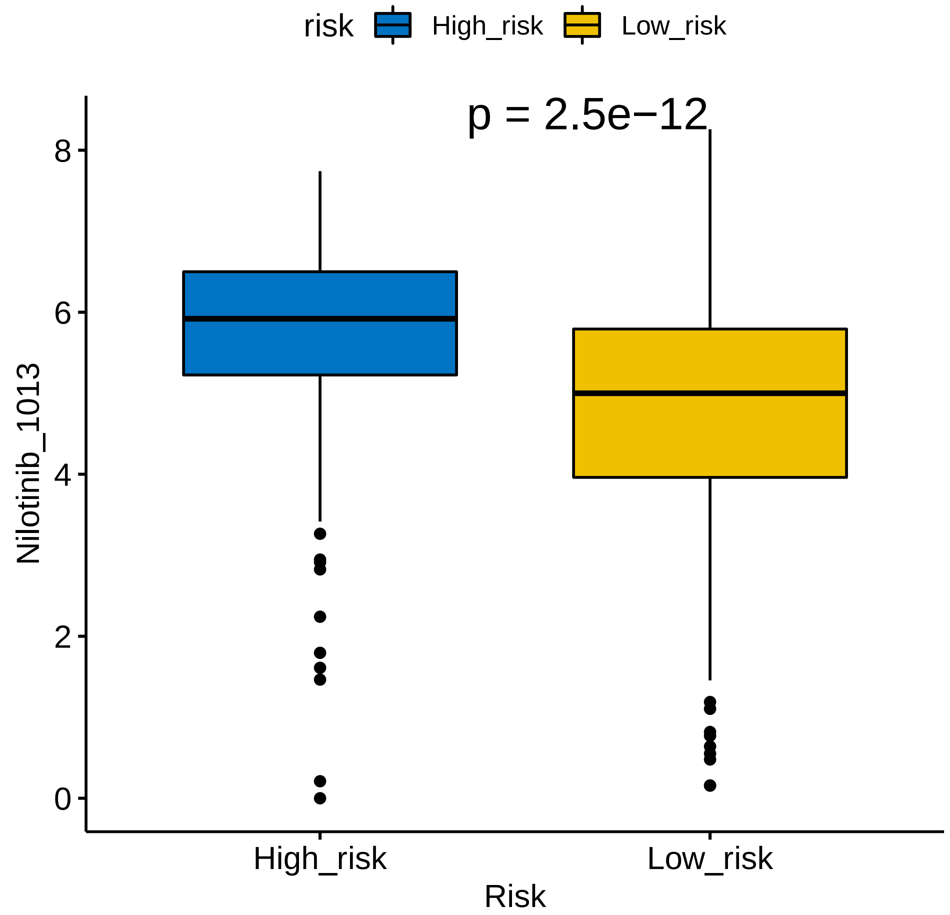

risk
High_risk
Low_risk
p = 2.5e−12
Nilotinib_1013
8
6
4
2
0
High_risk
Low_risk
Risk

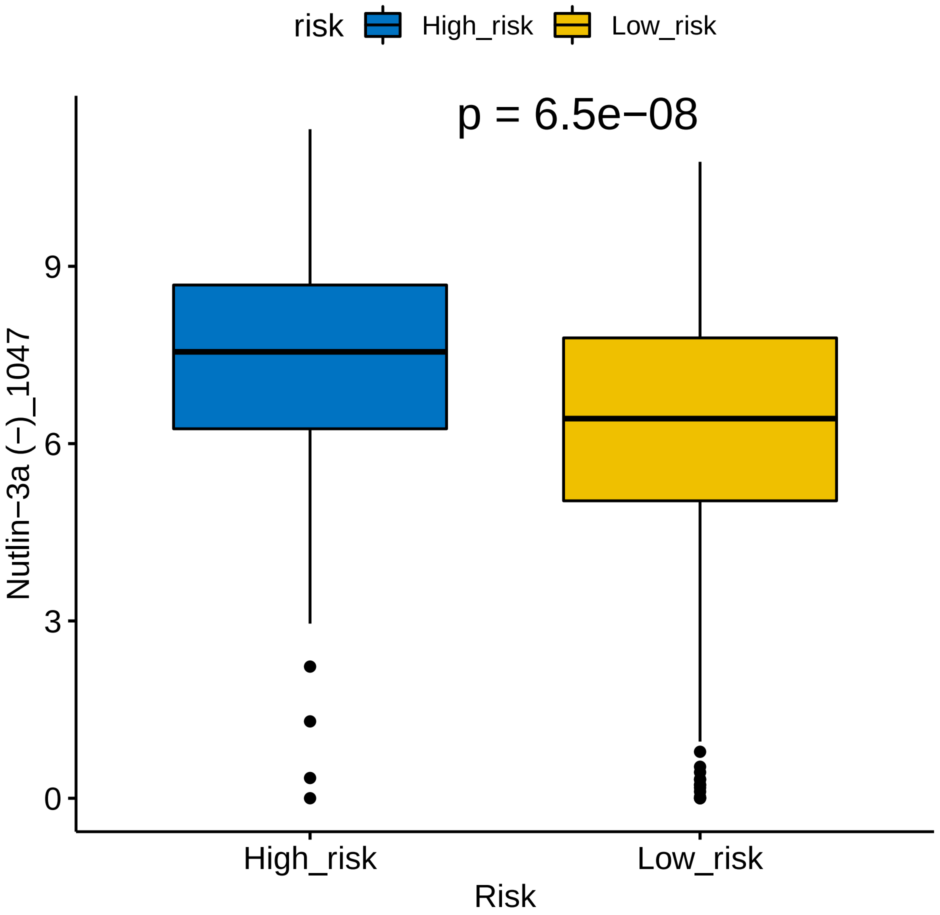
risk
High_risk
Low_risk
p = 6.5e−08
Nutlin−3a (−)_1047
9
6
3
0
High_risk
Low_risk
Risk

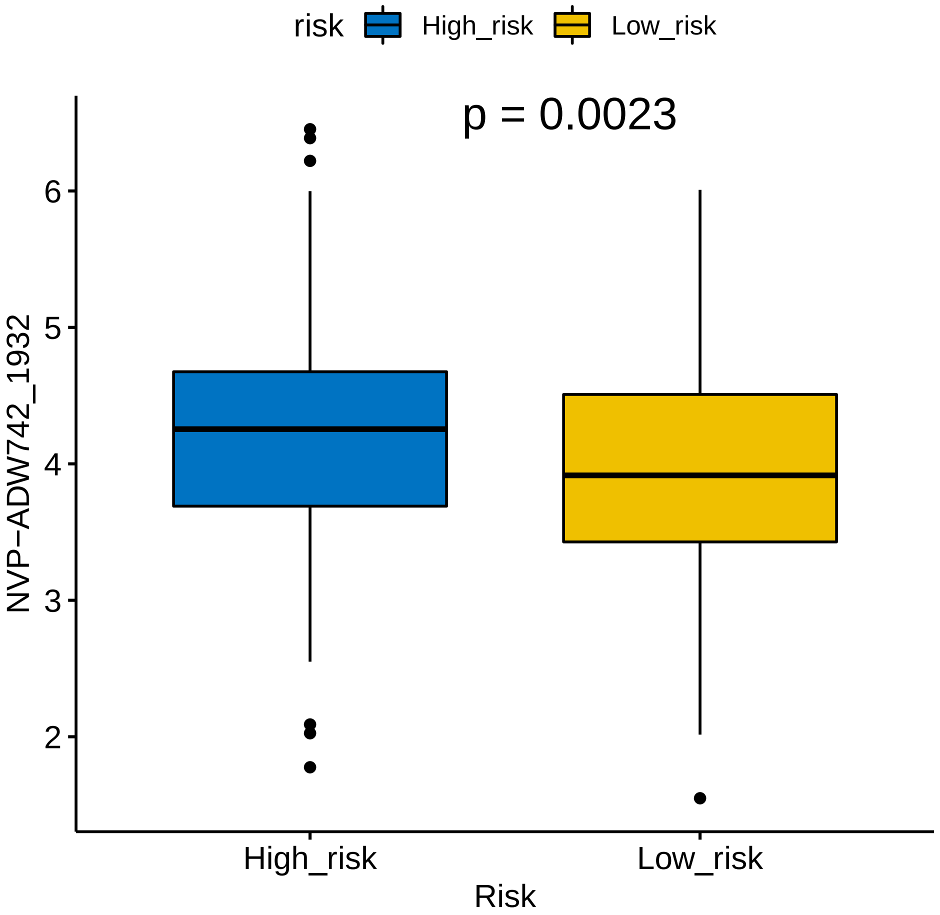
risk
High_risk
Low_risk
p = 0.0023
NVP−ADW742_1932
6
5
4
3
2
High_risk
Low_risk
Risk

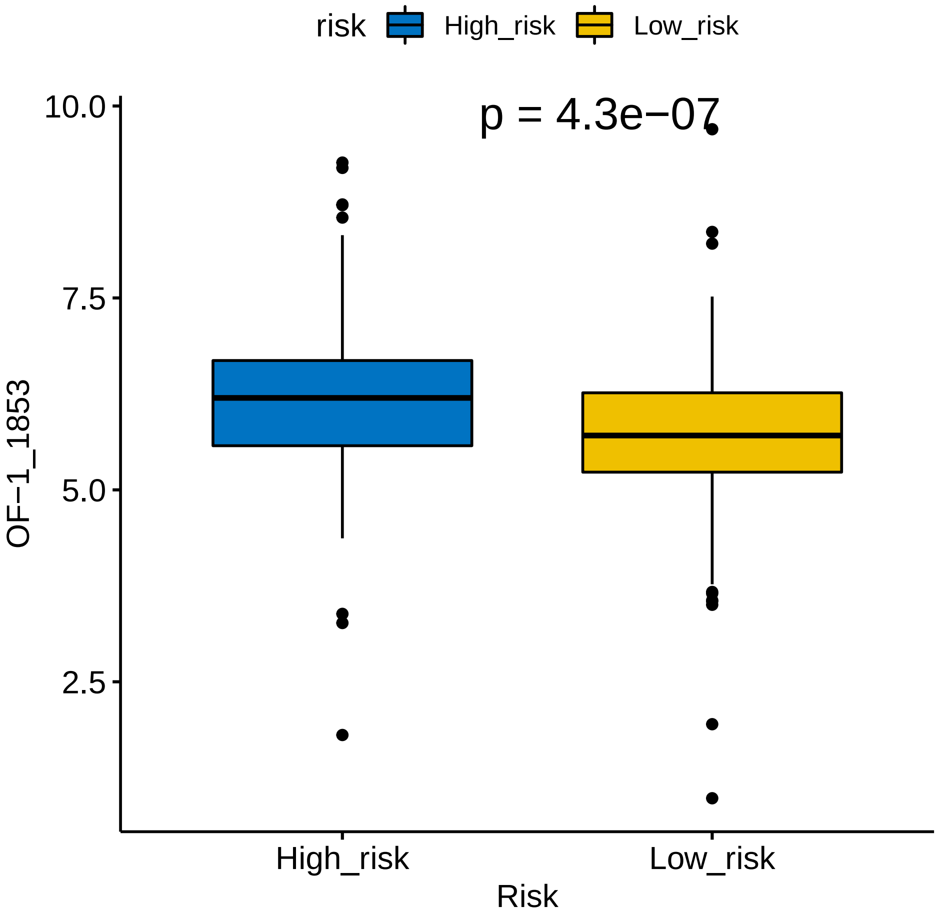

risk
High_risk
Low_risk
p = 4.3e−07
10.0
7.5
5.0
2.5
OF−1_1853
High_risk
Low_risk
Risk

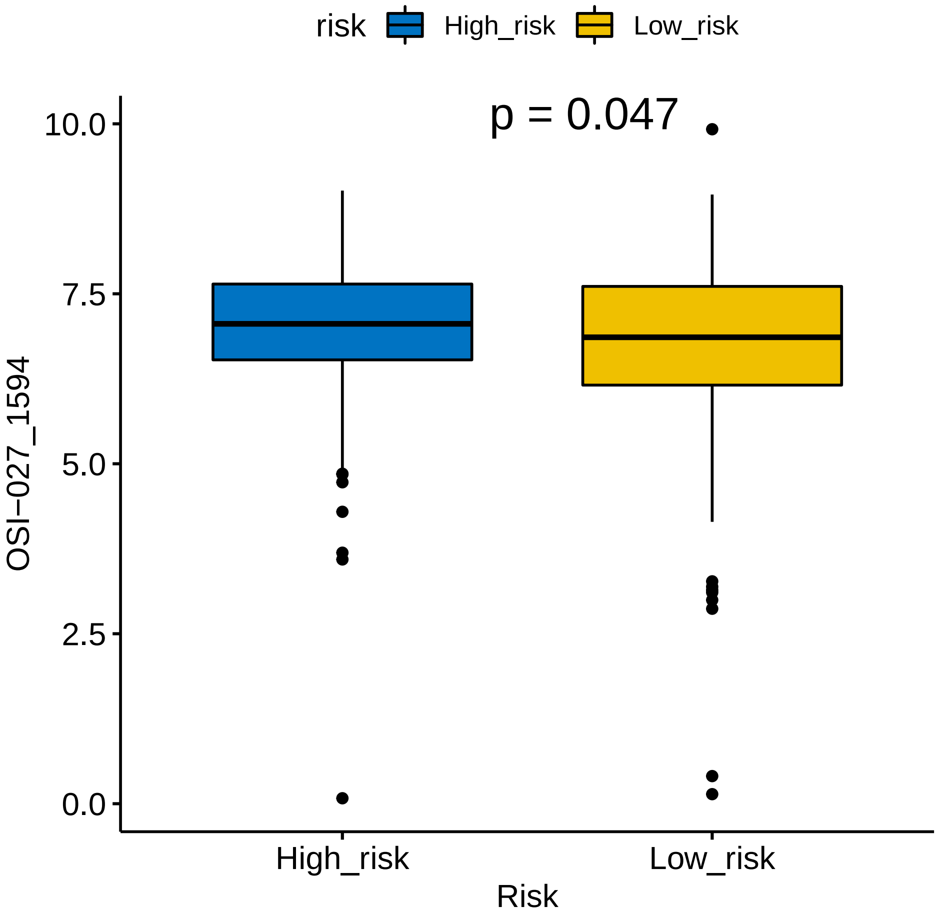

risk
High_risk
Low_risk
p = 0.047
10.0
7.5
5.0
2.5
0.0
OSI−027_1594
High_risk
Low_risk
Risk

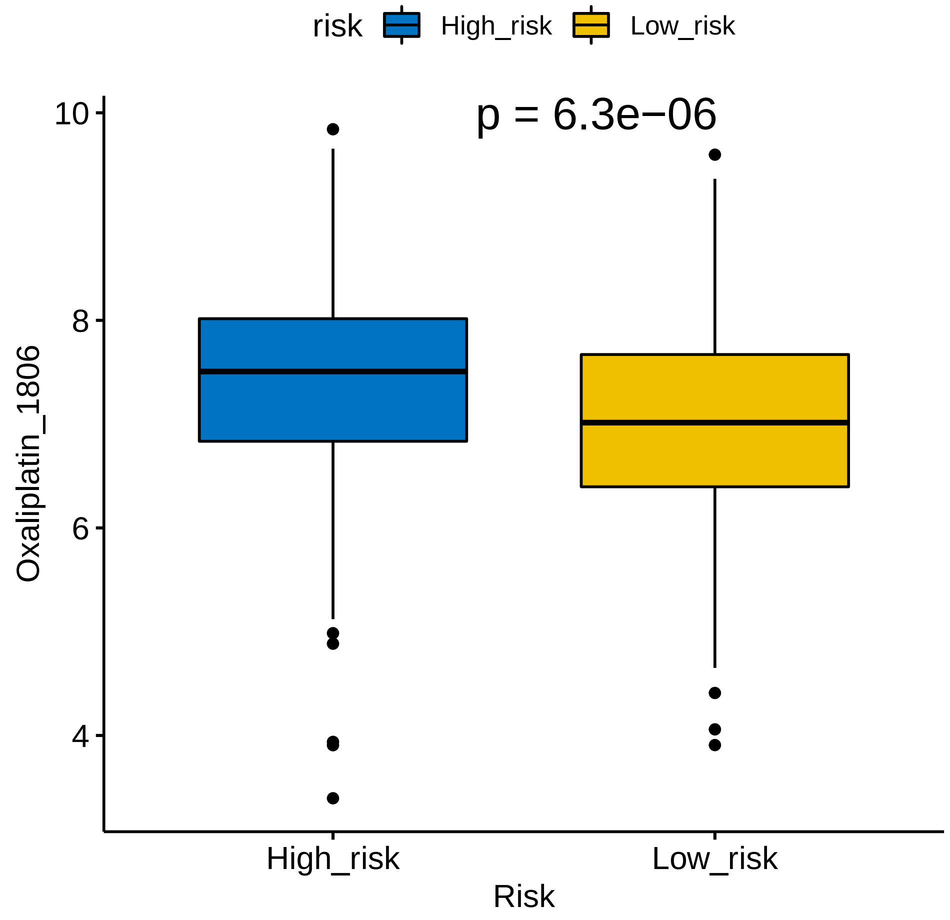

risk
High_risk
Low_risk
p = 6.3e−06
10
8
6
4
Oxaliplatin_1806
High_risk
Low_risk
Risk

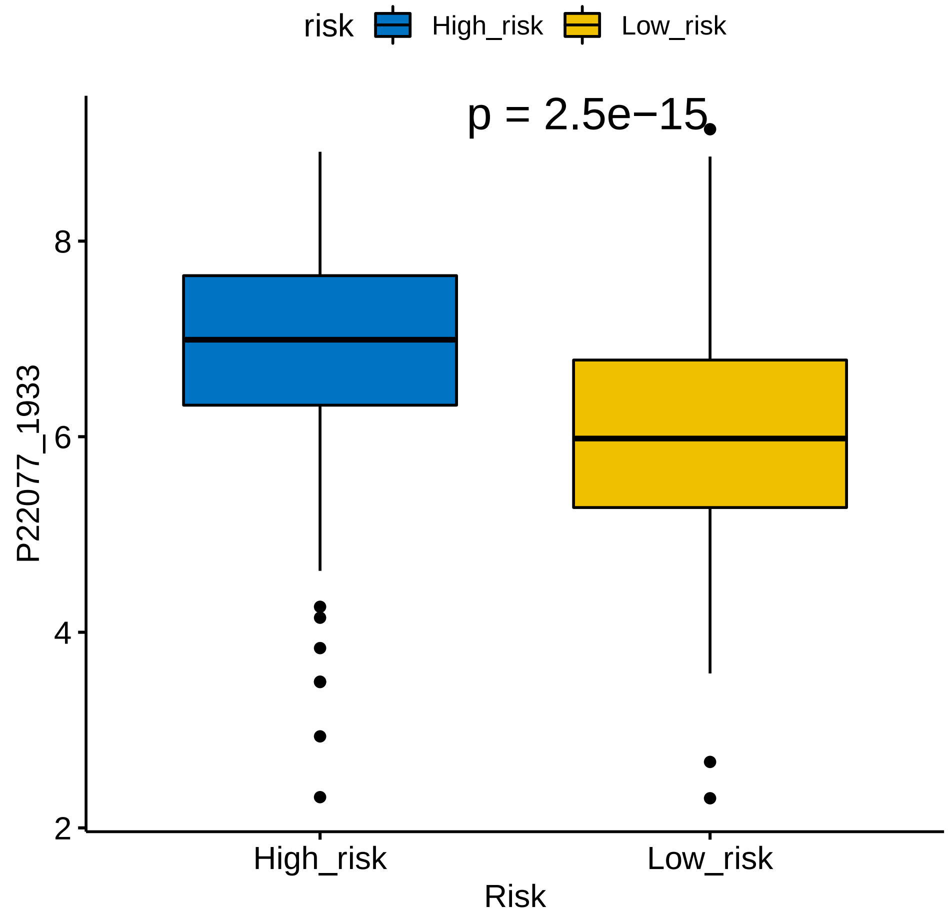

risk
High_risk
Low_risk
p = 2.5e−15
8
6
4
2
P22077_1933
High_risk
Low_risk
Risk

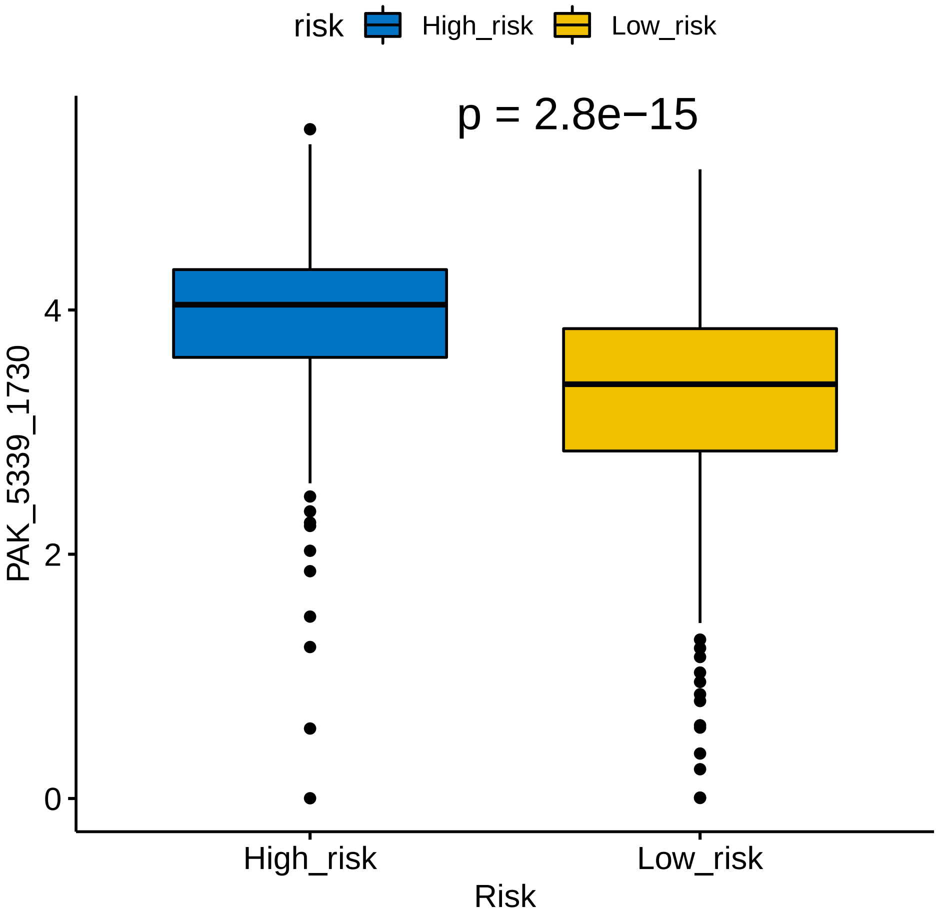

risk
High_risk
Low_risk
p = 2.8e−15
PAK_5339_1730
4
2
0
High_risk
Low_risk
Risk

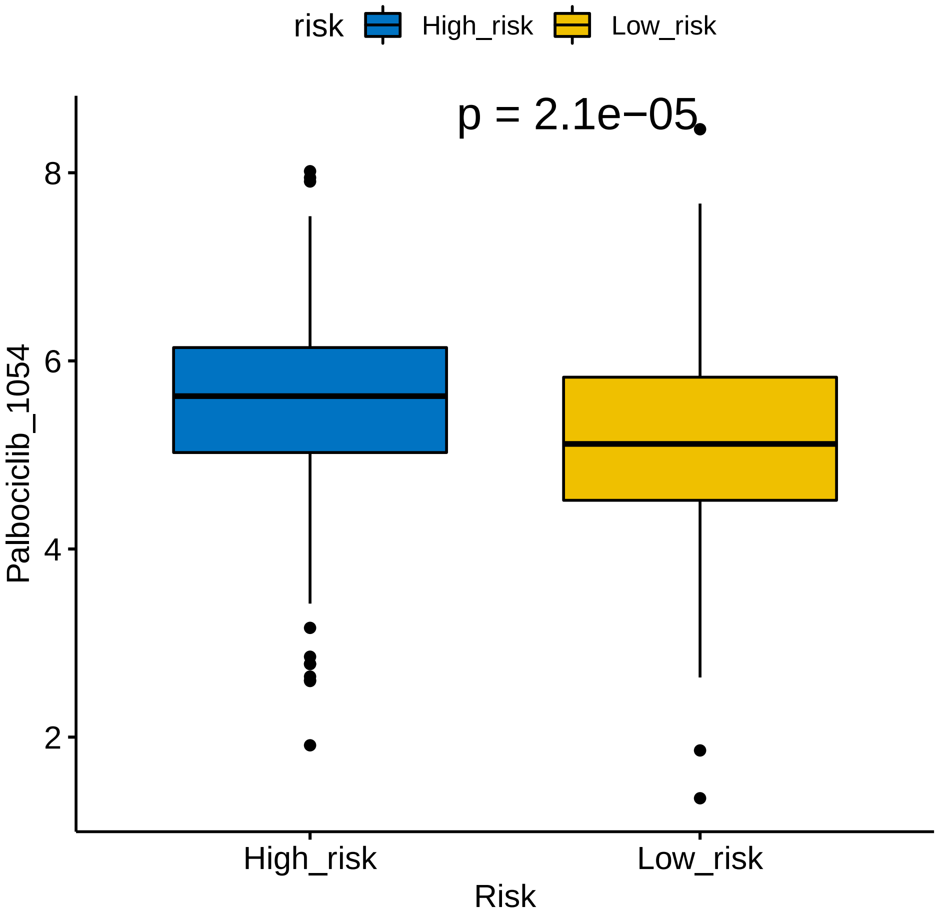

risk
High_risk
Low_risk
p = 2.1e−05
8
6
4
2
Palbociclib_1054
High_risk
Low_risk
Risk

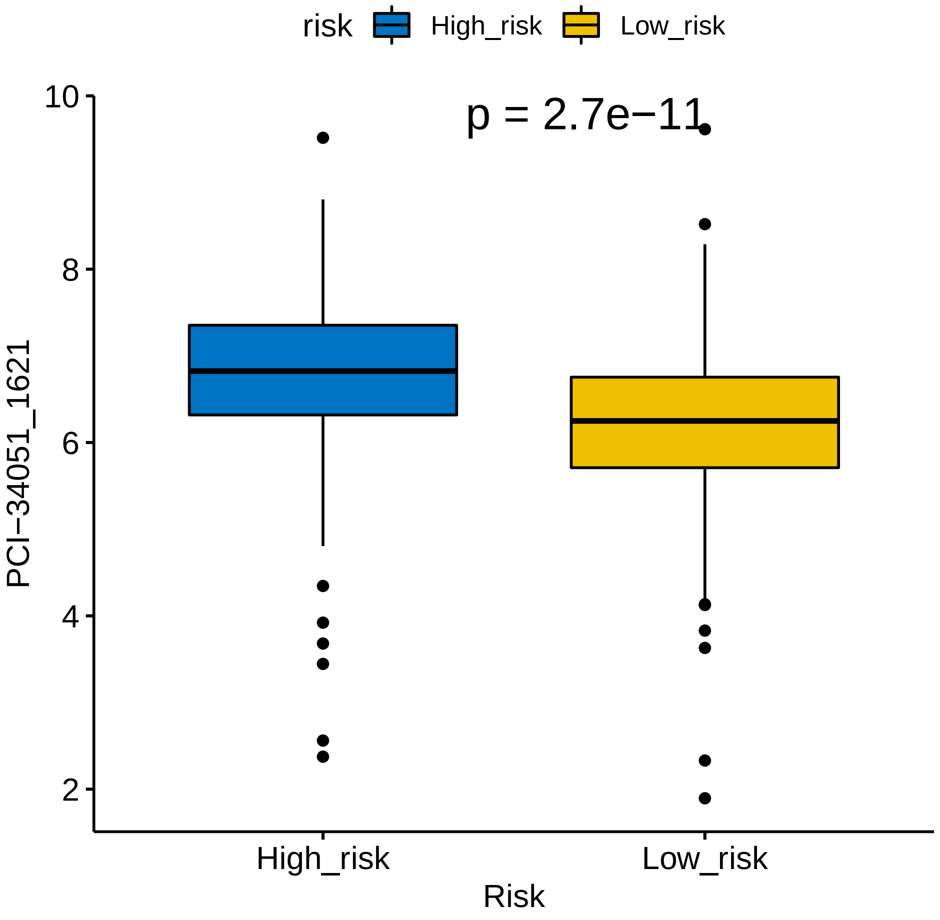

risk
High_risk
Low_risk
p = 2.7e−11
PCI−34051_1621
10
8
6
4
2
High_risk
Low_risk
Risk

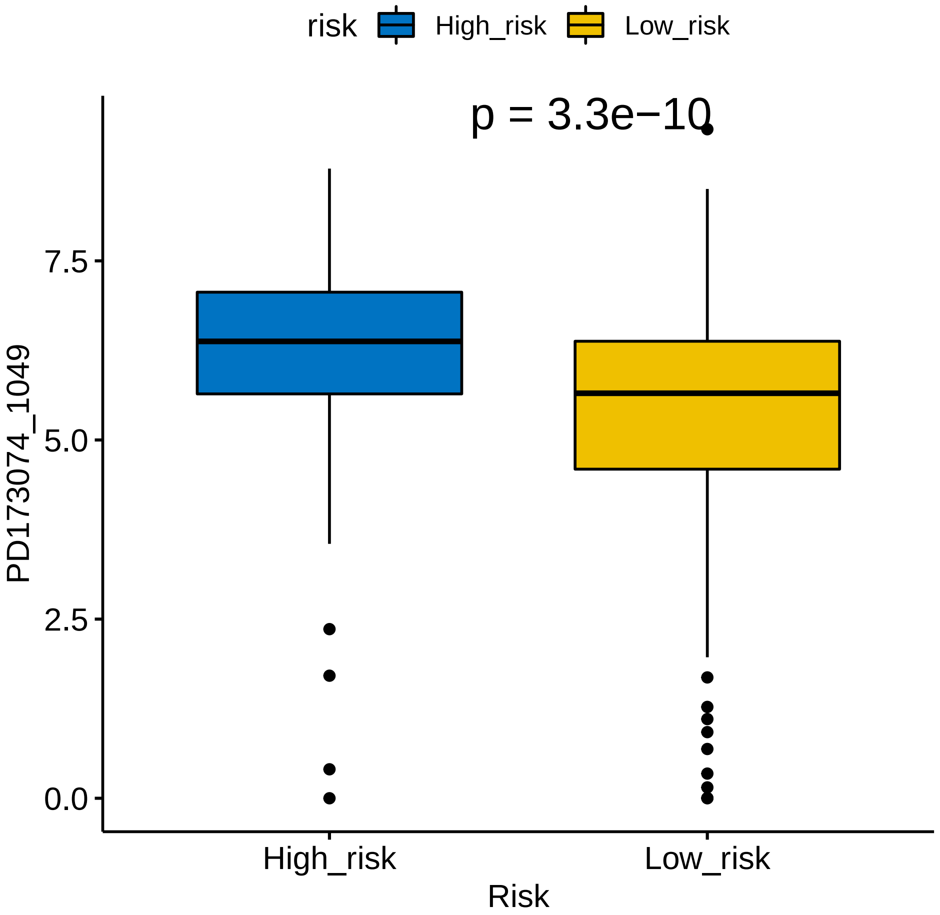

risk
High_risk
Low_risk
p = 3.3e−10
PD173074_1049
7.5
5.0
2.5
0.0
High_risk
Low_risk
Risk

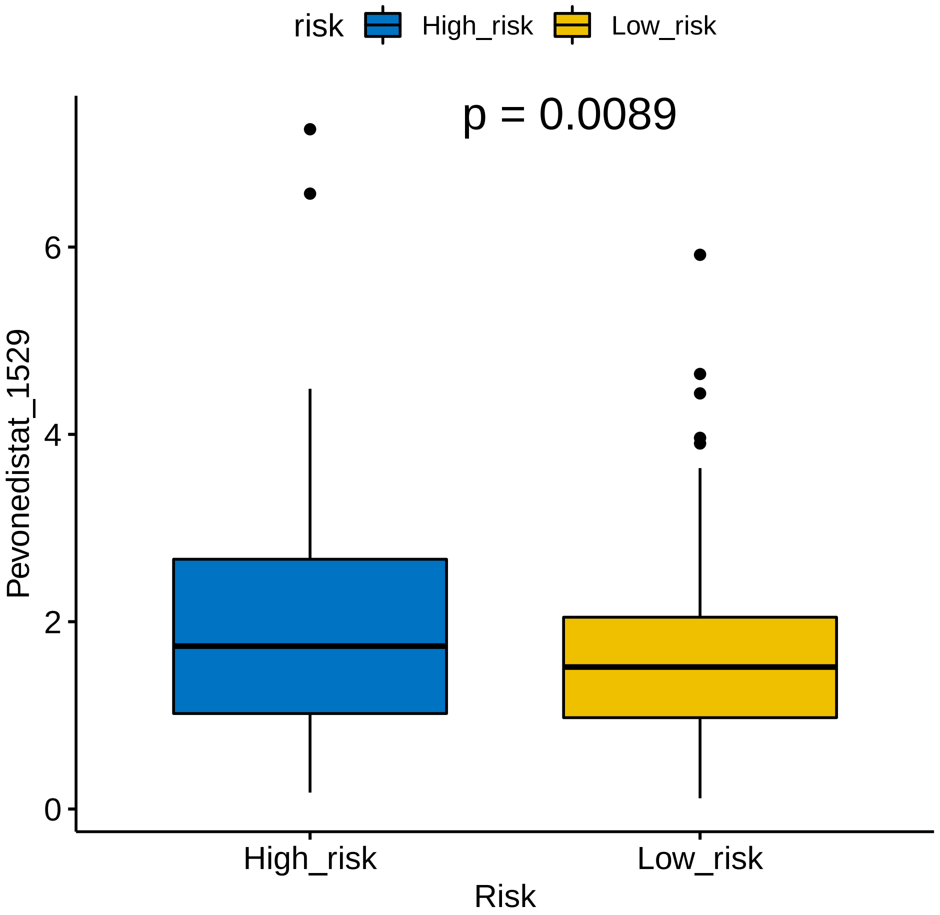

risk
High_risk
Low_risk
p = 0.0089
Pevonedistat_1529
6
4
2
0
High_risk
Low_risk
Risk

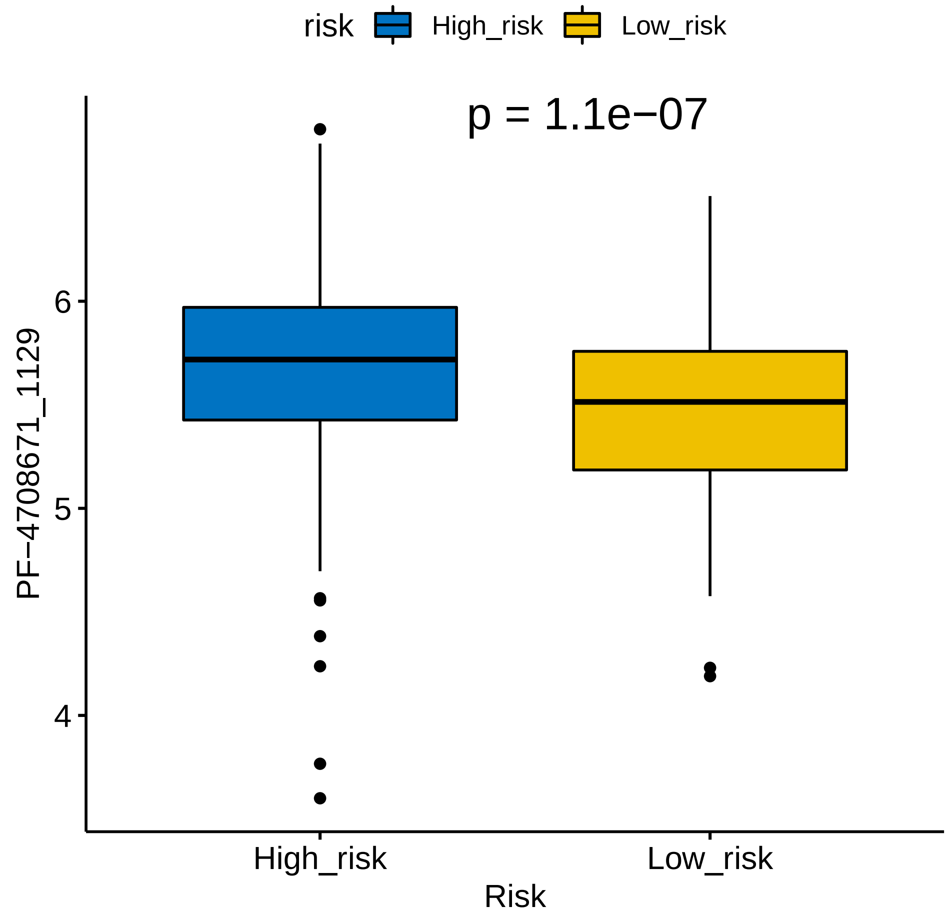

risk
High_risk
Low_risk
p = 1.1e−07
6
5
4
PF−4708671_1129
High_risk
Low_risk
Risk

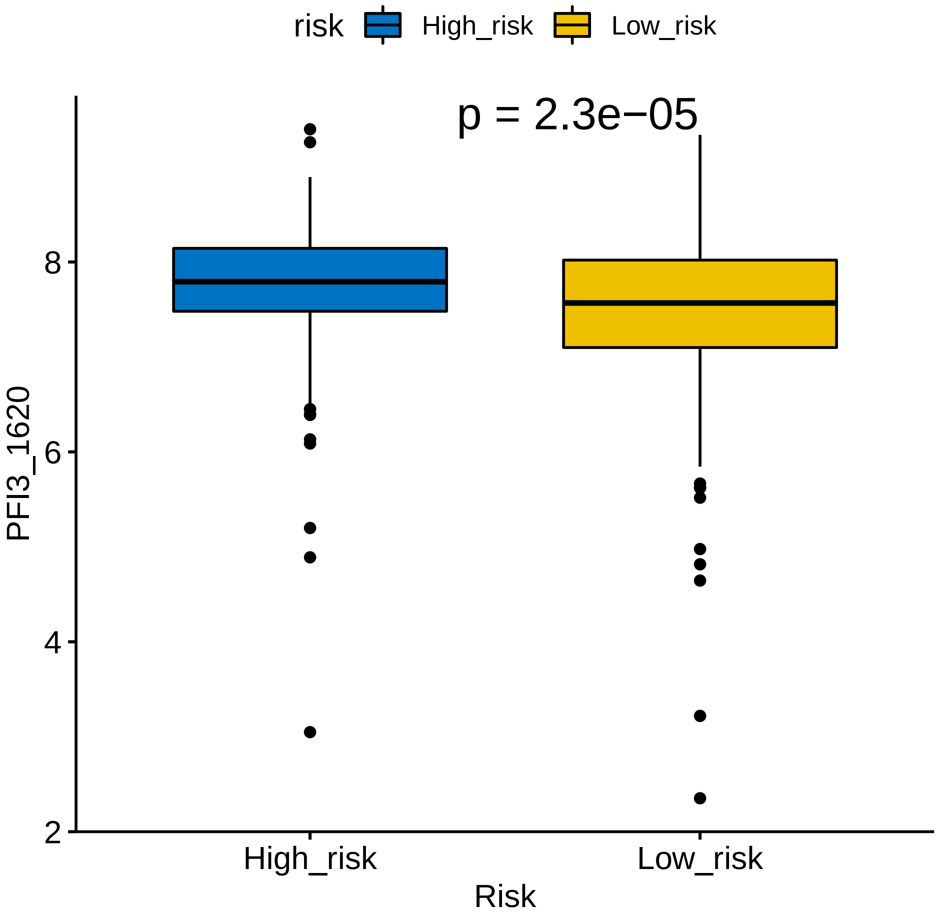

risk
High_risk
Low_risk
p = 2.3e−05
PFI3_1620
8
6
4
2
High_risk
Low_risk
Risk

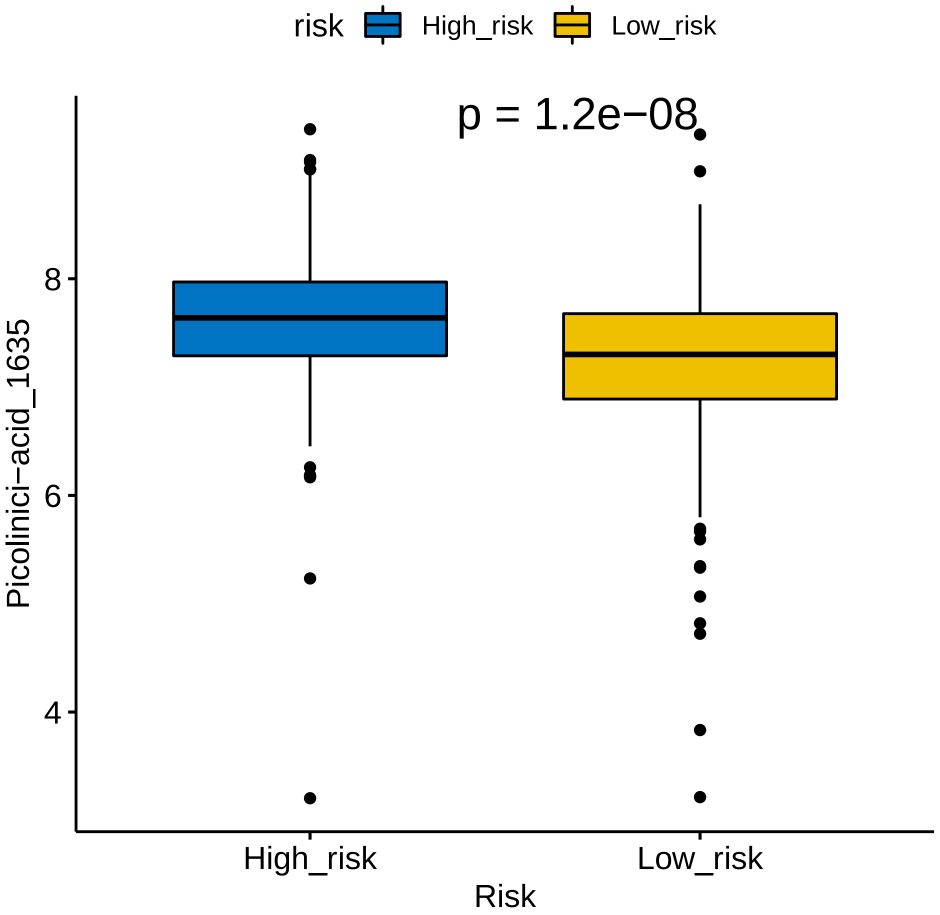

risk
High_risk
Low_risk
p = 1.2e−08
8
6
4
Picolinici−acid_1635
High_risk
Low_risk
Risk

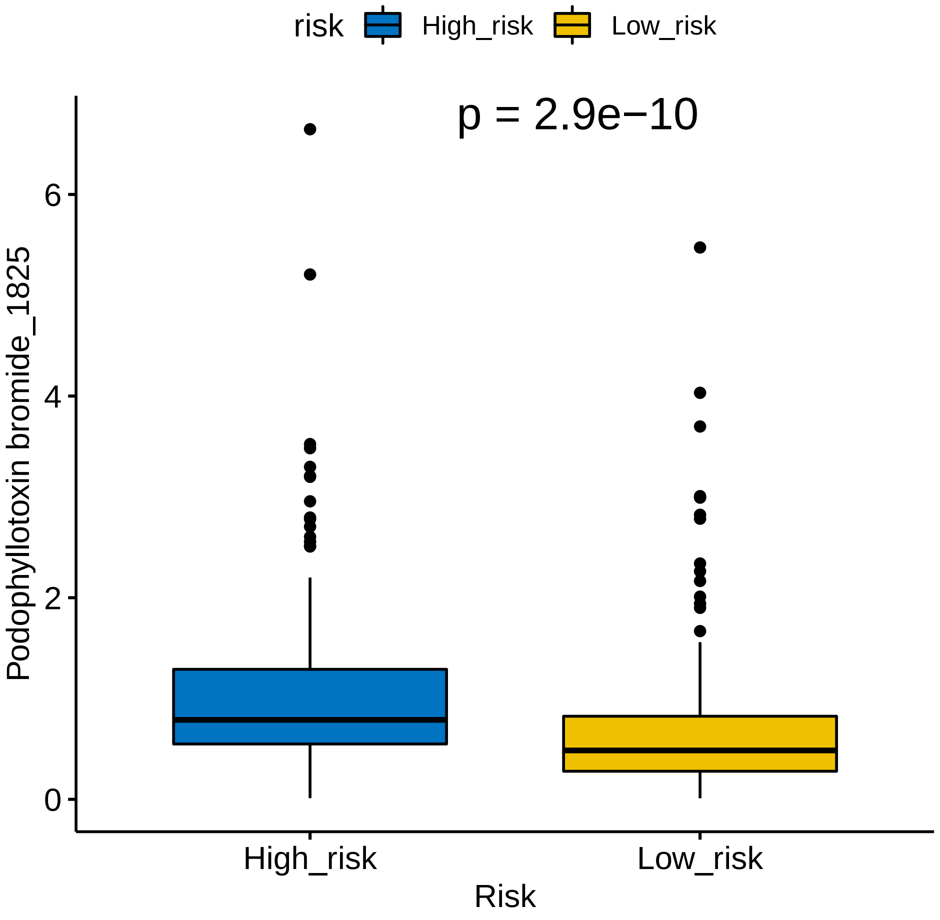

risk
High_risk
Low_risk
p = 2.9e−10
Podophyllotoxin bromide_1825
6
4
2
0
High_risk
Low_risk
Risk

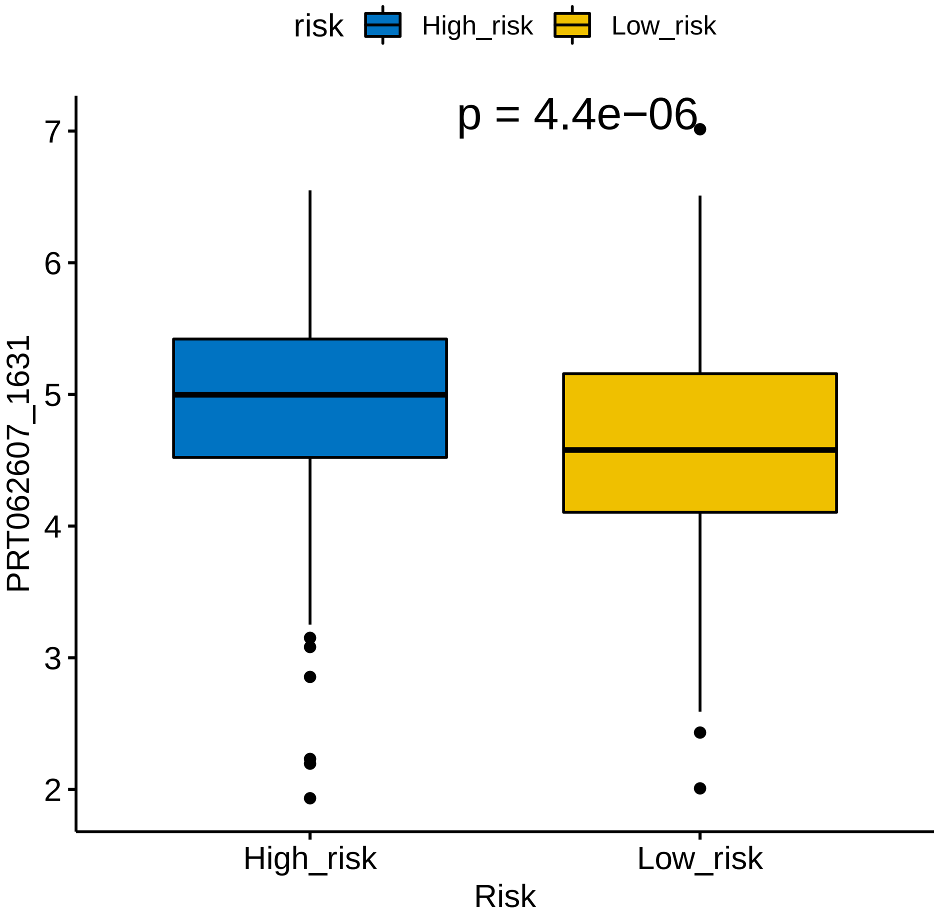

risk
High_risk
Low_risk
p = 4.4e−06
7
6
5
4
3
2
PRT062607_1631
High_risk
Low_risk
Risk

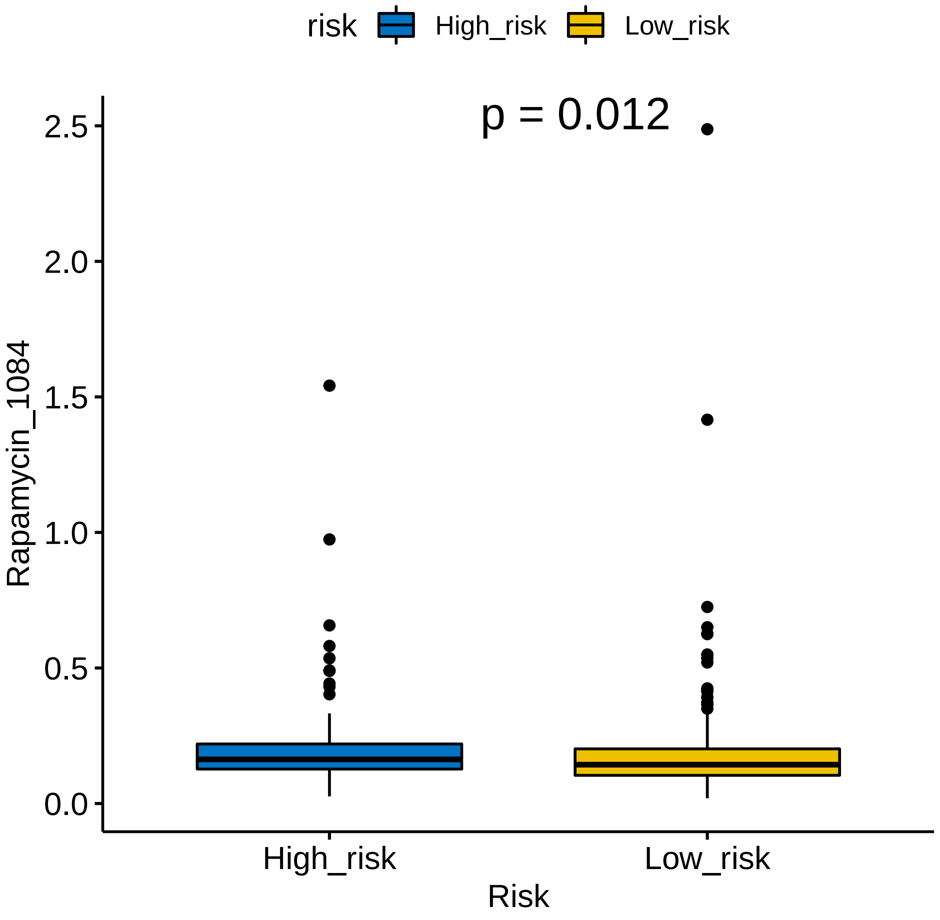
risk
High_risk
Low_risk
p = 0.012
2.5
2.0
1.5
1.0
0.5
0.0
Rapamycin_1084
High_risk
Low_risk
Risk

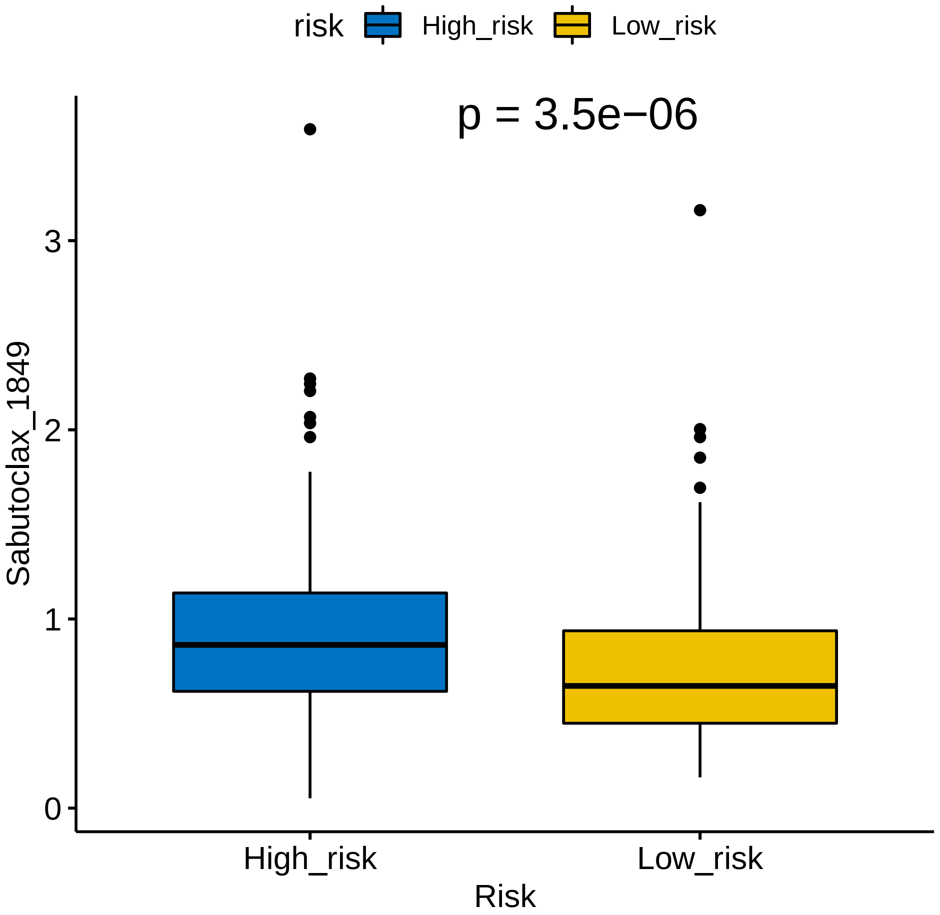

risk
High_risk
Low_risk
p = 3.5e−06
Sabutoclax_1849
0
1
2
3
High_risk
Low_risk
Risk

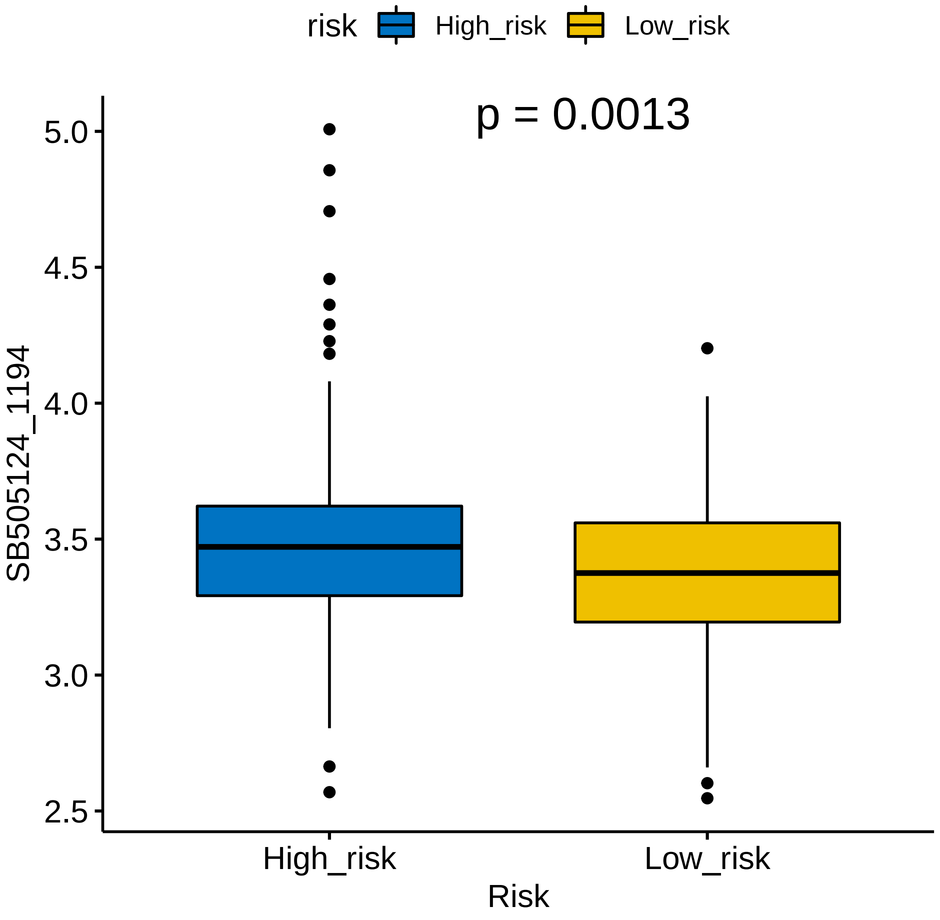

risk
High_risk
Low_risk
p = 0.0013
5.0
4.5
4.0
3.5
3.0
2.5
SB505124_1194
High_risk
Low_risk
Risk

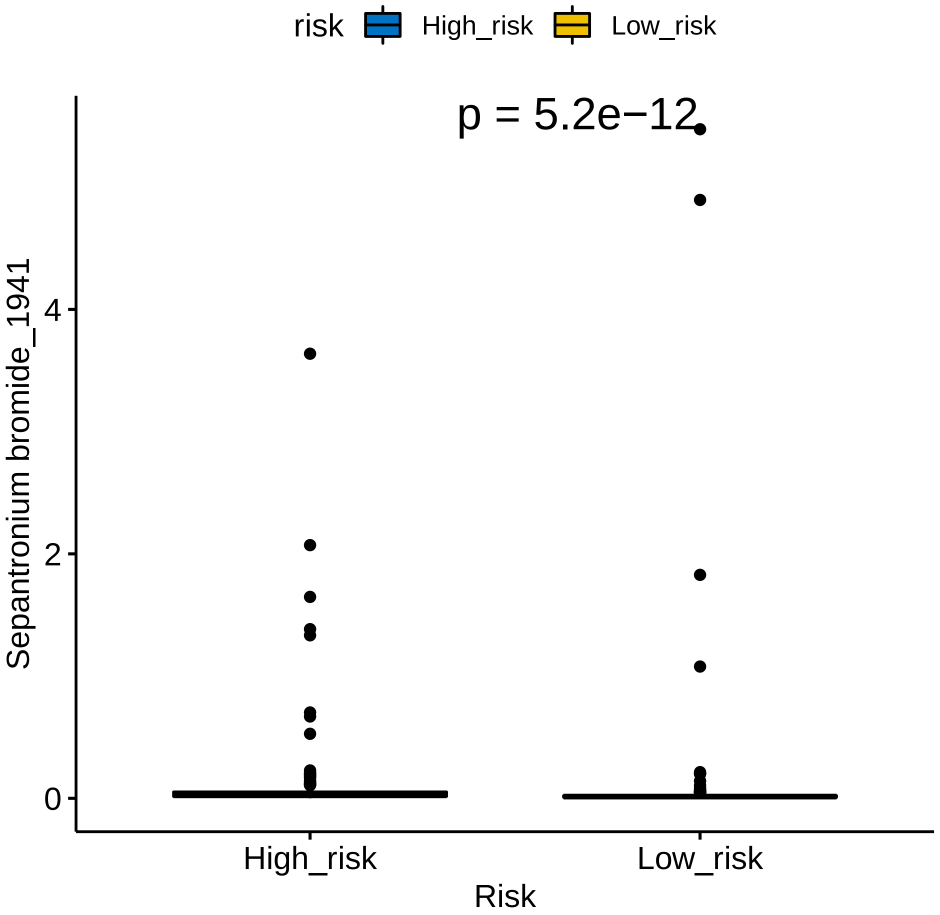

risk
High_risk
Low_risk
p = 5.2e−12
Sepantronium bromide_1941
4
2
0
High_risk
Low_risk
Risk

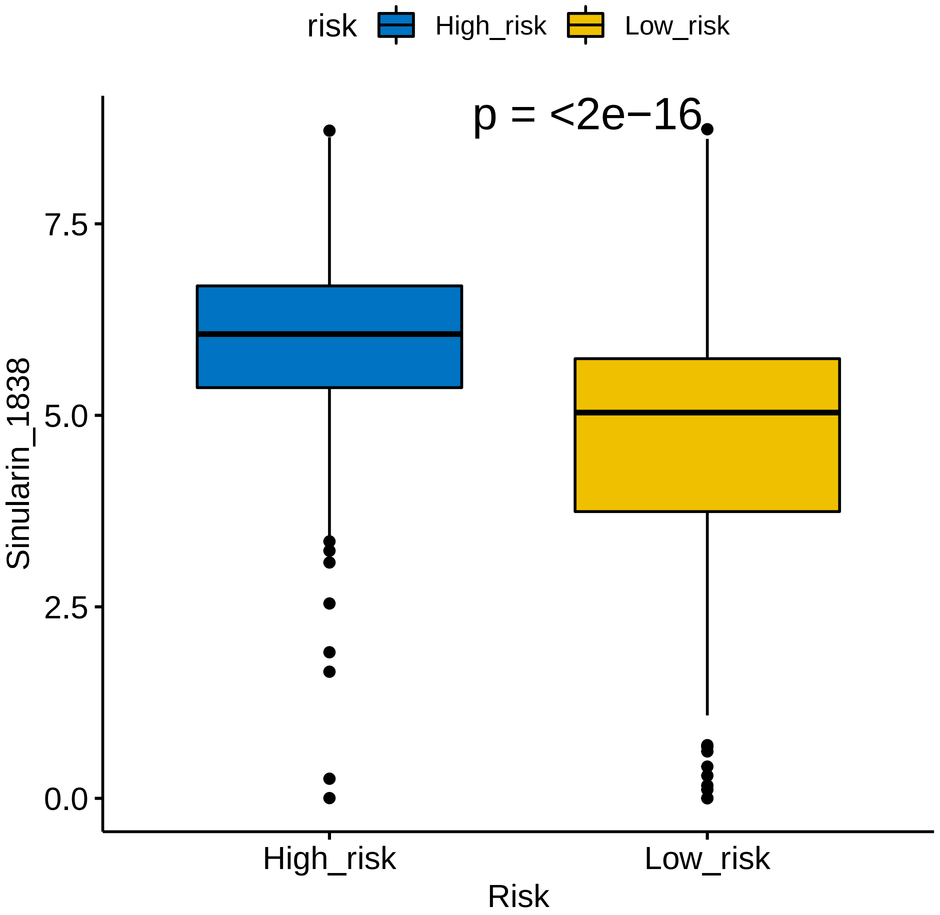

risk
High_risk
Low_risk
p = <2e−16
7.5
5.0
2.5
0.0
Sinularin_1838
High_risk
Low_risk
Risk

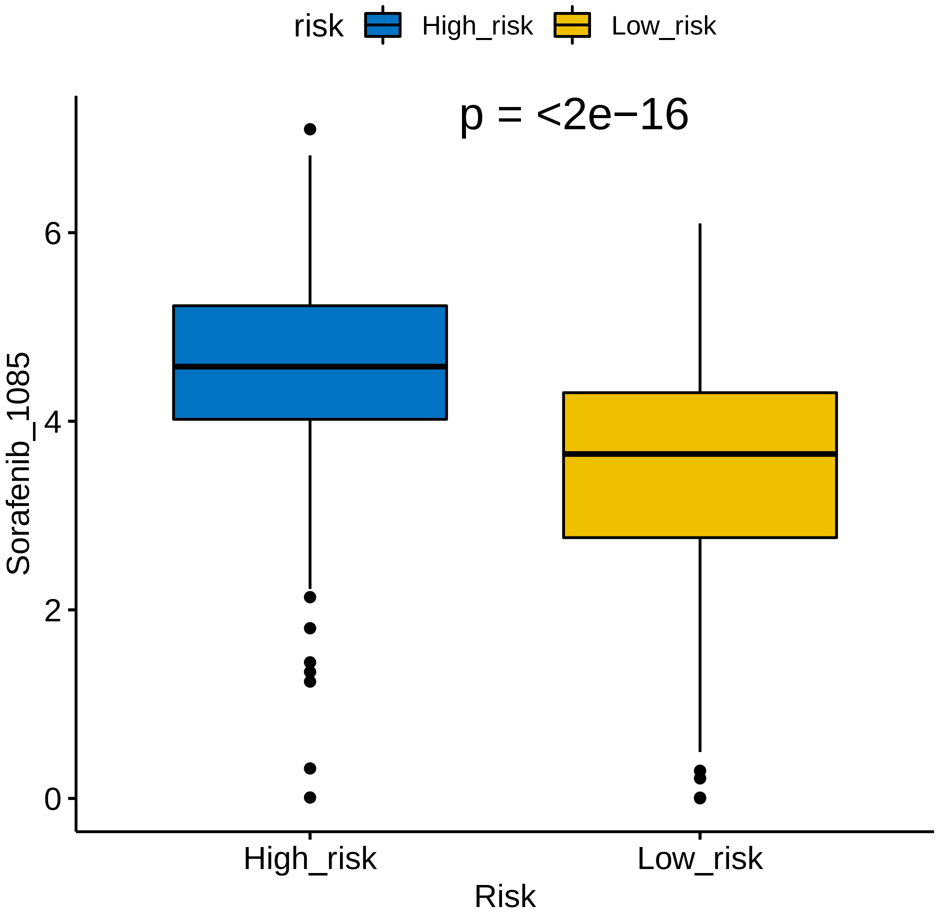
risk
High_risk
Low_risk
p = <2e−16
Sorafenib_1085
6
4
2
0
High_risk
Low_risk
Risk

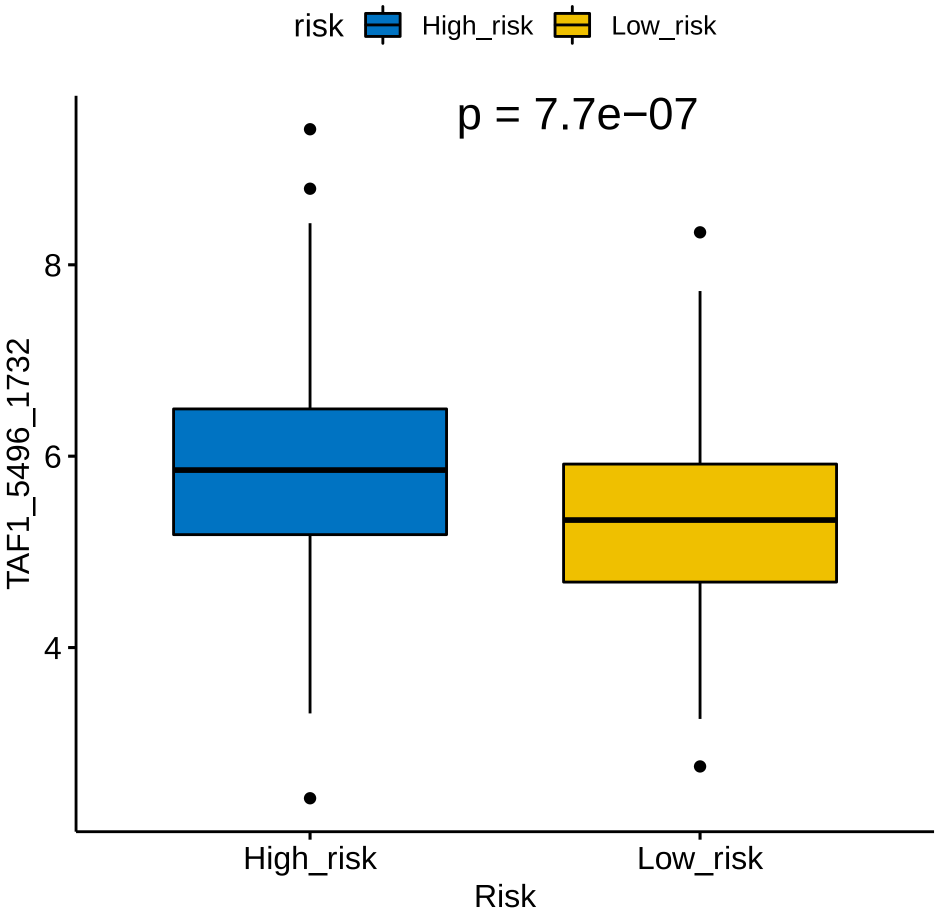
risk
High_risk
Low_risk
p = 7.7e−07
TAF1_5496_1732
8
6
4
High_risk
Low_risk
Risk

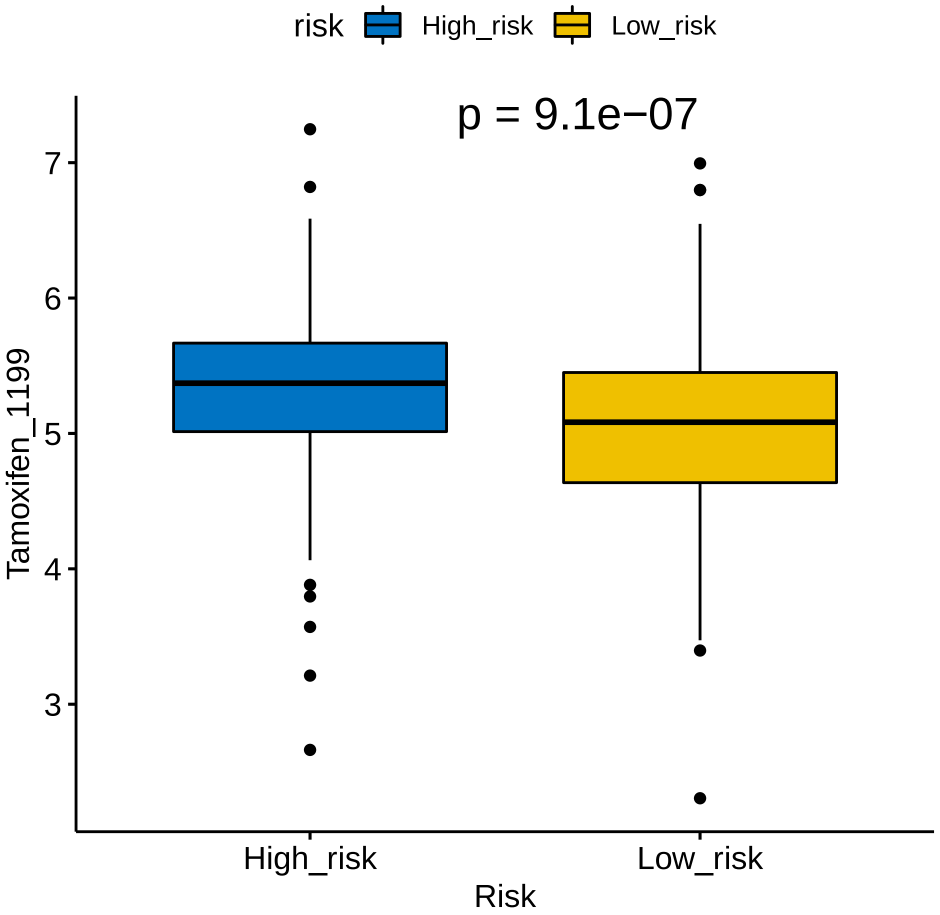

risk
High_risk
Low_risk
p = 9.1e−07
7
6
5
4
3
Tamoxifen_1199
High_risk
Low_risk
Risk

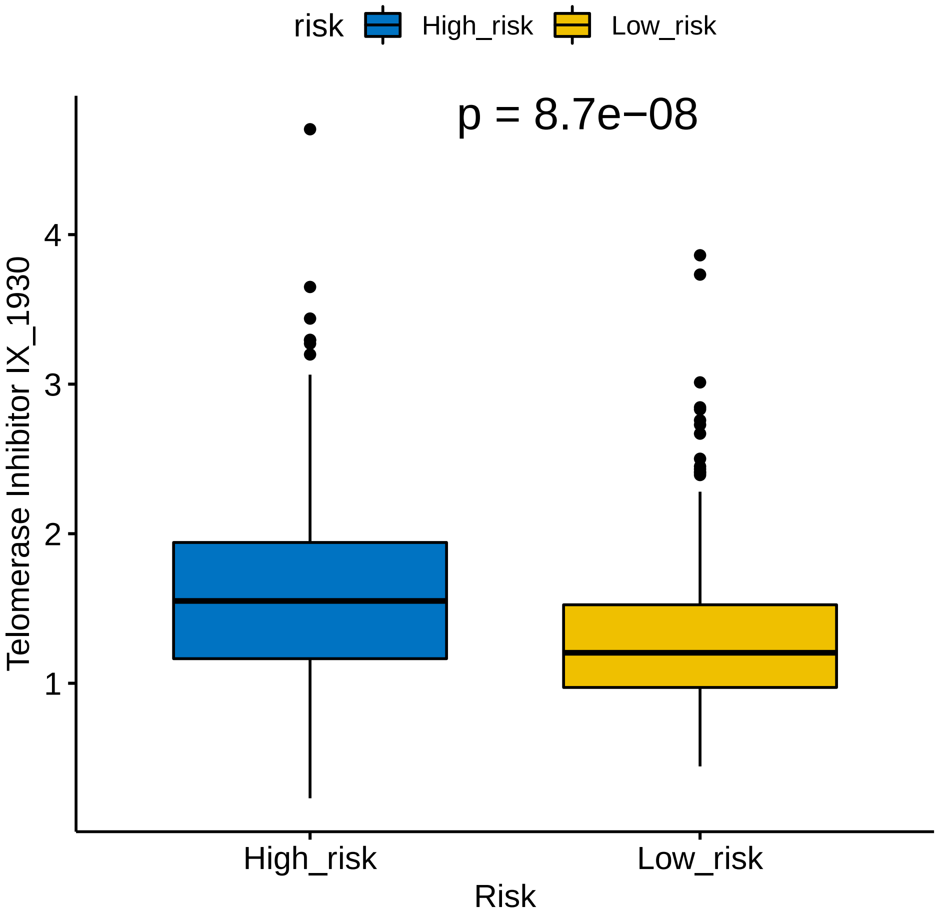
risk
High_risk
Low_risk
p = 8.7e−08
Telomerase Inhibitor IX_1930
4
3
2
1
High_risk
Low_risk
Risk

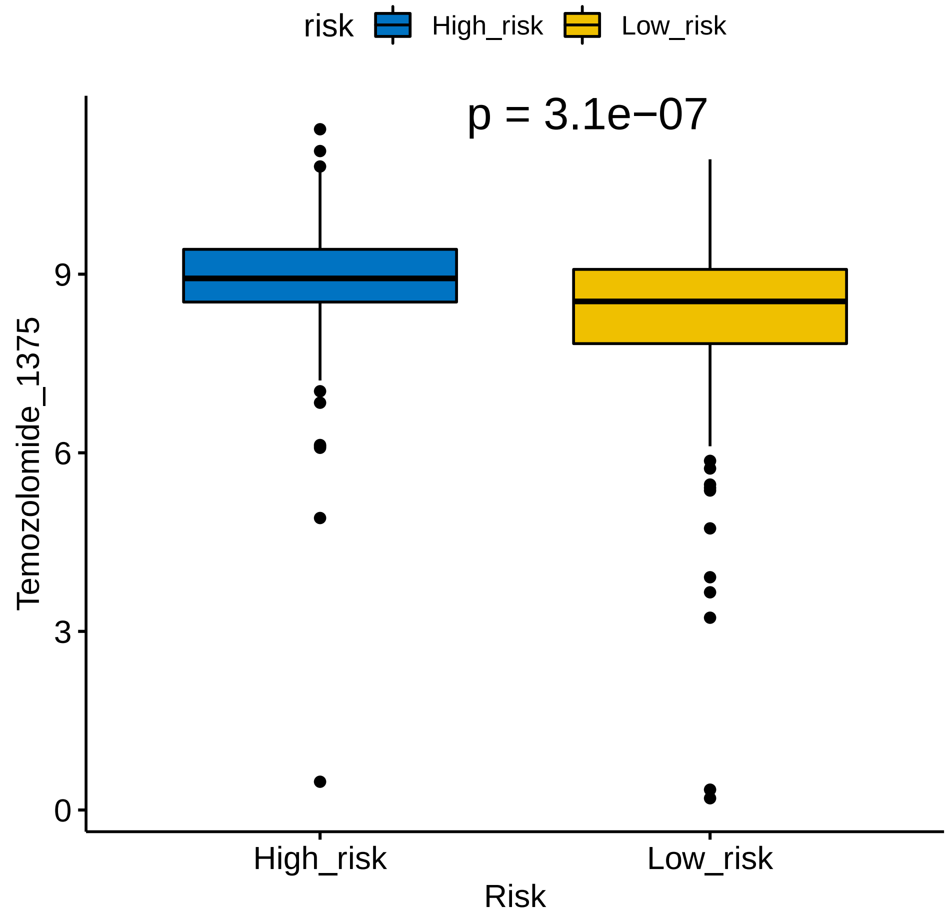

risk
High_risk
Low_risk
p = 3.1e−07
Temozolomide_1375
9
6
3
0
High_risk
Low_risk
Risk

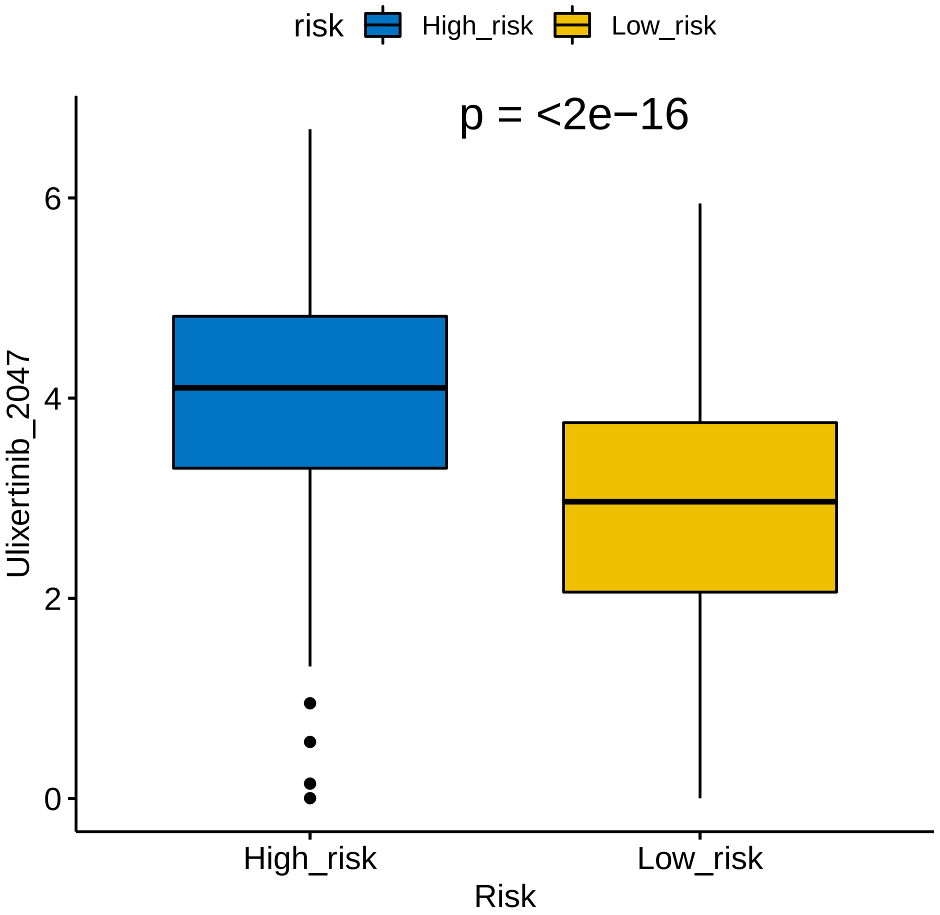
risk
High_risk
Low_risk
p = <2e−16
6
4
2
0
Ulixertinib_2047
High_risk
Low_risk
Risk

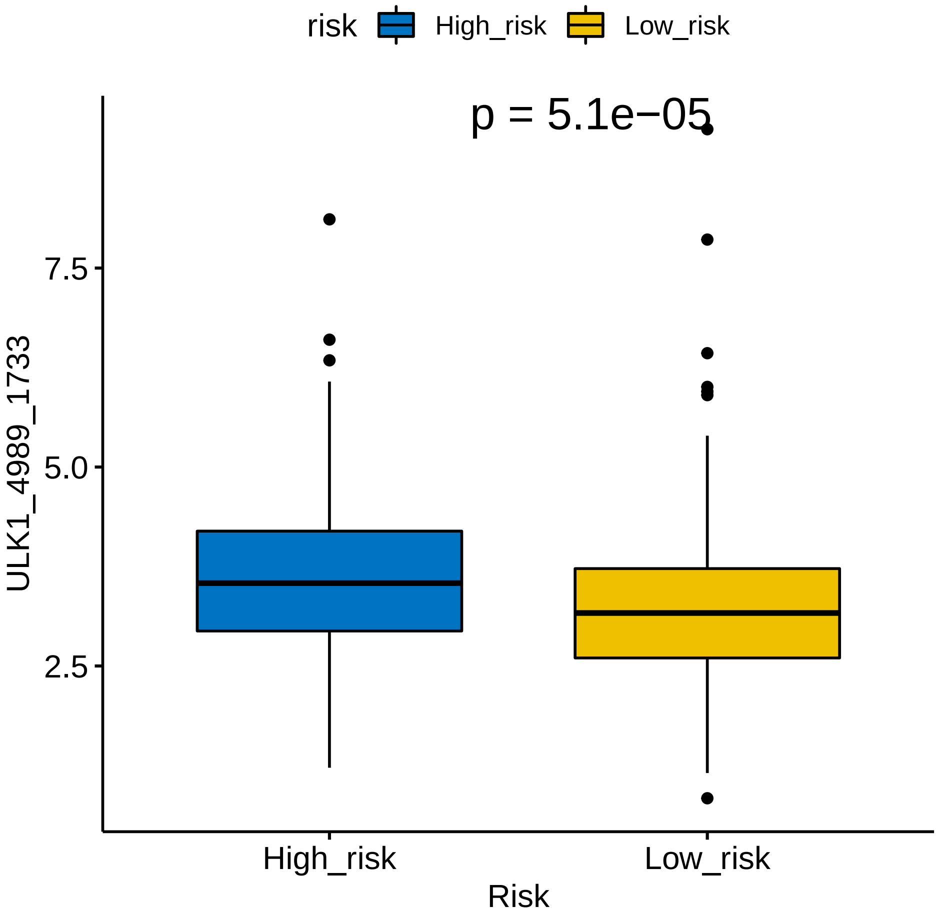

risk
High_risk
Low_risk
p = 5.1e−05
7.5
5.0
2.5
ULK1_4989_1733
High_risk
Low_risk
Risk

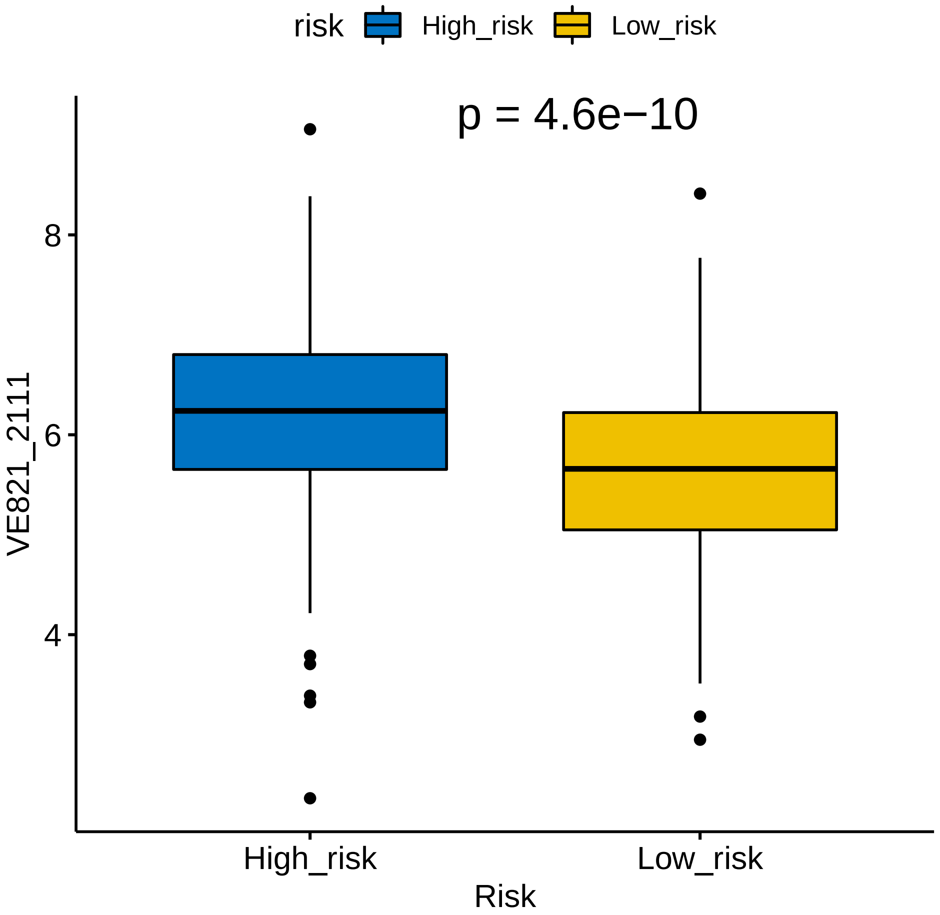

risk
High_risk
Low_risk
p = 4.6e−10
8
6
4
VE821_2111
High_risk
Low_risk
Risk

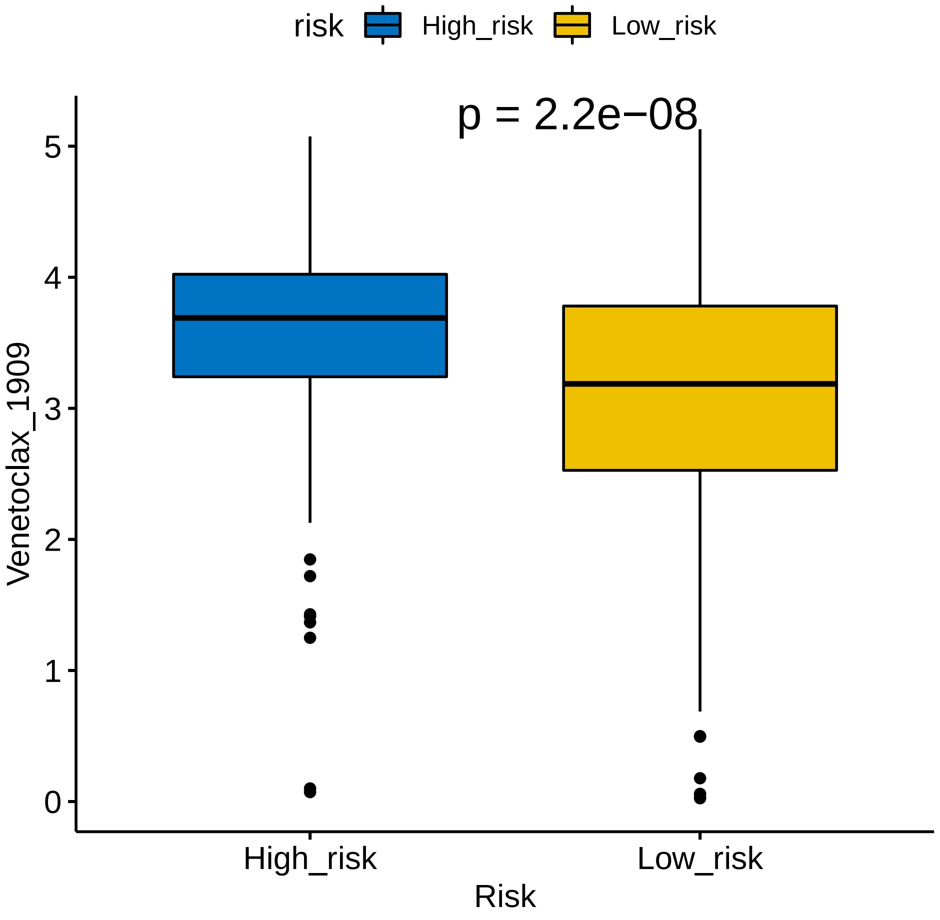

risk
High_risk
Low_risk
p = 2.2e−08
Venetoclax_1909
0
1
2
3
4
5
High_risk
Low_risk
Risk

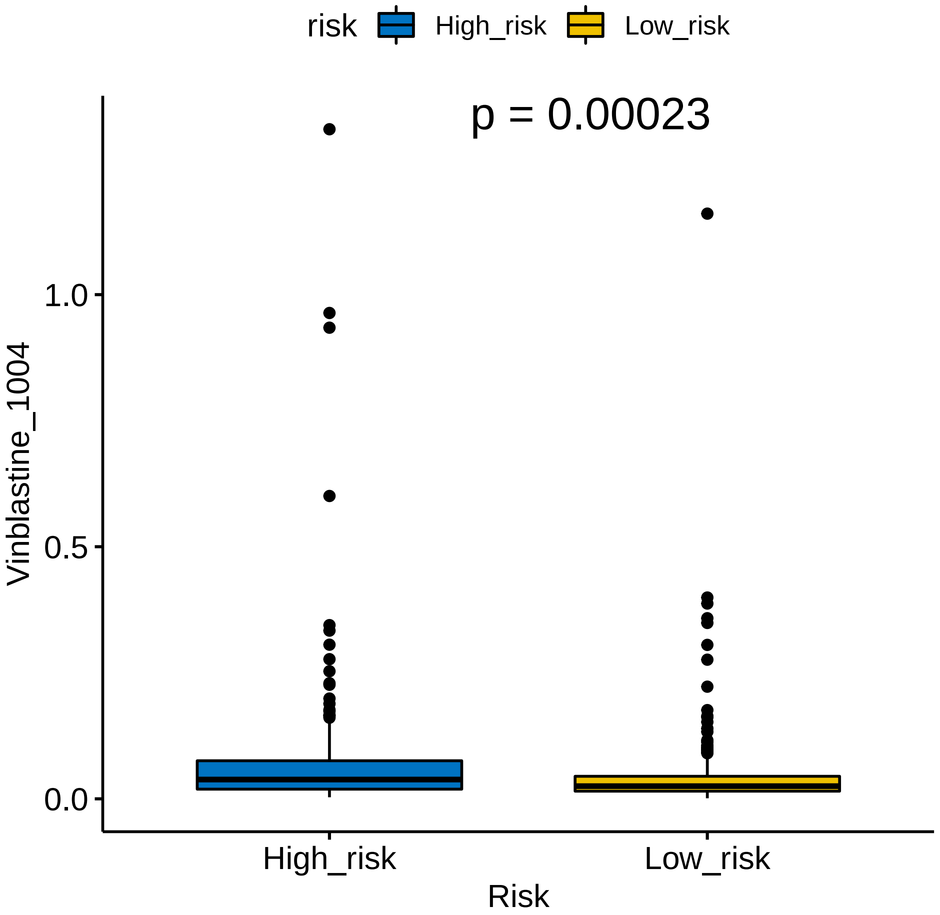
risk
High_risk
Low_risk
p = 0.00023
Vinblastine_1004
1.0
0.5
0.0
High_risk
Low_risk
Risk

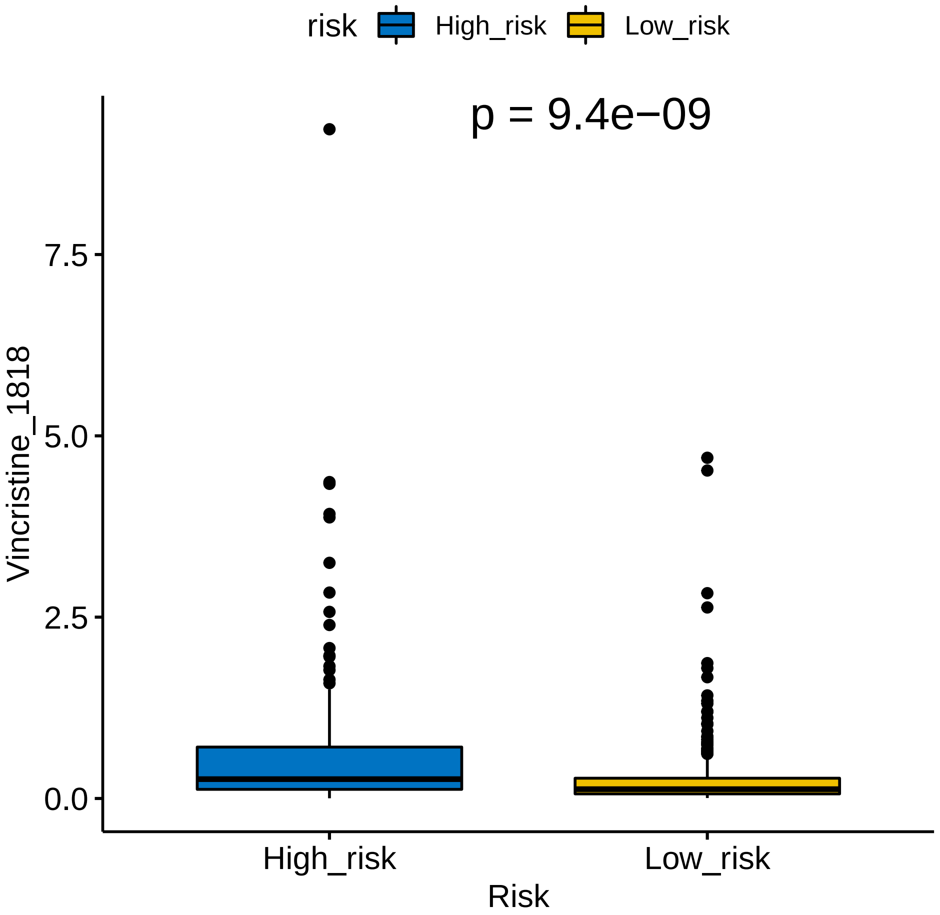

risk
High_risk
Low_risk
p = 9.4e−09
Vincristine_1818
7.5
5.0
2.5
0.0
High_risk
Low_risk
Risk

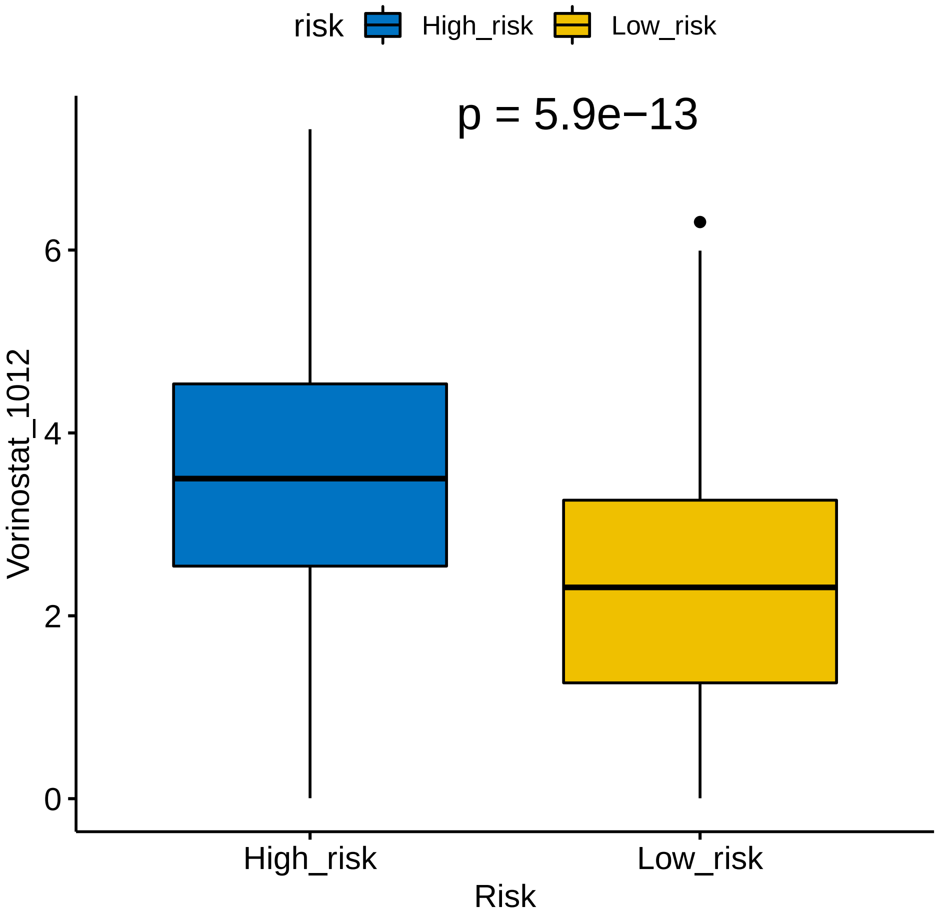

risk
High_risk
Low_risk
p = 5.9e−13
Vorinostat_1012
0
2
4
6
High_risk
Low_risk
Risk

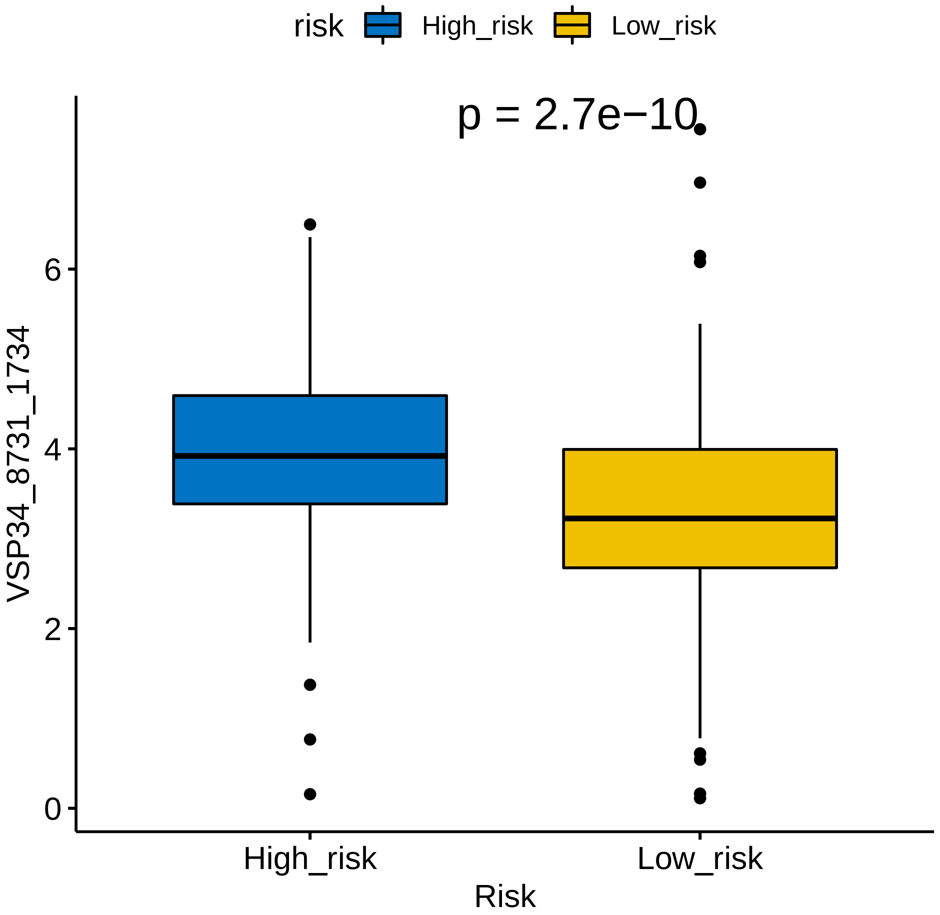

risk
High_risk
Low_risk
p = 2.7e−10
VSP34_8731_1734
6
4
2
0
High_risk
Low_risk
Risk

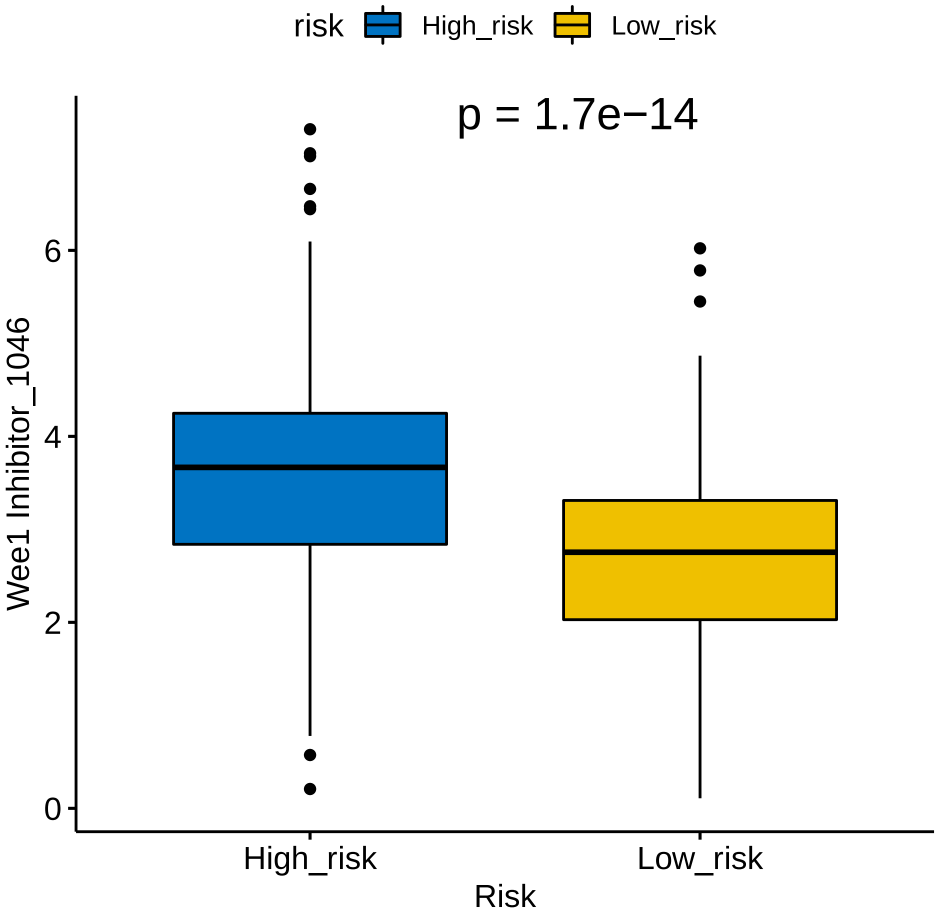
risk
High_risk
Low_risk
p = 1.7e−14
Wee1 Inhibitor_1046
6
4
2
0
High_risk
Low_risk
Risk

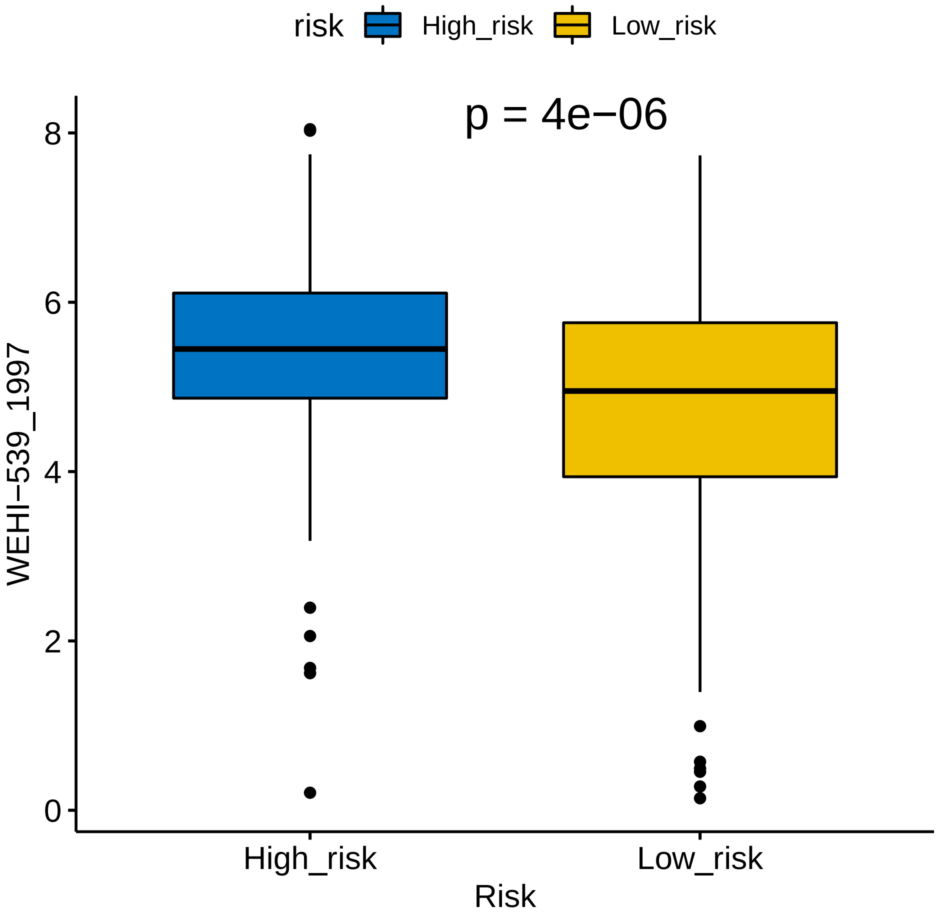

risk
High_risk
Low_risk
p = 4e−06
WEHI−539_1997
8
6
4
2
0
High_risk
Low_risk
Risk

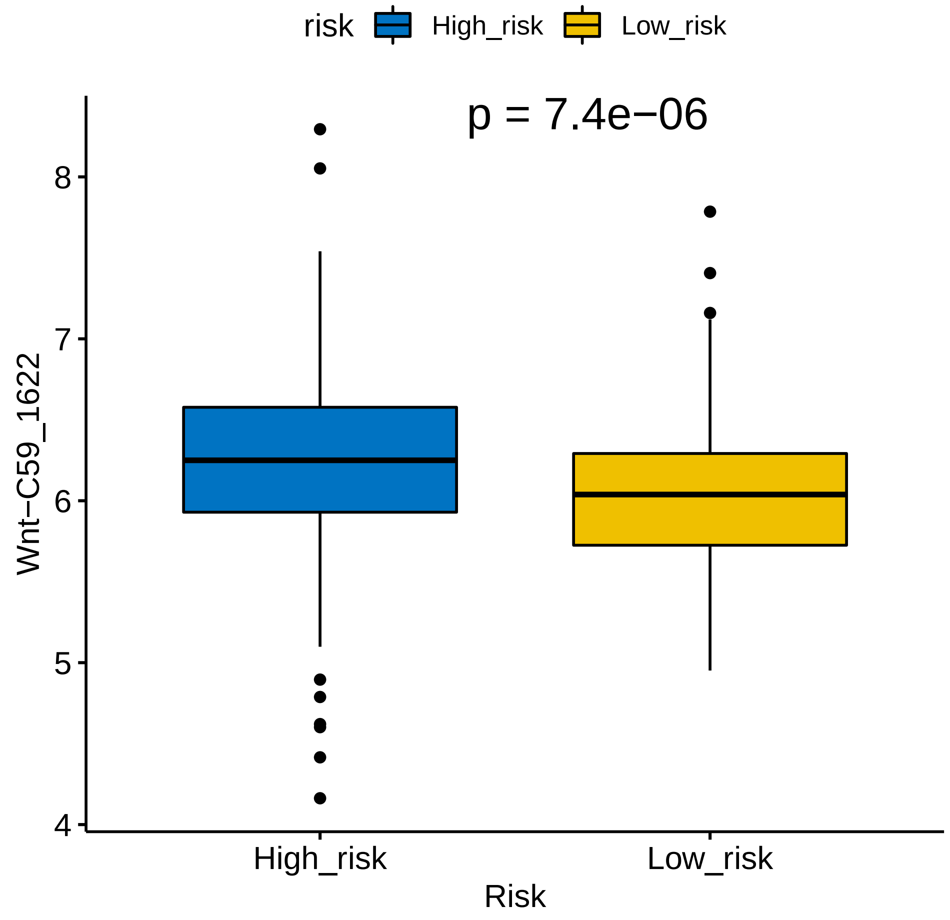

risk
High_risk
Low_risk
p = 7.4e−06
Wnt−C59_1622
8
7
6
5
4
High_risk
Low_risk
Risk

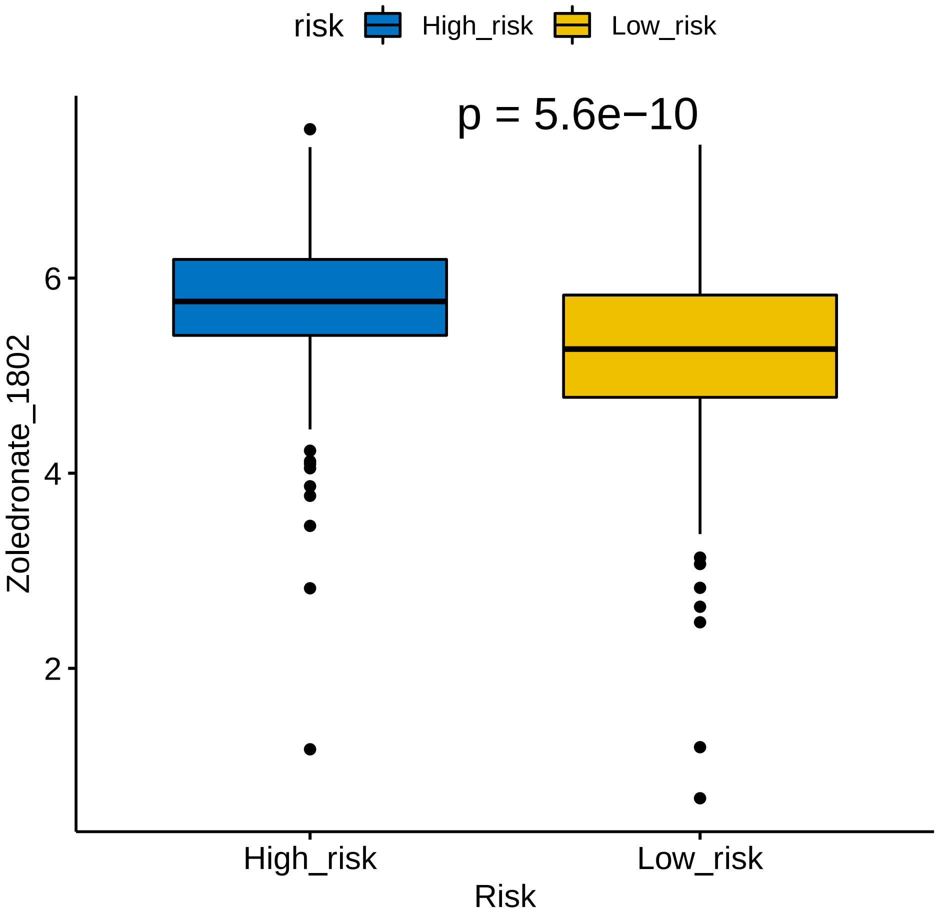
risk
High_risk
Low_risk
p = 5.6e−10
Zoledronate_1802
6
4
2
High_risk
Low_risk
Risk
