# Supplementary material for: Prognosis analysis and validation of lipid metabolism-associated lncRNAs and tumor immune microenvironment in bladder cancer
Source: Aging (Albany NY). 2023 Aug 24;15(16):8384–407. doi: 10.18632/aging.204975 (PMC10496992; doi:10.18632/aging.204975)
Supplement: Supplementary Tables [file aging-15-204975-s004.pdf]

SUPPLEMENTARY TABLES

Supplementary Table 1. Primer sequences used for reverse transcription-quantitative PCR.

Genes	Primer sequence (5′→3′)
MIR100HG	F: AACTTGGCTTCCTCGCTTCT<br>R: CTTTGTCCTTGCACTGGGGA
LINC00865	F: ACTGGATGTTCCCAGTTCTC<br>R: CTTGTACCCAACAGAGTCTC
RP11-465B22.8	F: GTACCCAGAGCCTCCCTGTG<br>R: GGTGCGTGAACTGCAGACTT
GAPDH	F: ACAACTTTGGTATCGTGGAAGG<br>R: GCCATCACGCCACAGTTTC

Supplementary Table 2. 47 DE-LMRGs between BLCA samples and normal samples.

Symbol	logFC	AveExpr	*t*	*P*.Value	adj.*P*.Val	B
RP11-867G23.10	−1.946136	0.392076	−17.54711	2.02E-52	7.08E-49	108.4001
ADAMTS9-AS1	−1.623903	0.223178	−16.94116	1.00E-49	2.81E-46	102.2615
AF131217.1	−1.614079	0.274368	−16.21864	1.55E-46	2.63E-43	94.99816
LINC01082	−3.160091	0.95484	−16.21037	1.69E-46	2.63E-43	94.91551
AF001548.6	−1.76174	0.299834	−15.06717	1.59E-41	1.86E-38	83.5865
PGM5-AS1	−4.159291	0.786111	−14.6306	1.18E-39	1.04E-36	79.32468
HAND2-AS1	−1.176201	0.205877	−13.96947	7.46E-37	5.24E-34	72.95189
MIR1-1HG-AS1	−1.245398	0.205737	−13.91137	1.31E-36	8.35E-34	72.39697
CTC-296K1.4	−2.853364	0.627719	−13.57402	3.35E-35	1.88E-32	69.19224
CTC-296K1.3	−3.153153	0.830367	−13.30481	4.35E-34	2.07E-31	66.65728
FENDRR	−2.709976	0.966052	−13.0049	7.38E-33	3.05E-30	63.85817
AC002398.12	−1.049263	0.183382	−12.90645	1.86E-32	7.25E-30	62.94532
RP11-6O2.3	−1.316601	0.317031	−12.76854	6.75E-32	2.43E-29	61.67173
RP11-887P2.5	−1.357555	0.212235	−12.75687	7.52E-32	2.64E-29	61.56426
MBNL1-AS1	−1.72294	0.658881	−12.19028	1.40E-29	4.10E-27	56.40006
C5orf66-AS1	−1.924639	0.594624	−12.00317	7.68E-29	2.07E-26	54.71935
NR4A1AS	−1.830838	0.796489	−11.65192	1.81E-27	4.71E-25	51.59942
RP11-554A11.4	−1.414086	0.550645	−11.18615	1.11E-25	2.73E-23	47.53737
RP11-498E2.9	−1.801452	0.590403	−11.06008	3.33E-25	7.79E-23	46.45329
MIR100HG	−2.008502	0.927027	−10.96475	7.60E-25	1.67E-22	45.63815
RP11-753H16.3	−1.300328	0.294339	−10.29824	2.19E-22	4.10E-20	40.05281
AC053503.6	−2.202909	0.608135	−9.780534	1.54E-20	2.46E-18	35.86101
ACTA2-AS1	−1.356784	0.568752	−9.43741	2.40E-19	3.54E-17	33.15849
LINC00641	−1.016138	0.941535	−8.8736	1.90E-17	2.36E-15	28.85736
LINC00865	−1.443252	1.190996	−8.647051	1.05E-16	1.21E-14	27.18032
SNHG1	1.359221	3.883086	8.504292	3.02E-16	3.37E-14	26.13921
ZNF710-AS1	−1.131329	1.045621	−8.334389	1.05E-15	1.11E-13	24.91625
RP11-553L6.5	−1.459297	2.427242	−8.205353	2.67E-15	2.78E-13	23.99935
MIR22HG	−1.623176	2.80716	−8.154589	3.85E-15	3.93E-13	23.64149
SNHG12	1.215186	2.953564	7.770595	5.76E-14	5.43E-12	20.9878

RGS5	−2.265292	3.381771	−7.397829	7.28E-13	6.12E-11	18.50441
CTD-2510F5.4	1.741091	2.899735	6.744602	4.94E-11	3.24E-09	14.38371
RP11-703I16.1	−1.070464	1.291835	−6.362939	5.06E-10	2.89E-08	12.11862
RP11-303E16.2	1.103857	3.361274	6.353876	5.34E-10	3.03E-08	12.06616
SNHG3	1.064048	3.218649	6.257583	9.43E-10	5.25E-08	11.51253
SNHG25	1.263032	2.143639	6.223272	1.15E-09	6.38E-08	11.31696
RP6-65G23.3	1.189491	2.2966	6.214588	1.21E-09	6.66E-08	11.2676
PCAT6	1.398388	2.868352	6.047879	3.18E-09	1.64E-07	10.33132
PPP1R14B-AS1	1.364422	2.451623	6.041606	3.30E-09	1.68E-07	10.29651
RP11-295G20.2	1.150859	2.650169	5.833411	1.07E-08	5.01E-07	9.158466
PCCA-DT	1.042885	3.223762	5.693309	2.31E-08	1.01E-06	8.411815
MAFG-DT	1.060909	1.787521	5.553827	4.89E-08	2.01E-06	7.683991
CTD-2537I9.12	1.085947	2.004335	5.432102	9.32E-08	3.67E-06	7.061609
MIR23AHG	−1.289774	3.522301	−5.077265	5.71E-07	1.93E-05	5.316384
CTA-384D8.36	1.024862	2.872703	5.076098	5.74E-07	1.93E-05	5.310814
SPINT1-AS1	1.214653	3.397013	4.904587	1.33E-06	4.08E-05	4.504871
RP1-86C11.7	1.11661	1.428936	4.884413	1.46E-06	4.43E-05	4.411697
RP3-523K23.2	1.2478	3.216132	4.737496	2.94E-06	8.19E-05	3.743546
RP11-50C13.1	1.215676	2.771355	4.662735	4.17E-06	0.000112	3.410601
MIR200CHG	1.535653	3.953889	4.552689	6.90E-06	0.000176	2.929239
CH17-360D5.2	1.037275	2.066593	4.330007	1.86E-05	0.000416	1.987211
RP11-670E13.6	1.005279	2.343464	4.307895	2.04E-05	0.000449	1.896027
VPS9D1-AS1	1.023731	1.982811	4.289357	2.21E-05	0.000478	1.819913
LASTR	1.365122	1.51384	4.130502	4.35E-05	0.000865	1.18005
RP3-406A7.7	1.020943	2.769143	3.805855	0.000162	0.00266	−0.05795
NUDT19-DT	1.047823	2.401061	3.661312	0.000282	0.004262	−0.578701
CASC9	1.057251	2.31302	3.558255	0.000415	0.005954	−0.938417
KLHDC7B-DT	1.036457	1.506904	3.448439	0.000619	0.008343	−1.311059
IGFL2-AS1	1.268883	1.462697	3.441953	0.000634	0.008483	−1.332724
RP11-465B22.8	1.051318	2.533107	3.218674	0.001385	0.015924	−2.054886
KRT7-AS	1.18984	2.819216	3.120334	0.001928	0.020737	−2.358319
LINC01518	1.007683	1.091127	3.116905	0.00195	0.020879	−2.368739
AATBC	1.01853	1.893502	3.097783	0.002077	0.021891	−2.426634
LINC01819	1.107012	1.721722	3.076947	0.002224	0.023219	−2.489333
CTD-2021H9.3	1.073609	1.558676	3.026578	0.002621	0.02628	−2.639229
LINC01133	−1.074726	1.431864	−3.026473	0.002622	0.02628	−2.639537
LINC01980	1.139638	1.779023	3.010273	0.002763	0.027435	−2.687243
RP11-109M17.2	1.101517	1.469562	2.91865	0.003699	0.03419	−2.952421
RP11-54H7.4	1.044085	1.0726	2.86592	0.004361	0.038776	−3.101465
MIR205HG	1.182663	4.109484	2.852583	0.004545	0.040029	−3.138748

Supplementary Table 3. 70 DE-LncRNAs between BLCA samples and normal samples.

Symbol	logFC	AveExpr	*t*	*P*.Value	adj.*P*.Val	B
SLC2A4	−2.489706	0.5449	−14.73967	3.90E-40	3.03E-37	80.29017
ACACB	−1.481464	0.853814	−11.91687	1.65E-28	6.38E-26	53.95403
ACOX2	−1.312222	0.572971	−10.45182	5.97E-23	1.54E-20	41.37253
EBF1	−1.41762	0.819794	−10.06465	1.50E-21	2.91E-19	38.20695
PLPP3	−1.987317	3.201628	−9.892062	6.17E-21	9.57E-19	36.81955
PTGS1	−3.376291	2.15695	−9.458587	2.01E-19	1.73E-17	33.40224
BCHE	−1.790734	0.603735	−9.315577	6.21E-19	4.81E-17	32.29667
CH25H	−1.967207	1.348607	−8.859131	2.11E-17	1.48E-15	28.84382
KLF4	−2.326709	3.360207	−8.705745	6.70E-17	4.00E-15	27.71024
TNFAIP8L3	−1.797003	1.221633	−8.67609	8.37E-17	4.63E-15	27.49268
PPP1CA	1.071662	6.440257	8.221976	2.36E-15	1.14E-13	24.22707
LYPLA2	1.151503	5.802534	8.1729	3.36E-15	1.53E-13	23.88175
PRKAG2	−1.060382	1.902452	−8.161794	3.64E-15	1.57E-13	23.8038
MGLL	−1.835234	2.055851	−8.02189	9.86E-15	3.82E-13	22.82864
EBP	1.3197	4.78328	7.927315	1.92E-14	7.09E-13	22.17649
PTGIS	−3.008734	1.896224	−7.887455	2.54E-14	8.96E-13	21.90335
EGR2	−1.824696	1.560406	−7.762461	6.07E-14	2.05E-12	21.05352
PLA2G5	−1.260114	0.633826	−7.669621	1.15E-13	3.72E-12	20.42893
DHCR7	1.515731	4.107957	7.572913	2.23E-13	6.27E-12	19.78439
OSBPL10	−1.180758	1.506631	−7.558883	2.45E-13	6.55E-12	19.6914
SQLE	1.648506	4.107132	7.286094	1.52E-12	3.94E-11	17.9098
PPM1L	−1.252799	1.444074	−7.262629	1.78E-12	4.45E-11	17.75892
PLA2G15	1.004141	3.075382	6.977152	1.14E-11	2.75E-10	15.95402
EPHX2	−1.389083	1.549747	−6.799022	3.51E-11	7.77E-10	14.85701
PTGDS	−2.745181	2.897305	−6.619111	1.07E-10	2.19E-09	13.77228
MBOAT7	1.195575	5.304839	6.251062	9.78E-10	1.76E-08	11.62759
FASN	1.370834	5.271571	6.09541	2.42E-09	4.06E-08	10.75128
HILPDA	1.691945	4.169001	6.041664	3.29E-09	5.14E-08	10.453
PTGS2	−2.310917	3.002981	−5.890265	7.75E-09	1.05E-07	9.624785
UGCG	−1.019877	3.887731	−5.823019	1.13E-08	1.46E-07	9.262642
TBL1X	−1.018187	2.468248	−5.693197	2.30E-08	2.88E-07	8.573581
TRIB3	1.377929	3.115824	5.444608	8.72E-08	8.89E-07	7.291557
SCD	1.793232	5.687892	5.294075	1.91E-07	1.76E-06	6.539459
CYP27A1	−1.406311	2.279413	−5.09686	5.17E-07	4.45E-06	5.582192
CYP1B1	−1.555609	1.575804	−4.896905	1.38E-06	1.07E-05	4.644558
PLA2G4A	−1.207523	2.330584	−4.810888	2.08E-06	1.53E-05	4.251516
DHCR24	1.618315	5.645491	4.74381	2.85E-06	1.99E-05	3.949357
SREBF1	1.077913	4.364146	4.67374	3.96E-06	2.64E-05	3.637801
CAV1	−1.74292	4.514939	−4.442161	1.13E-05	6.55E-05	2.63802
LPCAT1	1.080504	4.092869	4.419695	1.25E-05	7.18E-05	2.543489
FABP4	−2.76082	3.157021	−4.352379	1.68E-05	9.45E-05	2.26286
PLA2G2A	−2.511362	3.427335	−4.277942	2.32E-05	0.000123	1.957142
PLPP2	1.073745	3.546775	3.82306	0.000151	0.000681	0.194956

ALDH3B2	1.275369	3.634809	3.146638	0.001766	0.005848	−2.081017
FABP6	1.283005	2.597581	3.088721	0.00214	0.006909	−2.256367
PLA2G2F	1.438437	2.692196	2.886872	0.004086	0.012321	−2.843078
GDPD3	1.041551	3.337134	2.50197	0.012719	0.033413	−3.855808
